# Divergent Three-Component Assembly of Densely Functionalized Dibenzofurans via ZnCl_2_‑Mediated Cascade Annulation

**DOI:** 10.1021/acs.orglett.6c02234

**Published:** 2026-07-01

**Authors:** Yanan Liu, Qiang Tang, Pui Ying Choy, Yangzilin Kong, Ni Gu, Susuo Lu, Yongjia Shang, Fuk Yee Kwong, Xinwei He

**Affiliations:** † Key Laboratory of Functional Molecular Solids, Ministry of Education, Anhui Laboratory of Molecule-Based Materials (State Key Laboratory Cultivation Base), College of Chemistry and Materials Science, 12514 Anhui Normal University, Wuhu 241000, P. R. China; ‡ The Translational Research Institute for Neurological Disorders & Interdisciplinary Research Center of Neuromedicine and Chemical Biology of Wannan Medical College and Anhui Normal University, Department of Neurosurgery, The First Affiliated Hospital of Wannan Medical College (Yijishan Hospital of Wannan Medical College), Wuhu 241001, P. R. China; § Department of Chemistry and State Key Laboratory of Synthetic Chemistry, 26451 The Chinese University of Hong Kong, Shatin, New Territories, Hong Kong, P. R. China

## Abstract

A ZnCl_2_-promoted three-component cascade ring closure of *o*-hydroxyphenyl propargylamines, pyridinium 1,4-zwitterionic thiolates, and nitroalkanes has been established for the rapid assembly of densely functionalized, non-symmetric tricyclic dibenzofuran frameworks. This step-economical strategy simultaneously constructs the central furan core, peripheral arene moiety, and two ester groups in a single operation. Notably, the reaction proceeds smoothly under ambient air conditions and employs undried nitroalkanes as both a C1 synthon and solvent. The protocol features mild conditions, a broad substrate scope, and excellent functional group compatibility. Moreover, its operational simplicity, scalability, and suitability for late-stage diversification underscore the synthetic utility of this methodology for complex molecular construction.

## Graphical abstract

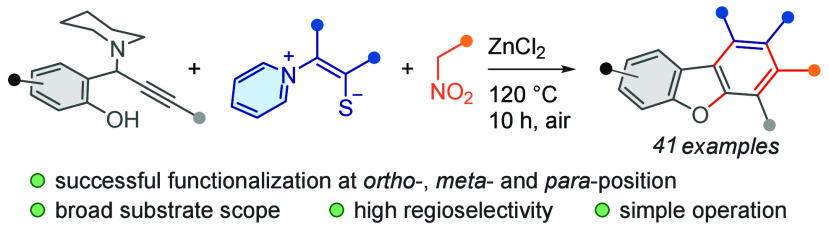

Dibenzofurans are important structural motifs widely embedded in natural products,^1^ pharmaceuticals,^2^ and advanced functional materials.^3^ Their rigid, planar arene–furan-fused architecture not only imparts valuable physicochemical properties but also underpins a broad spectrum of potent biological activities, including antiallergic and anticancer effects.^4^ Thus, dibenzofuran derivatives are extensively explored as promising drug entities, phosphorescent organic light-emitting diodes (PhOLEDs), and specific chemical probes. Despite their versatility, the preparation of densely functionalized dibenzofuran^5^ remains challenging, primarily due to the hurdle in achieving precise regioselective control when installing functional groups across two distinct aryl rings concurrently.

Traditional methods for functionalization of dibenzofuran (Scheme 1A)^6^ via electrophilic substitution or Friedel–Crafts acylation are typically limited for *ortho* and *para* selectivities relative to the oxygen atom, whereas the installation of functional groups, at the same time, at *meta* position is difficult to achieve directly. Additionally, steric hindrance at the “bay region” further complicates site-specific functionalization. While intramolecular ring closure via C–C^7^ or C–O^8,9^ bond formation represents the most common scheme for accessing the central furan core (Scheme 1B), it frequently requires expensive transition metal catalysts or harsh conditions, which would be inferior with sensitive functional groups. Other synthetic strategies,^10^ for instance, the Diels–Alder reaction focusing on benzene ring formation from benzofurans,^11^ contend with competitive addition pathways that lead to undesirable product formation. The cascade annulation process offers an alternative synthetic route, which enables rapid construction of the dibenzofuran core in a single operation.^12^ These methods typically proceed via *in situ* generation of reactive intermediates (e.g., arynes or allenyl ketones) followed by sequential addition and annulation. Nevertheless, several challenges persist, including limited substrate scope (failure with electron-deficient or steric-hindered substrates), poor regiocontrol (especially in assembling highly substituted unsymmetrical systems), and side product formation (polymerization). These limitations render the efficient synthesis of specifically substituted, non-symmetrical dibenzofuran cores a demanding task that requires balancing reactivity with structural precision.

**Scheme 1:**
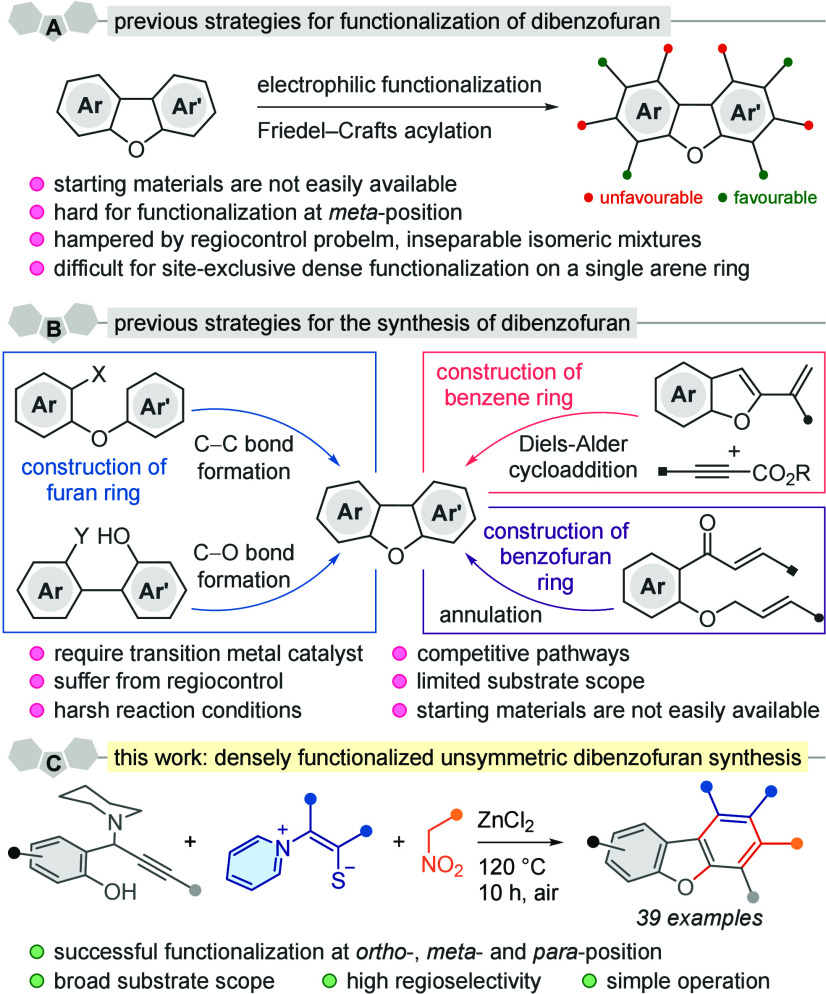
Synthetic Strategies for Accessing Functionalized Dibenzofurans and Our Work

To address these limitations and continuing our interest in the assembly of polycyclic frameworks^13^ and *ortho*-quinone methide (*o*-AQM) chemistry,^14^ we herein report a ZnCl_2_-driven three-component reaction of *o*-hydroxyphenyl propargylamines,^15^ pyridinium 1,4-zwitterionic thiolates,^16^ and readily available nitroalkanes that affords densely functionalized dibenzofurans (Scheme 1C). This protocol utilizes readily available undried nitromethane as a C1 synthon, offering a straightforward, operationally simple route to structurally diverse fused arenes without the need for high-order substrates or laborious multistep procedures.

To commence our study, we first examined the annulation between propargylamine **1a** and pyridinium 1,4-zwitterionic thiolate **2a** (Table 1). Screening of various Lewis acids identified ZnCl_2_ as the most effective choice, delivering dibenzofuran **4a** in 59% yield (Table 1, entries 1–5). Increasing ZnCl_2_ loading to 1.2 equiv improved the yield to 71% (entries 1 vs 6–8). Control experiments revealed that nitromethane plays a dual role as both a solvent and reactant. The reaction was completely shut down when acetonitrile was used as the sole solvent in the absence of nitromethane, whereas a mixture of acetonitrile (2.0 mL) and nitromethane (0.05 mL) gave 29% product yield (entries 7 vs 9–11). Further increasing the nitromethane content in acetonitrile did not improve the yield (entries 10–13), and other solvents with 0.1 mL of nitromethane provided 22% yield or no product (entries 14–17). Subsequent optimization of the reaction concentration, temperature, and time (entries 7 vs 18–22) ultimately furnished **3aa** in 85% yield under the best conditions (entry 22). Employing dried nitromethane did not enhance the yield (entries 22 and 23). Notably, the replacement of the piperidinyl group of **1a** by morpholinyl (**1b**), pyrrolinyl (**1c**), hydroxyl (**1d**), methoxy (**1e**), acetoxy (**1f**), and tosyl (**1g**) groups led to significantly lower yields (entries 22 vs 24–29). The product yield slightly decreased when the reaction was performed under an argon atmosphere (entries 23 vs 30). The structure of **4a** was unambiguously confirmed by single-crystal X-ray analysis (Scheme 2A; CCDC 2556074; see the Supporting Information). Furthermore, the scale-up efficiency of the annulation strategy was demonstrated through a gram-scale experiment, providing **4a** in a satisfactory yield.

**Table 1:**
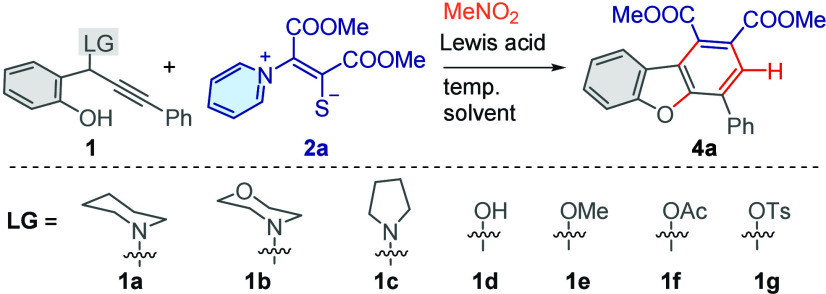
Optimization of Reaction Conditions^a^

entry	substrate	Lewis acid (equiv)	solvent (mL)	yield (%)^b^
1	**1a**	ZnCl_2_ (1.0)	MeNO_2_ (2.0)	59
2	**1a**	ZnI_2_ (1.0)	MeNO_2_ (2.0)	55
3	**1a**	Zn(OAc)_2_ (1.0)	MeNO_2_ (2.0)	trace
4	**1a**	FeCl_3_ (1.0)	MeNO_2_ (2.0)	trace
5	**1a**	AlCl_3_ (1.0)	MeNO_2_ (2.0)	trace
6	**1a**	ZnCl_2_ (0.6)	MeNO_2_ (2.0)	56
7	**1a**	ZnCl_2_ (1.2)	MeNO_2_ (2.0)	71
8	**1a**	ZnCl_2_ (1.4)	MeNO_2_ (2.0)	60
9^c^	**1a**	ZnCl_2_ (1.2)	MeCN (2.0)	n.d.
10	**1a**	ZnCl_2_ (1.2)	MeNO_2_/MeCN (0.03/2.0)	trace
11	**1a**	ZnCl_2_ (1.2)	MeNO_2_/MeCN (0.05/2.0)	29
12	**1a**	ZnCl_2_ (1.2)	MeNO_2_/MeCN (0.1/2.0)	28
13	**1a**	ZnCl_2_ (1.2)	MeNO_2_/MeCN (1.0/1.0)	26
14	**1a**	ZnCl_2_ (1.2)	MeNO_2_/EtOH (0.1/1.0)	22
15	**1a**	ZnCl_2_ (1.2)	MeNO_2_/DMF (0.1/1.0)	n.d.
16	**1a**	ZnCl_2_ (1.2)	MeNO_2_/DMSO (0.1/1.0)	n.d.
17	**1a**	ZnCl_2_ (1.2)	MeNO_2_/toluene (0.1/1.0)	n.d.
18	**1a**	ZnCl_2_ (1.2)	MeNO_2_ (1.5)	74
19	**1a**	ZnCl_2_ (1.2)	MeNO_2_ (2.5)	69
20^d^	**1a**	ZnCl_2_ (1.2)	MeNO_2_ (1.5)	55
21^e^	**1a**	ZnCl_2_ (1.2)	MeNO_2_ (1.5)	70
22^f^	**1a**	ZnCl_2_ (1.2)	MeNO_2_ (1.5)	85
23^f^	**1a**	ZnCl_2_ (1.2)	MeNO_2_ (1.5)^g^	80
24	**1b**	ZnCl_2_ (1.2)	MeNO_2_ (1.5)	49
25	**1c**	ZnCl_2_ (1.2)	MeNO_2_ (1.5)	73
26	**1d**	ZnCl_2_ (1.2)	MeNO_2_ (1.5)	48
27	**1e**	ZnCl_2_ (1.2)	MeNO_2_ (1.5)	n.r.
28	**1f**	ZnCl_2_ (1.2)	MeNO_2_ (1.5)	n.r.
29	**1g**	ZnCl_2_ (1.2)	MeNO_2_ (1.5)	n.r.
30^f^ ^,^ ^h^	**1a**	ZnCl_2_ (1.2)	MeNO_2_ (1.5)	80

a Reaction conditions: propargylamine **1a** (0.1 mmol), pyridinium 1,4-zwitterionic thiolate **2a** (0.1 mmol), Lewis acid (as indicated) in undried MeNO_2_ (as indicated), and solvent (2.0 mL) under an air atmosphere at 120 °C for 12 h.

b Isolated yields. n.d., not determined; n.r., no reaction.

c No MeNO_2_ was added.

d At 100 °C.

e At 110 °C.

f For 10 h.

g Dried MeNO_2_ was used.

h Under an argon atmosphere.

**Scheme 2:**
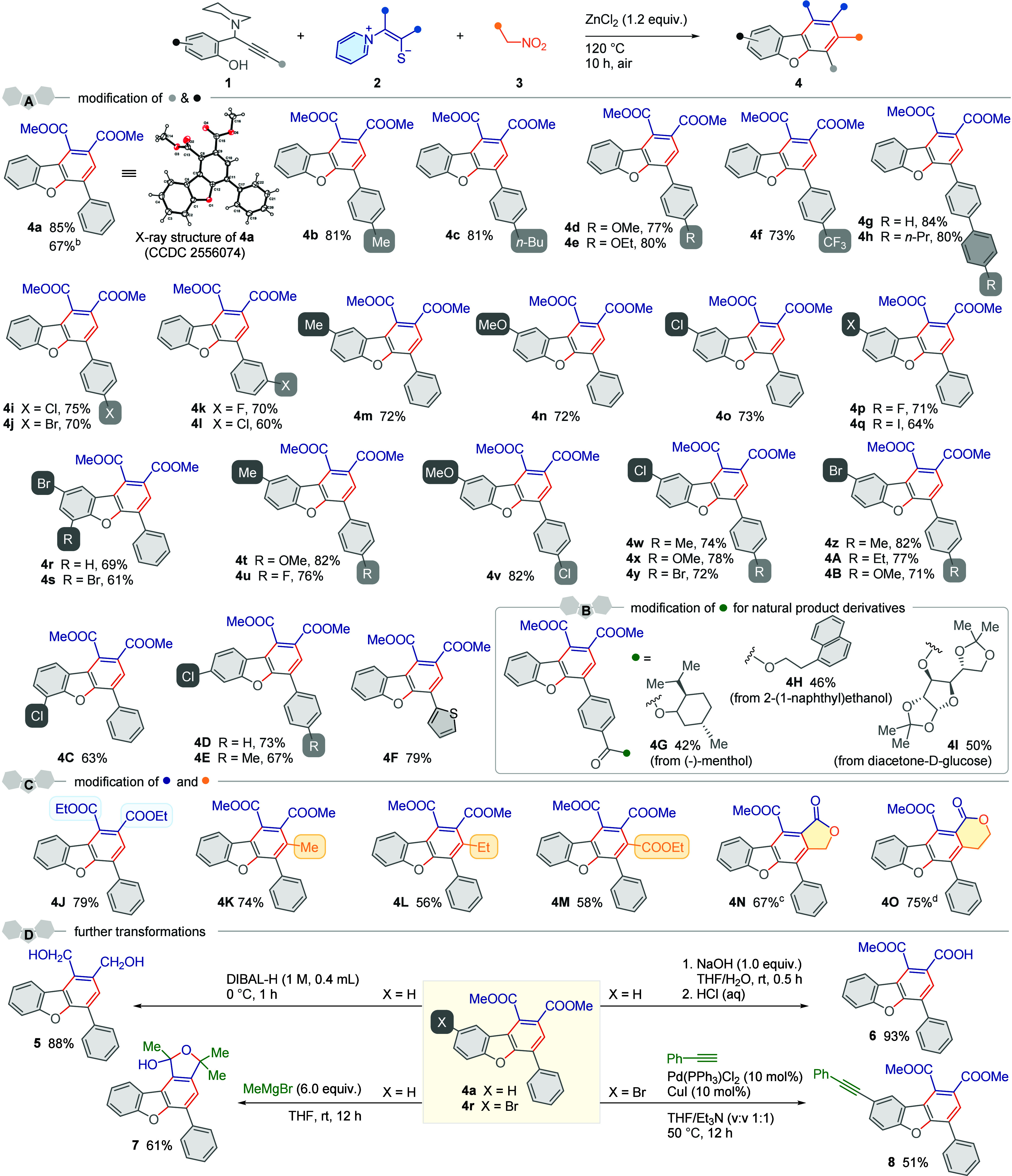
Substrate Scope and Further Transformations^a^

a Reaction conditions: propargylamines **1** (0.2 mmol), pyridinium 1,4-zwitterionic thiolate **2** (0.2 mmol), undried nitroalkanes **3** (3.0 mL), and ZnCl_2_ (0.24 mmol) were stirred at 120 °C under an air atmosphere for 10 h. Isolated yields were reported.
b Gram-scale synthesis.
c 2-Nitroethan-1-ol (3.0 mmol) and MeCN (1.0 mL) were used.
d 3-Nitropropan-1-ol (3.0 mmol) and MeCN (1.0 mL) were used.

Under the optimized reaction conditions, we next conducted a comprehensive evaluation of the substrate scope. First, we examined various propargylamines **1** to access densely functionalized dibenzofuran **4** (Scheme 2A). The reaction demonstrated excellent compatibility with propargylamines bearing electron-neutral (products **4b**, **4c**, **4g**, and **4h**), electron-rich (products **4d** and **4e**), and electron-deficient (products **4f** and **4i**–**4l**) substituents at alkynyl arene, delivering the corresponding dibenzofurans in 70–84% yields. *para*- (products **4m**–**4r**), *meta*- (products **4D** and **4E**) and *ortho*- (product **4C**) substituted propargylamines relative to the hydroxyl group also reacted smoothly, affording desired products in satisfactory yields. Disubstituted propargylamines were capable substrates (products **4s**–**4B**). Of particular note is the fact that halo groups, e.g., −I, −Br, −Cl, and −F groups, located at either phenolic arene or alkynyl arene, remained intact under these reaction conditions (products **4i**–**4l**, **4o**–**4q**, **4s**, and **4u**–**4E**), which allows potential modifications through established cross-coupling strategies at a later stage.^17^ Notably, heteroaryl 2-thienyl-substituted propargylamines also reacted smoothly, yielding the desired dibenzofuran in 79% yield. Remarkably, this protocol proved applicable to complex propargylamine architectures derived from bioactive drug frameworks (Scheme 2B), such as (−)-menthol, 2-(1-naphthyl)­ethanol, and diacetone-d-glucose (products **4G**–**4I**).

In addition to **1**, pyridinium 1,4-zwitterionic thiolates **2** and nitroalkane **3** were probed (Scheme 2C). When the carbomethoxy group of pyridinium 1,4-zwitterionic thiolate **2a** was replaced with a carboethoxy group, the desired product **4J** was obtained in 79% yield. Other nitroalkanes, such as nitroethane and nitropropanes, as well as nitroalkanoate were competent substrates, furnishing the corresponding densely functionalized dibenzofurans smoothly (products **4K**–**4M**). Interestingly, β-nitro alcohols, for instance, 2-nitroethan-1-ol and 3-nitropropan-1-ol, underwent subsequent intramolecular cyclization to give polycyclic dibenzofurans bearing five-membered dihydrofuran-2­(3*H*)-one **4N** and six-membered tetrahydro-2*H*-pyran-2-one **4O** moieties, respectively, in good yields.

To further investigate the synthetic utility of this methodology, polysubstituted dibenzofuran **4a** was subjected to a series of further synthetic transformations (Scheme 2D). The ester handle of **4a** was readily manipulated, undergoing efficient reduction to deliver alcohol **5** in 88% yield and standard saponification to provide carboxylic acid derivative **6** in 93% yield. Exposure of **4a** to magnesium bromide allowed a nucleophilic addition/intramolecular annulation sequence, furnishing tetracyclic structures **7** in 61% yields. Furthermore, dibenzofuran **4r** readily underwent Sonogashira coupling to afford alkynylated dibenzofuran **8** smoothly.

To gain insight into the reaction mechanism, a series of control experiments were conducted (Scheme 3A–C). When propargylamine **9**, which lacks an *ortho*-hydroxyl group, was subjected to the standard conditions, the reaction failed (Scheme 3A). In a similar context, relocation of the hydroxyl group to the *meta* or *para* position on propargylamine **1** did not yield the desired product **4a**, underscoring the critical role of the *ortho*-hydroxyl moiety. Furthermore, when dimethylbut-2-ynedioate was used in place of **2a** under standard conditions, no product was detected. It is worth noting that a multicomponent one-pot, two-step strategy was also attempted, in which the initial reaction between pyridine, elemental sulfur, and dimethylbut-2-ynedioate was followed by the subsequent addition of **1a**, and the desired product **4a** was delivered in 62% yield (Scheme 3B). Finally, when the model reaction was performed in deuterated nitromethane, isotopically labeled product **4a**-*d* was afforded (Scheme 3C). This deuterium-labeling result explicitly confirms that nitromethane functions as the C1 synthon during the construction of the peripheral arene ring.

**Scheme 3:**
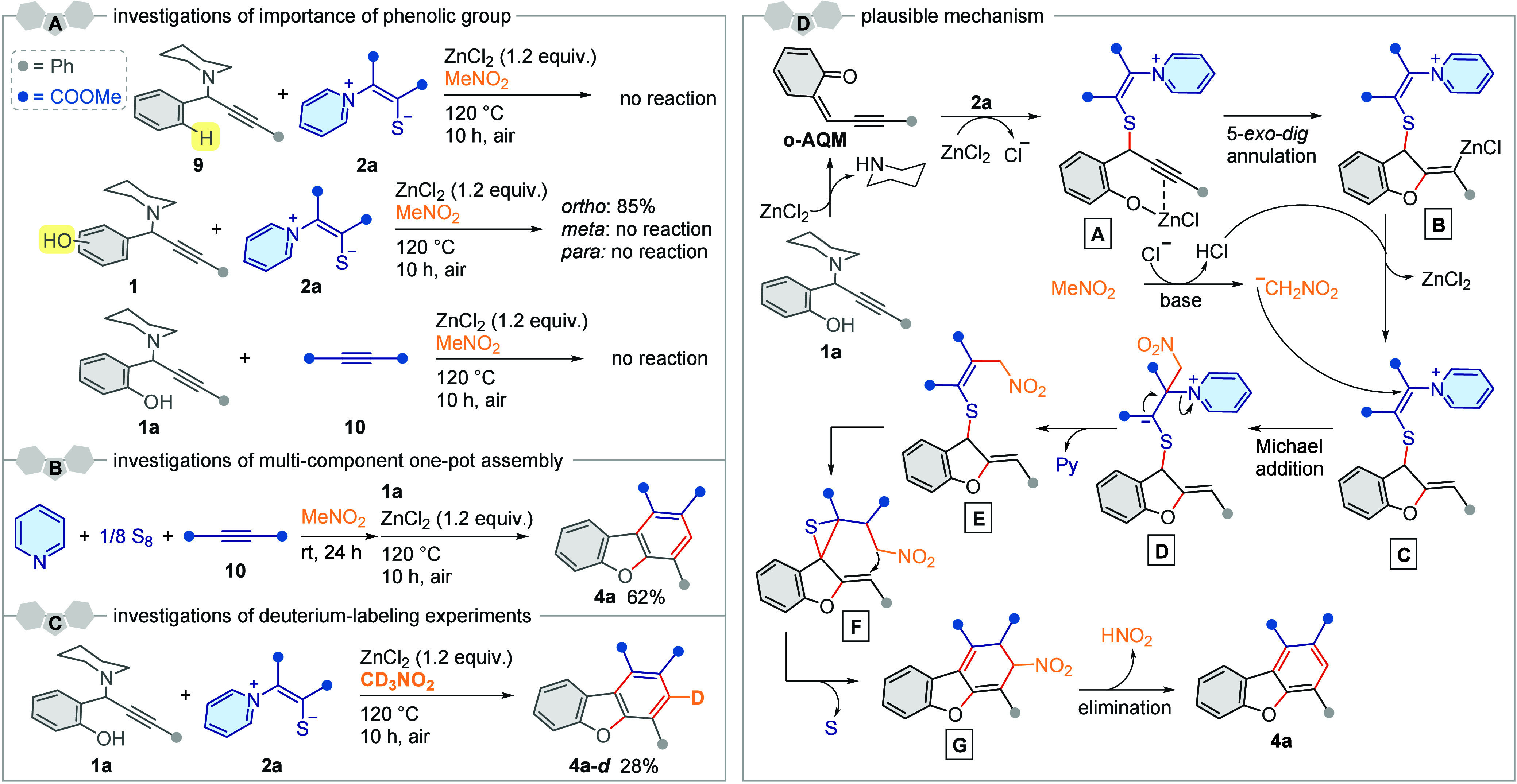
Control Experiment and Proposed Mechanism

On the basis of the control experiments and documented precedents,^18^ a plausible reaction mechanism is proposed (Scheme 3D). Initially, coordination between Lewis acid ZnCl_2_ and *ortho*-hydroxyl propargylamine **1a** promotes the generation of an *ortho*-alkynyl quinone methide (*o*-AQM)^16,19^ intermediate. Subsequently, a 1,4-conjugate addition of pyridinium 1,4-zwitterionic thiolate **2a** to *o*-AQM delivers intermediate **A**, which is further activated by Lewis acid coordination. Intermediate **A** then undergoes a regioselective intramolecular 5-*exo*-*dig* cyclization to furnish benzofuran species **B**. Subsequent protonolysis of the alkenyl–zinc bond transforms **B** into intermediate **C**. Concurrently, nitromethane is deprotonated by piperidine to generate the corresponding nitronate anion, which undergoes addition to intermediate **C**, yielding intermediate **D**. A subsequent elimination/cyclization cascade generates intermediate **F**,^20^ which then afford tricyclic framework **G** through sequential extrusion of elemental sulfur. Finally, elimination of nitrous acid gives the desired dibenzofuran product **4a**.

In conclusion, a robust and efficient ZnCl_2_-driven three-component ring closure protocol has been developed for the modular assembly of structurally diverse, densely functionalized tricyclic dibenzofuran frameworks from *o*-hydroxyphenyl propargylamines, pyridinium 1,4-zwitterionic thiolates, and nitroalkanes. This cascade strategy effectively overcomes traditional synthetic challenges associated with the regioselection, non-symmetrical polysubstitution of the dibenzofuran core by forging the furan and benzene rings concurrently while introducing a versatile ester functionality. Given its broad substrate scope, excellent functional group compatibility, scalability, and capacity for late-stage diversification, this approach constitutes a highly practical advancement for the construction of complex unsymmetrical polysubstituted dibenzofurans, which are privileged scaffolds that are widely found in natural products, pharmaceuticals, and functional materials.

## Supplementary Material



## Data Availability

The data underlying this study are available in the published article and its Supporting Information.

## Notes

Experimental details, procedures, crystal data, characterization of compounds, and ^1^H, ^13^C, and ^19^F NMR spectra and characterization data of the products (PDF)
