# Supplementary material for: Divergent Three-Component Assembly of Densely Functionalized Dibenzofurans via ZnCl2‑Mediated Cascade Annulation
Source: Org Lett. 2026 Jul 1;28(28):8859–65. doi: 10.1021/acs.orglett.6c02234 (PMC13386530; doi:10.1021/acs.orglett.6c02234)
Supplement: Supplementary file 1 [file ol6c02234_si_001.pdf]

Supporting Information

Divergent Three-Component Assembly of Densely Functionalized Dibenzofurans via $ZnCl_2$-Mediated Cascade Annulation

Yanan Liu,[a] Qiang Tang,[a,b] Pui Ying Choy,[c] Yangzilin Kong,[a] Ni Gu,[a] Susuo Lu,[a] Yongjia Shang,*,[a] Fuk Yee Kwong,*,[c] Xinwei He*,[a]

[a]Key Laboratory of Functional Molecular Solids, Ministry of Education, Anhui Laboratory of Molecule-Based Materials (State Key Laboratory Cultivation Base), College of Chemistry and Materials Science, Anhui Normal University, Wuhu 241000, P. R. China.

[b]The Translational Research Institute for Neurological Disorders & Interdisciplinary Research Center of Neuromedicine and Chemical Biology of Wannan Medical College and Anhui Normal University, Department of Neurosurgery, The First Affiliated Hospital of Wannan Medical College (Yijishan Hospital of Wannan Medical College), Wuhu 241001, P.R. China.

[c]Department of Chemistry and State Key Laboratory of Synthetic Chemistry, The Chinese University of Hong Kong, New Territories, Shatin, Hong Kong, P. R. China.

E-mail: shyj@mail.ahnu.edu.cn, fykwong@cuhk.edu.hk, xinweihe@mail.ahnu.edu.cn

Table of contents

1. General Considerations S2
2. General Procedures for Synthesis of Propargylamines **1** S2
3. General Procedures for Reaction Condition Screenings S3
4. General Procedures for $ZnCl_2$-mediated Cascade Annulation Reaction S3
5. General Procedures for gram-scale synthesis of compound **4a** S3
6. General Procedures for Synthesis of Product **5** S4
7. General Procedures for Synthesis of Product **6** S4
8. General Procedures for Synthesis of Product **7** S5
9. General Procedures for Synthesis of Product **8** S5
10. General Procedures for Deuterium Labeling Experiment S6
11. X-ray Crystallographic Data of Product **4a** S7
12. Characterization Data for All Products S8
13. ^{1}H, ^{13}C, and ^{19}F NMR Spectra S26
14. References S76

1. General Considerations

Unless otherwise noted, all reagents were purchased from commercial suppliers and used as received without purification. All cascade reactions were performed in a resealable screw-capped Schlenk flask (approx. 25 mL volume) in the presence of Teflon coated magnetic stirrer bar (4 mm × 10 mm). Thin layer chromatography was conducted on precoated silica gel 60 F_{254} plates. Silica gel (200-400 mesh) was used for column chromatography. Melting points were recorded on an uncorrected instrument. ^{1}H NMR spectra were recorded on a 400 and 500 MHz spectrometer. Spectra were referenced internally to the residual proton resonance in $CDCl_3$ (δ 7.26 ppm) or *d*-DMSO (δ 2.50 ppm). Chemical shifts (δ) were reported as part per million (ppm) in δ scale downfield from TMS . ^{13}C{^{1}H} NMR spectra were recorded on a 100 and 125 MHz spectrometer and the spectra were referenced to $CDCl_3$ (δ 77.16 ppm, the middle peak) or *d*-DMSO (δ 39.5 ppm). Multiplicities are described as s (singlet), d (doublet), t (triplet), q (quartet), or m (multiplet), and the coupling constants (J) are reported in Hertz (Hz). High-resolution mass spectra (HRMS) were obtained by quadrupole time-of-flight mass spectrometer yielded ion mass/charge (m/z) ratios in atomic mass units. Compounds described in the literature were characterized by comparison of their ^{1}H, and/or ^{13}C NMR spectra to the previously reported data.

2. General Procedures for Synthesis of Propargylamines 1

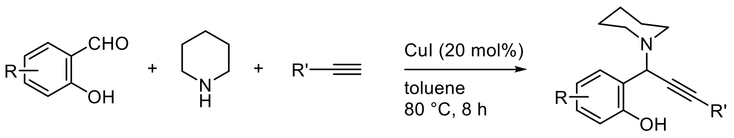

According the reported literature,[1] piperidine (6.5 mmol), aldehyde (5.0 mmol, 1.0 equiv.), acetylene (6.5 mmol), copper(I) iodide (20 mol%) and toluene (10 mL) were added to a 25 mL round-bottom flask equipped with a magnetic stir bar. The mixture was degassed and backfilled with nitrogen, and then stirred in a preheated oil bath at 80 °C for 8 h (monitored by TLC). After the reaction completed (as determined using TLC), the reaction mixture was cooled to room temperature, diluted with dichloromethane (CH_2Cl_2) (10 mL) and filtered through a thin pad of silica gel. The filter cake was washed with CH_2Cl_2, and the combined filtrate was concentrated in vacuum. The crude product was purified by flash column chromatography on silica gel to afford the corresponding propargylamines **1**. All the substrates **1** were known compounds.

3. General Procedures for Reaction Condition Screenings

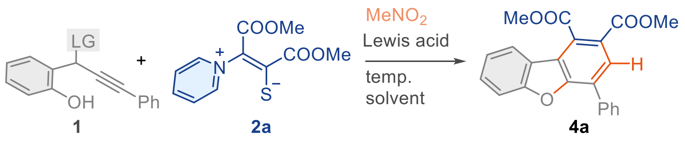

Propargylamine **1** (0.2 mmol, 1.0 equiv.), 1,4-dimethoxy-1,4-dioxo-3-(pyridin-1-ium-1-yl)but-2-ene-2-thiolate (**2a**) (1.0 equiv.), Lewis acid (as indicated in Table 1), nitromethane (**3a**) (as indicated in Table 1) and solvent (as indicated in Table 1) were loaded to Schlenk tube with a Teflon-coated magnetic stir bar. The tube was then placed into a pre-heated oil bath (indicated in Table 1) under air atmosphere and stirred for the duration as indicated. The reaction tube was allowed to cool to room temperature. CH_2Cl_2 (~3 x 10 mL), and brine were added for extraction. The organic layer was purified by flash column chromatography with ethyl acetate and petroleum ether as the elution solvent to give desired products **4a**.

4. General Procedures for $ZnCl_2$-mediated Cascade Annulation Reaction

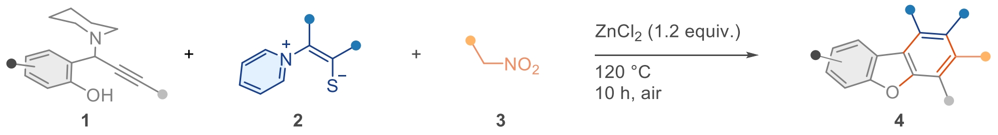

Propargylamine **1** (0.2 mmol, 1.0 equiv.), pyridinium 1,4-zwitterionic thiolate **2** (1.0 equiv.), $ZnCl_2$ (1.2 equiv.), and nitroalkanes **3** (3.0 mL) were loaded to Schlenk tube with a Teflon-coated magnetic stir bar. The tube was then placed into a pre-heated oil bath at 120 °C and stirred for 10 h under air atmosphere. The reaction tube was allowed to cool to room temperature. CH_2Cl_2 (~3 x 10 mL), and brine were added for extraction. The organic layer was purified by flash column chromatography with ethyl acetate and petroleum ether as the elution solvent to give desired products **4**.

5. General Procedures for gram-scale synthesis of compound 4a

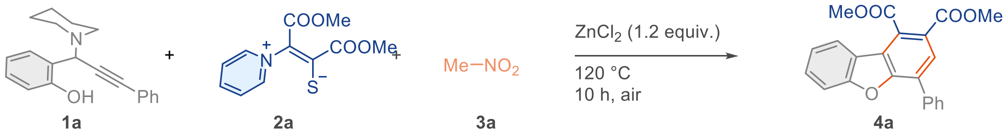

Propargylamine **1a** (5.0 mmol, 1.0 equiv.), 1,4-dimethoxy-1,4-dioxo-3-(pyridin-1-ium-1-yl)but-2-ene-2-thiolate (**2a**) (1.0 equiv.), $ZnCl_2$ (1.2 equiv.), and nitromethane (**3a**) (10.0 mL) were loaded to Schlenk tube with a Teflon-coated magnetic stir bar. The tube was then placed into a pre-heated oil

bath at 120 °C and stirred for 10 h under air atmosphere. The reaction tube was allowed to cool to room temperature. CH_2Cl_2, and brine were added for extraction. The organic layer was purified by flash column chromatography with ethyl acetate and petroleum ether as the elution solvent to give desired products **4a** in 67% (1.21 g).

6. General Procedures for Synthesis of Product 5

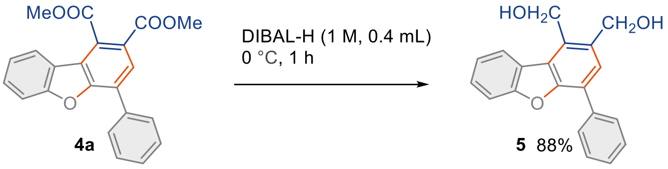

An oven-dried reaction flask was equipped with a Teflon-coated magnetic stir bar, was charged compound **4a** (0.2 mmol, 1.0 equiv.). Tetrahydrofuran (THF) (2.0 mL) was added, and the solution was cooled to 0 °C in ice-water bath. Diisobutylaluminum hydride (DIBAL-H) (1.0 M, 0.4 mL) was added to the reaction mixture dropwise slowly. Upon completion of addition, the reaction mixture was kept at 0 °C and stirred until all of the raw materials were consumed (monitored by TLC). After the reaction completed (as determined using TLC), the crude products were extracted with CH_2Cl_2 (3 × 20 mL) and washed with brine. The organic layers were combined, dried over Na_2SO_4, filtered, and then evaporated under vacuum. The residue was recrystallized by acetone to give the product **5** as a white solid in 88% yield (54 mg).

7. General Procedures for Synthesis of Product 6

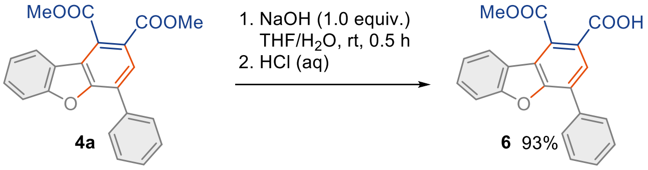

According to the reported literature,[2] to a stirred solution of compound **4a** (0.2 mmol) in THF (1.0 mL), NaOH (1M, 1.0 mL) was added dropwise and stirred at room temperature for 0.5 h. After the reaction completed (monitored by TLC), the reaction was quenched with diluted HCl and acidified until pH = 2. The crude products were extracted with CH_2Cl_2, water and washed with brine. The organic layers were combined, dried over Na_2SO_4, filtered, and then evaporated under vacuum. The residue was recrystallized by acetone to give the product **6** as a white solid in 93% yield (64 mg).

8. General Procedures for Synthesis of Product 7

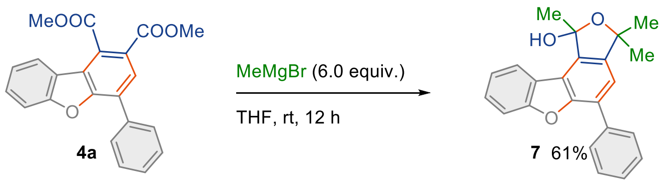

According to the reported literature,[3] an oven-dried Schlenk tube was equipped with a Teflon-coated magnetic stir bar, was charged compound **4a** (0.2 mmol, 1.0 equiv.). Anhydrous tetrahydrofuran (THF) (2.0 mL) was added. Then phenylmagnesium bromide (6.0 equiv.) was added dropwise slowly at room temperature and stirred for 12 h. After completion of the reaction (monitored by TLC analysis), the reaction was quenched with saturated ammonium chloride solution and extracted with CH_2Cl_2, and washed with brine. The organic layers were combined, dried over Na_2SO_4, filtered, and then evaporated under vacuum. The residue was purified by flash column chromatography on silica gel (200-300 mesh) using ethyl acetate and petroleum ether (1:10, v/v) as the elution solvent to give the product **7** as white solid in 61% yield (42 mg).

9. General Procedures for Synthesis of Product 8

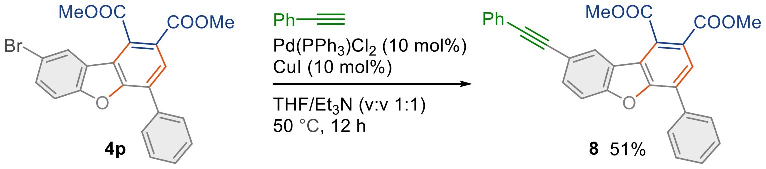

According to the reported literature,[4] an oven-dried Schlenk tube was equipped with a Teflon-coated magnetic stir bar and reflux condenser, was charged $Pd(PPh_3)_2Cl_2$ (10 mmol%), CuI (10 mmol%), phenylacetylene (2.0 equiv), and compound **4p** (0.2 mmol, 1.0 equiv.) under an argon atmosphere. Tetrahydrofuran (THF) (1.5 mL) and Et_3N (1.5 mL) were added. The tube was then placed into a pre-heated oil bath at 50 °C and stirred for 12 h under argon atmosphere. After completion of the reaction (monitored by TLC analysis), the reaction was quenched with brine and extracted with CH_2Cl_2. The organic layers were combined, dried over Mg_2SO_4, filtered, and then evaporated under vacuum. The residue was purified by flash column chromatography on silica gel (200-300 mesh) using ethyl acetate and petroleum ether (1:12, v/v) as the elution solvent to give the product **8** as a white solid in 51% yield (47 mg).

10. General Procedures for Deuterium Labeling Experiment

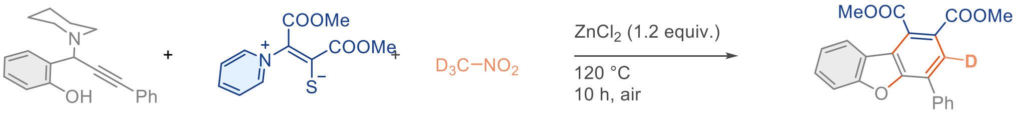

Propargylamine **1a** (0.2 mmol, 1.0 equiv.), 1,4-dimethoxy-1,4-dioxo-3-(pyridin-1-ium-1-yl)but-2-ene-2-thiolate (**2a**) (1.0 equiv.), $ZnCl_2$ (1.2 equiv.), and CD_3NO_2 (**3a-*d*₃**) (3.0 mL) were loaded to Schlenk tube with a Teflon-coated magnetic stir bar. The tube was then placed into a pre-heated oil bath at 120 °C and stirred for 10 h under air atmosphere. The reaction tube was allowed to cool to room temperature. CH_2Cl_2, and brine were added for extraction. The organic layer was purified by flash column chromatography with ethyl acetate and petroleum ether as the elution solvent to give desired products **4a-*d*** as a white solid in 28% (20 mg).

11. X-ray Crystallographic Data of Product 4a

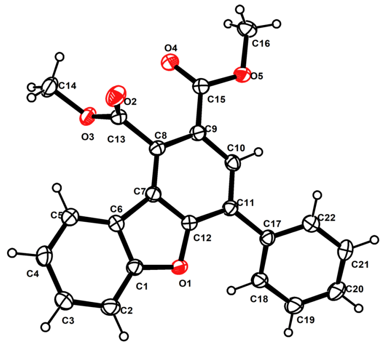

The purified compound **4a** is dissolved in a mixed solvent of ethyl acetate and petroleum ether, and placed in a dark cabinet to slowly evaporate. After several days, a colourless bulk crystal was obtained. The X-ray crystal-structure determinations were obtained on a Bruker Smart CCDC APEX-2 diffractometer (graphite- monochromated Mo *Kα* radiation, *λ*=0.71073 nm) at 298 K.

Figure S1. ORTEP drawing of product **4a** (CCDC 2556074) (The ellipsoid contour 30% probability levels)

Table S3. Crystal data and structure refinement for product **4a**

CCDC number	2556074	
Identification code	240718c	
Empirical formula	C22 H16 O5	
Formula weight	360.35	
Temperature	298 K	
Radiation	MoKα (λ = 0.71073 Å)	
Crystal system	triclinic	
Space group	P-1	
Unit cell dimensions	a = 9.905(3) Å	α= 100.671(4)°
	b = 10.721(3) Å	β= 98.921(4)°
	c = 18.666(5) Å	γ= 112.459(4)°
Volume	1743.6(9) $Å^3$	
Z	4	
Density (calculated)	1.373 g/cm^3	
μ	0.098 mm^{-1}	
F(000)	752.0	
Crystal size	0.15 × 0.13 × 0.12 mm^3	
2Θ range for data collection	2.292 to 52.394°	
Index ranges	-12≤h≤12, -13≤k≤11, -23≤l≤16	
Reflections collected	9677	
Independent reflections	6872[R_{int} = 0.0251, R_{sigma} = 0.0596]	
Data/restraints/parameters	6872 / 0 / 492	
Goodness-of-fit on F^2	1.039	
Final R indices [I>2σ (I)]	R_1 = 0.0552, wR_2 = 0.1406	
Final R indices (all data)	R_1 = 0.0890, wR_2 = 0.1585	
Largest diff. peak/hole	0.25 and -0.26 e.$Å^{-3}$	

12. Characterization Data for All Products

Dimethyl 4-phenyldibenzo[*b,d*]furan-1,2-dicarboxylate (product 4a)

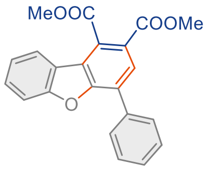

This compound was purified by column chromatography (ethyl acetate/petroleum ether = 1:10, R_f = 0.6) to afford a white solid in 85% yield (61 mg); mp 139-140 °C; **^{1}H NMR** (400 MHz, $CDCl_3$) δ 8.28 (s, 1H), 7.91 (d, *J* = 7.4 Hz, 3H), 7.63 (d, *J* = 8.2 Hz, 1H), 7.60 – 7.53 (m, 3H), 7.51 – 7.44 (m, 1H), 7.39 (t, *J* = 7.6 Hz, 1H), 4.14 (s, 3H), 3.98 (s, 3H); **^{13}C{^{1}H} NMR** (125 MHz, $CDCl_3$) δ 168.8, 166.3, 157.0, 155.5, 134.9, 129.1, 129.0, 128.9, 128.8, 128.2, 127.0, 123.8, 123.3, 122.8, 122.2, 122.1, 112.2, 53.2, 52.8; **HRMS** (ESI-TOF) *m/z*: $[M+H]^+$ calcd for $C_{22}H_{17}O_5$ 361.1071; found 361.1072.

Dimethyl 4-(*p*-tolyl)dibenzo[*b,d*]furan-1,2-dicarboxylate (product 4b)

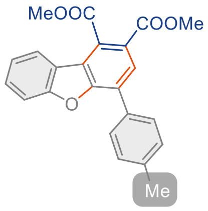

This compound was purified by column chromatography (ethyl acetate/petroleum ether = 1:10, R_f = 0.6) to afford a white solid in 81% yield (60 mg); mp 183-184 °C; **^{1}H NMR** (400 MHz, $CDCl_3$) δ 8.26 (s, 1H), 7.90 (d, *J* = 7.9 Hz, 1H), 7.81 (d, *J* = 8.1 Hz, 2H), 7.62 (d, *J* = 8.2 Hz, 1H), 7.58 – 7.49 (m, 1H), 7.43 – 7.34 (m, 3H), 4.14 (s, 3H), 3.98 (s, 3H), 2.46 (s, 3H); **^{13}C{^{1}H} NMR** (100 MHz, $CDCl_3$) δ 168.8, 166.4, 157.0, 155.5, 138.8, 132.0, 129.7, 128.9, 128.7, 128.6, 127.9, 127.0, 123.8, 123.2, 122.7, 122.2 (2C), 112.2, 53.2, 52.8, 21.5; **HRMS** (ESI-TOF) *m/z*: $[M+H]^+$ calcd for $C_{23}H_{19}O_5$ 375.1227; found 375.1222.

Dimethyl 4-(4-butylphenyl)dibenzo[*b,d*]furan-1,2-dicarboxylate (product 4c)

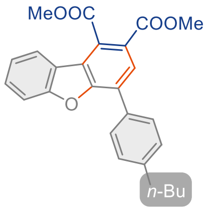

This compound was purified by column chromatography (ethyl acetate/petroleum ether = 1:10, R_f = 0.6) to afford a white solid in 81% yield (67 mg); mp 108-109 °C; **^{1}H NMR** (500 MHz, $CDCl_3$) δ 8.27 (s, 1H), 7.93 – 7.88 (m, 1H), 7.83 (d, *J* = 8.2 Hz, 2H), 7.63 (d, *J* = 8.2 Hz, 1H), 7.57 – 7.50 (m, 1H),

7.41 – 7.36 (m, 3H), 4.14 (s, 3H), 3.98 (s, 3H), 2.75 – 2.70 (m, 2H), 1.72 – 1.66 (m, 2H), 1.47 – 1.39 (m, 2H), 0.98 (t, *J* = 7.4 Hz, 3H); ^{13}**C{^{1}H} NMR** (100 MHz, $CDCl_3$) δ 168.8, 166.4, 157.0, 155.5, 143.8, 132.2, 129.1, 128.9, 128.7, 128.6, 127.9, 127.0, 123.8, 123.2, 122.7, 122.2 (2C), 112.2, 53.2, 52.8, 35.6, 33.7, 22.5, 14.1; **HRMS** (ESI-TOF) *m/z*: $[M+H]^+$ calcd for $C_{26}H_{25}O_5$ 417.1697; found 417.1688.

Dimethyl 4-(4-methoxyphenyl)dibenzo[*b,d*]furan-1,2-dicarboxylate (product 4d)

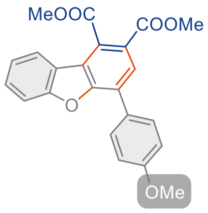

This compound was purified by column chromatography (ethyl acetate/petroleum ether = 1:10, R_f = 0.6) to afford a white solid in 77% yield (60 mg); mp 163-164 °C; ^{1}**H NMR** (400 MHz, $CDCl_3$) δ 8.23 (s, 1H), 7.93 – 7.83 (m, 3H), 7.62 (d, *J* = 8.3 Hz, 1H), 7.57 – 7.49 (m, 1H), 7.38 (s, 1H), 7.13 – 7.05 (m, 2H), 4.14 (s, 3H), 3.98 (s, 3H), 3.90 (s, 3H); ^{13}**C{^{1}H} NMR** (100 MHz, $CDCl_3$) δ 168.8, 166.4, 160.1, 157.0, 155.3, 130.2, 128.7, 128.2, 127.5, 127.2, 126.6, 123.8, 123.3, 122.7, 122.2 (2C), 114.4, 112.1, 55.5, 53.2, 52.8; **HRMS** (ESI-TOF) *m/z*: $[M+H]^+$ calcd for $C_{23}H_{19}O_6$ 391.1176; found 391.1180.

Dimethyl 4-(4-ethoxyphenyl)dibenzo[*b,d*]furan-1,2-dicarboxylate (product 4e)

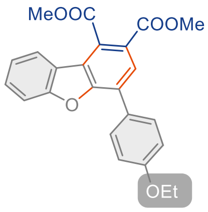

This compound was purified by column chromatography (ethyl acetate/petroleum ether = 1:10, R_f = 0.6) to afford a white solid in 80% yield (65 mg); mp 207-208 °C; ^{1}**H NMR** (500 MHz, $CDCl_3$) δ 8.23 (s, 1H), 7.90 (d, *J* = 7.8 Hz, 1H), 7.86 (d, *J* = 8.3 Hz, 2H), 7.63 (d, *J* = 8.3 Hz, 1H), 7.53 (t, *J* = 7.8 Hz, 1H), 7.38 (t, *J* = 7.6 Hz, 1H), 7.08 (d, *J* = 8.3 Hz, 2H), 4.17 – 4.11 (m, 5H), 3.97 (s, 3H), 1.48 (t, *J* = 7.0 Hz, 3H); ^{13}**C{^{1}H} NMR** (100 MHz, $CDCl_3$) δ 168.8, 166.5, 159.5, 157.0, 155.4, 130.2, 128.7, 128.2, 127.5, 127.1, 126.7, 123.8, 123.3, 122.7, 122.2 (2C), 115.0, 112.2, 63.7, 53.2, 52.8, 15.0; **HRMS** (ESI-TOF) *m/z*: $[M+H]^+$ calcd for $C_{24}H_{21}O_6$ 405.1333; found 405.1342.

Dimethyl 4-(4-(trifluoromethyl)phenyl)dibenzo[*b,d*]furan-1,2-dicarboxylate (product 4f)

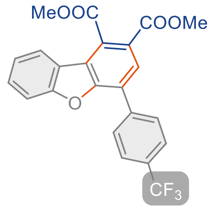

This compound was purified by column chromatography (ethyl acetate/petroleum ether = 1:10, R_f = 0.6) to afford a white solid in 73% yield (62 mg); mp 190-191 °C; **^{1}H NMR** (500 MHz, $CDCl_3$) δ 8.28 (s, 1H), 8.03 (d, *J* = 8.1 Hz, 2H), 7.90 (d, *J* = 7.9 Hz, 1H), 7.82 (d, *J* = 8.2 Hz, 2H), 7.63 (d, *J* = 8.3 Hz, 1H), 7.59 - 7.52 (m, 1H), 7.41 (dd, *J* = 8.1, 7.1 Hz, 1H), 4.15 (s, 3H), 3.99 (s, 3H); **^{13}C{^{1}H} NMR** (100 MHz, $CDCl_3$) δ 168.5, 166.1, 157.0, 155.4, 138.5, 130.7 (q, J_{C-F} = 32.5 Hz), 129.4, 129.1 (q, J_{C-F} = 3.4 Hz), 128.8, 125.9 (q, J_{C-F} = 3.7 Hz), 125.3, 124.2 (q, J_{C-F} = 270.6 Hz), 124.1, 123.4, 123.1, 122.3, 121.9, 112.2, 53.3, 52.9; **^{19}F NMR** (470 MHz, $CDCl_3$) δ -62.6; **HRMS** (ESI-TOF) *m/z*: $[M+H]^+$ calcd for $C_{23}H_{16}F_3O_5$ 429.0944; found 429.0946.

Dimethyl 4-([1,1'-biphenyl]-4-yl)dibenzo[*b,d*]furan-1,2-dicarboxylate (product 4g)

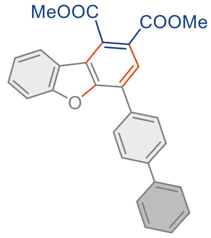

This compound was purified by column chromatography (ethyl acetate/petroleum ether = 1:10, R_f = 0.6) to afford a white solid in 84% yield (73 mg); mp 197-198 °C; **^{1}H NMR** (500 MHz, $CDCl_3$) δ 8.33 (s, 1H), 8.01 (d, *J* = 8.3 Hz, 2H), 7.92 (d, *J* = 7.8 Hz, 1H), 7.83 – 7.78 (m, 2H), 7.72 – 7.63 (m, 3H), 7.53 (m, *J* = 26.4, 8.1 Hz, 3H), 7.44 – 7.37 (m, 2H), 4.15 (d, *J* = 0.8 Hz, 3H), 3.99 (d, *J* = 0.9 Hz, 3H); **^{13}C{^{1}H} NMR** (100 MHz, $CDCl_3$) δ 168.7, 166.3, 157.0, 155.5, 141.6, 140.6, 133.8, 129.4, 129.0, 128.8, 128.6, 128.2, 127.8, 127.7, 127.3, 126.5, 123.9, 123.4, 122.9, 122.3, 122.1, 112.2, 53.2, 52.8; **HRMS** (ESI-TOF) *m/z*: $[M+H]^+$ calcd for $C_{28}H_{21}O_5$ 437.1384; found 437.1383.

Dimethyl 4-(4'-propyl-[1,1'-biphenyl]-4-yl)dibenzo[*b,d*]furan-1,2-dicarboxylate (product 4h)

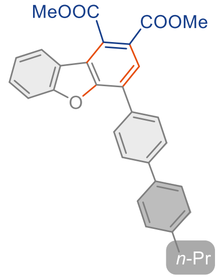

This compound was purified by column chromatography (ethyl acetate/petroleum ether = 1:10, R_f = 0.6) to afford a white solid in 80% yield (76 mg); mp 168-169 °C; **^{1}H NMR** (400 MHz, $CDCl_3$) δ 8.35 (s, 1H), 8.02 (d, J = 8.4 Hz, 2H), 7.95 (d, J = 7.9 Hz, 1H), 7.81 (d, J = 8.4 Hz, 2H), 7.72 – 7.62 (m, 3H), 7.57 (t, J = 7.8 Hz, 1H), 7.43 (t, J = 7.6 Hz, 1H), 7.33 (d, J = 7.9 Hz, 2H), 4.18 (s, 3H), 4.02 (s, 3H), 2.69 (t, J = 7.7 Hz, 2H), 1.78 – 1.73 (m, 2H), 1.03 (t, J = 7.4 Hz, 3); **^{13}C{^{1}H} NMR** (100 MHz, $CDCl_3$) δ 168.7, 166.4, 157.0, 155.5, 142.5, 141.6, 138.0, 133.5, 129.4, 129.2, 128.8, 128.6, 128.1, 127.5, 127.1, 126.6, 123.9, 123.3, 122.8, 122.3, 122.1, 112.2, 53.2, 52.8, 37.9, 24.7, 14.0; **HRMS** (ESI-TOF) *m/z*: $[M+H]^+$ calcd for $C_{31}H_{27}O_5$ 479.1853; found 479.1854.

Dimethyl 4-(4-chlorophenyl)dibenzo[*b,d*]furan-1,2-dicarboxylate (product 4i)

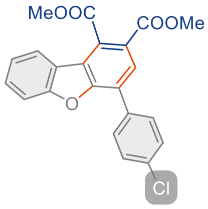

This compound was purified by column chromatography (ethyl acetate/petroleum ether = 1:10, R_f = 0.6) to afford a white solid in 75% yield (59 mg); mp 169-170 °C; **^{1}H NMR** (400 MHz, $CDCl_3$) δ 8.23 (s, 1H), 7.90 (d, J = 7.8 Hz, 1H), 7.85 (d, J = 8.6 Hz, 2H), 7.63 (d, J = 8.2 Hz, 1H), 7.58 – 7.49 (m, 3H), 7.44 – 7.35 (m, 1H), 4.14 (s, 3H), 3.98 (s, 3H); **^{13}C{^{1}H} NMR** (100 MHz, $CDCl_3$) δ 168.6, 166.2, 157.0, 155.3, 134.9, 133.3, 130.3, 129.2, 128.9, 128.5, 128.5, 125.7, 124.0, 123.3, 122.9, 122.3, 122.0, 112.2, 53.2, 52.9; **HRMS** (ESI-TOF) *m/z*: $[M+H]^+$ calcd for $C_{22}H_{16}ClO_5$ 395.0681; found 395.0688.

Dimethyl 4-(4-bromophenyl)dibenzo[*b,d*]furan-1,2-dicarboxylate (product 4j)

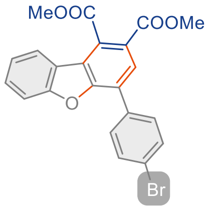

This compound was purified by column chromatography (ethyl acetate/petroleum ether = 1:10, R_f = 0.6) to afford a white solid in 70% yield (61 mg); mp 196-197 °C; **^{1}H NMR** (400 MHz, $CDCl_3$) δ 8.23 (s, 1H), 7.89 (d, J = 7.8 Hz, 1H), 7.81 – 7.75 (m, 2H), 7.68 (d, J = 8.5 Hz, 2H), 7.62 (d, J = 8.4 Hz, 1H), 7.53 (t, J = 7.8 Hz, 1H), 7.39 (t, J = 7.6 Hz, 1H), 4.14 (s, 3H), 3.98 (s, 3H); **^{13}C{^{1}H} NMR** (100 MHz, $CDCl_3$) δ 168.6, 166.2, 157.0, 155.3, 133.8, 132.2, 130.6, 128.9, 128.5, 128.4, 125.7, 124.0, 123.4, 123.1, 123.0, 122.3, 122.0, 112.2, 53.2, 52.9; **HRMS** (ESI-TOF) *m/z*: $[M+H]^+$ calcd for $C_{22}H_{16}BrO_5$ 439.0176; found 439.0186.

Dimethyl 4-(3-fluorophenyl)dibenzo[*b,d*]furan-1,2-dicarboxylate (product 4k)

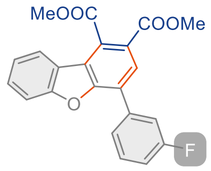

This compound was purified by column chromatography (ethyl acetate/petroleum ether = 1:10, R_f = 0.6) to afford a white solid in 70% yield (53 mg); mp 164-165 °C; **^{1}H NMR** (500 MHz, $CDCl_3$) δ 8.27 (s, 1H), 7.92 – 7.87 (m, 1H), 7.72 – 7.68 (m, 1H), 7.68 – 7.62 (m, 2H), 7.59 – 7.49 (m, 2H), 7.44 – 7.37 (m, 1H), 7.22 – 7.14 (m, 1H), 4.14 (s, 3H), 3.98 (s, 3H); **^{13}C NMR** (125 MHz, $CDCl_3$) δ 168.6, 166.2, 163.1 (d, $J_{C\text{-}F}$ = 244.4 Hz), 157.0, 155.4, 137.0 (d, $J_{C\text{-}F}$ = 8.1 Hz), 130.5 (d, $J_{C\text{-}F}$ = 8.3 Hz), 129.0, 128.7, 128.6, 125.5, 124.7 (d, $J_{C\text{-}F}$ = 3.1 Hz), 124.0, 123.3, 123.0, 122.2, 122.0, 116.0 (d, $J_{C\text{-}F}$ = 22.1 Hz), 115.7 (d, $J_{C\text{-}F}$ = 20.9 Hz), 112.2, 53.3, 52.9; **^{19}F NMR** (376 MHz, $CDCl_3$) δ -112.4. **HRMS** (ESI-TOF) *m/z*: $[M+H]^+$ calcd for $C_{22}H_{16}FO_5$ 379.0976, found 379.0970.

Dimethyl 4-(3-chlorophenyl)dibenzo[*b,d*]furan-1,2-dicarboxylate (product 4l)

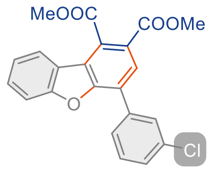

This compound was purified by column chromatography (ethyl acetate/petroleum ether = 1:10, R_f = 0.6) to afford a white solid in 59% yield (47 mg); mp 170-171 °C; **^{1}H NMR** (500 MHz, $CDCl_3$) δ 8.25 (s, 1H), 7.92 – 7.87 (m, 2H), 7.83 – 7.77 (m, 1H), 7.68 – 7.63 (m, 1H), 7.59 – 7.52 (m, 1H), 7.52 – 7.48 (m, 1H), 7.48 – 7.37 (m, 2H), 4.14 (s, 3H), 3.99 (s, 3H); **^{13}C NMR** (125 MHz, $CDCl_3$) δ 168.6, 166.1, 157.0, 155.4, 136.7, 134.9, 130.2, 129.0, 128.9 (2C), 128.8, 128.7, 127.2, 125.4, 124.0, 123.3, 123.0, 122.3, 122.0, 112.3, 53.3, 52.9; **HRMS** (ESI-TOF) *m/z*: $[M+H]^+$ calcd for $C_{22}H_{16}ClO_5$ 395.0681, found 395.0690.

Dimethyl 8-methyl-4-phenyldibenzo[*b,d*]furan-1,2-dicarboxylate (product 4m)

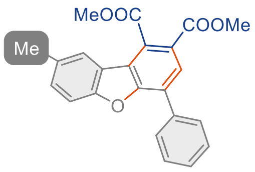

This compound was purified by column chromatography (ethyl acetate/petroleum ether = 1:10, R_f = 0.6) to afford a white solid in 72% yield (54 mg); mp 160-161 °C; **^{1}H NMR** (400 MHz, $CDCl_3$) δ 8.26 (s, 1H), 7.94 – 7.87 (m, 2H), 8.26 (s, 1H), 7.56 (t, J = 7.5 Hz, 2H), 7.52 – 7.45 (m, 2H), 7.33 (dd, J = 8.5, 1.8 Hz, 1H), 4.15 (s, 3H), 3.98 (s, 3H), 2.51 (s, 3H); **^{13}C{^{1}H} NMR** (100 MHz, $CDCl_3$) δ 168.9, 166.4, 155.8, 155.4, 135.0, 133.4, 129.9, 129.0, 128.9, 128.7, 128.6, 128.2, 126.8, 122.9, 122.7, 122.1

(2C), 111.7, 53.2, 52.8, 21.7; **HRMS** (ESI-TOF) *m/z*: $[M+H]^+$ calcd for $C_{23}H_{19}O_5$ 375.1227; found 375.1224.

Dimethyl 8-methoxy-4-phenyldibenzo[*b,d*]furan-1,2-dicarboxylate (product 4n)

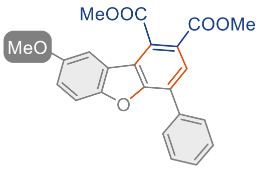

This compound was purified by column chromatography (ethyl acetate/petroleum ether = 1:6, R_f = 0.6) to afford a white solid in 72% yield (56 mg); mp 133-134 °C; **^{1}H NMR** (500 MHz, $CDCl_3$) δ 8.25 (s, 1H), 7.92 – 7.86 (m, 2H), 7.59 – 7.44 (m, 4H), 7.35 (d, *J* = 2.6 Hz, 1H), 7.11 (dd, *J* = 9.0, 2.7 Hz, 1H), 4.14 (s, 3H), 3.98 (s, 3H), 3.90 (s, 3H); **^{13}C NMR** (100 MHz, $CDCl_3$) δ 168.7, 166.4, 156.4, 156.2, 151.8, 134.9, 129.0, 128.9, 128.7, 128.6, 128.1, 126.9, 123.0, 122.9, 122.6, 116.8, 112.6, 105.1, 56.0, 53.1, 52.8; **HRMS** (ESI-TOF) *m/z*: $[M+H]^+$ calcd for $C_{23}H_{19}O_6$ 391.1176; found 391.1182.

Dimethyl 8-chloro-4-phenyldibenzo[*b,d*]furan-1,2-dicarboxylate (product 4o)

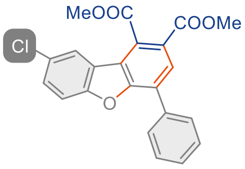

This compound was purified by column chromatography (ethyl acetate/petroleum ether = 1:10, R_f = 0.6) to afford a white solid in 73% yield (58 mg); mp 167-168 °C; **^{1}H NMR** (400 MHz, $CDCl_3$) δ 8.27 (s, 1H), 7.91 – 7.85 (m, 3H), 7.59 – 7.53 (m, 3H), 7.51 – 7.45 (m, 2H), 4.14 (s, 3H), 3.98 (s, 3H); **^{13}C{^{1}H} NMR** (100 MHz, $CDCl_3$) δ 168.3, 166.2, 155.9, 155.3, 134.6, 129.4, 129.3, 129.0, 128.9, 128.2, 127.3, 123.9, 123.5, 122.1, 122.0, 113.2, 53.4, 52.9; **HRMS** (ESI-TOF) *m/z*: $[M+H]^+$ calcd for $C_{22}H_{16}ClO_5$ 395.0681; found 395.0681.

Dimethyl 8-fluoro-4-phenyldibenzo[*b,d*]furan-1,2-dicarboxylate (product 4p)

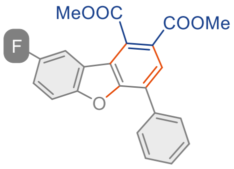

This compound was purified by column chromatography (ethyl acetate/petroleum ether = 1:10, R_f = 0.6) to afford a white solid in 71% yield (54 mg); mp 158-159 °C; **^{1}H NMR** (400 MHz, $CDCl_3$) δ 8.27 (s, 1H), 7.92 – 7.84 (m, 2H), 7.64 – 7.53 (m, 4H), 7.49 (t, *J* = 7.4 Hz, 1H), 7.28 – 7.22 (m, 1H), 4.14 (s, 3H), 3.98 (s, 3H); **^{13}C{^{1}H} NMR** (100 MHz, $CDCl_3$) δ 168.3, 166.3, 160.5, 158.1, 156.4, 153.1,

134.6, 129.2, 129.0, 128.9, 128.2, 127.3, 123.7, 123.0 (d, $J_{C\text{-}F}$ = 10.6 Hz), 122.5 (d, $J_{C\text{-}F}$ = 3.6 Hz), 116.4 (d, $J_{C\text{-}F}$ = 25.9 Hz), 113.0 (d, $J_{C\text{-}F}$ = 9.3 Hz), 108.5 (d, $J_{C\text{-}F}$ = 25.9 Hz), 53.3, 52.9; **^{19}F NMR** (375 MHz, $CDCl_3$) δ -118.4; **HRMS** (ESI-TOF) *m/z*: $[M+H]^+$ calcd for $C_{22}H_{16}FO_5$ 379.0976; found 379.0986.

Dimethyl 8-iodo-4-phenyldibenzo[*b,d*]furan-1,2-dicarboxylate (product 4q)

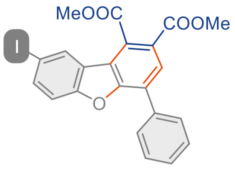

This compound was purified by column chromatography (ethyl acetate/petroleum ether = 1:10, R_f = 0.6) to afford a white solid in 64% yield (62 mg); mp 200-201 °C; **^{1}H NMR** (400 MHz, $CDCl_3$) δ 8.26 (s, 1H), 8.19 (d, J = 1.8 Hz, 1H), 7.87 (d, J = 7.0 Hz, 2H), 7.80 (dd, J = 8.6, 1.8 Hz, 1H), 7.56 (t, J = 7.4 Hz, 2H), 7.48 (t, J = 7.4 Hz, 1H), 7.39 (d, J = 8.6 Hz, 1H), 4.14 (s, 3H), 3.98 (s, 3H); **^{13}C{^{1}H} NMR** (100 MHz, $CDCl_3$) δ 168.3, 166.2, 156.3, 155.4, 137.4, 134.5, 131.2, 129.3, 129.0, 128.9, 128.2, 127.2, 124.7, 123.9, 121.5, 114.1, 87.1, 53.3, 52.9; **HRMS** (ESI-TOF) *m/z*: $[M+H]^+$ calcd for $C_{22}H_{16}IO_5$ 487.0037; found 487.0046.

Dimethyl 8-bromo-4-phenyldibenzo[*b,d*]furan-1,2-dicarboxylate (product 4r)

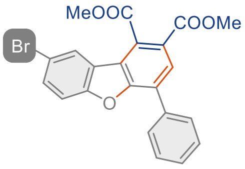

This compound was purified by column chromatography (ethyl acetate/petroleum ether = 1:10, R_f = 0.6) to afford a white solid in 69% yield (60 mg); mp 185-186 °C; **^{1}H NMR** (400 MHz, $CDCl_3$) δ 8.27 (s, 1H), 8.02 (d, J = 2.0 Hz, 1H), 7.88 (dd, J = 7.4, 1.8 Hz, 2H), 7.63 (dd, J = 8.8, 2.0 Hz, 1H), 7.56 (t, J = 7.5 Hz, 2H), 7.52 – 7.43 (m, 2H), 4.14 (s, 3H), 3.98 (s, 3H); **^{13}C{^{1}H} NMR** (100 MHz, $CDCl_3$) δ 168.3, 166.2, 155.8, 155.7, 134.6, 131.7, 129.4, 129.0 (2C), 128.2, 127.3, 125.2, 124.1, 124.0, 121.8, 116.8, 113.7 53.4, 52.9; **HRMS** (ESI-TOF) *m/z*: $[M+H]^+$ calcd for $C_{22}H_{16}BrO_5$ 439.0176; found 439.0173.

Dimethyl 6,8-dibromo-4-phenyldibenzo[*b,d*]furan-1,2-dicarboxylate (product 4s)

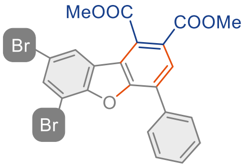

This compound was purified by column chromatography (ethyl acetate/petroleum ether = 1:10, R_f = 0.6) to afford a white solid in 61% yield (63 mg); mp 206-207 °C; **^{1}H NMR** (400 MHz, $CDCl_3$) δ 8.33 (s, 1H), 7.99 – 7.91 (m, 3H), 7.83 (d, J = 1.8 Hz, 1H), 7.62 – 7.55 (m, 2H), 7.53 – 7.46 (m, 1H), 4.13 (s, 3H), 3.99 (s, 3H); **^{13}C{^{1}H} NMR** (100 MHz, $CDCl_3$) δ 168.0, 166.1, 155.4, 153.3, 134.1, 133.9, 129.7, 129.4, 129.1, 129.0, 128.4, 127.6, 124.9, 124.7, 124.3, 121.9, 116.9, 105.7, 53.4, 53.0; **HRMS** (ESI-TOF) *m/z*: $[M+H]^+$ calcd for $C_{22}H_{15}Br_2O_5$ 516.9281; found 516.9284.

Dimethyl 4-(4-methoxyphenyl)-8-methyldibenzo[*b,d*]furan-1,2-dicarboxylate (product 4t)

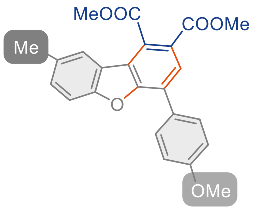

This compound was purified by column chromatography (ethyl acetate/petroleum ether = 1:6, R_f = 0.6) to afford a white solid in 82% yield (66 mg); mp 176-177 °C; **^{1}H NMR** (400 MHz, $CDCl_3$) δ 8.21 (s, 1H), 7.86 (d, J = 8.4 Hz, 2H), 7.65 (s, 1H), 7.49 (d, J = 8.4 Hz, 1H), 7.32 (d, J = 8.5 Hz, 1H), 7.08 (d, J = 8.3 Hz, 2H), 4.14 (s, 3H), 3.97 (s, 3H), 3.89 (s, 3H), 2.50 (s, 3H); **^{13}C{^{1}H} NMR** (100 MHz, $CDCl_3$) δ 168.9, 166.5, 160.0, 155.6, 155.3, 133.3, 130.2, 129.8, 128.0, 127.5, 127.3, 126.5, 123.0, 122.7, 122.1, 122.0, 114.4, 111.6, 55.5, 53.1, 52.8, 21.6; **HRMS** (ESI-TOF) *m/z*: $[M+H]^+$ calcd for $C_{24}H_{21}O_6$ 405.1333; found 405.1336.

Dimethyl 4-(4-fluorophenyl)-8-methyldibenzo[*b,d*]furan-1,2 dicarboxylate (product 4u)

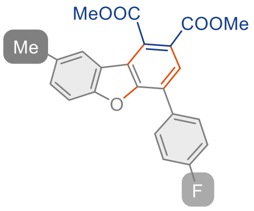

This compound was purified by column chromatography (ethyl acetate/petroleum ether = 1:10, R_f = 0.6) to afford a white solid in 76% yield (59 mg); mp 187-188 °C; **^{1}H NMR** (400 MHz, $CDCl_3$) δ 8.20 (s, 1H), 7.88 (dd, J = 8.6, 5.4 Hz, 2H), 7.65 (s, 1H), 7.49 (d, J = 8.4 Hz, 1H), 7.34 (d, J = 8.4 Hz, 1H), 7.27 – 7.19 (m, 2H), 4.14 (s, 3H), 3.98 (s, 3H), 2.51 (s, 3H); **^{13}C{^{1}H} NMR** (100 MHz, $CDCl_3$) δ 168.7, 166.3, 164.3, 161.8, 155.6, 155.4, 133.5, 131.0 (d, $J_{C\text{-}F}$ = 3.3 Hz), 130.8 (d, $J_{C\text{-}F}$ = 8.2 Hz), 130.0, 128.3, 128.2, 125.8, 122.9 (d, $J_{C\text{-}F}$ = 23.3 Hz), 122.0, 122.0, 116.0 (d, $J_{C\text{-}F}$ = 21.4 Hz), 111.7, 53.2, 52.8, 21.7; **^{19}F NMR** (375 MHz, $CDCl_3$) δ -112.9; **HRMS** (ESI-TOF) *m/z*: $[M+H]^+$ calcd for $C_{23}H_{18}FO_5$ 393.1133; found 393.1134.

Dimethyl 4-(4-chlorophenyl)-8-methoxydibenzo[*b,d*]furan-1,2-dicarboxylate (product 4v)

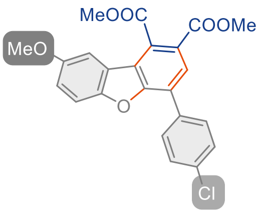

This compound was purified by column chromatography (ethyl acetate/petroleum ether = 1:6, R_f = 0.6) to afford a white solid in 82% yield (69 mg); mp 155-156 °C; **^{1}H NMR** (400 MHz, $CDCl_3$) δ 8.20 (s, 1H), 7.87 – 7.79 (m, 2H), 7.56 – 7.47 (m, 3H), 7.33 (d, J = 2.6 Hz, 1H), 7.11 (dd, J = 9.0, 2.7 Hz, 1H), 4.13 (s, 3H), 3.97 (s, 3H), 3.90 (s, 3H); **^{13}C{^{1}H} NMR** (100 MHz, $CDCl_3$) δ 168.5, 166.2, 156.4, 156.0, 151.7, 134.8, 133.3, 130.2, 129.2, 128.4, 128.3, 125.6, 123.1, 123.0, 122.5, 117.0, 112.6, 105.1, 56.0, 53.1, 52.9; **HRMS** (ESI-TOF) *m/z*: $[M+H]^+$ calcd for $C_{23}H_{18}ClO_6$ 425.0786; found 425.0791.

Dimethyl 8-chloro-4-(*p*-tolyl)dibenzo[*b,d*]furan-1,2-dicarboxylate (product 4w)

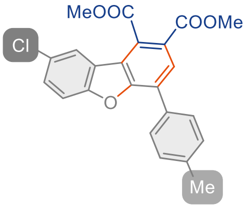

This compound was purified by column chromatography (ethyl acetate/petroleum ether = 1:10, R_f = 0.6) to afford a white solid in 74% yield (60 mg); mp 201-202 °C; **^{1}H NMR** (400 MHz, $CDCl_3$) δ 8.25 (s, 1H), 7.87 (d, J = 2.1 Hz, 1H), 7.78 (d, J = 8.2 Hz, 2H), 7.54 (d, J = 8.8 Hz, 1H), 7.48 (dd, J = 8.8, 2.1 Hz, 1H), 7.37 (d, J = 7.9 Hz, 2H), 4.14 (s, 3H), 3.98 (s, 3H), 2.46 (s, 3H); **^{13}C{^{1}H} NMR** (100 MHz, $CDCl_3$) δ 168.4, 166.3, 155.9, 155.3, 139.0, 131.6, 129.7, 129.3, 129.1, 128.89, 127.9, 127.4, 123.9, 123.6, 122.2, 121.9, 113.2, 53.3, 52.9, 21.5; **HRMS** (ESI-TOF) *m/z*: $[M+H]^+$ calcd for $C_{23}H_{18}ClO_5$ 409.0837; found 409.0843.

Dimethyl 8-chloro-4-(4-methoxyphenyl)dibenzo[*b,d*]furan-1,2-dicarboxylate (product 4x)

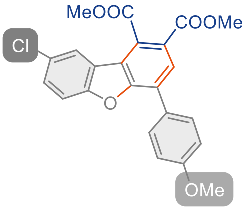

This compound was purified by column chromatography (ethyl acetate/petroleum ether = 1:10, R_f = 0.6) to afford a white solid in 78% yield (66 mg); mp 168-169 °C; **^{1}H NMR** (400 MHz, $CDCl_3$) δ 8.22 (s, 1H), 7.89 – 7.80 (m, 3H), 7.54 (d, J = 8.8 Hz, 1H), 7.48 (dd, J = 8.8, 2.1 Hz, 1H), 7.08 (d, J = 8.8 Hz, 2H), 4.13 (s, 3H), 3.98 (s, 3H), 3.90 (s, 3H); **^{13}C{^{1}H} NMR** (100 MHz, $CDCl_3$) δ 168.4, 166.4,

160.2, 155.8, 155.2, 130.2, 129.3, 128.8 (2C), 127.5, 127.0, 126.9, 124.0, 123.6, 122.2, 121.9, 114.5, 113.2, 55.5, 53.3, 52.9; **HRMS** (ESI-TOF) *m/z*: $[M+H]^+$ calcd for $C_{23}H_{18}ClO_6$ 425.0786; found 425.0781.

Dimethyl 4-(4-bromophenyl)-8-chlorodibenzo[*b,d*]furan-1,2-dicarboxylate (product 4y)

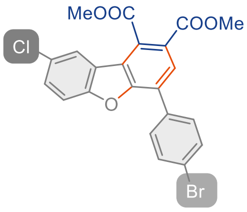

This compound was purified by column chromatography (ethyl acetate/petroleum ether = 1:10, R_f = 0.6) to afford a white solid in 72% yield (68 mg); mp 199-200 °C; ^{1}**H NMR** (500 MHz, $CDCl_3$) δ 8.22 (s, 1H), 7.85 (d, J = 2.1 Hz, 1H), 7.78 – 7.72 (m, 2H), 7.72 – 7.65 (m, 2H), 7.54 (d, J = 8.8 Hz, 1H), 7.49 (dd, J = 8.8, 2.1 Hz, 1H), 4.14 (s, 3H), 3.98 (s, 3H); ^{13}**C{^{1}H} NMR** (100 MHz, $CDCl_3$) δ 168.1, 166.1, 155.7, 155.2, 133.4, 132.2, 130.5, 129.5, 129.1, 129.0, 128.5, 126.0, 124.0, 123.4, 123.3, 122.2, 122.1, 113.2, 53.4, 53.0; **HRMS** (ESI-TOF) *m/z*: $[M+H]^+$ calcd for $C_{22}H_{15}ClBrO_5$ 472.9786; found 472.9779.

Dimethyl 8-bromo-4-(*p*-tolyl)dibenzo[*b,d*]furan-1,2-dicarboxylate (product 4z)

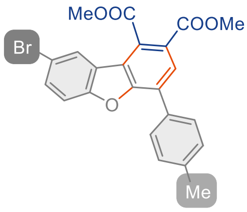

This compound was purified by column chromatography (ethyl acetate/petroleum ether = 1:10, R_f = 0.6) to afford a white solid in 82% yield (74 mg); mp 202-203 °C; ^{1}**H NMR** (500 MHz, $CDCl_3$) δ 8.25 (s, 1H), 8.01 (d, J = 2.0 Hz, 1H), 7.78 (d, J = 8.2 Hz, 2H), 7.63 (dd, J = 8.8, 2.0 Hz, 1H), 7.50 (d, J = 8.7 Hz, 1H), 7.37 (d, J = 7.6 Hz, 2H), 4.14 (s, 3H), 3.98 (s, 3H), 2.46 (s, 3H); ^{13}**C{^{1}H} NMR** (100 MHz, $CDCl_3$) δ 168.4, 166.3, 155.8, 155.7, 139.0, 131.6, 129.8, 129.1, 128.9, 127.9, 127.4, 125.2, 124.2, 124.0, 121.8, 116.7, 113.7, 53.3, 52.9, 21.5; **HRMS** (ESI-TOF) *m/z*: $[M+H]^+$ calcd for $C_{23}H_{18}BrO_5$ 453.0332; found 453.0329.

Dimethyl 8-bromo-4-(4-ethylphenyl)dibenzo[*b,d*]furan-1,2-dicarboxylate (product 4A)

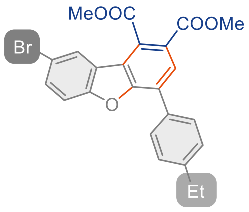

This compound was purified by column chromatography (ethyl acetate/petroleum ether = 1:10, R_f = 0.6) to afford a white solid in 77% yield (72 mg); mp 152-153 °C; **^{1}H NMR** (500 MHz, $CDCl_3$) δ 8.26 (s, 1H), 8.01 (d, *J* = 2.0 Hz, 1H), 7.83 – 7.78 (m, 2H), 7.63 (dd, *J* = 8.8, 2.0 Hz, 1H), 7.50 (d, *J* = 8.7 Hz, 1H), 7.40 (d, *J* = 8.1 Hz, 2H), 4.14 (s, 3H), 3.98 (s, 3H), 2.76 (q, *J* = 7.6 Hz, 2H), 1.32 (t, *J* = 7.6 Hz, 3H); **^{13}C{^{1}H} NMR** (100 MHz, $CDCl_3$) δ 168.4, 166.3, 155.8, 155.7, 145.3, 131.9, 131.6, 129.2, 129.0, 128.6, 127.9, 127.4, 125.2, 124.2, 124.0, 121.7, 116.7, 113.7, 53.3, 52.9, 28.8, 15.7; **HRMS** (ESI-TOF) *m/z*: $[M+H]^+$ calcd for $C_{24}H_{20}BrO_5$ 467.0489; found 467.0489.

Dimethyl 8-bromo-4-(4-methoxyphenyl)dibenzo[*b,d*]furan-1,2-dicarboxylate (product 4B)

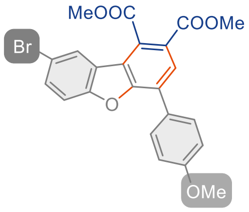

This compound was purified by column chromatography (ethyl acetate/petroleum ether = 1:10, R_f = 0.6) to afford a white solid in 71% yield (66 mg); mp 182-183 °C; **^{1}H NMR** (500 MHz, $CDCl_3$) δ 8.21 (d, *J* = 2.1 Hz, 1H), 8.01 (t, *J* = 2.1 Hz, 1H), 7.83 (dd, *J* = 8.8, 2.1 Hz, 2H), 7.64 – 7.58 (m, 1H), 7.48 (dd, *J* = 8.8, 2.0 Hz, 1H), 7.08 (dd, *J* = 8.8, 2.1 Hz, 2H), 4.13 (s, 3H), 3.97 (s, 3H), 3.90 (s, 3H); **^{13}C{^{1}H} NMR** (100 MHz, $CDCl_3$) δ 168.3, 166.4, 160.2, 155.7, 155.6, 131.6, 130.2, 128.8 127.5, 127.0, 126.8, 125.2, 124.2, 124.0, 121.7, 116.7, 114.5, 113.6, 55.5, 53.3, 52.9; **HRMS** (ESI-TOF) *m/z*: $[M+H]^+$ calcd for $C_{23}H_{18}BrO_6$ 469.0281; found 469.0284.

Dimethyl 6-chloro-4-phenyldibenzo[*b,d*]furan-1,2-dicarboxylate (product 4C)

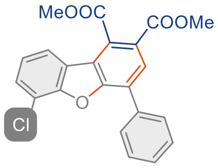

This compound was purified by column chromatography (ethyl acetate/petroleum ether = 1:10, R_f = 0.6) to afford a white solid in 63% yield (50 mg); mp 136-137 °C; **^{1}H NMR** (500 MHz, $CDCl_3$) δ 8.33 (s, 1H), 8.00 – 7.94 (m, 2H), 7.80 (dd, *J* = 7.9, 1.1 Hz, 1H), 7.62 – 7.53 (m, 3H), 7.53 – 7.46 (m, 1H), 7.33 (t, *J* = 7.9 Hz, 1H), 4.13 (s, 3H), 3.99 (s, 3H); **^{13}C{^{1}H} NMR** (101 MHz, $CDCl_3$) δ 168.4, 166.2,

155.3, 152.9, 134.4, 129.3, 129.1, 129.0, 128.8, 128.4, 127.3, 124.7, 124.0, 123.9, 122.8, 120.7, 117.7, 53.3, 52.9; **HRMS** (ESI-TOF) *m/z*: $[M+H]^+$ calcd for $C_{22}H_{16}ClO_5$ 395.0681; found 395.0680.

Dimethyl 7-chloro-4-phenyldibenzo[*b,d*]furan-1,2-dicarboxylate (product 4D)

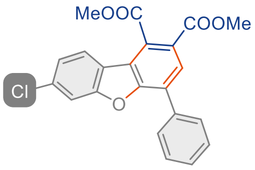

This compound was purified by column chromatography (ethyl acetate/petroleum ether = 1:10, R_f = 0.6) to afford a white solid in 73% yield (57 mg); mp 195-196 °C; ^{1}**H NMR** (400 MHz, $CDCl_3$) δ 8.26 (s, 1H), 7.91 – 7.85 (m, 2H), 7.83 (d, *J* = 8.4 Hz, 1H), 7.63 (d, *J* = 1.8 Hz, 1H), 7.60 – 7.53 (m, 2H), 7.51 – 7.45 (m, 1H), 7.36 (dd, *J* = 8.4, 1.8 Hz, 1H), 4.12 (s, 3H), 3.98 (s, 3H); ^{13}**C{^{1}H} NMR** (100 MHz, $CDCl_3$) δ 168.5, 166.3, 157.1, 155.7, 134.6, 134.5, 129.0 (2C), 128.9, 127.9, 127.2, 124.5, 124.0, 123.0, 122.2, 120.9, 112.8, 53.3, 52.9; **HRMS** (ESI-TOF) *m/z*: $[M+H]^+$ calcd for $C_{22}H_{16}ClO_5$ 395.0681; found 395.0683.

Dimethyl 7-chloro-4-(*p*-tolyl)dibenzo[*b,d*]furan-1,2-dicarboxylate (product 4E)

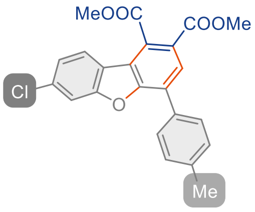

This compound was purified by column chromatography (ethyl acetate/petroleum ether = 1:10, R_f = 0.6) to afford a white solid in 67% yield (55 mg); mp 157-158 °C; ^{1}**H NMR** (400 MHz, $CDCl_3$) δ 8.24 (s, 1H), 7.83 (d, *J* = 8.4 Hz, 1H), 7.77 (d, *J* = 8.1 Hz, 2H), 7.64 (d, *J* = 1.8 Hz, 1H), 7.41 – 7.33 (m, 3H), 4.11 (s, 3H), 3.97 (s, 3H), 2.46 (s, 3H); ^{13}**C{^{1}H} NMR** (100 MHz, $CDCl_3$) δ 168.5, 166.4, 157.1, 155.7, 139.0, 134.4, 131.7, 129.8, 128.9, 128.8, 127.6, 127.3, 124.5, 124.0, 123.0, 122.2, 121.0, 112.8, 53.3, 52.9, 21.5; **HRMS** (ESI-TOF) *m/z*: $[M+H]^+$ calcd for $C_{23}H_{18}ClO_5$ 409.0837; found 409.0835.

Dimethyl 4-(thiophen-2-yl)dibenzo[*b,d*]furan-1,2-dicarboxylate (product 4F)

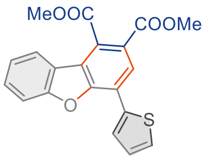

This compound was purified by column chromatography (ethyl acetate/petroleum ether = 1:10, R_f = 0.6) to afford a white solid in 79% yield (58 mg); mp 159-160 °C; ^{1}**H NMR** (400 MHz, $CDCl_3$) δ 8.37 (s, 1H), 7.98 (d, *J* = 3.6 Hz, 1H), 7.90 (d, *J* = 7.9 Hz, 1H), 7.69 (d, *J* = 8.1 Hz, 1H), 7.56 (t, *J* = 7.9 Hz,

1H), 7.49 (d, J = 5.1 Hz, 1H), 7.40 (t, J = 7.6 Hz, 1H), 7.26 – 7.20 (m, 1H), 4.12 (s, 3H), 3.99 (s, 3H); **$^{13}C\{^1H\}$ NMR** (100 MHz, $CDCl_3$) δ 168.6, 166.2, 157.0, 154.0, 136.7, 128.9, 128.3, 127.9, 127.5, 126.9, 126.6, 124.0, 123.5, 123.0, 122.4, 122.0, 120.5, 112.2, 53.2, 52.9; **HRMS** (ESI-TOF) *m/z*: $[M+H]^+$ calcd for $C_{20}H_{15}O_5S$ 367.0635; found 367.0626.

Dimethyl 4-(4-(((5-isopropyl-2-methylcyclohexyl)oxy)carbonyl) phenyl)dibenzo[*b,d*]furan-1,2-dicarboxylate (product 4G)

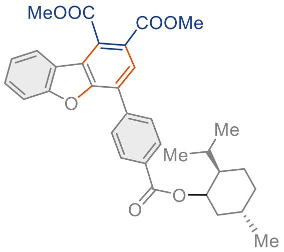

This compound was purified by column chromatography (ethyl acetate/petroleum ether = 1:10, R_f = 0.6) to afford a white solid in 42% yield (45 mg); mp 162-163 °C; **^1H NMR** (400 MHz, $CDCl_3$) δ 8.30 (s, 1H), 8.27 – 8.20 (m, 2H), 8.02 – 7.96 (m, 2H), 7.90 (d, J = 7.7 Hz, 1H), 7.67 – 7.60 (m, 1H), 7.56 (ddd, J = 8.4, 7.2, 1.3 Hz, 1H), 7.45 – 7.36 (m, 1H), 5.02 – 4.96 (m, 1H), 4.15 (s, 3H), 3.99 (s, 3H), 2.18 (d, J = 11.9 Hz, 1H), 2.03 – 1.97 (m, 1H), 1.80 – 1.72 (m, 2H), 1.64 – 1.61 (m, 2H), 1.22 – 1.09 (m, 2H), 0.96 (d, J = 2.5 Hz, 3H), 0.94 (d, J = 3.0 Hz, 3H), 0.83 (d, J = 6.9 Hz, 3H); **$^{13}C\{^1H\}$ NMR** (100 MHz, $CDCl_3$) δ 168.6, 166.1, 165.9, 157.0, 155.5, 139.2, 130.9, 130.2, 129.0, 128.9, 128.8, 125.8, 124.0, 123.3, 123.0, 122.3, 122.0, 112.2, 75.2, 53.3, 52.9, 47.5, 41.1, 34.5, 31.6, 26.7, 23.8, 22.2, 20.9, 16.7; **HRMS** (ESI-TOF) *m/z*: $[M+H]^+$ calcd for $C_{33}H_{35}O_7$ 543.2377; found 543.2368.

Dimethyl 4-(4-((2-(naphthalen-1-yl)ethoxy) carbonyl)phenyl)dibenzo [*b,d*] furan-1,2-dicarboxylate (product 4H)

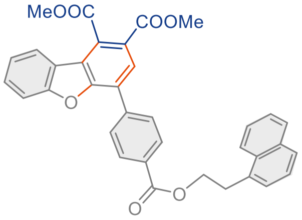

This compound was purified by column chromatography (ethyl acetate/petroleum ether = 1:10, R_f = 0.6) to afford a white solid in 46% yield (51 mg); mp 115-116 °C; **^1H NMR** (400 MHz, $CDCl_3$) δ 8.30 (d, J = 1.8 Hz, 1H), 8.25 – 8.17 (m, 3H), 7.98 (dd, J = 8.4, 1.8 Hz, 2H), 7.90 (d, J = 7.3 Hz, 2H), 7.80 (d, J = 7.0 Hz, 1H), 7.63 (t, J = 8.1 Hz, 1H), 7.61 – 7.51 (m, 3H), 7.49 – 7.38 (m, 3H), 4.73 (t, J = 7.4 Hz, 2H), 4.15 (s, 3H), 4.00 (s, 3H), 3.61 (t, J = 7.4 Hz, 2H); **$^{13}C\{^1H\}$ NMR** (100 MHz, $CDCl_3$) δ 168.5, 166.4, 166.1, 157.0, 155.5, 139.4, 134.0, 133.8, 132.2, 130.3, 130.2, 129.0, 128.9, 128.8, 127.7,

127.2, 126.4, 125.8, 125.7, 124.0, 123.8, 123.3, 123.0, 122.3, 121.9, 112.2, 65.3, 53.3, 52.9, 32.5; **HRMS** (ESI-TOF) *m/z*: $[M+H]^+$ calcd for $C_{35}H_{27}O_7$ 559.1751; found 559.1750.

Dimethyl 4-(4-((((3a*S*,5*S*,6*R*)-5-((*S*)-2,2-dimethyl-1,3-dioxolan-4-yl)-2,2-dimethyltetrahydrofuro[2,3-*d*][1,3]dioxol-6-yl) oxy)carbonyl)phenyl)dibenzo[*b,d*]furan-1,2-dicarboxylate (product 4I)

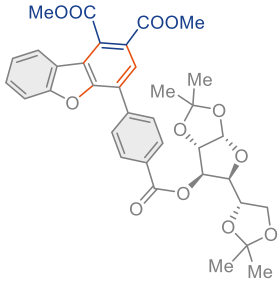

This compound was purified by column chromatography (ethyl acetate/petroleum ether = 1:10, R_f = 0.6) to afford a white solid in 50% yield (63 mg); mp 88-89 °C; **^{1}H NMR** (400 MHz, $CDCl_3$) δ 8.30 (s, 1H), 8.26 – 8.14 (m, 2H), 8.06 – 7.96 (m, 2H), 7.90 (d, *J* = 7.6 Hz, 1H), 7.68 – 7.61 (m, 1H), 7.61 – 7.52 (m, 1H), 7.46 – 7.37 (m, 1H), 5.99 (d, *J* = 3.7 Hz, 1H), 5.56 (d, *J* = 2.9 Hz, 1H), 4.68 (d, *J* = 3.7 Hz, 1H), 4.47 – 4.30 (m, 2H), 4.20 – 4.05 (m, 5H), 3.99 (s, 3H), 1.58 (s, 3H), 1.44 (s, 3H), 1.35 (s, 3H), 1.30 (s, 3H); **^{13}C{^{1}H} NMR** (100 MHz, $CDCl_3$) δ 168.5, 166.1, 165.0, 157.0, 155.5, 140.0, 130.4, 129.2, 129.1, 128.8, 125.5, 124.1, 123.4, 122.3, 122.2, 121.9, 112.6, 109.6, 105.3, 83.5, 80.1, 72.8, 67.4, 53.4, 53.3, 52.9, 27.0, 27.0, 26.9, 26.4, 25.4; **HRMS** (ESI-TOF) *m/z*: $[M+H]^+$ calcd for $C_{35}H_{35}O_{12}$ 647.2123; found 647.2120.

Diethyl 4-phenyldibenzo[*b,d*]furan-1,2-dicarboxylate (product 4J)

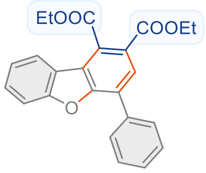

This compound was purified by column chromatography (ethyl acetate/petroleum ether = 1:10, R_f = 0.6) to afford a white solid in 79% yield (61 mg); mp 134-135 °C; **^{1}H NMR** (400 MHz, $CDCl_3$) δ 8.28 (s, 1H), 8.01 – 7.86 (m, 3H), 7.65 – 7.46 (m, 5H), 7.39 (t, *J* = 7.6 Hz, 1H), 4.63 (q, *J* = 7.2 Hz, 2H), 4.45 (q, *J* = 7.2 Hz, 2H), 1.53 – 1.39 (m, 6H); **^{13}C{^{1}H} NMR** (100 MHz, $CDCl_3$) δ 168.3, 165.9, 157.0, 155.4, 135.0, 129.1, 129.0, 128.8, 128.7, 128.6, 128.5, 126.8, 123.8, 123.6, 122.7, 122.3, 122.2, 112.2, 62.3, 61.8, 14.4, 14.2; **HRMS** (ESI-TOF) *m/z*: $[M+H]^+$ calcd for $C_{24}H_{21}O_5$ 389.1384; found 389.1379.

Dimethyl 3-methyl-4-phenyldibenzo[*b,d*]furan-1,2-dicarboxylate (product 4K)

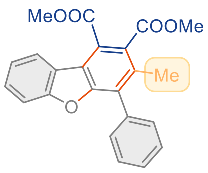

This compound was purified by column chromatography (ethyl acetate/petroleum ether = 1:10, R_f= 0.6) to afford a white solid in 74% yield (55 mg); mp 114-115 °C; **^{1}H NMR** (500 MHz, $CDCl_3$) δ 8.24 (d, J = 8.2 Hz, 1H), 7.59 – 7.52 (m, 2H), 7.52 – 7.45 (m, 3H), 7.43 – 7.39 (m, 2H), 7.37 – 7.32 (m, 1H), 4.08 (s, 3H), 3.97 (s, 3H), 2.34 (s, 3H); **^{13}C{^{1}H} NMR** (100 MHz, $CDCl_3$) δ 169.6, 167.3, 157.2, 154.9, 134.5, 133.6, 130.1, 130.0, 129.9, 128.7, 128.4, 128.4, 124.1, 123.9, 123.3, 122.7, 120.8, 111.9, 52.9, 52.8, 17.9; **HRMS** (ESI-TOF) *m/z*: $[M+H]^+$ calcd for $C_{23}H_{19}O_5$ 375.1227; found 375.1223.

Dimethyl 3-ethyl-4-phenyldibenzo[*b,d*]furan-1,2-dicarboxylate (product 4L)

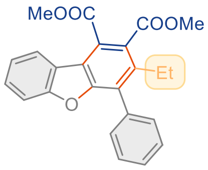

This compound was purified by column chromatography (ethyl acetate/petroleum ether = 1:10, R_f= 0.6) to afford a white solid in 56% yield (43 mg); mp 125-126 °C; **^{1}H NMR** (400 MHz, $CDCl_3$) δ 8.21 (d, J = 8.2 Hz, 1H), 7.59 – 7.50 (m, 3H), 7.46 (d, J = 3.6 Hz, 2H), 7.40 (d, J = 6.2 Hz, 2H), 7.36 – 7.31 (m, 1H), 4.07 (s, 3H), 3.96 (s, 3H), 2.75 (q, J = 7.5 Hz, 2H), 1.02 (t, J = 7.5 Hz, 3H); **^{13}C{^{1}H} NMR** (100 MHz, $CDCl_3$) δ 169.6, 167.4, 157.3, 155.1, 140.1, 134.4, 129.9, 129.6, 129.3, 128.7, 128.4, 128.4, 124.4, 124.1, 123.2, 122.7, 120.7, 112.0, 52.9, 52.7, 24.1, 16.0; **HRMS** (ESI-TOF) *m/z*: $[M+H]^+$ calcd for $C_{24}H_{21}O_5$ 389.1384; found 389.1382.

3-Ethyl 1,2-dimethyl 4-phenyldibenzo[*b,d*]furan-1,2,3-tricarboxylate (product 4M)

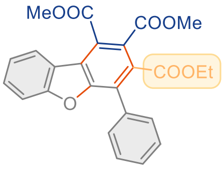

This compound was purified by column chromatography (ethyl acetate/petroleum ether = 1:10, R_f= 0.6) to afford a white solid in 58% yield (50 mg); mp 137-138 °C; **^{1}H NMR** (400 MHz, $CDCl_3$) δ 8.10 (d, J = 8.0 Hz, 1H), 7.57 – 7.48 (m, 7H), 7.43 – 7.36 (m, 1H), 4.14 – 4.03 (m, 5H), 3.92 (s, 3H), 1.01 (t, J = 7.1 Hz, 3H); **^{13}C{^{1}H} NMR** (100 MHz, $CDCl_3$) δ 167.3, 167.2, 167.1, 157.8, 154.7, 133.1, 132.9, 129.7, 129.4, 128.9, 128.5, 127.2, 126.5, 125.5, 123.9, 123.7, 123.3, 121.9, 112.3, 61.9, 53.2, 53.2, 13.7; **HRMS** (ESI-TOF) *m/z*: $[M+H]^+$ calcd for $C_{25}H_{21}O_7$ 433.1282; found 433.1280.

Methyl 1-oxo-4-phenyl-1,3-dihydro-1*H*-benzofuro[2,3-f]isobenzofuran-10-carboxylate (product 4N)

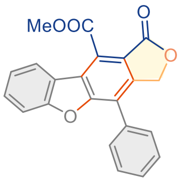

This compound was purified by column chromatography (ethyl acetate/petroleum ether = 1:10, R_f = 0.6) to afford a white solid in 67% yield (48 mg); mp 128-129 °C; **^{1}H NMR** (500 MHz, $CDCl_3$) δ 8.01 (d, *J* = 7.9 Hz, 1H), 7.66 – 7.58 (m, 5H), 7.58 – 7.52 (m, 2H), 7.46 – 7.39 (m, 1H), 5.43 (s, 2H), 4.20 (s, 3H); **^{13}C{^{1}H} NMR** (125 MHz, $CDCl_3$) *δ* 168.9, 166.2, 157.6, 156.8, 144.0, 131.7, 129.5, 129.4, 129.3, 129.1, 124.5, 124.4, 124.1, 122.9, 122.5, 121.8, 118.4, 112.3, 69.0, 53.5; **HRMS** (ESI-TOF) *m/z*: [M+H]$^+$ calcd for $C_{22}H_{15}O_5$ 359.0914; found 359.0919.

Methyl 1-oxo-5-phenyl-3,4-dihydro-1*H*-benzofuro[2,3-*g*]isochromene-11-carboxylate (product 4O)

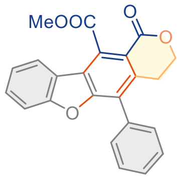

This compound was purified by column chromatography (ethyl acetate/petroleum ether = 1:10, R_f = 0.6) to afford a white solid in 75% yield (56 mg); mp 140-141 °C; **^{1}H NMR** (500 MHz, $CDCl_3$) *δ* 7.89 (d, *J* = 7.8 Hz, 1H), 7.61 – 7.56 (m, 2H), 7.55 – 7.49 (m, 3H), 7.46 (d, *J* = 7.5 Hz, 2H), 7.41 – 7.35 (m, 1H), 4.53 – 4.47 (m, 2H), 4.15 (s, 3H), 3.11 – 3.04 (m, 2H); **^{13}C{^{1}H} NMR** (125 MHz, $CDCl_3$) *δ* 168.4, 164.1, 157.0, 156.4, 137.6, 132.8, 130.1, 129.0, 128.9 (2C), 125.4, 124.1, 124.0, 122.30, 121.9, 121.6, 118.3, 112.2, 67.1, 53.5, 27.2; **HRMS** (ESI-TOF) *m/z*: [M+H]$^+$ calcd for $C_{23}H_{17}O_5$ 373.1071; found 373.1075.

(4-Phenyldibenzo[*b,d*]furan-1,2-diyl)dimethanol (product 5)

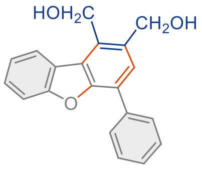

This compound was purified by column chromatography (ethyl acetate/petroleum ether = 1:10, R_f = 0.6) to afford a white solid in 88% yield (54 mg); mp 140-141 °C; **^{1}H NMR** (400 MHz, $CDCl_3$) *δ* 8.22 – 8.13 (m, 1H), 7.88 – 7.80 (m, 2H), 7.58 (d, *J* = 8.2 Hz, 1H), 7.56 – 7.49 (m, 3H), 7.49 – 7.40 (m, 2H), 7.39 – 7.32 (m, 1H), 5.25 (s, 2H), 4.91 (s, 2H), 3.16 (s, 1H), 2.85 (s, 1H); **^{13}C{^{1}H} NMR** (100 MHz, $CDCl_3$) *δ* 156.7, 153.4, 135.9, 134.6, 133.5, 128.9, 128.8, 128.6, 128.1, 127.5, 125.4, 124.7, 123.6, 123.3, 123.1, 112.1, 64.2, 59.3; **HRMS** (ESI-TOF) *m/z*: [M+H]$^+$ calcd for $C_{20}H_{17}O_3$ 305.1172; found 305.1164.

1-(Methoxycarbonyl)-4-phenyldibenzo[*b,d*]furan-2-carboxylic acid (product 6)

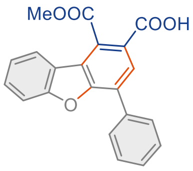

This compound was purified by column chromatography (ethyl acetate/petroleum ether = 1:10, R_f = 0.6) to afford a white solid in 93% yield (64 mg); mp 213-214 °C; **^{1}H NMR** (400 MHz, DMSO) δ 8.19 (s, 1H), 7.96 – 7.89 (m, 2H), 7.84 (d, *J* = 7.7 Hz, 2H), 7.62 (dt, *J* = 15.0, 7.4 Hz, 3H), 7.56 – 7.44 (m, 2H), 4.03 (s, 3H); **^{13}C{^{1}H} NMR** (100 MHz, DMSO) δ 168.3, 166.9, 156.7, 154.8, 134.6, 129.6, 129.5, 129.3, 129.2, 128.8, 128.3, 126.5, 124.9, 124.6, 122.1, 121.6, 112.8, 53.5; **HRMS** (ESI-TOF) *m/z*: [M+H]$^+$ calcd for $C_{21}H_{15}O_5$ 347.0914; found 347.0907.

1,3,3-Trimethyl-5-phenyl-isobenzofuro[5,4-*b*]benzofuran-1-ol (product 7)

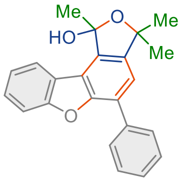

This compound was purified by column chromatography (ethyl acetate/petroleum ether = 1:10, R_f = 0.6) to afford a white solid in 61% yield (42 mg); mp 188-189 °C; **^{1}H NMR** (400 MHz, $CDCl_3$) δ 8.30 (d, *J* = 7.8 Hz, 1H), 7.92 (d, *J* = 1.5 Hz, 2H), 7.63 (d, *J* = 8.3 Hz, 1H), 7.61 – 7.55 (m, 2H), 7.54 – 7.44 (m, 2H), 7.36 (m, 1H), 7.33 (s, 1H), 3.33 (s, 1H), 2.07 (s, 3H), 1.71 (s, 3H), 1.65 (s, 3H); **^{13}C{^{1}H} NMR** (100 MHz, $CDCl_3$) δ 156.9, 153.9, 142.3, 136.4, 133.9, 129.0, 128.8, 128.2, 127.7, 127.3, 124.1, 123.2, 122.3, 119.2, 119.0, 111.9, 106.5, 85.4, 31.4, 29.9, 28.1; **HRMS** (ESI-TOF) *m/z*: [M+H]$^+$ calcd for $C_{23}H_{21}O_3$ 345.1485; found 345.1484.

Dimethyl 4-phenyl-8-(phenylethynyl)dibenzo[*b,d*]furan-1,2-dicarboxylate (product 8)

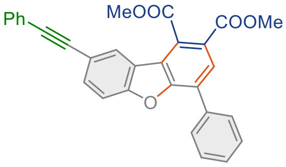

This compound was purified by column chromatography (ethyl acetate/petroleum ether = 1:10, R_f = 0.6) to afford a white solid in 51% yield (47 mg); mp 188-189 °C; **^{1}H NMR** (400 MHz, $CDCl_3$) δ 8.28 (s, 1H), 8.06 (d, *J* = 1.7 Hz, 1H), 7.90 (dd, *J* = 8.4, 1.3 Hz, 2H), 7.70 (dd, *J* = 8.5, 1.7 Hz, 1H), 7.63 – 7.53 (m, 5H), 7.53 – 7.45 (m, 1H), 7.43 – 7.32 (m, 3H), 4.18 (s, 3H), 3.99 (s, 3H); **^{13}C{^{1}H} NMR** (100 MHz, $CDCl_3$) δ 168.5, 166.3, 156.5, 155.8, 134.7, 132.5, 131.8, 129.19, 129.0, 129.0, 128.9, 128.5 (2C), 128.3, 127.2, 125.5, 123.7, 123.2, 122.4, 122.2, 119.1, 112.4, 89.2, 89.1, 53.4, 52.9; **HRMS** (ESI-TOF) *m/z*: [M+H]$^+$ calcd for $C_{30}H_{21}O_5$ 461.1384; found 461.1393.

Dimethyl 4-phenyldibenzo[*b*,*d*]furan-1,2-dicarboxylate-3-*d* (product 3a-*d*)

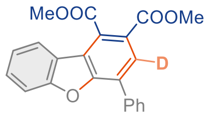

This compound was purified by column chromatography (ethyl acetate/petroleum ether = 1:10, R_f = 0.6) to afford a white solid in 28% yield (20 mg); mp 188-189 °C; **^{1}H NMR** (500 MHz, $CDCl_3$) δ 7.94 – 7.87 (m, 3H), 7.63 (d, J = 8.3 Hz, 1H), 7.61 – 7.52 (m, 3H), 7.51 – 7.46 (m, 1H), 7.41 – 7.38 (m, 1H),4.14 (s, 3H), 3.98 (s, 3H); **^{13}C{^{1}H} NMR** (100 MHz, $CDCl_3$) δ 168.5, 166.3, 157.0, 155.5, 134.9, 134.8, 129.0, 129.0, 128.8, 128.2, 126.9, 123.8, 123.3, 123.2, 122.8, 122.2, 122.1, 112.2, 53.2, 52.8; **HRMS** (ESI-TOF) *m/z*: $[M+H]^+$ calcd for $C_{22}H_{16}DO_5$ 362.1133; found 362.1142.

13. ^{1}H, ^{13}C, and ^{19}F NMR Spectra

Dimethyl 4-phenyldibenzo[*b*,*d*]furan-1,2-dicarboxylate (product 4a)

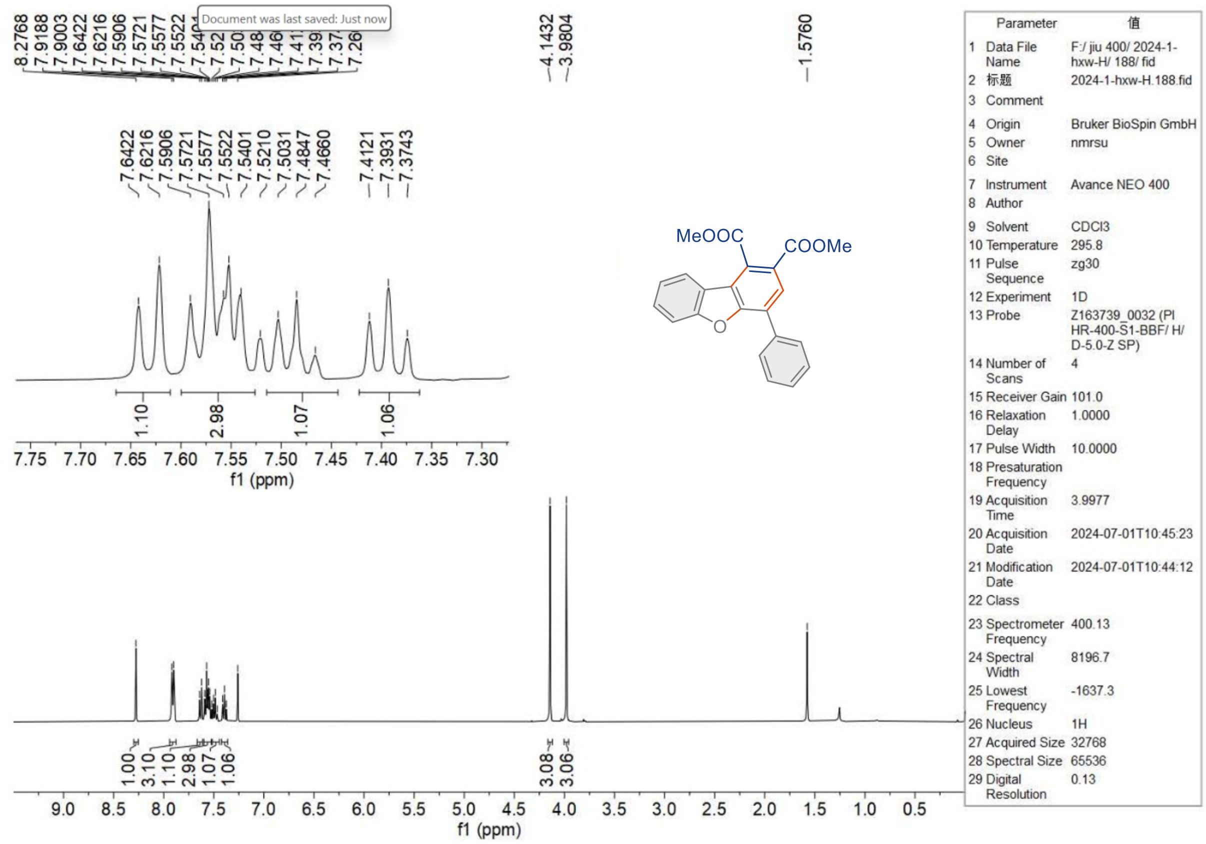

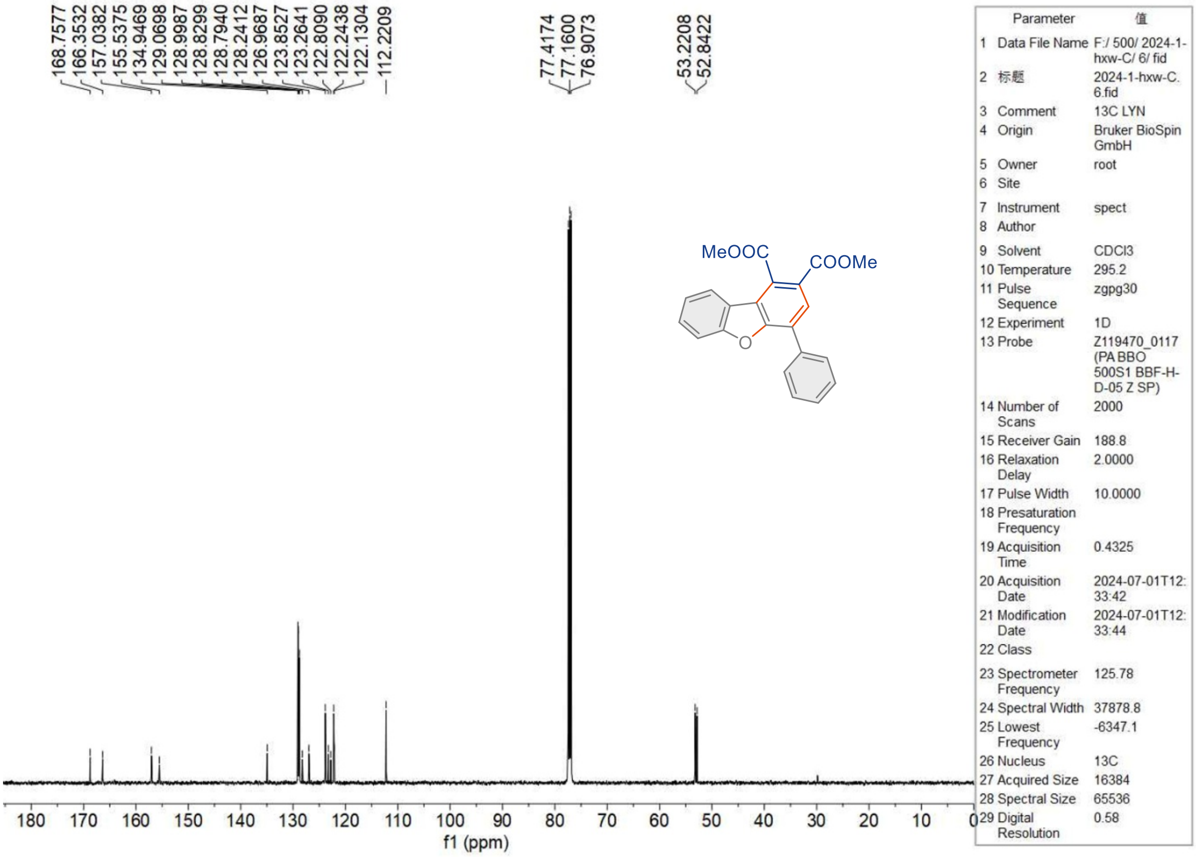

Dimethyl 4-(*p*-tolyl)dibenzo[*b*,*d*]furan-1,2-dicarboxylate (product 4b)

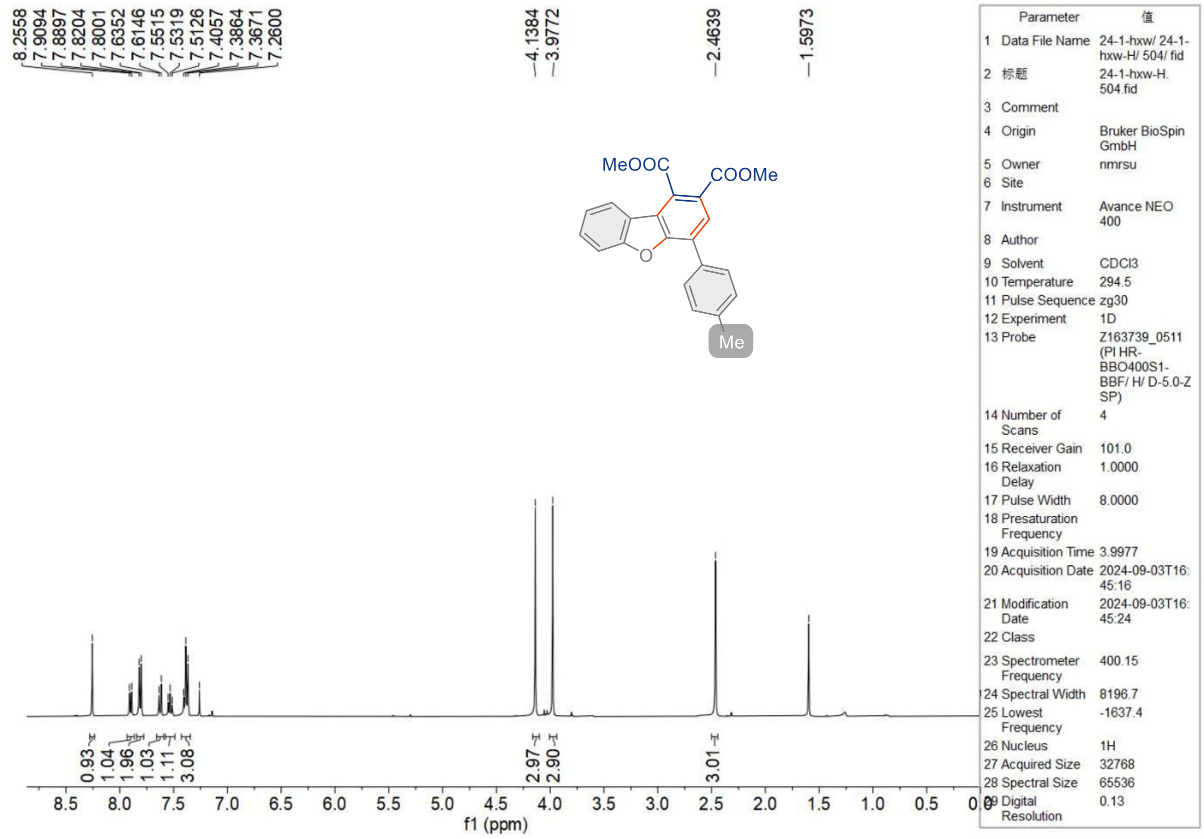

Parameter	值
1 Data File Name	24-1-hxw/ 24-1-hxw-H/ 504/ fid
2 标题	24-1-hxw-H.504.fid
3 Comment	
4 Origin	Bruker BioSpin GmbH
5 Owner	nmrsu
6 Site	
7 Instrument	Avance NEO 400
8 Author	
9 Solvent	CDCl3
10 Temperature	294.5
11 Pulse Sequence	zg30
12 Experiment	1D
13 Probe	Z163739_0511 (PI HR-BBO400S1-BBF/ H/ D-5.0-Z SP)
14 Number of Scans	4
15 Receiver Gain	101.0
16 Relaxation Delay	1.0000
17 Pulse Width	8.0000
18 Presaturation Frequency	
19 Acquisition Time	3.9977
20 Acquisition Date	2024-09-03T16:45:16
21 Modification Date	2024-09-03T16:45:24
22 Class	
23 Spectrometer Frequency	400.15
24 Spectral Width	8196.7
25 Lowest Frequency	-1637.4
26 Nucleus	1H
27 Acquired Size	32768
28 Spectral Size	65536
29 Digital Resolution	0.13

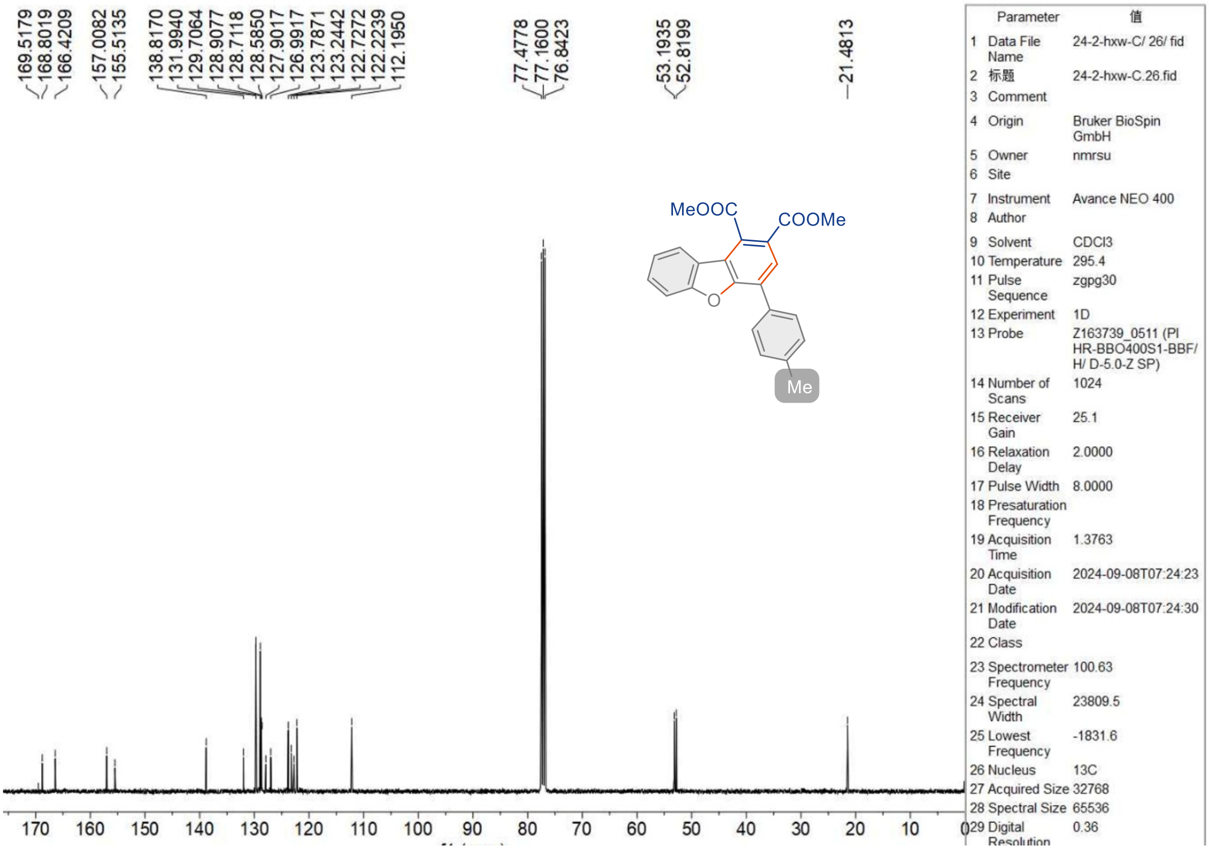

Parameter	值
1 Data File Name	24-2-hxw-C/ 26/ fid
2 标题	24-2-hxw-C.26.fid
3 Comment	
4 Origin	Bruker BioSpin GmbH
5 Owner	nmrsu
6 Site	
7 Instrument	Avance NEO 400
8 Author	
9 Solvent	CDCl3
10 Temperature	295.4
11 Pulse Sequence	zgpg30
12 Experiment	1D
13 Probe	Z163739_0511 (PI HR-BBO400S1-BBF/ H/ D-5.0-Z SP)
14 Number of Scans	1024
15 Receiver Gain	25.1
16 Relaxation Delay	2.0000
17 Pulse Width	8.0000
18 Presaturation Frequency	
19 Acquisition Time	1.3763
20 Acquisition Date	2024-09-08T07:24:23
21 Modification Date	2024-09-08T07:24:30
22 Class	
23 Spectrometer Frequency	100.63
24 Spectral Width	23809.5
25 Lowest Frequency	-1831.6
26 Nucleus	13C
27 Acquired Size	32768
28 Spectral Size	65536
29 Digital Resolution	0.36

Dimethyl 4-(4-butylphenyl)dibenzo[*b,d*]furan-1,2-dicarboxylate (product 4c)

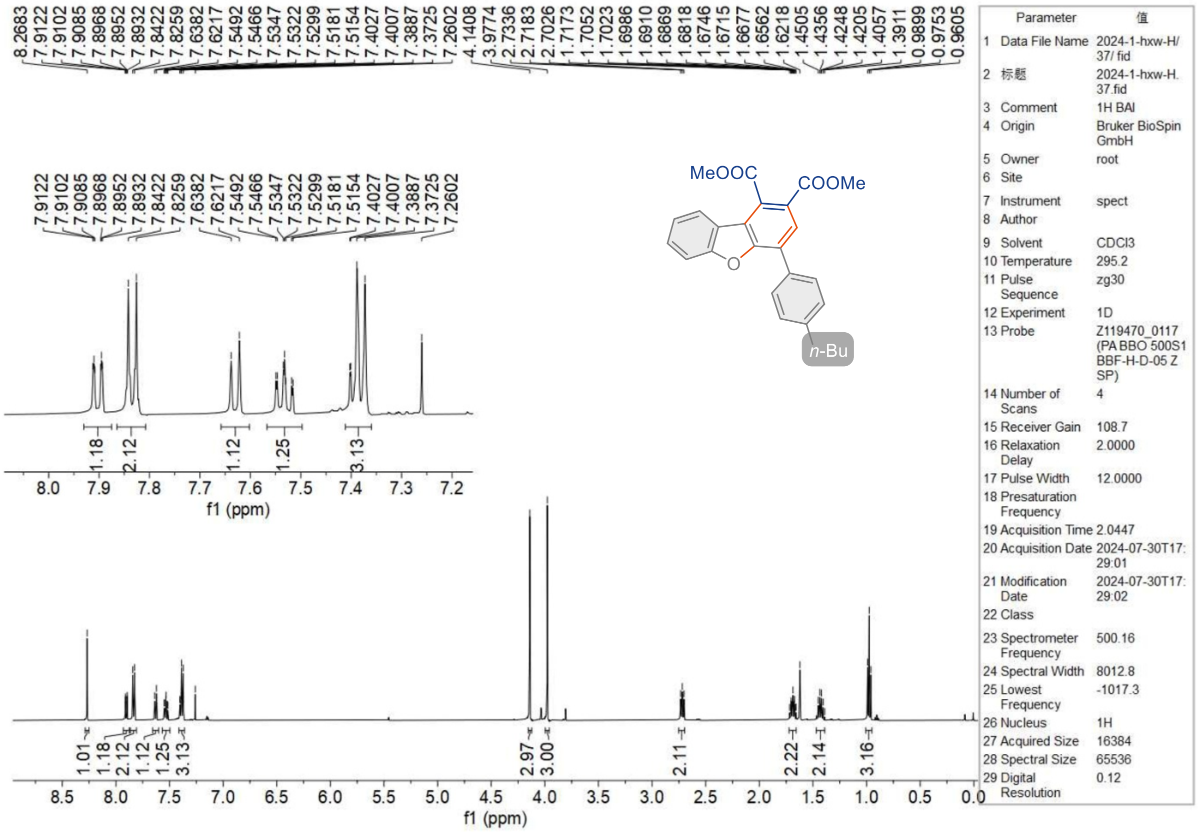

Parameter	值
1 Data File Name	2024-1-hxw-H/37/ fid
2 标题	2024-1-hxw-H.37.fid
3 Comment	1H BAI
4 Origin	Bruker BioSpin GmbH
5 Owner	root
6 Site	
7 Instrument	spect
8 Author	
9 Solvent	CDCl3
10 Temperature	295.2
11 Pulse Sequence	zg30
12 Experiment	1D
13 Probe	Z119470_0117 (PA BBO 500S1 BBF-H-D-05 Z SP)
14 Number of Scans	4
15 Receiver Gain	108.7
16 Relaxation Delay	2.0000
17 Pulse Width	12.0000
18 Presaturation Frequency	
19 Acquisition Time	2.0447
20 Acquisition Date	2024-07-30T17:29:01
21 Modification Date	2024-07-30T17:29:02
22 Class	
23 Spectrometer Frequency	500.16
24 Spectral Width	8012.8
25 Lowest Frequency	-1017.3
26 Nucleus	1H
27 Acquired Size	16384
28 Spectral Size	65536
29 Digital Resolution	0.12

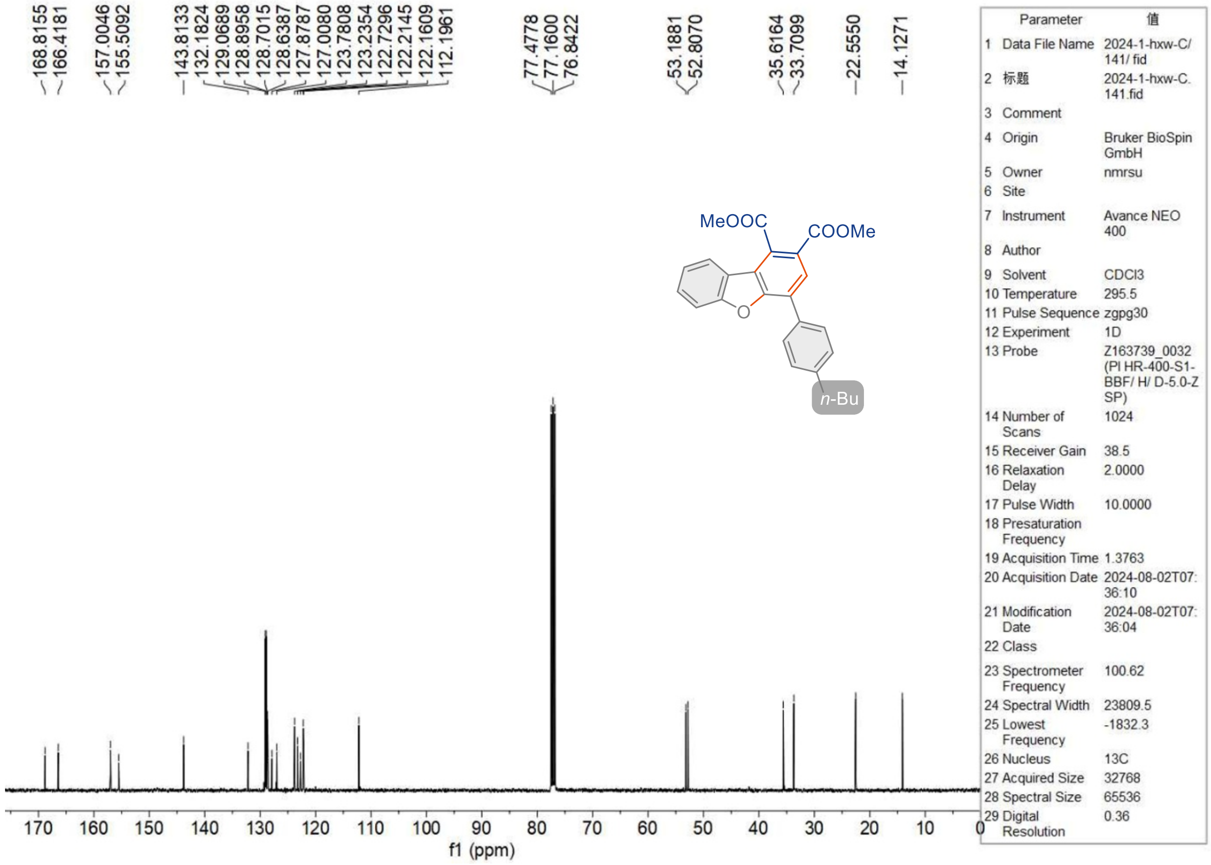

Parameter	值
1 Data File Name	2024-1-hxw-C/141/ fid
2 标题	2024-1-hxw-C.141.fid
3 Comment	
4 Origin	Bruker BioSpin GmbH
5 Owner	nmrsu
6 Site	
7 Instrument	Avance NEO 400
8 Author	
9 Solvent	CDCl3
10 Temperature	295.5
11 Pulse Sequence	zgpg30
12 Experiment	1D
13 Probe	Z163739_0032 (PI HR-400-S1-BBF/ H/ D-5.0-Z SP)
14 Number of Scans	1024
15 Receiver Gain	38.5
16 Relaxation Delay	2.0000
17 Pulse Width	10.0000
18 Presaturation Frequency	
19 Acquisition Time	1.3763
20 Acquisition Date	2024-08-02T07:36:10
21 Modification Date	2024-08-02T07:36:04
22 Class	
23 Spectrometer Frequency	100.62
24 Spectral Width	23809.5
25 Lowest Frequency	-1832.3
26 Nucleus	13C
27 Acquired Size	32768
28 Spectral Size	65536
29 Digital Resolution	0.36

Dimethyl 4-(4-methoxyphenyl)dibenzo[*b,d*]furan-1,2-dicarboxylate (product 4d)

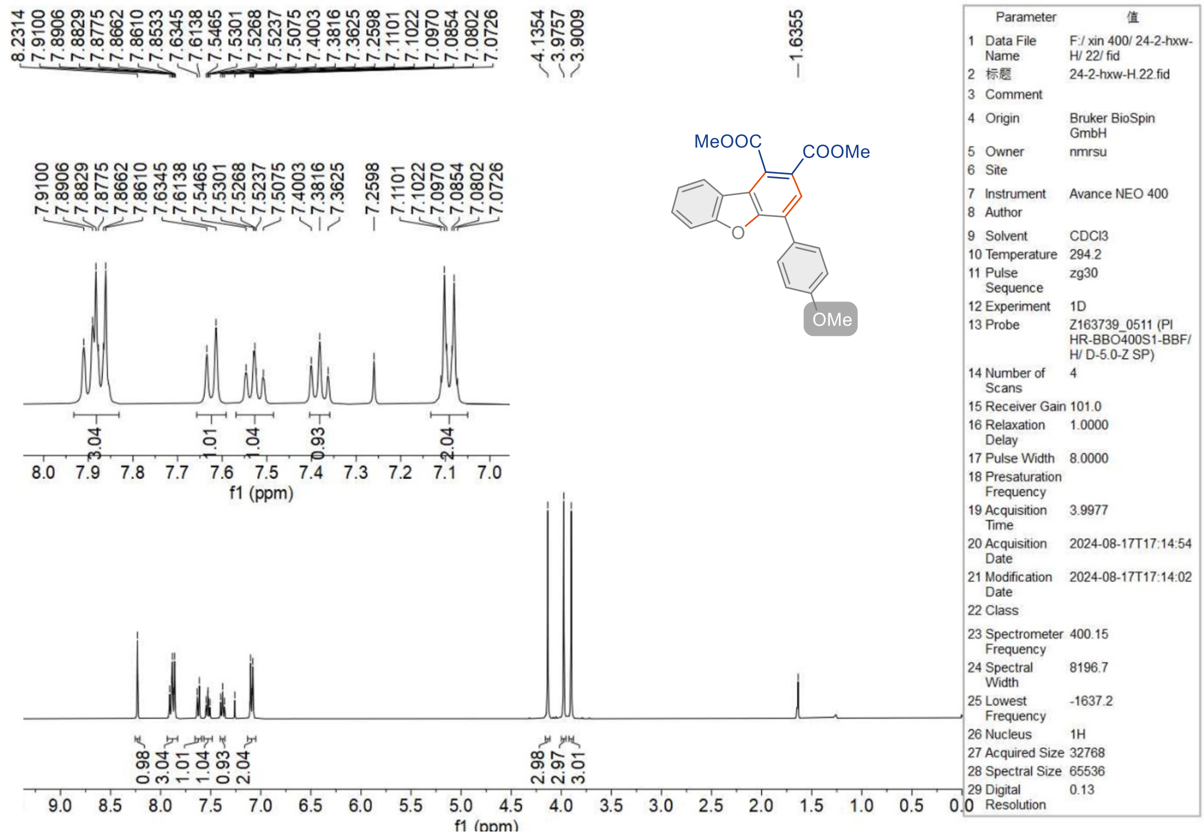

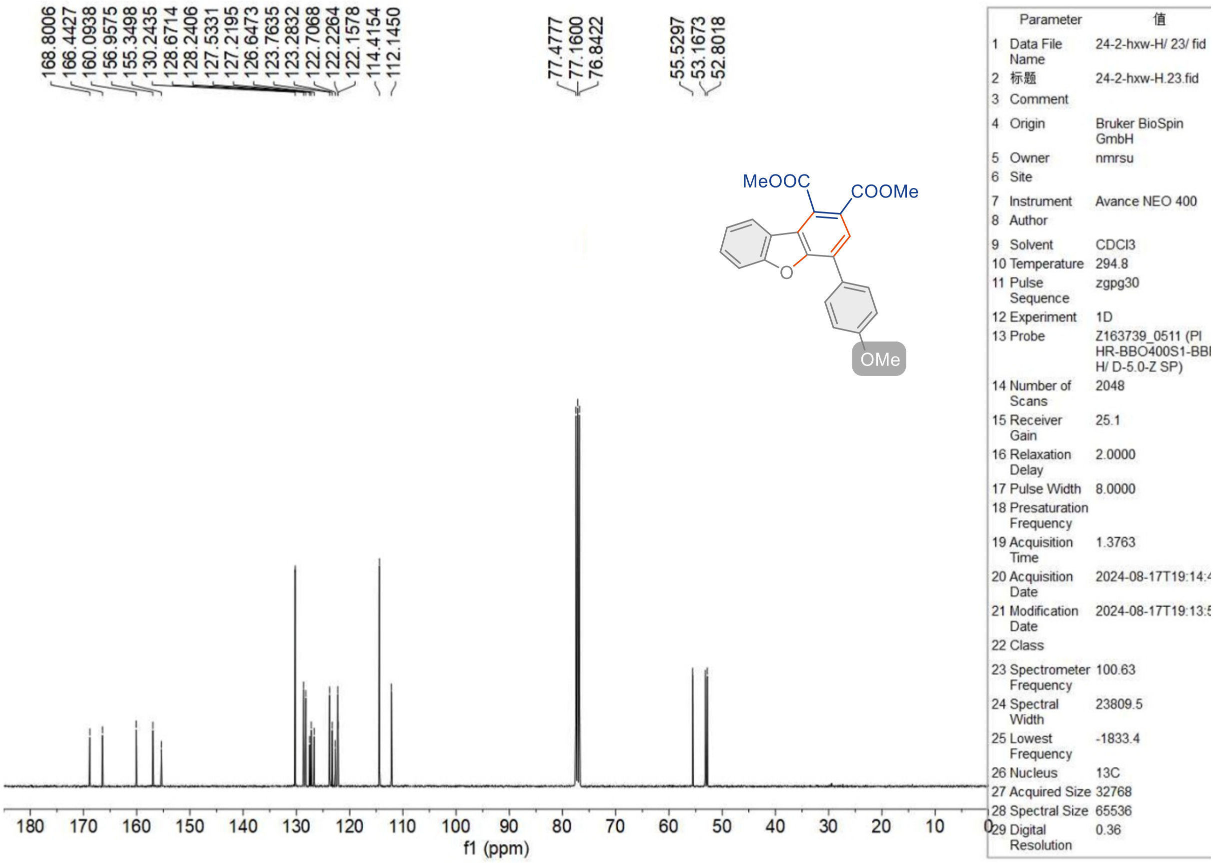

Dimethyl 4-(4-ethoxyphenyl)dibenzo[*b*,*d*]furan-1,2-dicarboxylate (product 4e)

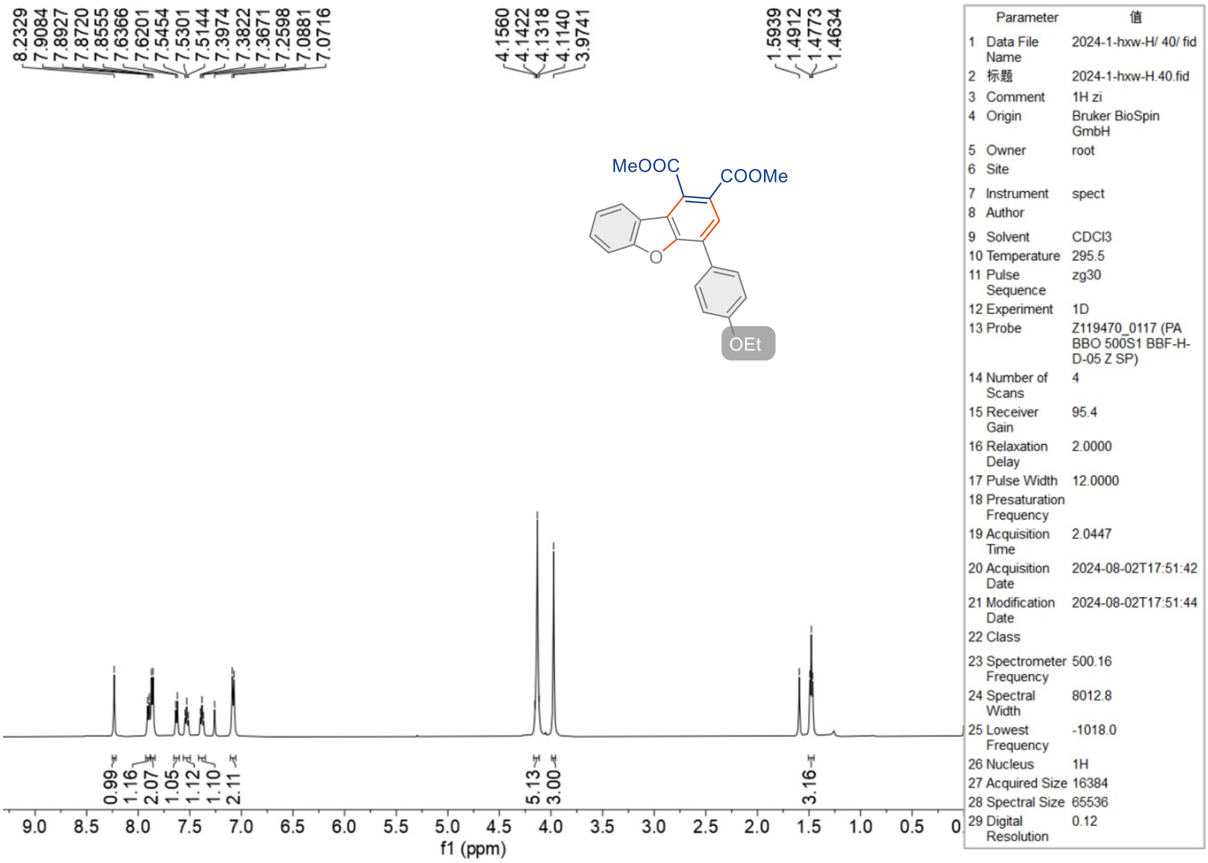

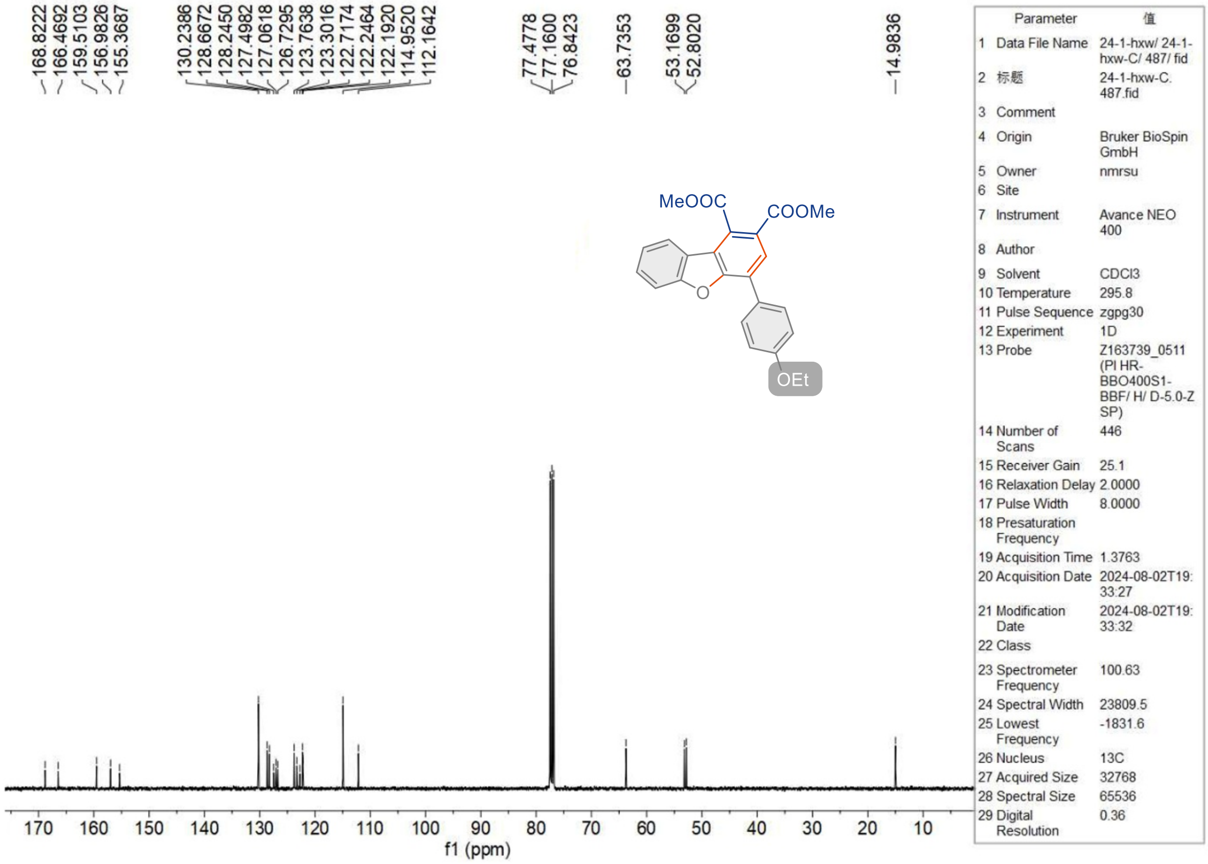

Dimethyl 4-(4-(trifluoromethyl)phenyl)dibenzo[*b*,*d*]furan-1,2-dicarboxylate (product 4f)

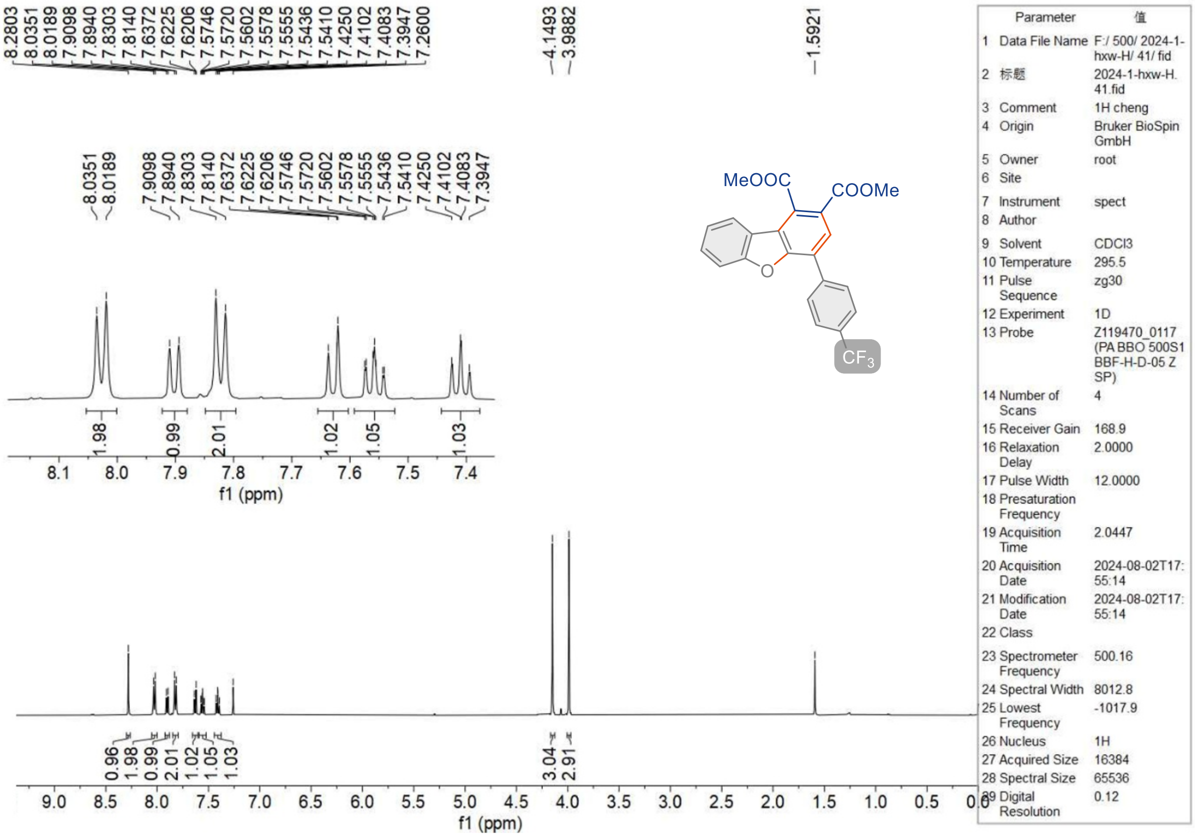

Parameter	值
1 Data File Name	F:/ 500/ 2024-1-hxw-H/ 41/ fid
2 标题	2024-1-hxw-H.41.fid
3 Comment	1H cheng
4 Origin	Bruker BioSpin GmbH
5 Owner	root
6 Site	
7 Instrument	spect
8 Author	
9 Solvent	CDCl3
10 Temperature	295.5
11 Pulse Sequence	zg30
12 Experiment	1D
13 Probe	Z119470_0117 (PA BBO 500S1 BBF-H-D-05 Z SP)
14 Number of Scans	4
15 Receiver Gain	168.9
16 Relaxation Delay	2.0000
17 Pulse Width	12.0000
18 Presaturation Frequency	
19 Acquisition Time	2.0447
20 Acquisition Date	2024-08-02T17:55:14
21 Modification Date	2024-08-02T17:55:14
22 Class	
23 Spectrometer Frequency	500.16
24 Spectral Width	8012.8
25 Lowest Frequency	-1017.9
26 Nucleus	1H
27 Acquired Size	16384
28 Spectral Size	65536
29 Digital Resolution	0.12

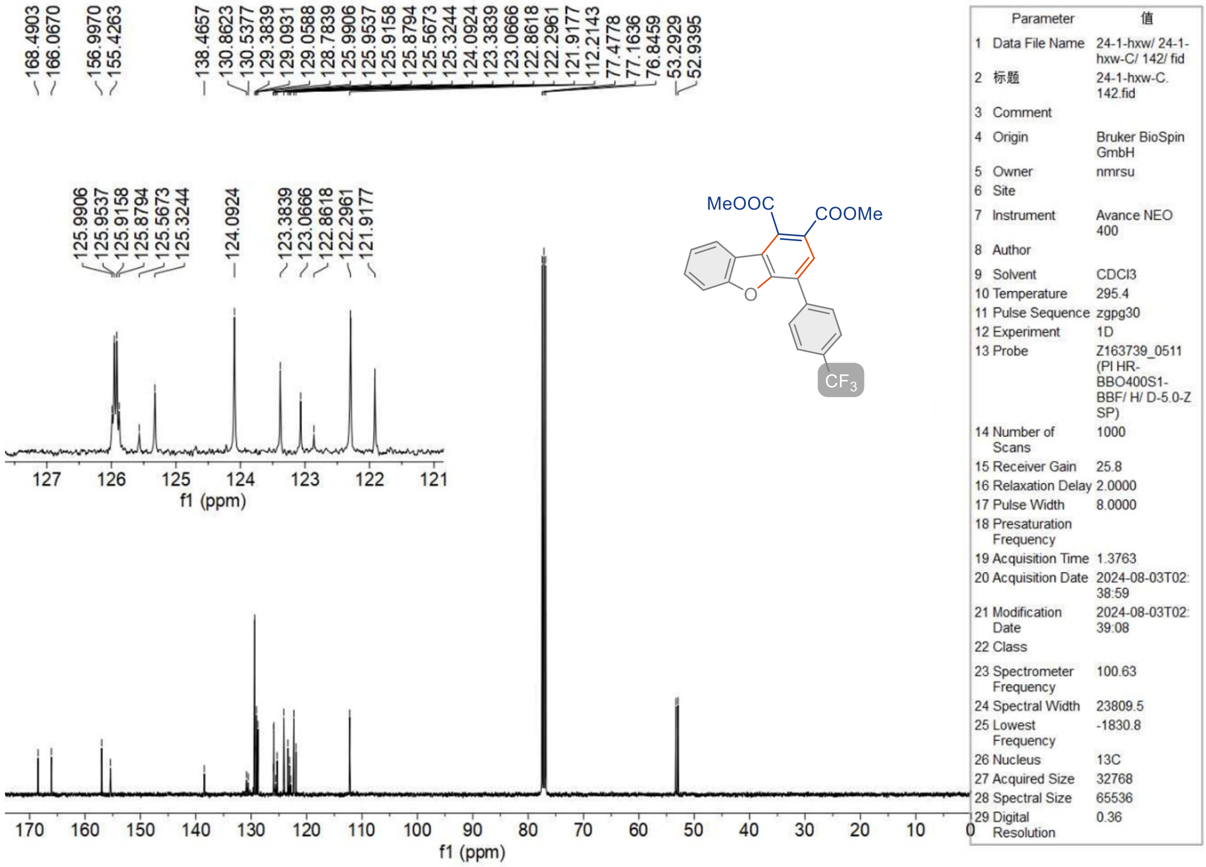

Parameter	值
1 Data File Name	24-1-hxw/ 24-1-hxw-C/ 142/ fid
2 标题	24-1-hxw-C.142.fid
3 Comment	
4 Origin	Bruker BioSpin GmbH
5 Owner	nmrsu
6 Site	
7 Instrument	Avance NEO 400
8 Author	
9 Solvent	CDCl3
10 Temperature	295.4
11 Pulse Sequence	zgpg30
12 Experiment	1D
13 Probe	Z163739_0511 (PI HR-BBO400S1-BBF/ H/ D-5.0-Z SP)
14 Number of Scans	1000
15 Receiver Gain	25.8
16 Relaxation Delay	2.0000
17 Pulse Width	8.0000
18 Presaturation Frequency	
19 Acquisition Time	1.3763
20 Acquisition Date	2024-08-03T02:38:59
21 Modification Date	2024-08-03T02:39:08
22 Class	
23 Spectrometer Frequency	100.63
24 Spectral Width	23809.5
25 Lowest Frequency	-1830.8
26 Nucleus	13C
27 Acquired Size	32768
28 Spectral Size	65536
29 Digital Resolution	0.36

Dimethyl 4-(4-(trifluoromethyl)phenyl)dibenzo[*b*,*d*]furan-1,2-dicarboxylate (product 4f)

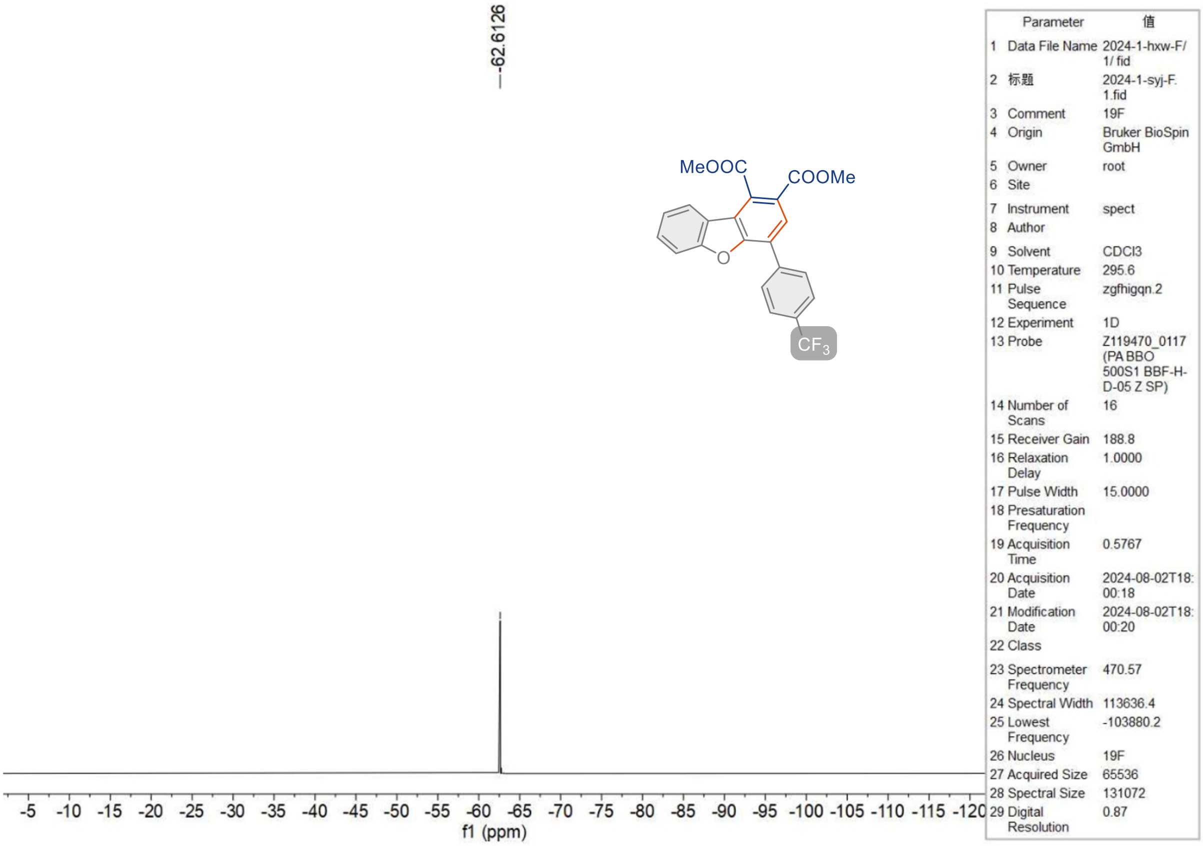

	Parameter	值
1	Data File Name	2024-1-hxw-F/1/ fid
2	标题	2024-1-syj-F.1.fid
3	Comment	19F
4	Origin	Bruker BioSpin GmbH
5	Owner	root
6	Site	
7	Instrument	spect
8	Author	
9	Solvent	CDCl3
10	Temperature	295.6
11	Pulse Sequence	zgfhigqn.2
12	Experiment	1D
13	Probe	Z119470_0117 (PA BBO 500S1 BBF-H-D-05 Z SP)
14	Number of Scans	16
15	Receiver Gain	188.8
16	Relaxation Delay	1.0000
17	Pulse Width	15.0000
18	Presaturation Frequency	
19	Acquisition Time	0.5767
20	Acquisition Date	2024-08-02T18:00:18
21	Modification Date	2024-08-02T18:00:20
22	Class	
23	Spectrometer Frequency	470.57
24	Spectral Width	113636.4
25	Lowest Frequency	-103880.2
26	Nucleus	19F
27	Acquired Size	65536
28	Spectral Size	131072
29	Digital Resolution	0.87

Dimethyl 4-([1,1'-biphenyl]-4-yl)dibenzo[*b*,*d*]furan-1,2-dicarboxylate (product 4g)

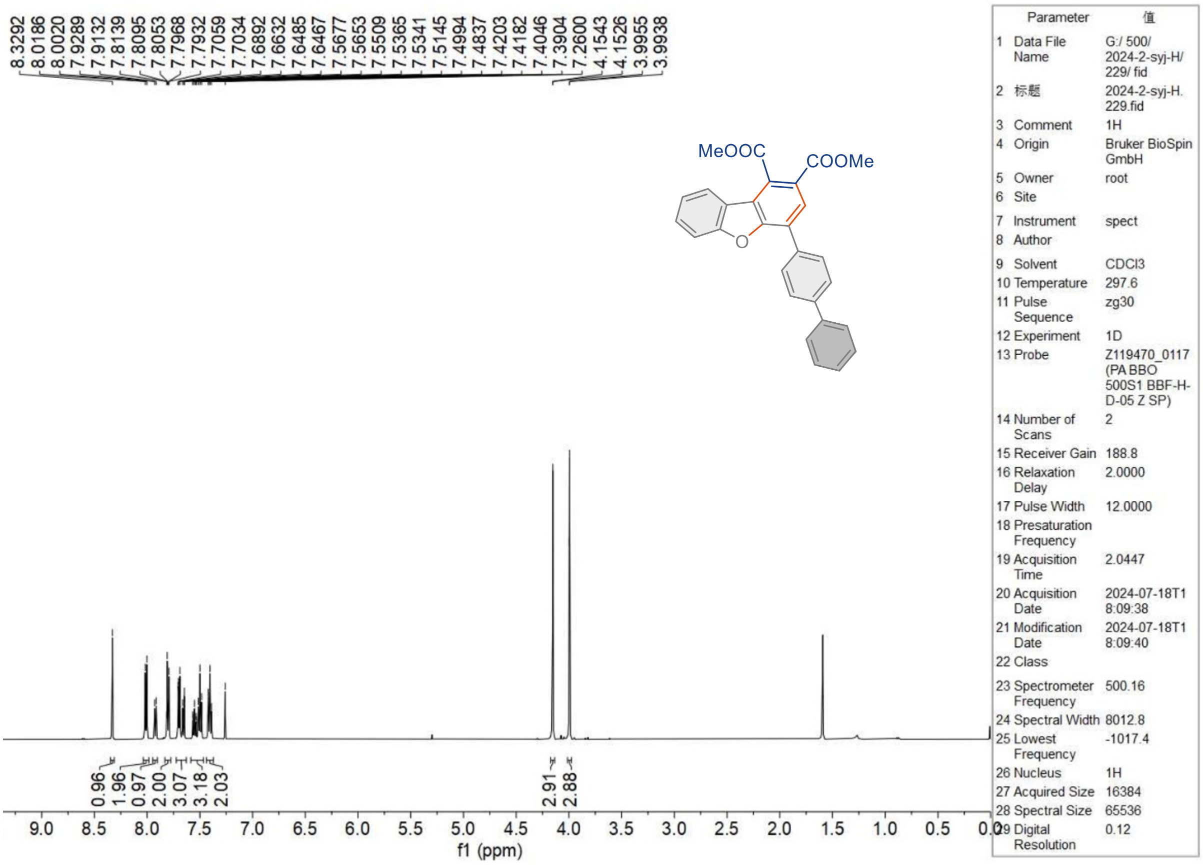

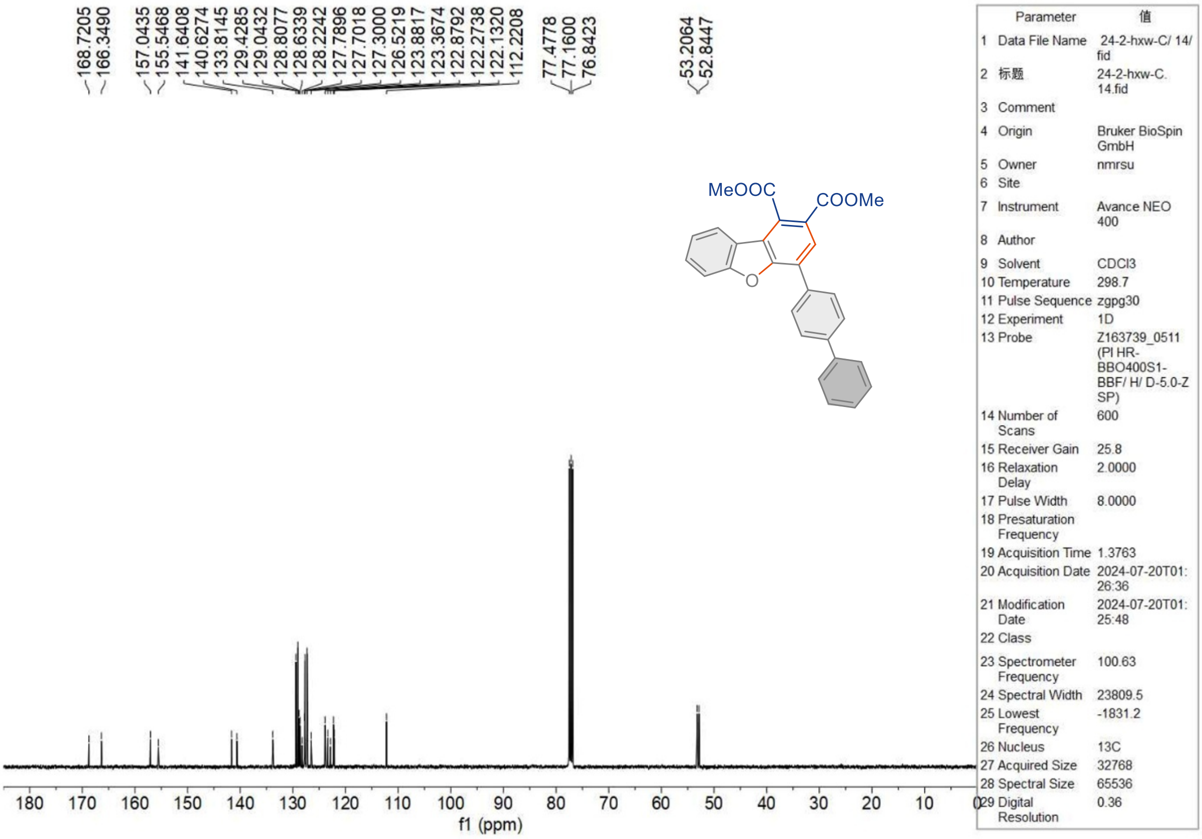

Dimethyl 4-(4'-propyl-[1,1'-biphenyl]-4-yl)dibenzo[*b*,*d*]furan-1,2-dicarboxylate (product 4h)

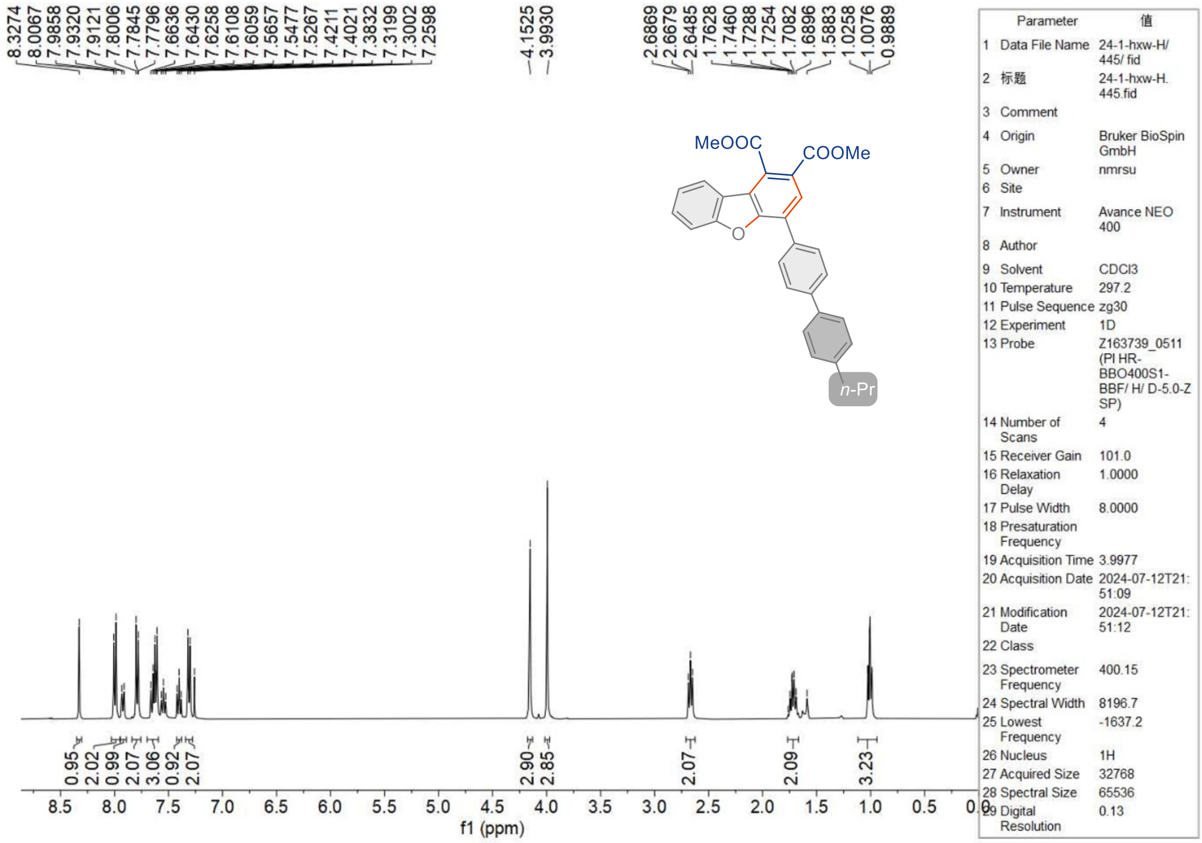

Parameter	值
1 Data File Name	24-1-hxw-H/ 445/ fid
2 标题	24-1-hxw-H. 445.fid
3 Comment	
4 Origin	Bruker BioSpin GmbH
5 Owner	nmrsu
6 Site	
7 Instrument	Avance NEO 400
8 Author	
9 Solvent	CDCl3
10 Temperature	297.2
11 Pulse Sequence	zg30
12 Experiment	1D
13 Probe	Z163739_0511 (PI HR-BBO400S1-BBF/ H/ D-5.0-Z SP)
14 Number of Scans	4
15 Receiver Gain	101.0
16 Relaxation Delay	1.0000
17 Pulse Width	8.0000
18 Presaturation Frequency	
19 Acquisition Time	3.9977
20 Acquisition Date	2024-07-12T21:51:09
21 Modification Date	2024-07-12T21:51:12
22 Class	
23 Spectrometer Frequency	400.15
24 Spectral Width	8196.7
25 Lowest Frequency	-1637.2
26 Nucleus	1H
27 Acquired Size	32768
28 Spectral Size	65536
29 Digital Resolution	0.13

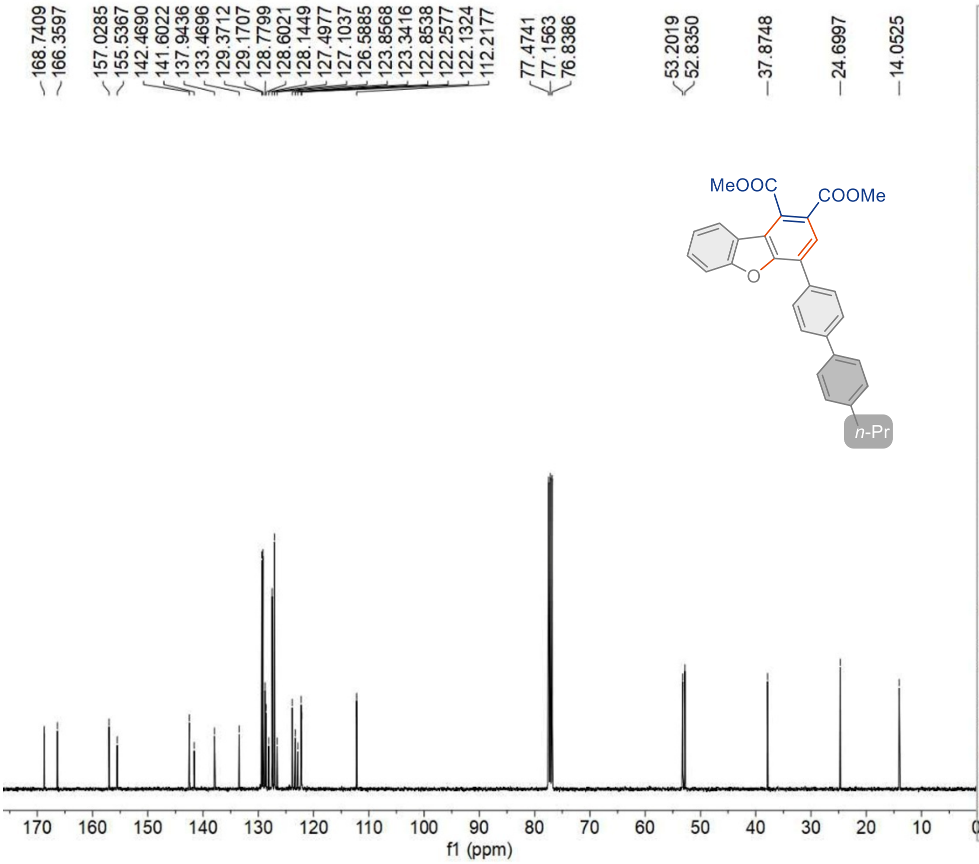

Parameter	值
1 Data File Name	24-1-hxw/ 24-1-hxw-C/ 126/ fid
2 标题	24-1-hxw-C. 126.fid
3 Comment	
4 Origin	Bruker BioSpin GmbH
5 Owner	nmrsu
6 Site	
7 Instrument	Avance NEO 400
8 Author	
9 Solvent	CDCl3
10 Temperature	297.4
11 Pulse Sequence	zgpg30
12 Experiment	1D
13 Probe	Z163739_0511 (PI HR-BBO400S1-BBF/ H/ D-5.0-Z SP)
14 Number of Scans	1024
15 Receiver Gain	25.8
16 Relaxation Delay	2.0000
17 Pulse Width	8.0000
18 Presaturation Frequency	
19 Acquisition Time	1.3763
20 Acquisition Date	2024-07-13T00:43:06
21 Modification Date	2024-07-13T00:43:14
22 Class	
23 Spectrometer Frequency	100.63
24 Spectral Width	23809.5
25 Lowest Frequency	-1831.9
26 Nucleus	13C
27 Acquired Size	32768
28 Spectral Size	65536
29 Digital Resolution	0.36

Dimethyl 4-(4-chlorophenyl)dibenzo[*b*,*d*]furan-1,2-dicarboxylate (product 4i)

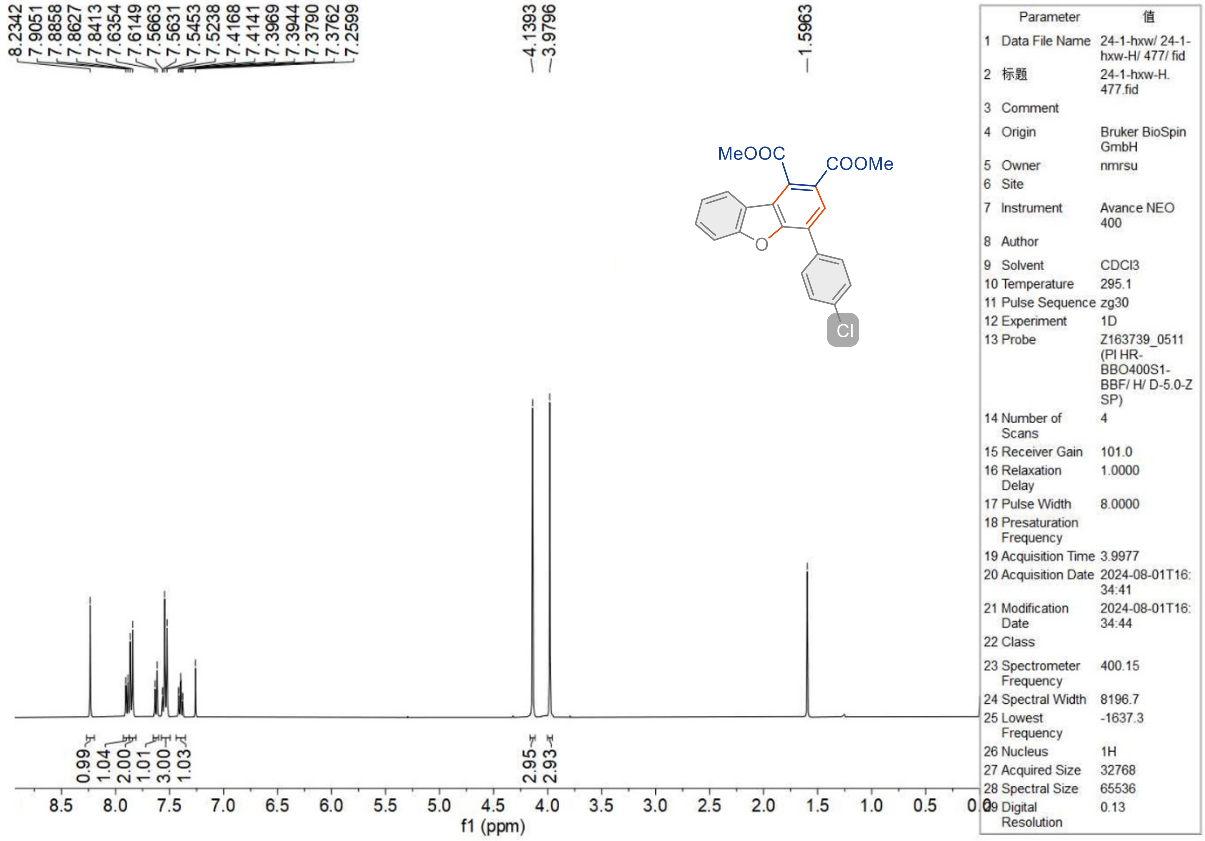

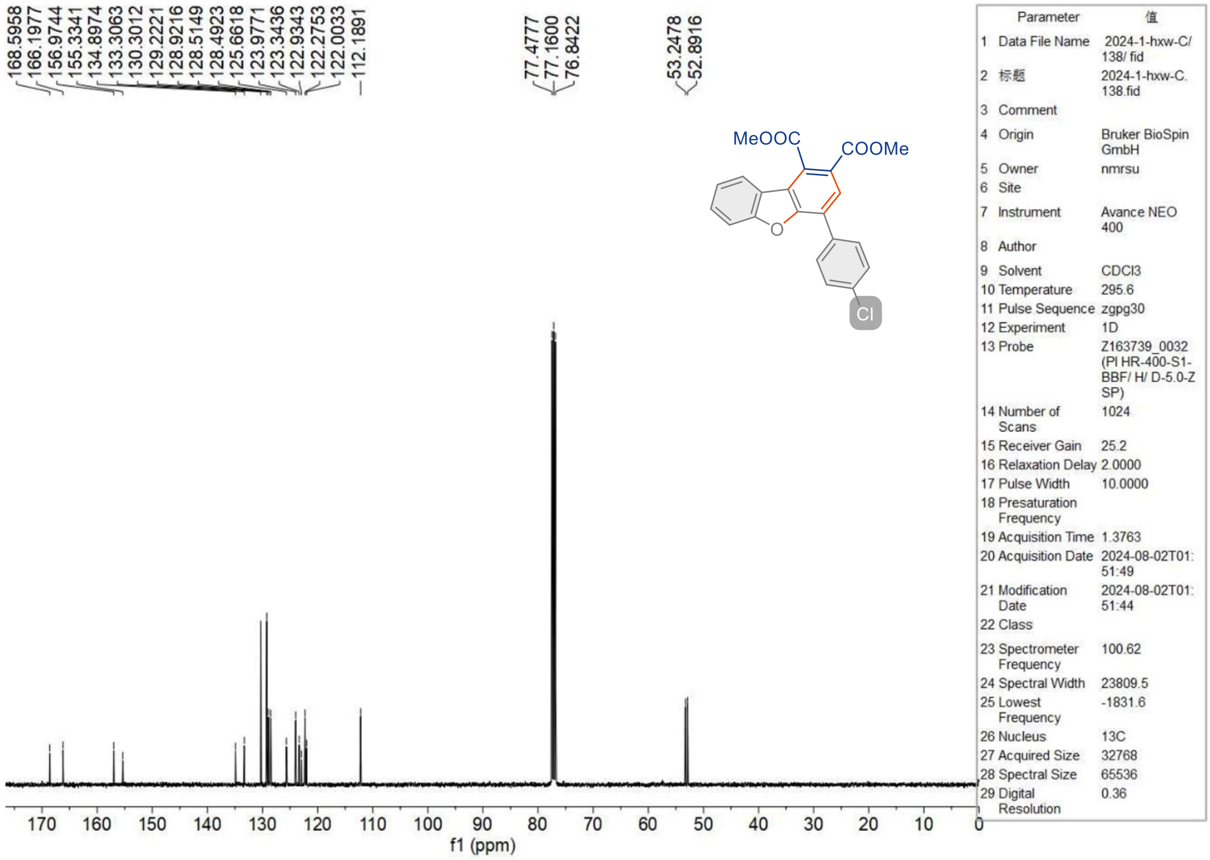

Dimethyl 4-(4-bromophenyl)dibenzo[*b*,*d*]furan-1,2-dicarboxylate (product 4j)

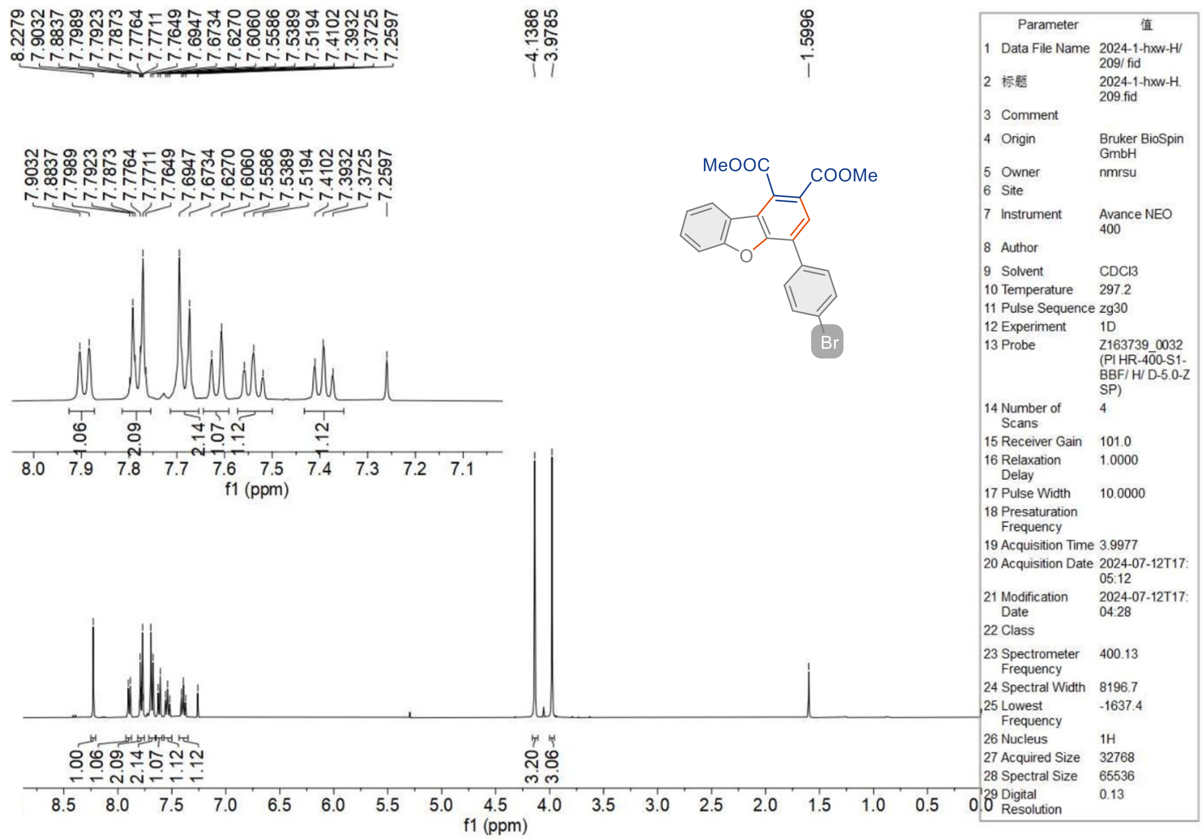

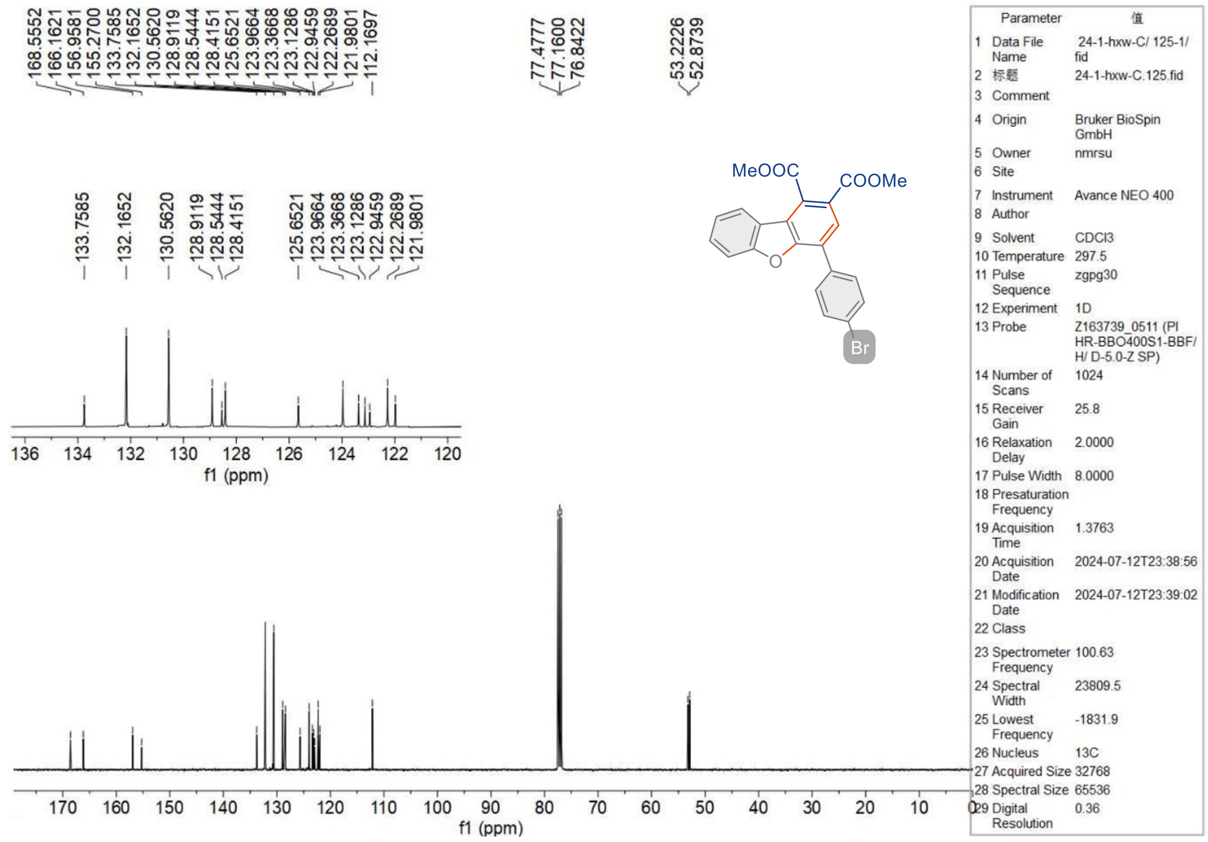

Dimethyl 4-(3-fluorophenyl)dibenzo[*b,d*]furan-1,2-dicarboxylate (product 4k)

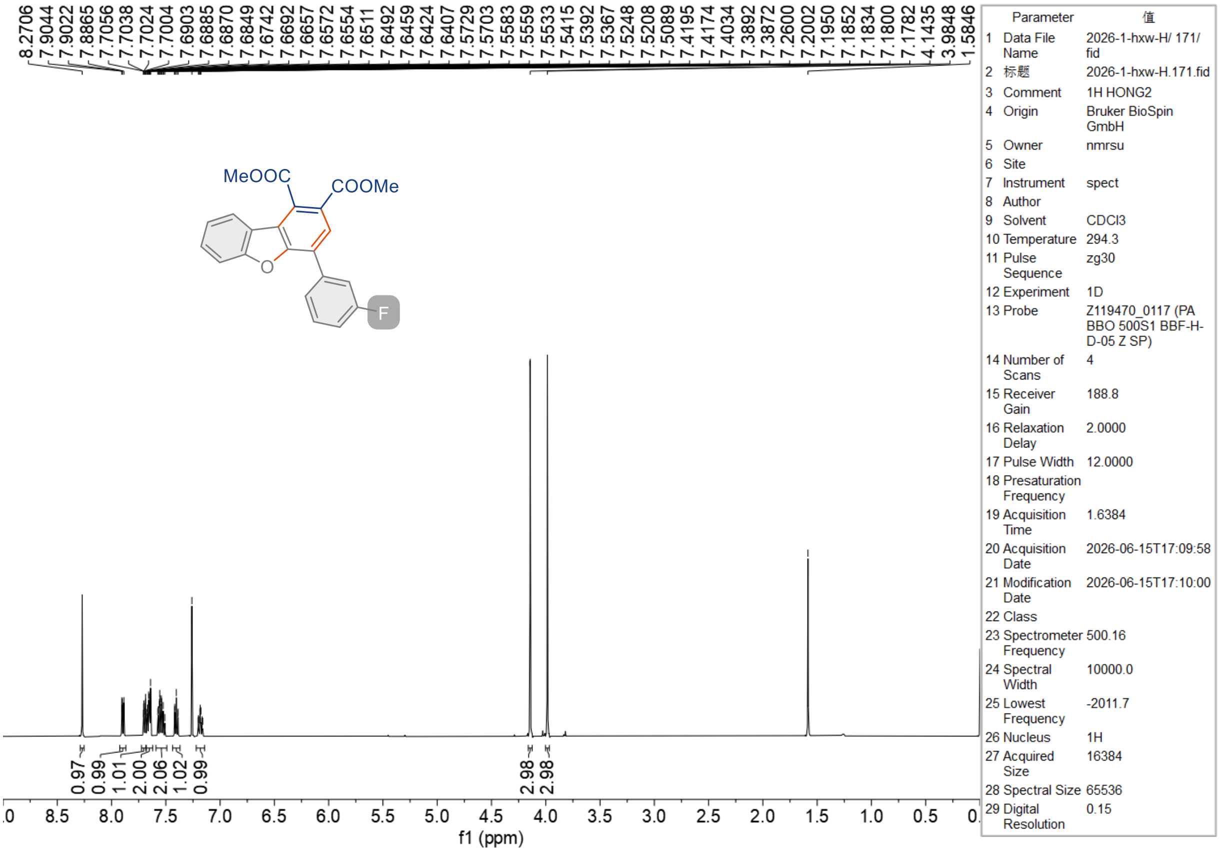

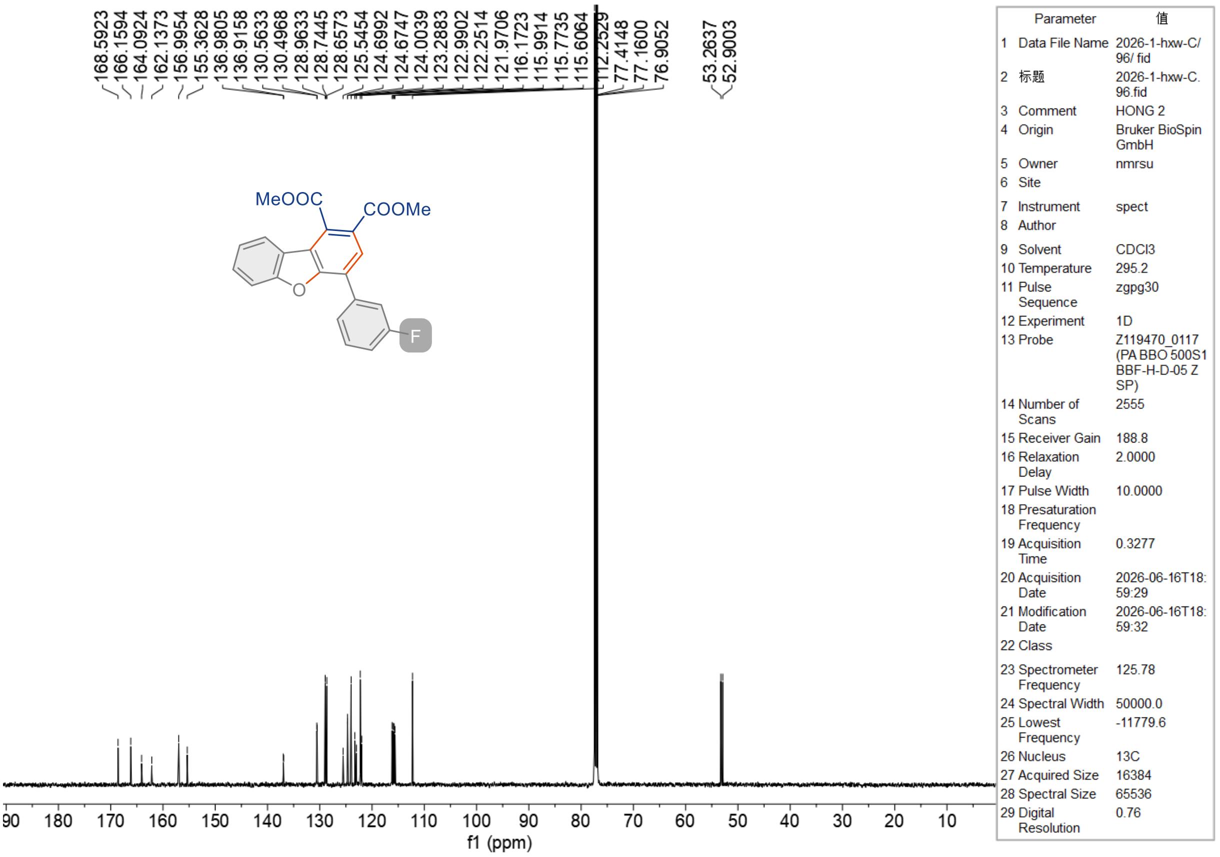

Dimethyl 4-(3-fluorophenyl)dibenzo[*b*,*d*]furan-1,2-dicarboxylate (product 4k)

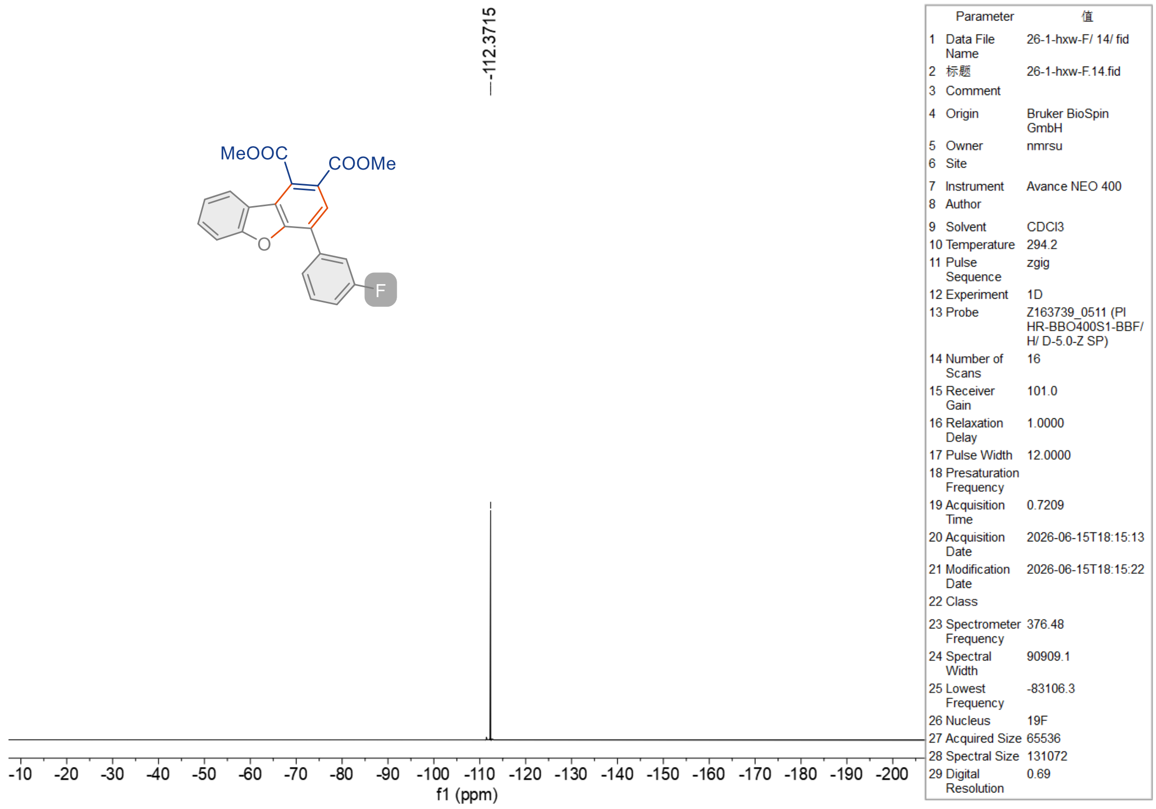

	Parameter	值
1	Data File Name	26-1-hxw-F/ 14/ fid
2	标题	26-1-hxw-F.14.fid
3	Comment	
4	Origin	Bruker BioSpin GmbH
5	Owner	nmrsu
6	Site	
7	Instrument	Avance NEO 400
8	Author	
9	Solvent	CDCl3
10	Temperature	294.2
11	Pulse Sequence	zgig
12	Experiment	1D
13	Probe	Z163739_0511 (PI HR-BBO400S1-BBF/ H/ D-5.0-Z SP)
14	Number of Scans	16
15	Receiver Gain	101.0
16	Relaxation Delay	1.0000
17	Pulse Width	12.0000
18	Presaturation Frequency	
19	Acquisition Time	0.7209
20	Acquisition Date	2026-06-15T18:15:13
21	Modification Date	2026-06-15T18:15:22
22	Class	
23	Spectrometer Frequency	376.48
24	Spectral Width	90909.1
25	Lowest Frequency	-83106.3
26	Nucleus	19F
27	Acquired Size	65536
28	Spectral Size	131072
29	Digital Resolution	0.69

Dimethyl 4-(3-chlorophenyl)dibenzo[*b,d*]furan-1,2-dicarboxylate (product 4l)

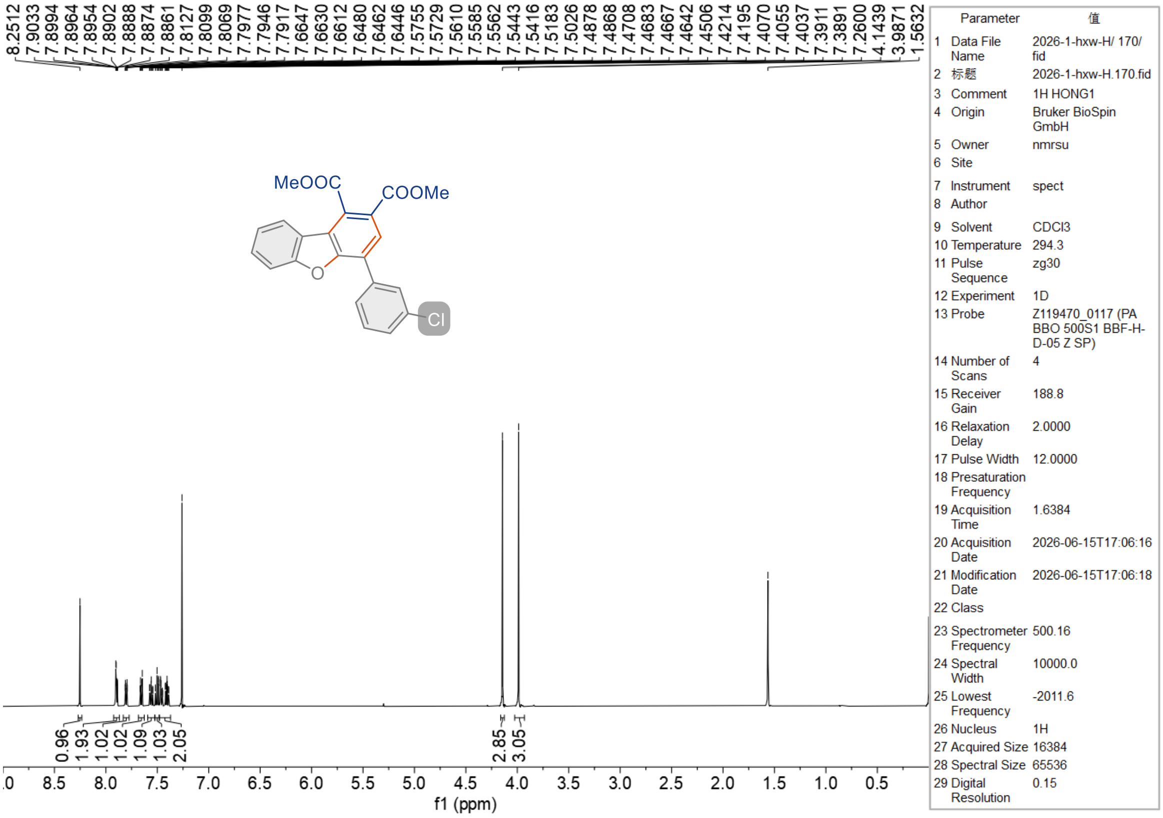

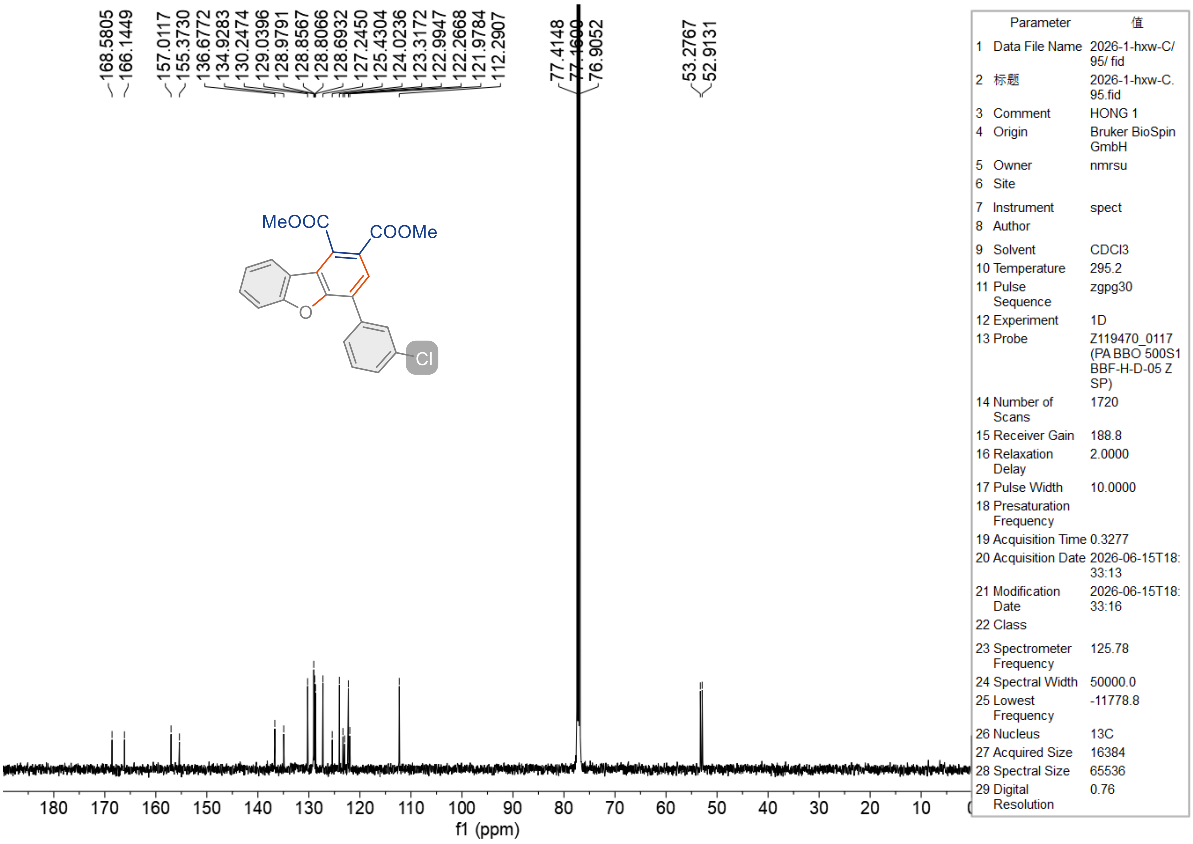

Dimethyl 8-methyl-4-phenyldibenzo[*b*,*d*]furan-1,2-dicarboxylate (product 4m)

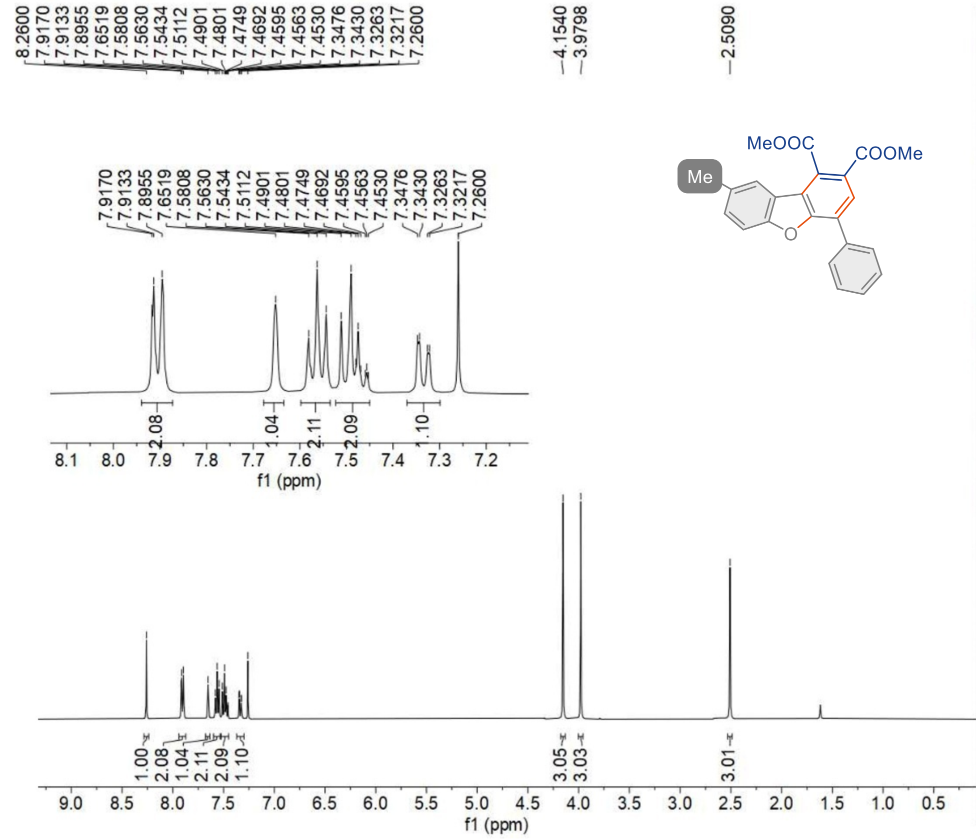

Parameter	值
1 Data File Name	F:/ 旧400/ 2024-2-hxw-H/ 91/ fid
2 标题	2024-2-hxw-H. 91.fid
3 Comment	
4 Origin	Bruker BioSpin GmbH
5 Owner	nmrsu
6 Site	
7 Instrument	Avance NEO 400
8 Author	
9 Solvent	CDCl3
10 Temperature	294.1
11 Pulse Sequence	zg30
12 Experiment	1D
13 Probe	Z163739_0032 (PI HR-400-S1-BBF/ H/ D-5.0-Z SP)
14 Number of Scans	4
15 Receiver Gain	101.0
16 Relaxation Delay	1.0000
17 Pulse Width	10.0000
18 Presaturation Frequency	
19 Acquisition Time	3.9977
20 Acquisition Date	2024-10-18T22:48:26
21 Modification Date	2024-10-18T22:48:08
22 Class	
23 Spectrometer Frequency	400.13
24 Spectral Width	8196.7
25 Lowest Frequency	-1637.3
26 Nucleus	1H
27 Acquired Size	32768
28 Spectral Size	65536
29 Digital Resolution	0.13

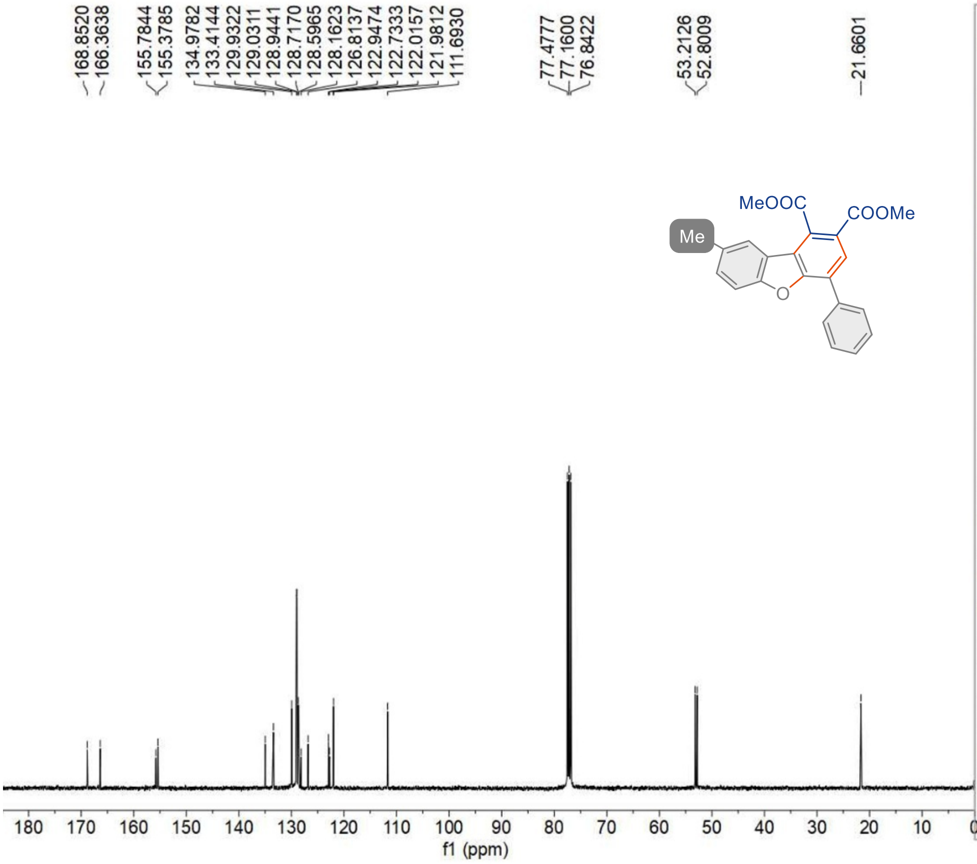

Parameter	值
1 Data File Name	F:/ 旧400/ 2024-2-hxw-C/ 61/ fid
2 标题	2024-2-hxw-C. 61.fid
3 Comment	LV
4 Origin	Bruker BioSpin GmbH
5 Owner	nmrsu
6 Site	
7 Instrument	Avance NEO 400
8 Author	
9 Solvent	CDCl3
10 Temperature	293.7
11 Pulse Sequence	zgpg30
12 Experiment	1D
13 Probe	Z163739_0032 (PI HR-400-S1-BBF/ H/ D-5.0-Z SP)
14 Number of Scans	1500
15 Receiver Gain	17.6
16 Relaxation Delay	2.0000
17 Pulse Width	10.0000
18 Presaturation Frequency	
19 Acquisition Time	1.3763
20 Acquisition Date	2024-10-20T01:18:20
21 Modification Date	2024-10-20T01:17:56
22 Class	
23 Spectrometer Frequency	100.62
24 Spectral Width	23809.5
25 Lowest Frequency	-1834.1
26 Nucleus	13C
27 Acquired Size	32768
28 Spectral Size	65536
29 Digital Resolution	0.36

Dimethyl 8-methoxy-4-phenyldibenzo[*b*,*d*]furan-1,2-dicarboxylate (product 4n)

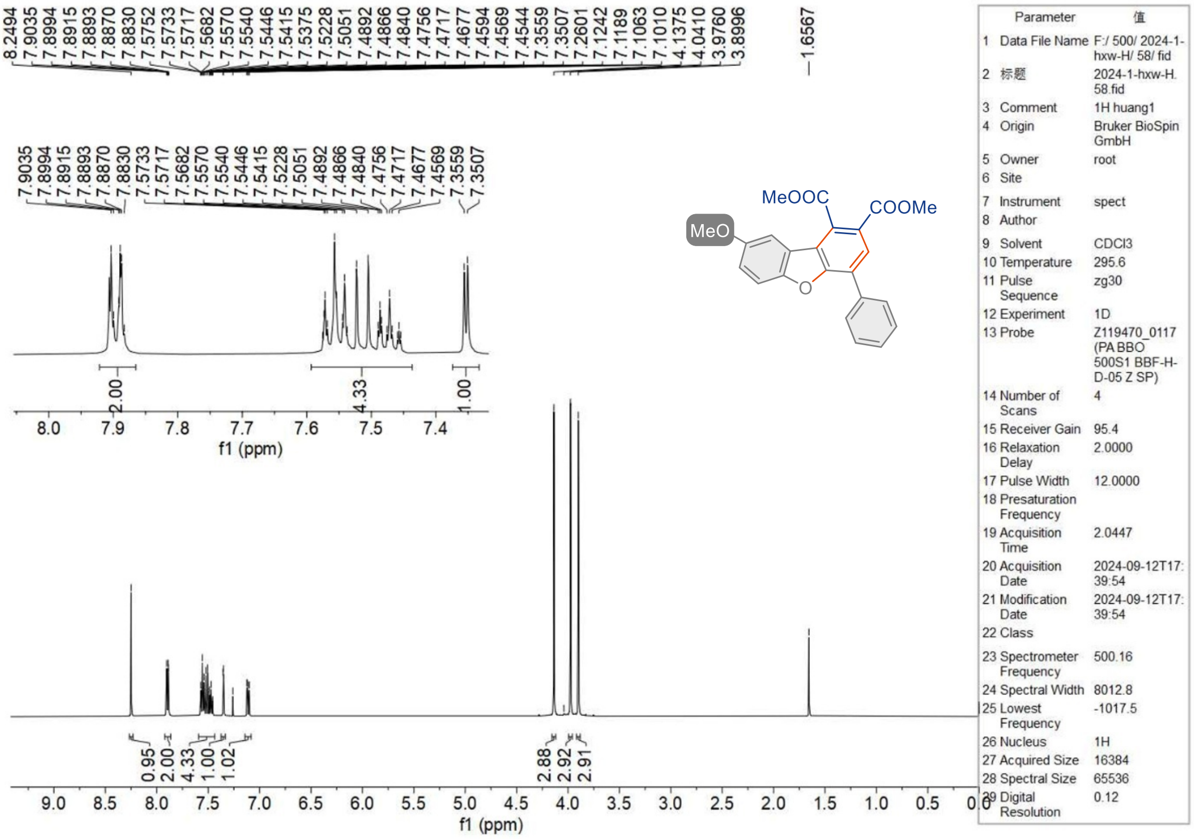

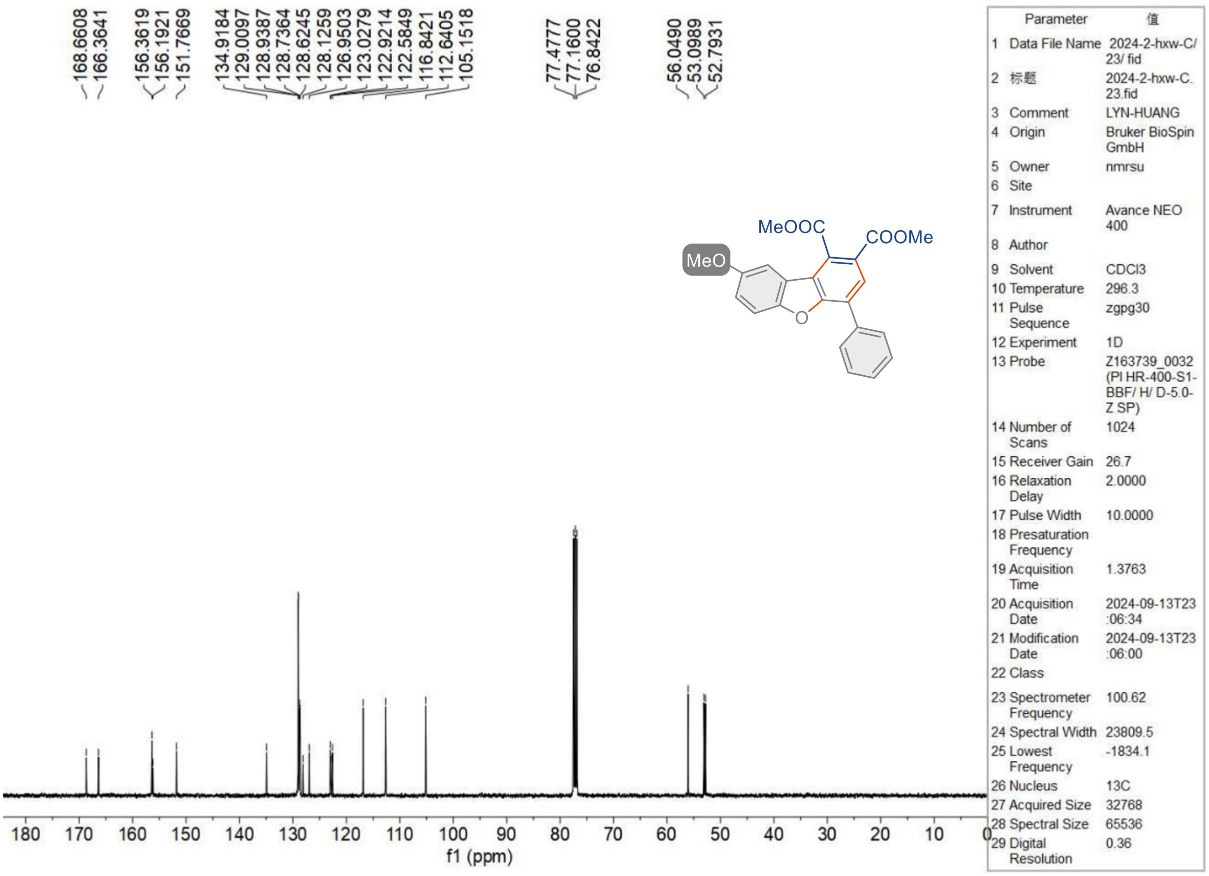

Dimethyl 8-chloro-4-phenyldibenzo[*b,d*]furan-1,2-dicarboxylate (product 4o)

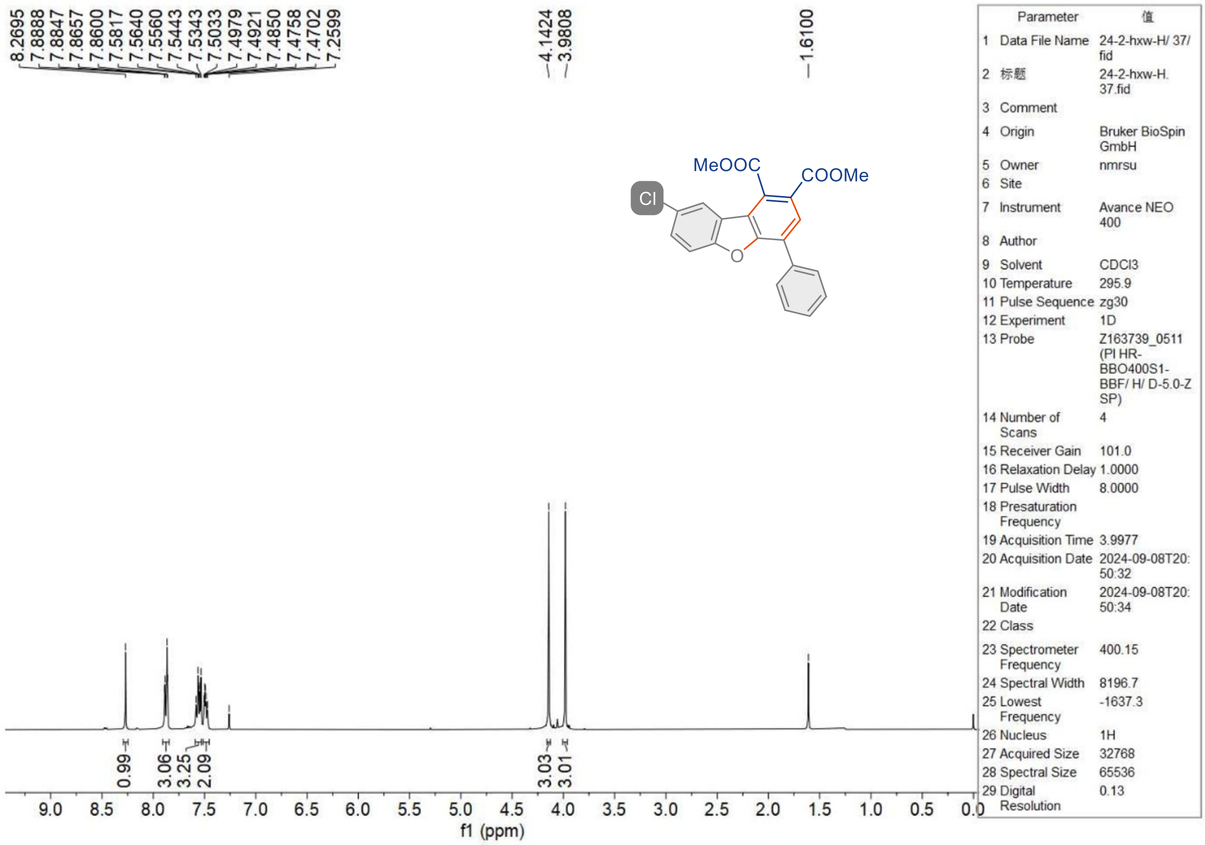

	Parameter	值
1	Data File Name	24-2-hxw-H/ 37/ fid
2	标题	24-2-hxw-H. 37.fid
3	Comment	
4	Origin	Bruker BioSpin GmbH
5	Owner	nmrsu
6	Site	
7	Instrument	Avance NEO 400
8	Author	
9	Solvent	CDCl3
10	Temperature	295.9
11	Pulse Sequence	zg30
12	Experiment	1D
13	Probe	Z163739_0511 (PI HR-BBO400S1-BBF/ H/ D-5.0-Z SP)
14	Number of Scans	4
15	Receiver Gain	101.0
16	Relaxation Delay	1.0000
17	Pulse Width	8.0000
18	Presaturation Frequency	
19	Acquisition Time	3.9977
20	Acquisition Date	2024-09-08T20:50:32
21	Modification Date	2024-09-08T20:50:34
22	Class	
23	Spectrometer Frequency	400.15
24	Spectral Width	8196.7
25	Lowest Frequency	-1637.3
26	Nucleus	1H
27	Acquired Size	32768
28	Spectral Size	65536
29	Digital Resolution	0.13

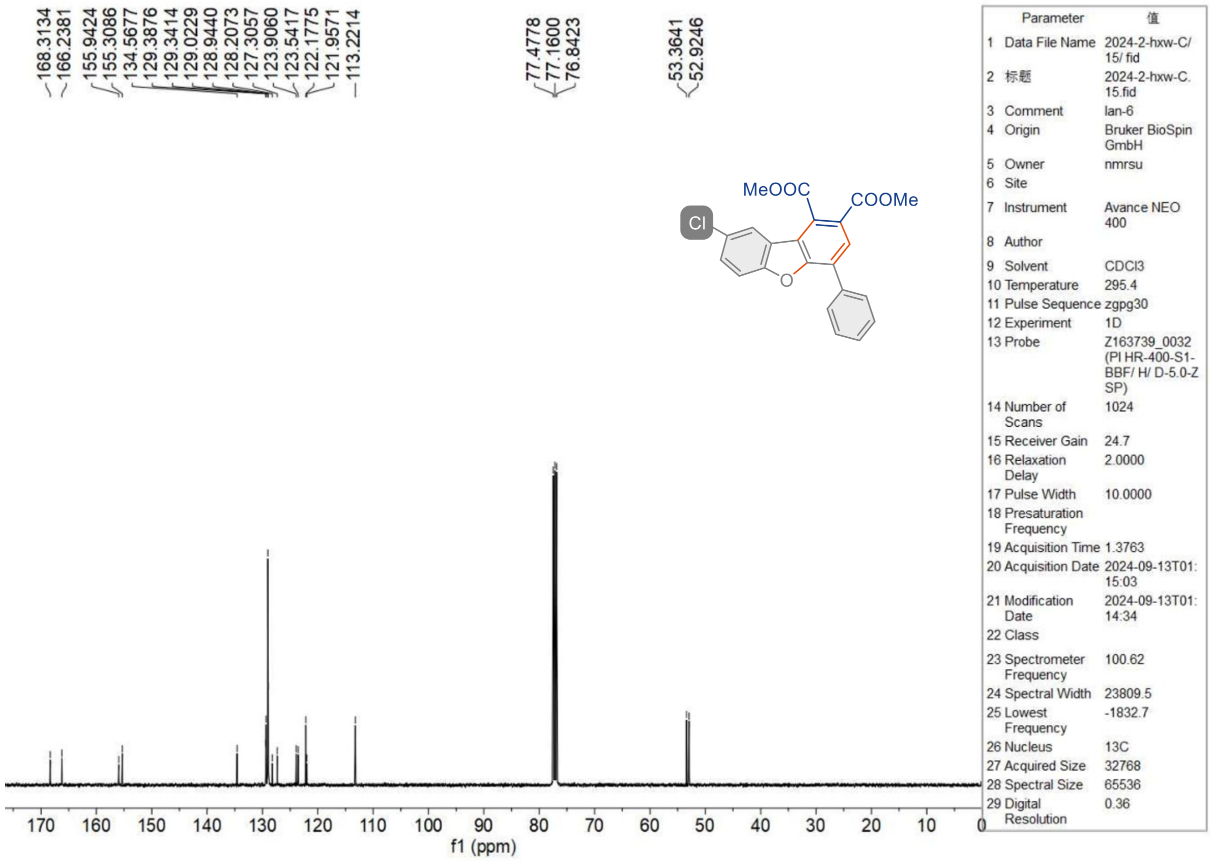

	Parameter	值
1	Data File Name	2024-2-hxw-C/ 15/ fid
2	标题	2024-2-hxw-C. 15.fid
3	Comment	lan-6
4	Origin	Bruker BioSpin GmbH
5	Owner	nmrsu
6	Site	
7	Instrument	Avance NEO 400
8	Author	
9	Solvent	CDCl3
10	Temperature	295.4
11	Pulse Sequence	zgpg30
12	Experiment	1D
13	Probe	Z163739_0032 (PI HR-400-S1-BBF/ H/ D-5.0-Z SP)
14	Number of Scans	1024
15	Receiver Gain	24.7
16	Relaxation Delay	2.0000
17	Pulse Width	10.0000
18	Presaturation Frequency	
19	Acquisition Time	1.3763
20	Acquisition Date	2024-09-13T01:15:03
21	Modification Date	2024-09-13T01:14:34
22	Class	
23	Spectrometer Frequency	100.62
24	Spectral Width	23809.5
25	Lowest Frequency	-1832.7
26	Nucleus	13C
27	Acquired Size	32768
28	Spectral Size	65536
29	Digital Resolution	0.36

Dimethyl 8-fluoro-4-phenyldibenzo[*b,d*]furan-1,2-dicarboxylate (product 4p)

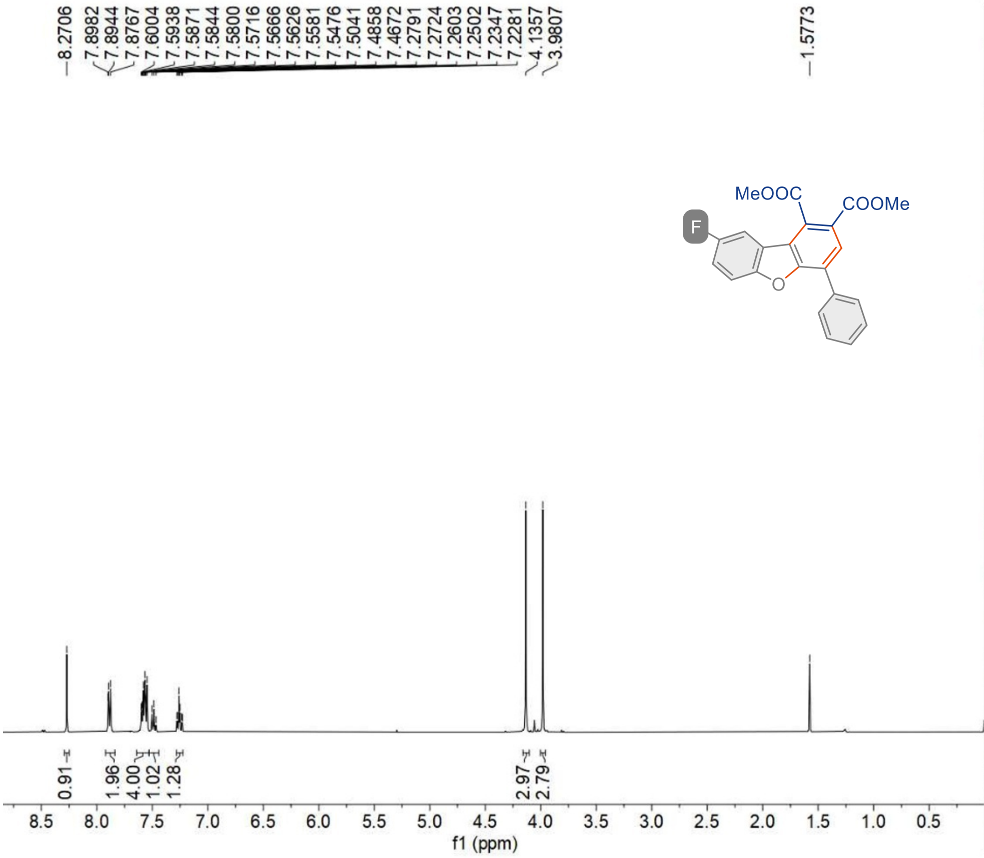

Parameter	值
1 Data File Name	2024-1-hxw-H/227/ fid
2 标题	2024-1-hxw-H.227.fid
3 Comment	
4 Origin	Bruker BioSpin GmbH
5 Owner	nmrsu
6 Site	
7 Instrument	Avance NEO 400
8 Author	
9 Solvent	CDCl3
10 Temperature	299.2
11 Pulse Sequence	zg30
12 Experiment	1D
13 Probe	Z163739_0032 (PI HR-400-S1-BBF/ H/ D-5.0-Z SP)
14 Number of Scans	4
15 Receiver Gain	101.0
16 Relaxation Delay	1.0000
17 Pulse Width	10.0000
18 Presaturation Frequency	
19 Acquisition Time	3.9977
20 Acquisition Date	2024-07-23T16:50:34
21 Modification Date	2024-07-23T16:49:42
22 Class	
23 Spectrometer Frequency	400.13
24 Spectral Width	8196.7
25 Lowest Frequency	-1637.3
26 Nucleus	1H
27 Acquired Size	32768
28 Spectral Size	65536
29 Digital Resolution	0.13

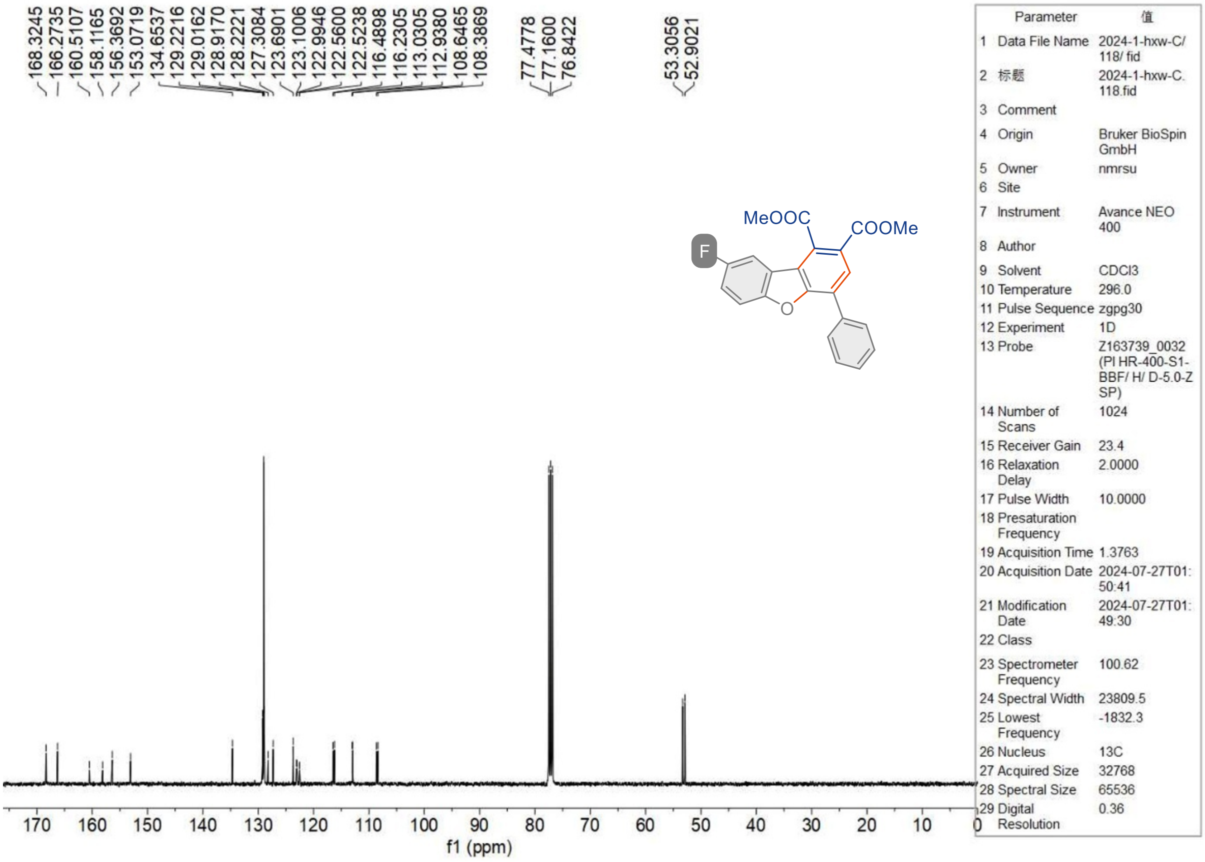

Parameter	值
1 Data File Name	2024-1-hxw-C/118/ fid
2 标题	2024-1-hxw-C.118.fid
3 Comment	
4 Origin	Bruker BioSpin GmbH
5 Owner	nmrsu
6 Site	
7 Instrument	Avance NEO 400
8 Author	
9 Solvent	CDCl3
10 Temperature	296.0
11 Pulse Sequence	zgpg30
12 Experiment	1D
13 Probe	Z163739_0032 (PI HR-400-S1-BBF/ H/ D-5.0-Z SP)
14 Number of Scans	1024
15 Receiver Gain	23.4
16 Relaxation Delay	2.0000
17 Pulse Width	10.0000
18 Presaturation Frequency	
19 Acquisition Time	1.3763
20 Acquisition Date	2024-07-27T01:50:41
21 Modification Date	2024-07-27T01:49:30
22 Class	
23 Spectrometer Frequency	100.62
24 Spectral Width	23809.5
25 Lowest Frequency	-1832.3
26 Nucleus	13C
27 Acquired Size	32768
28 Spectral Size	65536
29 Digital Resolution	0.36

Dimethyl 8-fluoro-4-phenyldibenzo[*b,d*]furan-1,2-dicarboxylate (product 4p)

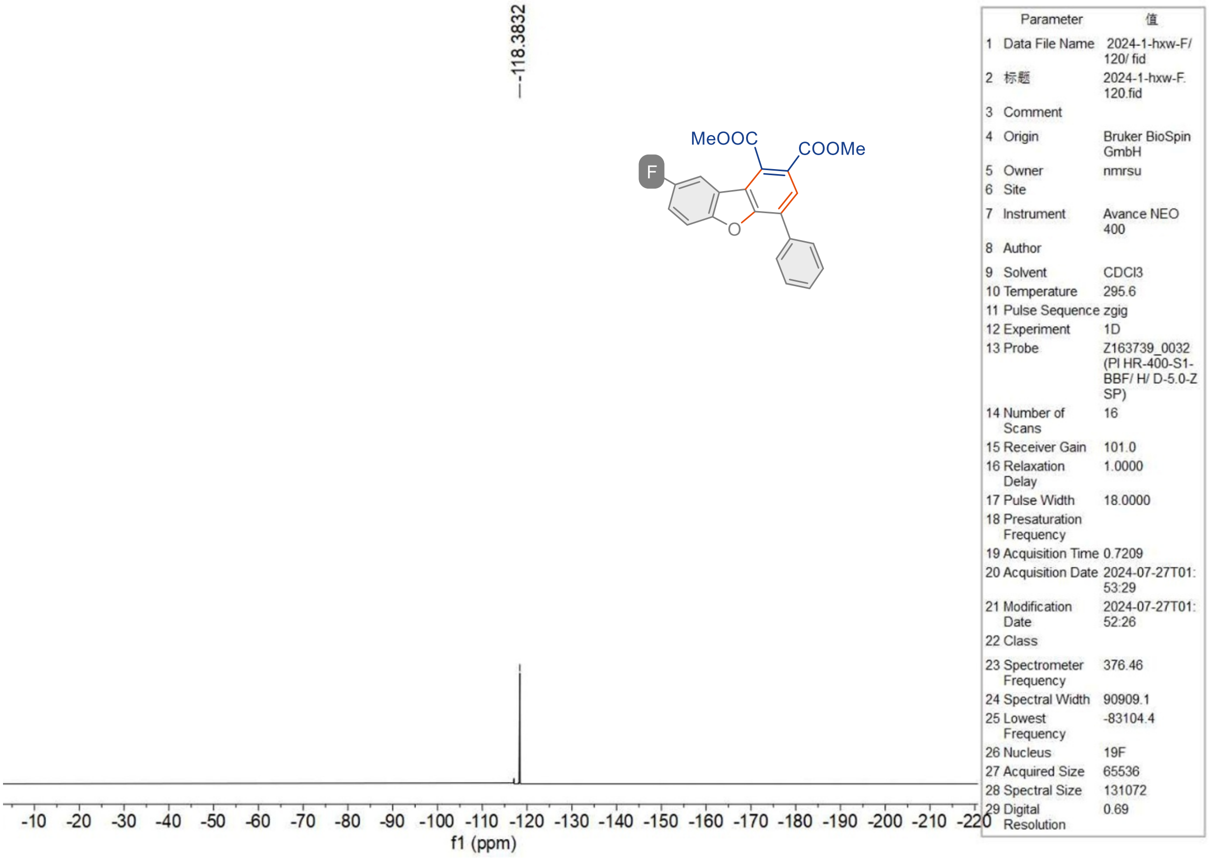

	Parameter	值
1	Data File Name	2024-1-hxw-F/ 120/ fid
2	标题	2024-1-hxw-F. 120.fid
3	Comment	
4	Origin	Bruker BioSpin GmbH
5	Owner	nmrsu
6	Site	
7	Instrument	Avance NEO 400
8	Author	
9	Solvent	CDCl3
10	Temperature	295.6
11	Pulse Sequence	zgig
12	Experiment	1D
13	Probe	Z163739_0032 (PI HR-400-S1-BBF/ H/ D-5.0-Z SP)
14	Number of Scans	16
15	Receiver Gain	101.0
16	Relaxation Delay	1.0000
17	Pulse Width	18.0000
18	Presaturation Frequency	
19	Acquisition Time	0.7209
20	Acquisition Date	2024-07-27T01:53:29
21	Modification Date	2024-07-27T01:52:26
22	Class	
23	Spectrometer Frequency	376.46
24	Spectral Width	90909.1
25	Lowest Frequency	-83104.4
26	Nucleus	19F
27	Acquired Size	65536
28	Spectral Size	131072
29	Digital Resolution	0.69

Dimethyl 8-iodo-4-phenyldibenzo[*b*,*d*]furan-1,2-dicarboxylate (product 4q)

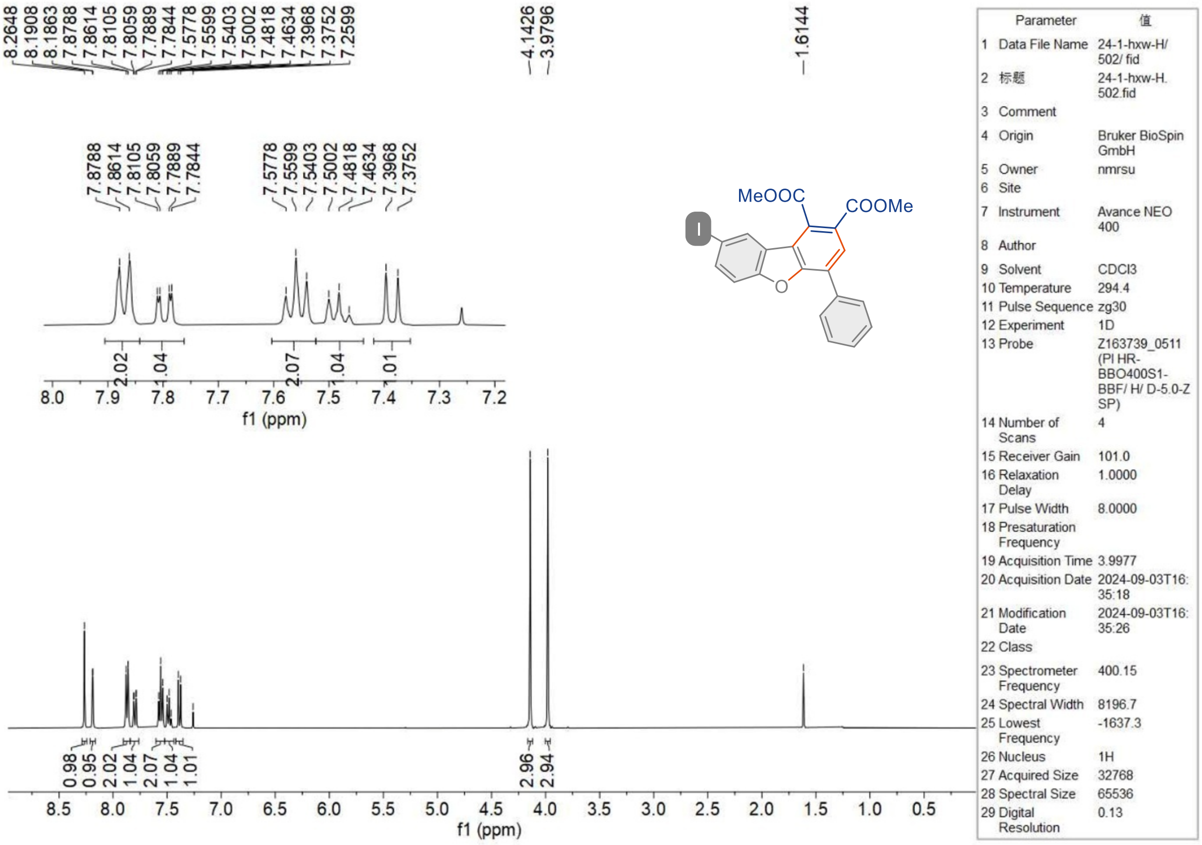

Parameter	值
1 Data File Name	24-1-hxw-H/502/ fid
2 标题	24-1-hxw-H.502.fid
3 Comment	
4 Origin	Bruker BioSpin GmbH
5 Owner	nmrsu
6 Site	
7 Instrument	Avance NEO 400
8 Author	
9 Solvent	CDCl3
10 Temperature	294.4
11 Pulse Sequence	zg30
12 Experiment	1D
13 Probe	Z163739_0511 (PI HR-BBO400S1-BBF/ H/ D-5.0-Z SP)
14 Number of Scans	4
15 Receiver Gain	101.0
16 Relaxation Delay	1.0000
17 Pulse Width	8.0000
18 Presaturation Frequency	
19 Acquisition Time	3.9977
20 Acquisition Date	2024-09-03T16:35:18
21 Modification Date	2024-09-03T16:35:26
22 Class	
23 Spectrometer Frequency	400.15
24 Spectral Width	8196.7
25 Lowest Frequency	-1637.3
26 Nucleus	1H
27 Acquired Size	32768
28 Spectral Size	65536
29 Digital Resolution	0.13

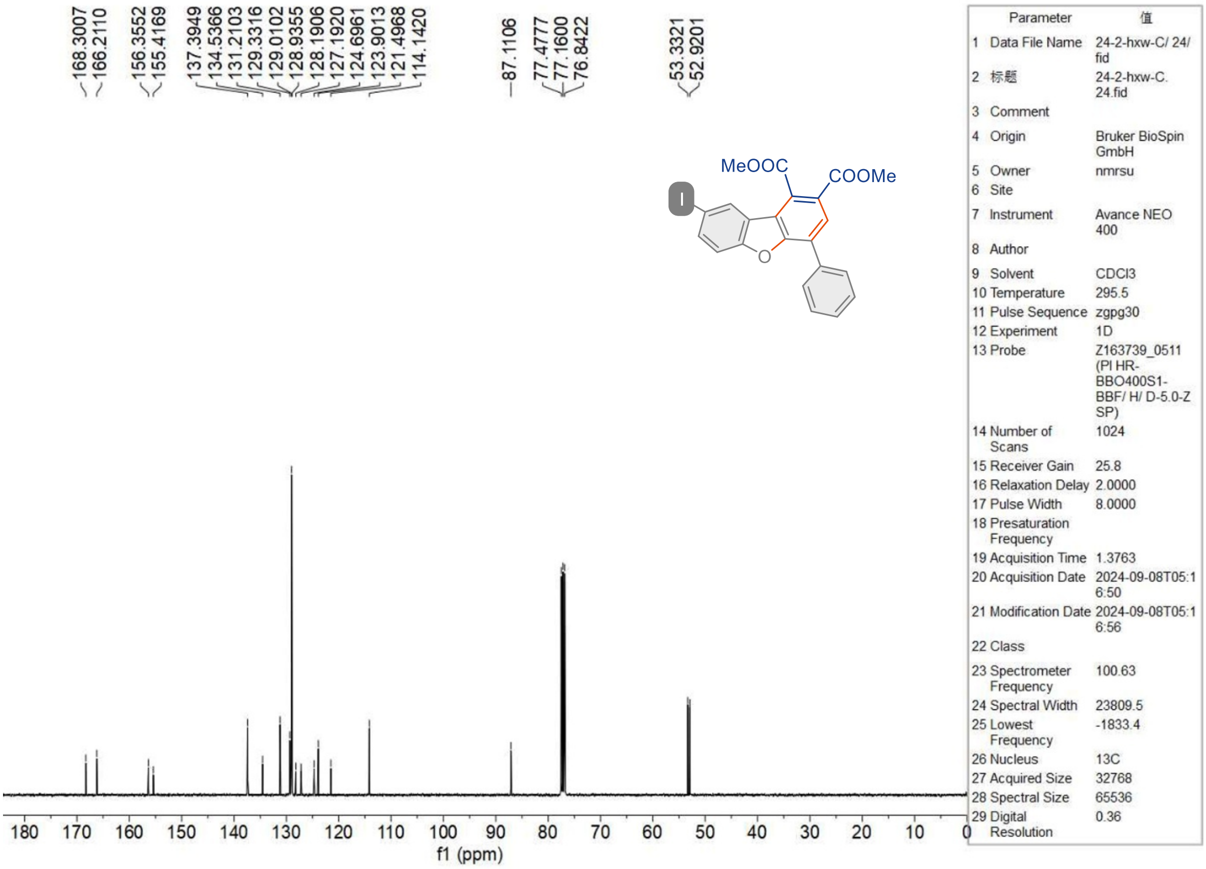

Parameter	值
1 Data File Name	24-2-hxw-C/ 24/ fid
2 标题	24-2-hxw-C.24.fid
3 Comment	
4 Origin	Bruker BioSpin GmbH
5 Owner	nmrsu
6 Site	
7 Instrument	Avance NEO 400
8 Author	
9 Solvent	CDCl3
10 Temperature	295.5
11 Pulse Sequence	zgpg30
12 Experiment	1D
13 Probe	Z163739_0511 (PI HR-BBO400S1-BBF/ H/ D-5.0-Z SP)
14 Number of Scans	1024
15 Receiver Gain	25.8
16 Relaxation Delay	2.0000
17 Pulse Width	8.0000
18 Presaturation Frequency	
19 Acquisition Time	1.3763
20 Acquisition Date	2024-09-08T05:16:50
21 Modification Date	2024-09-08T05:16:56
22 Class	
23 Spectrometer Frequency	100.63
24 Spectral Width	23809.5
25 Lowest Frequency	-1833.4
26 Nucleus	13C
27 Acquired Size	32768
28 Spectral Size	65536
29 Digital Resolution	0.36

Dimethyl 8-bromo-4-phenyldibenzo[*b*,*d*]furan-1,2-dicarboxylate (product 4r)

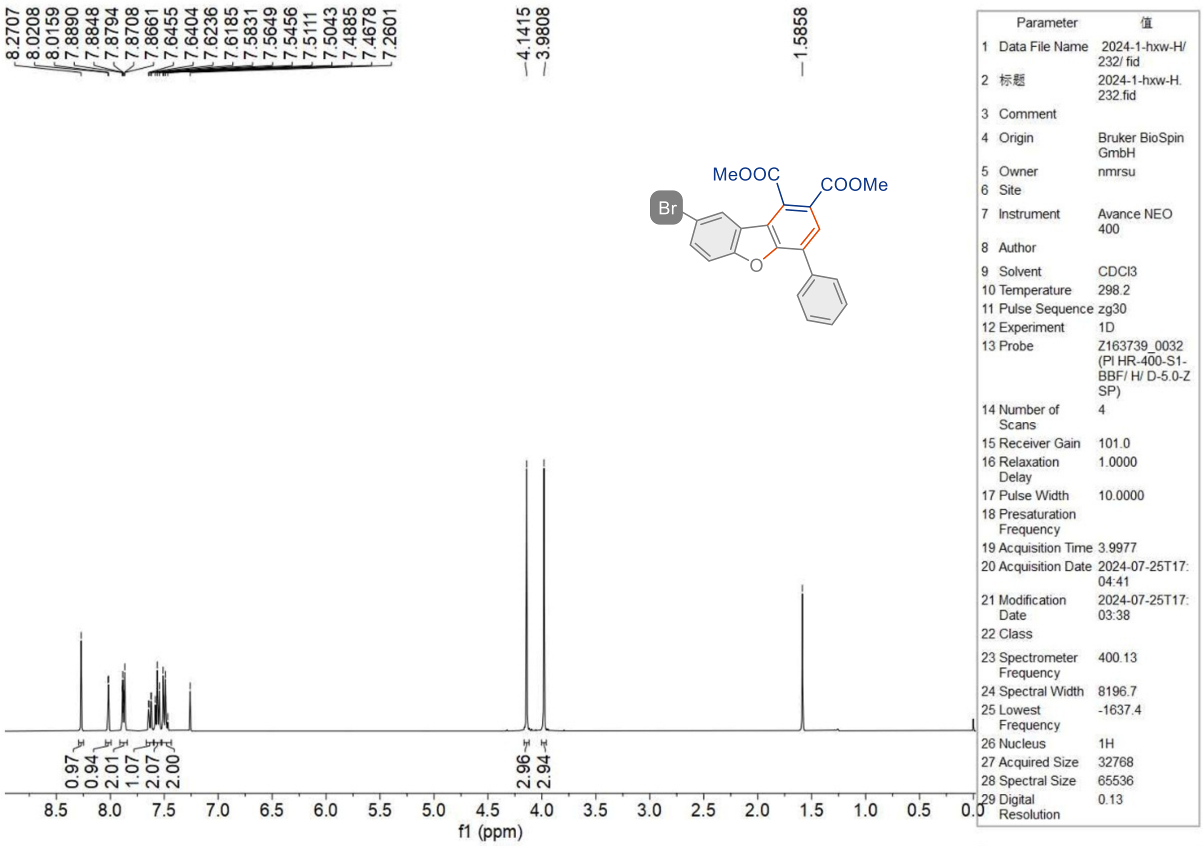

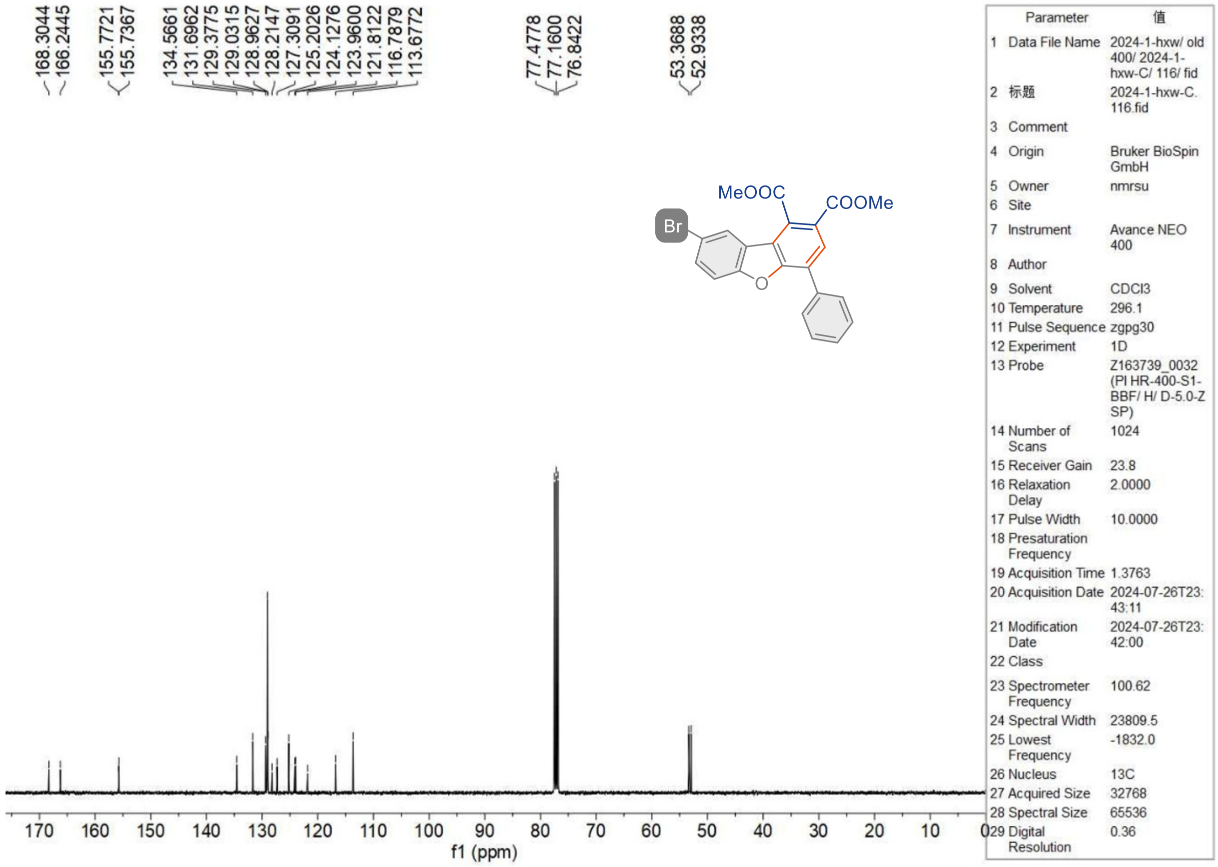

Dimethyl 6,8-dibromo-4-phenyldibenzo[*b*,*d*]furan-1,2-dicarboxylate (product 4s)

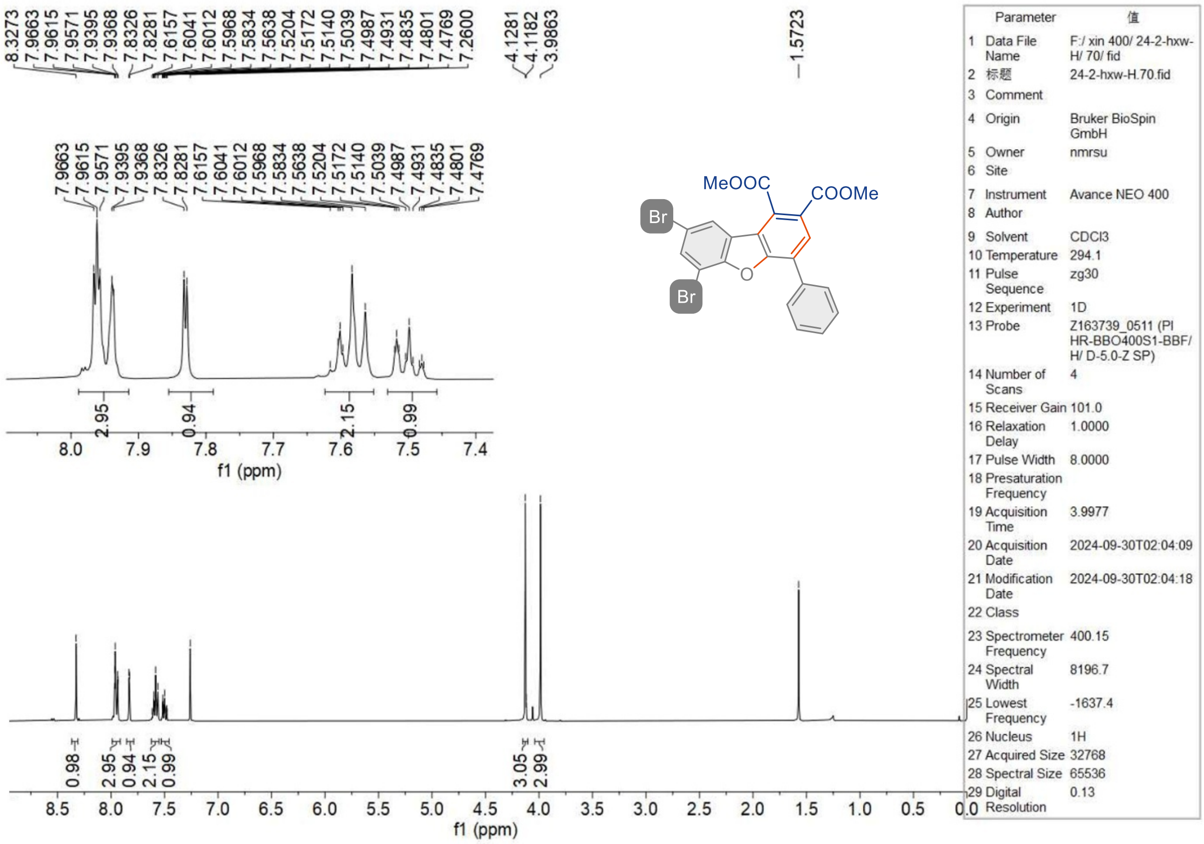

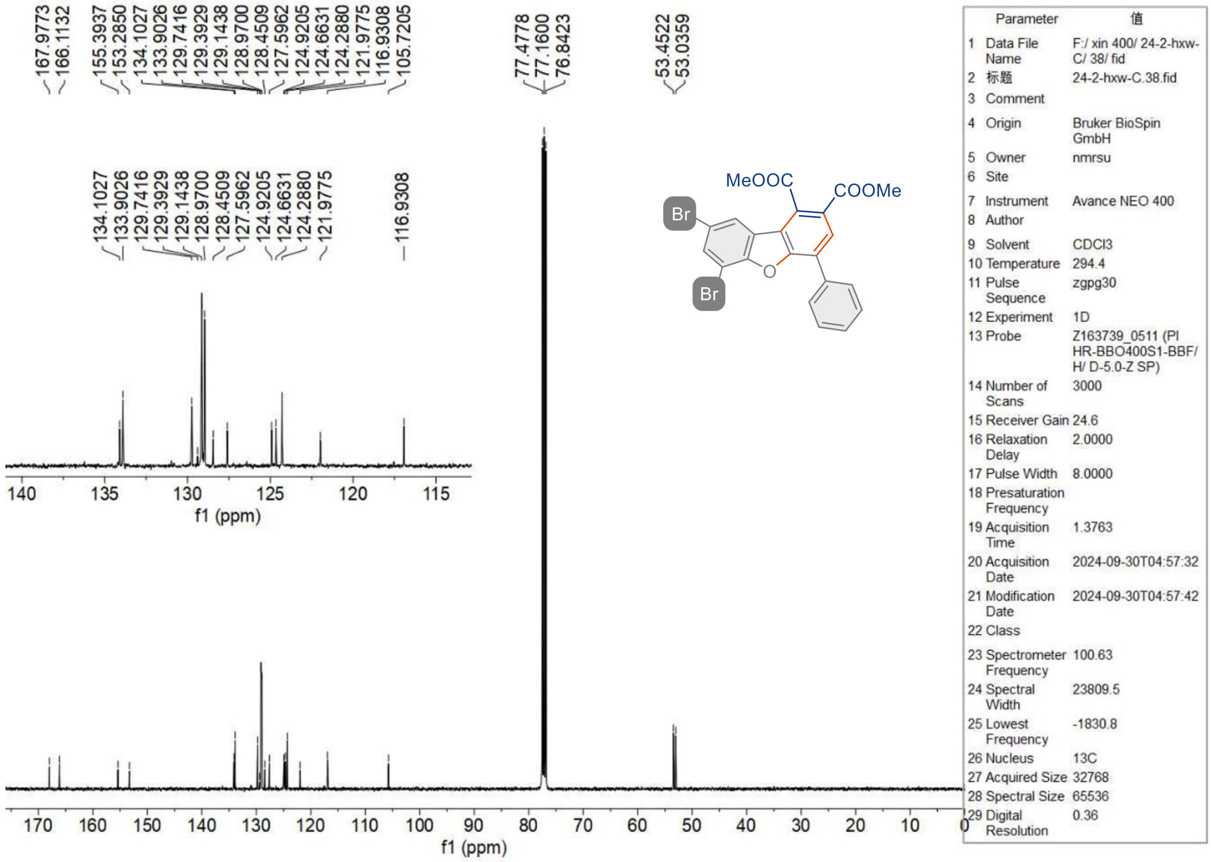

Dimethyl 4-(4-methoxyphenyl)-8-methyldibenzo[*b*,*d*]furan-1,2-dicarboxylate (product 4t)

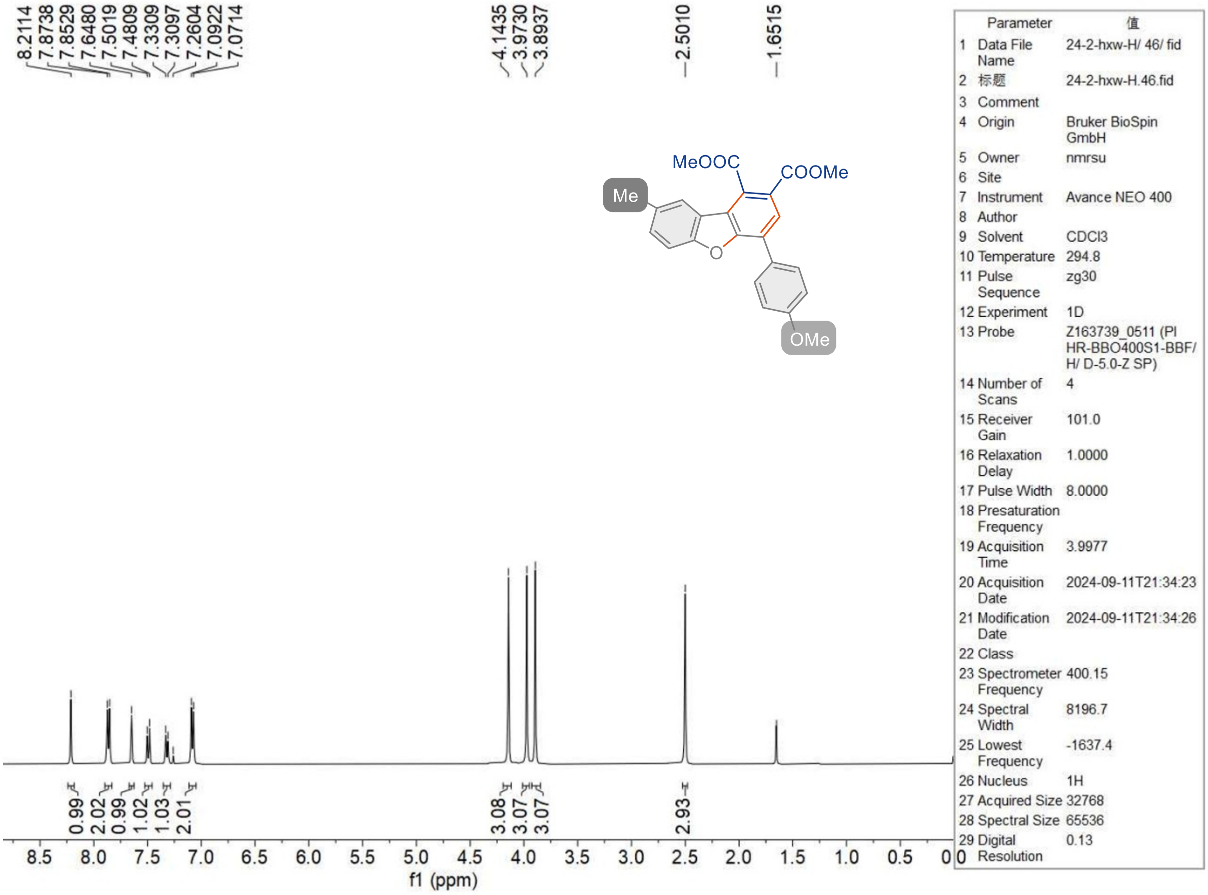

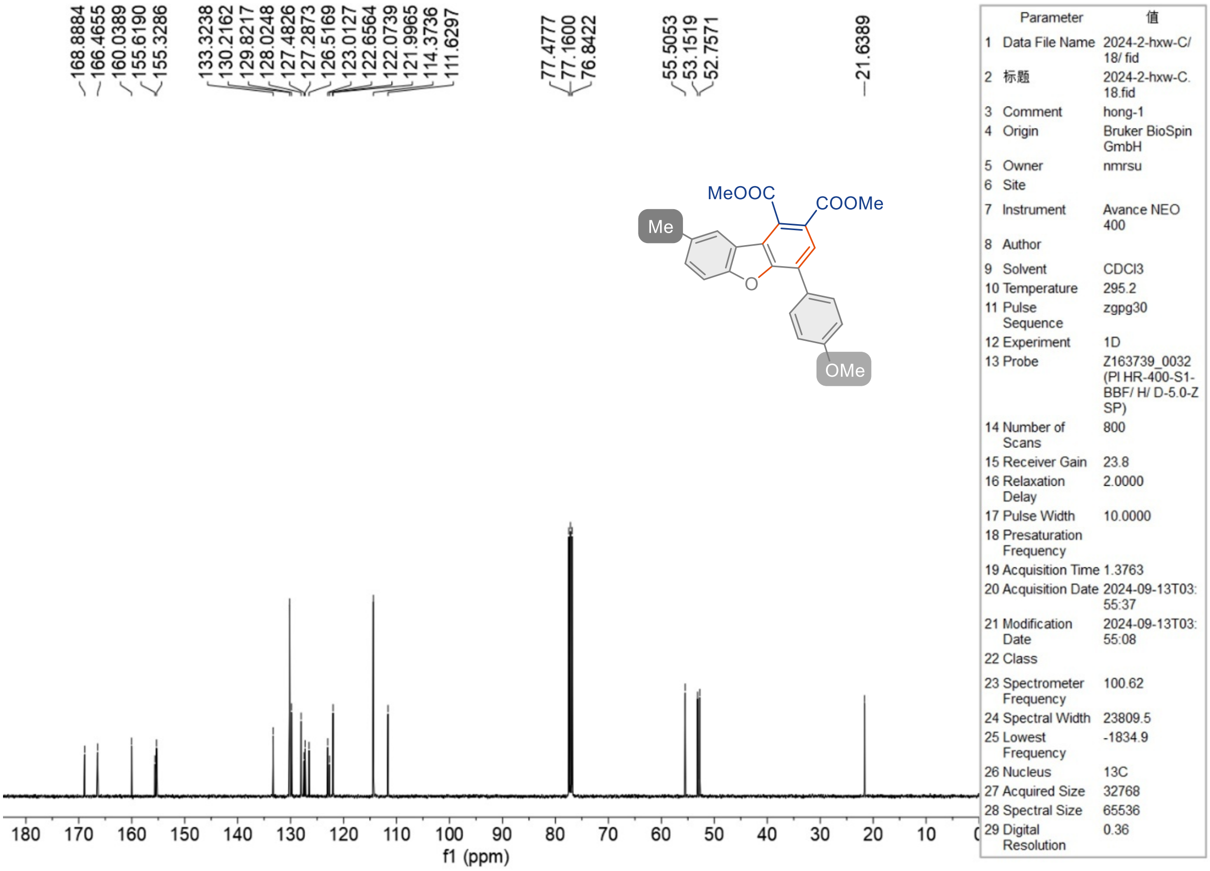

Dimethyl 4-(4-fluorophenyl)-8-methyldibenzo[*b*,*d*]furan-1,2 dicarboxylate (product 4u)

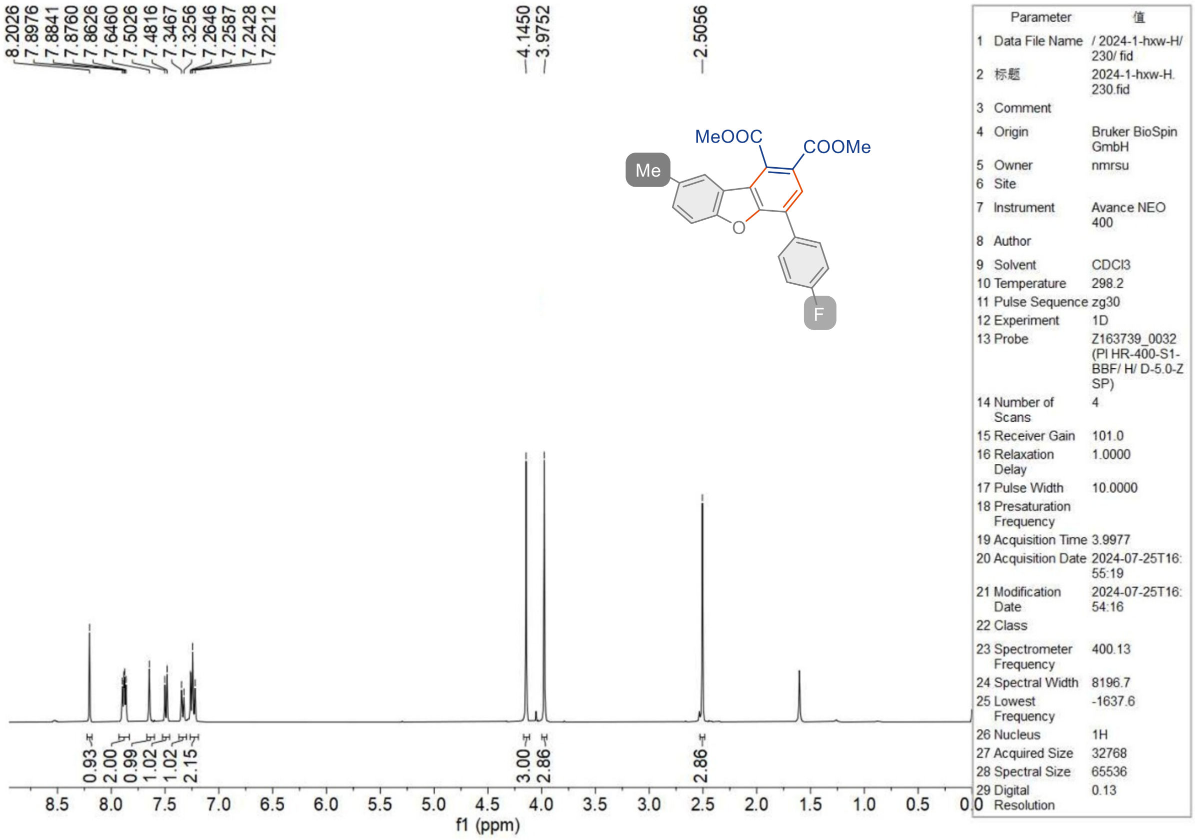

	Parameter	值
1	Data File Name	/ 2024-1-hxw-H/ 230/ fid
2	标题	2024-1-hxw-H. 230.fid
3	Comment	
4	Origin	Bruker BioSpin GmbH
5	Owner	nmrsu
6	Site	
7	Instrument	Avance NEO 400
8	Author	
9	Solvent	CDCl3
10	Temperature	298.2
11	Pulse Sequence	zg30
12	Experiment	1D
13	Probe	Z163739_0032 (PI HR-400-S1-BBF/ H/ D-5.0-Z SP)
14	Number of Scans	4
15	Receiver Gain	101.0
16	Relaxation Delay	1.0000
17	Pulse Width	10.0000
18	Presaturation Frequency	
19	Acquisition Time	3.9977
20	Acquisition Date	2024-07-25T16:55:19
21	Modification Date	2024-07-25T16:54:16
22	Class	
23	Spectrometer Frequency	400.13
24	Spectral Width	8196.7
25	Lowest Frequency	-1637.6
26	Nucleus	1H
27	Acquired Size	32768
28	Spectral Size	65536
29	Digital Resolution	0.13

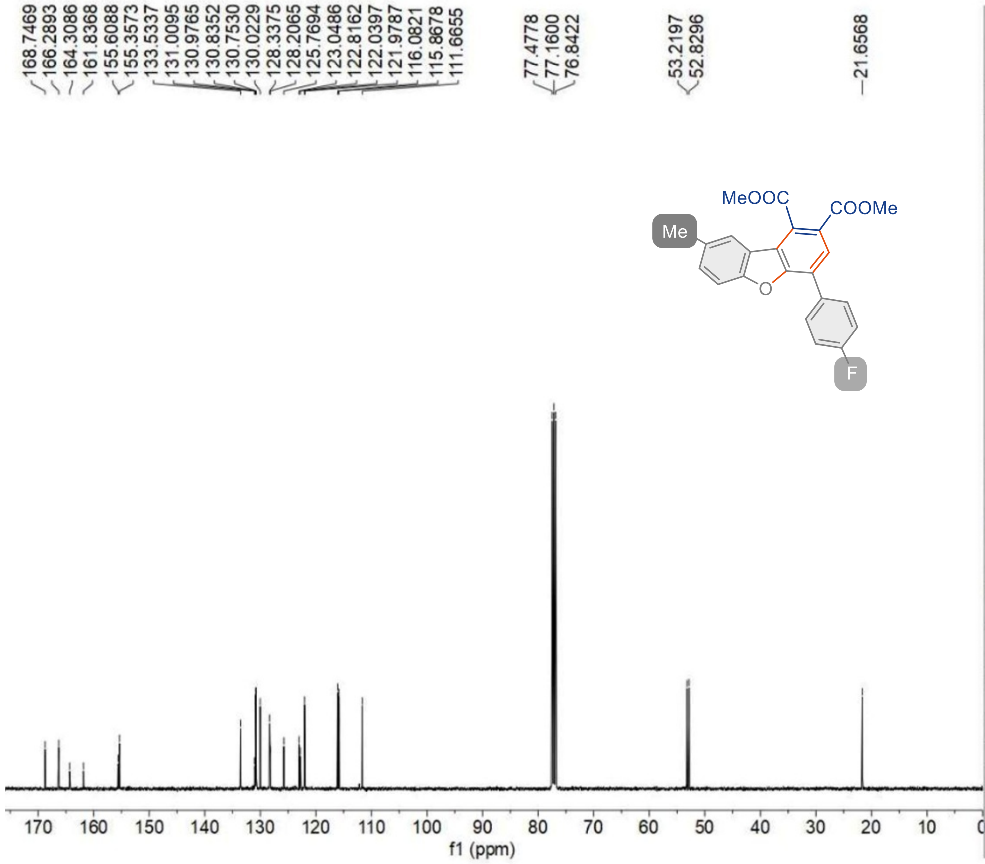

	Parameter	值
1	Data File Name	2024-1-hxw-C/ 119/ fid
2	标题	2024-1-hxw-C. 119.fid
3	Comment	
4	Origin	Bruker BioSpin GmbH
5	Owner	nmrsu
6	Site	
7	Instrument	Avance NEO 400
8	Author	
9	Solvent	CDCl3
10	Temperature	295.9
11	Pulse Sequence	zgpg30
12	Experiment	1D
13	Probe	Z163739_0032 (PI HR-400-S1-BBF/ H/ D-5.0-Z SP)
14	Number of Scans	1024
15	Receiver Gain	24.7
16	Relaxation Delay	2.0000
17	Pulse Width	10.0000
18	Presaturation Frequency	
19	Acquisition Time	1.3763
20	Acquisition Date	2024-07-27T02:58:36
21	Modification Date	2024-07-27T02:57:24
22	Class	
23	Spectrometer Frequency	100.62
24	Spectral Width	23809.5
25	Lowest Frequency	-1832.3
26	Nucleus	13C
27	Acquired Size	32768
28	Spectral Size	65536
29	Digital Resolution	0.36

Dimethyl 4-(4-fluorophenyl)-8-methyldibenzo[*b*,*d*]furan-1,2 dicarboxylate (product 4u)

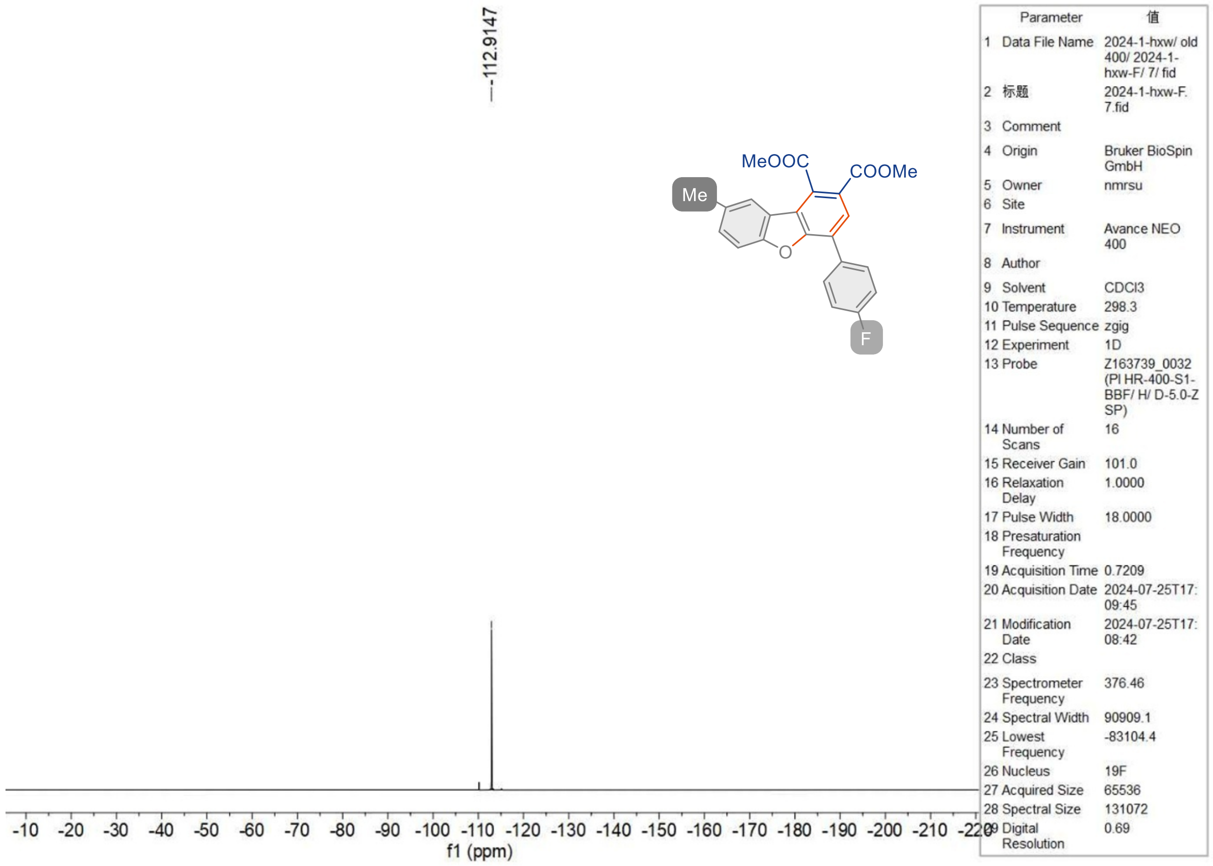

Dimethyl 4-(4-chlorophenyl)-8-methoxydibenzo[*b*,*d*]furan-1,2-dicarboxylate (product 4v)

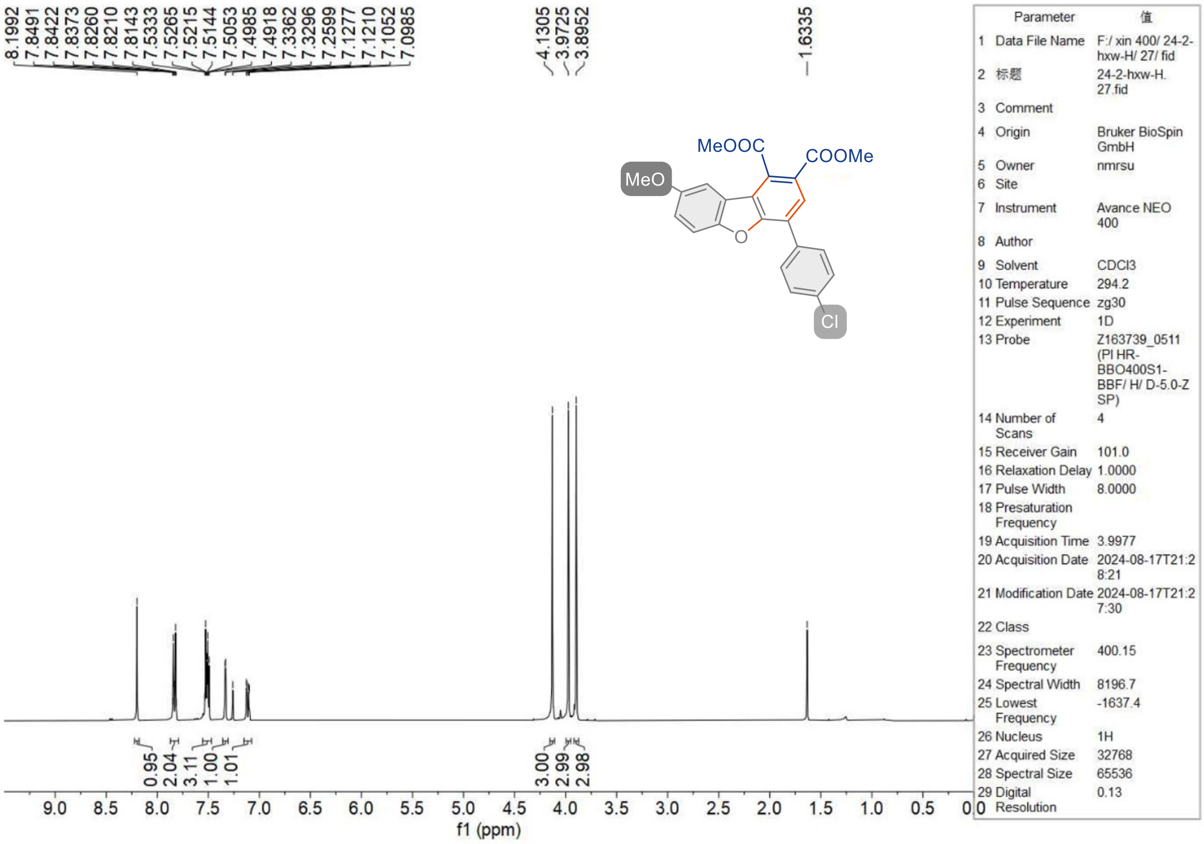

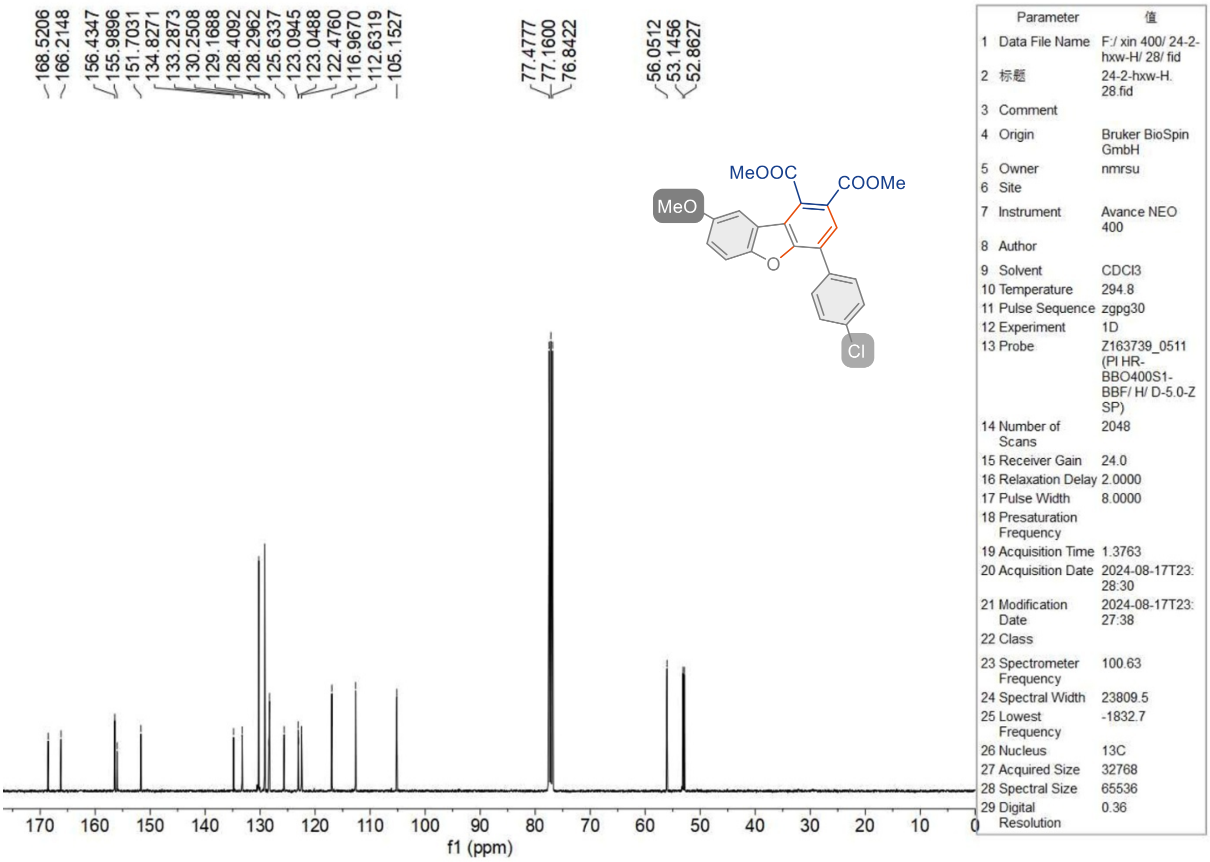

Dimethyl 8-chloro-4-(*p*-tolyl)dibenzo[*b*,*d*]furan-1,2-dicarboxylate (product 4w)

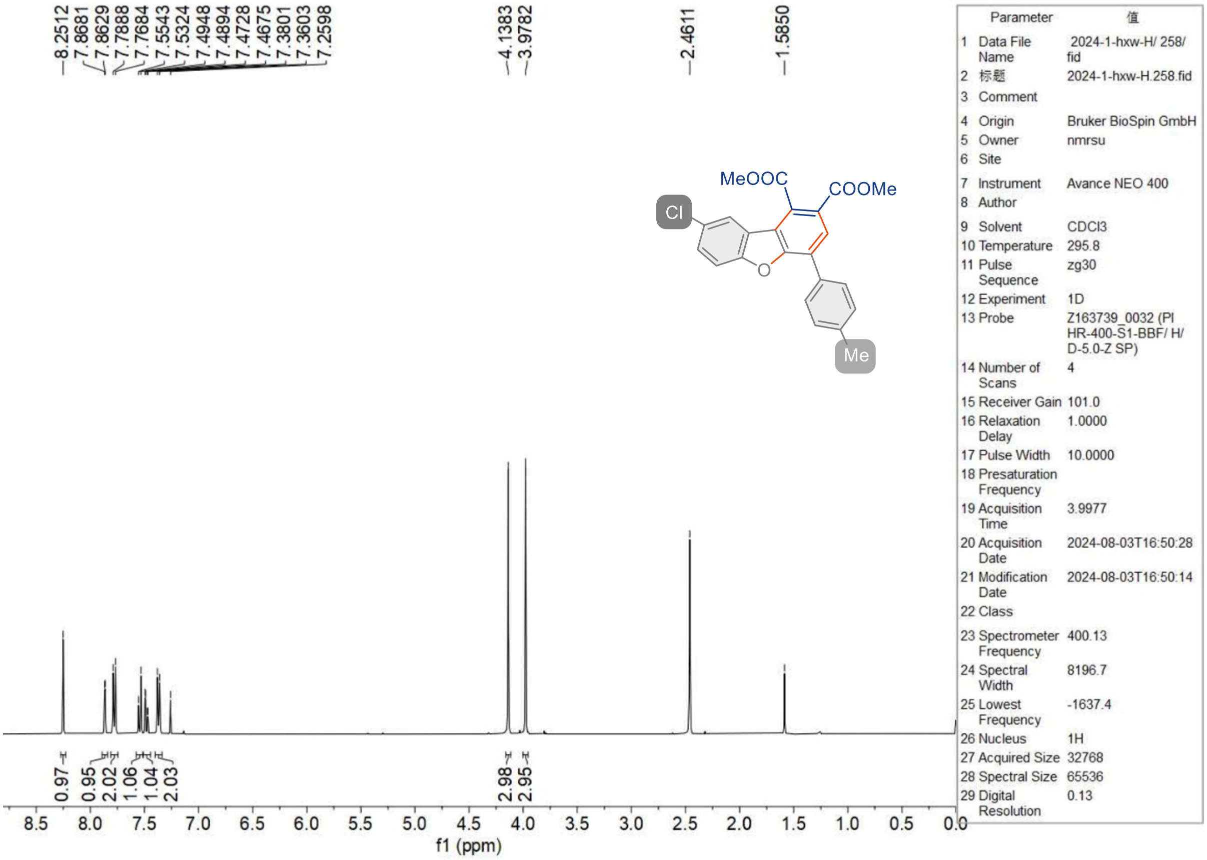

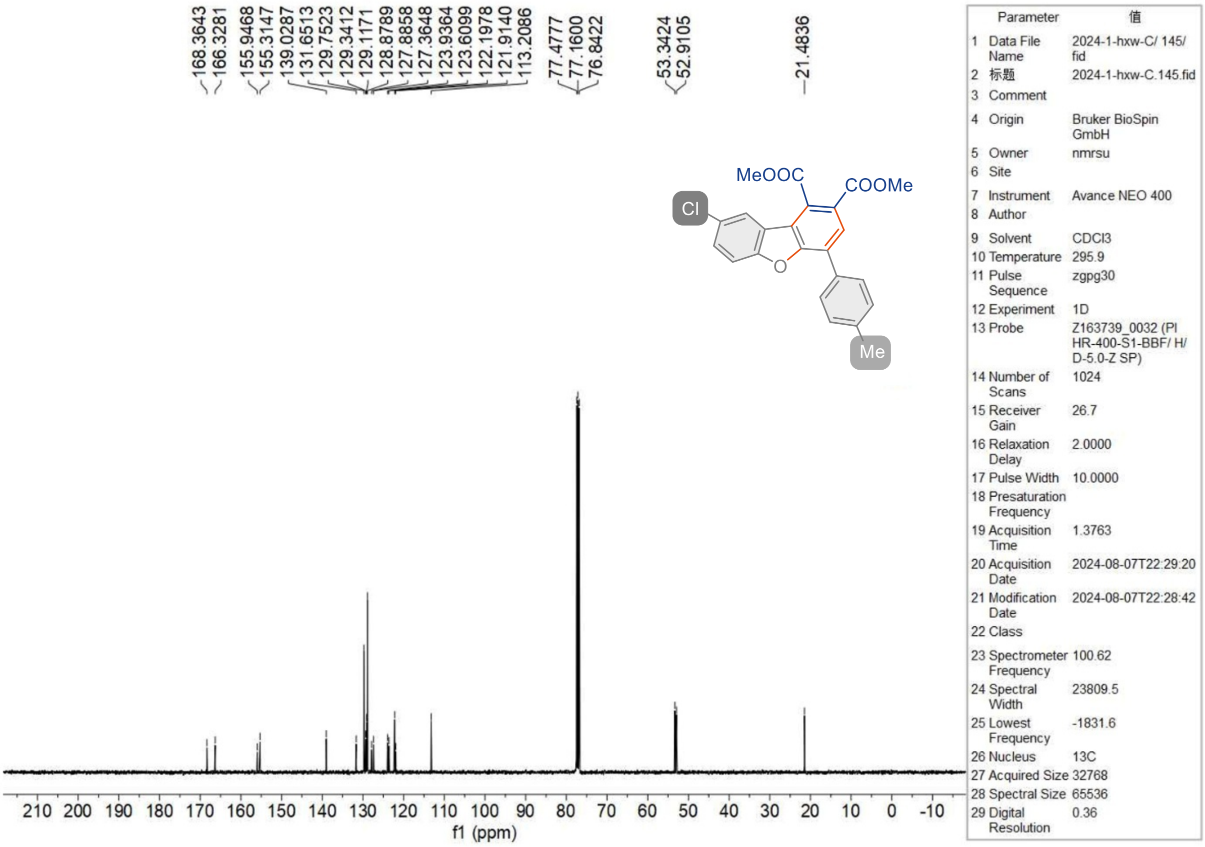

Dimethyl 8-chloro-4-(4-methoxyphenyl)dibenzo[*b*,*d*]furan-1,2-dicarboxylate (product 4x)

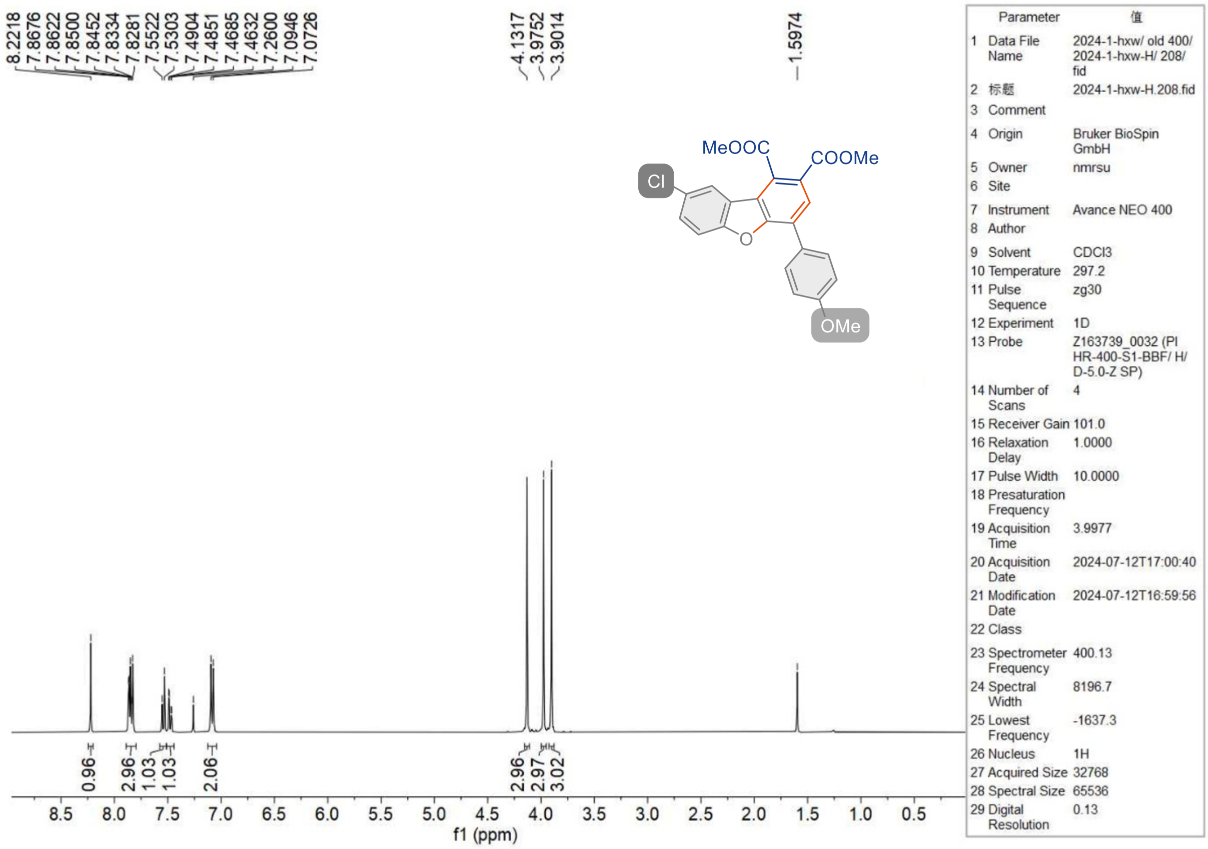

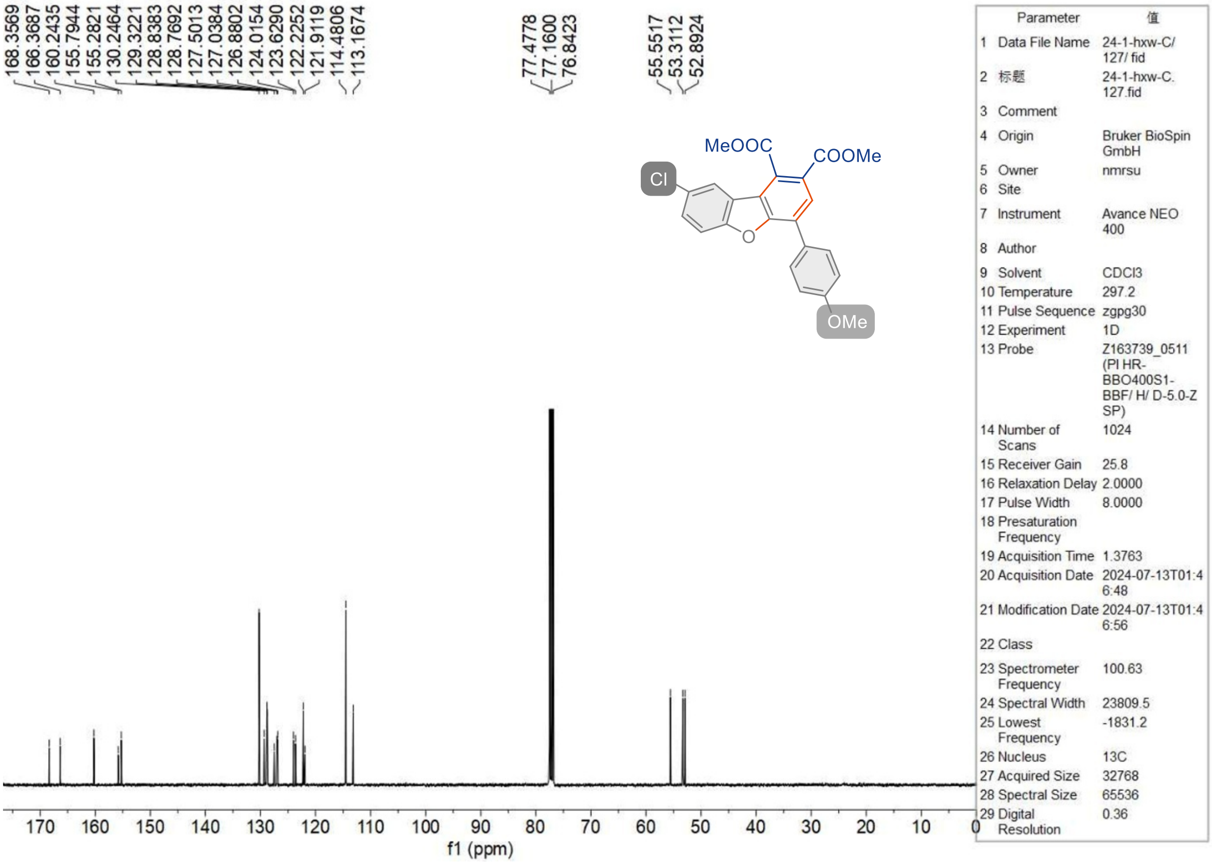

Dimethyl 4-(4-bromophenyl)-8-chlorodibenzo[*b*,*d*]furan-1,2-dicarboxylate (product 4y)

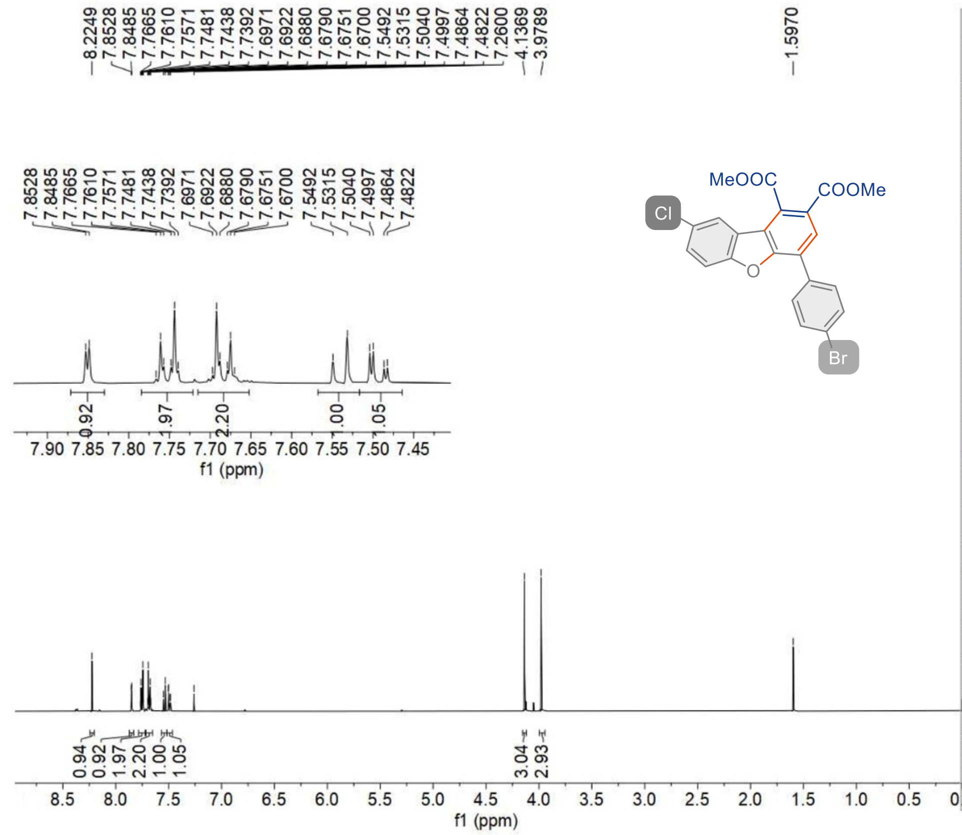

	Parameter	值
1	Data File Name	F:/ 500/ 2024-1-hxw-H/ 46/ fid
2	标题	2024-1-hxw-H.46.fid
3	Comment	1H yellow
4	Origin	Bruker BioSpin GmbH
5	Owner	root
6	Site	
7	Instrument	spect
8	Author	
9	Solvent	CDCl3
10	Temperature	296.1
11	Pulse Sequence	zg30
12	Experiment	1D
13	Probe	Z119470_0117 (PA BBO 500S1 BBF-H-D-05 Z SP)
14	Number of Scans	4
15	Receiver Gain	188.8
16	Relaxation Delay	2.0000
17	Pulse Width	12.0000
18	Presaturation Frequency	
19	Acquisition Time	2.0447
20	Acquisition Date	2024-09-05T17:30:57
21	Modification Date	2024-09-05T17:30:58
22	Class	
23	Spectrometer Frequency	500.16
24	Spectral Width	8012.8
25	Lowest Frequency	-1017.5
26	Nucleus	1H
27	Acquired Size	16384
28	Spectral Size	65536
29	Digital Resolution	0.12

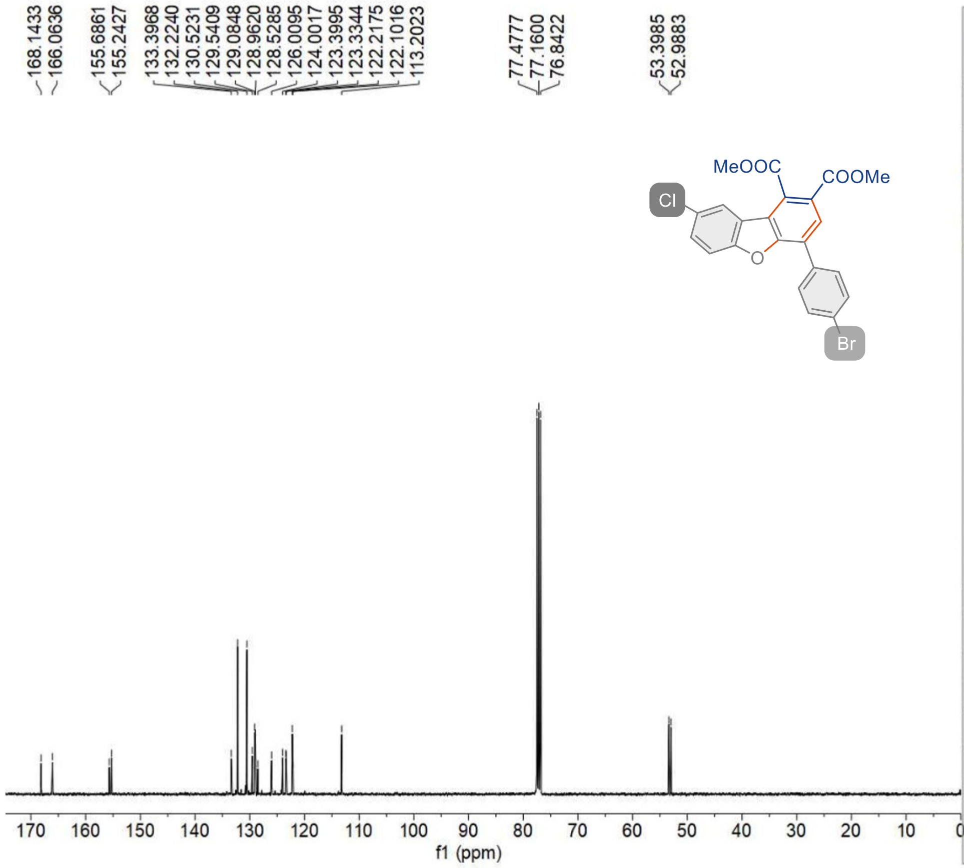

	Parameter	值
1	Data File Name	24-2-hxw-C/ 28/ fid
2	标题	24-2-hxw-C.28.fid
3	Comment	
4	Origin	Bruker BioSpin GmbH
5	Owner	nmrsu
6	Site	
7	Instrument	Avance NEO 400
8	Author	
9	Solvent	CDCl3
10	Temperature	295.6
11	Pulse Sequence	zgpg30
12	Experiment	1D
13	Probe	Z163739_0511 (PI HR-BBO400S1-BBF/ H/ D-5.0-Z SP)
14	Number of Scans	1200
15	Receiver Gain	25.1
16	Relaxation Delay	2.0000
17	Pulse Width	8.0000
18	Presaturation Frequency	
19	Acquisition Time	1.3763
20	Acquisition Date	2024-09-08T09:51:47
21	Modification Date	2024-09-08T09:51:50
22	Class	
23	Spectrometer Frequency	100.63
24	Spectral Width	23809.5
25	Lowest Frequency	-1831.9
26	Nucleus	13C
27	Acquired Size	32768
28	Spectral Size	65536
29	Digital Resolution	0.36

Dimethyl 8-bromo-4-(*p*-tolyl)dibenzo[*b*,*d*]furan-1,2-dicarboxylate (product 4z)

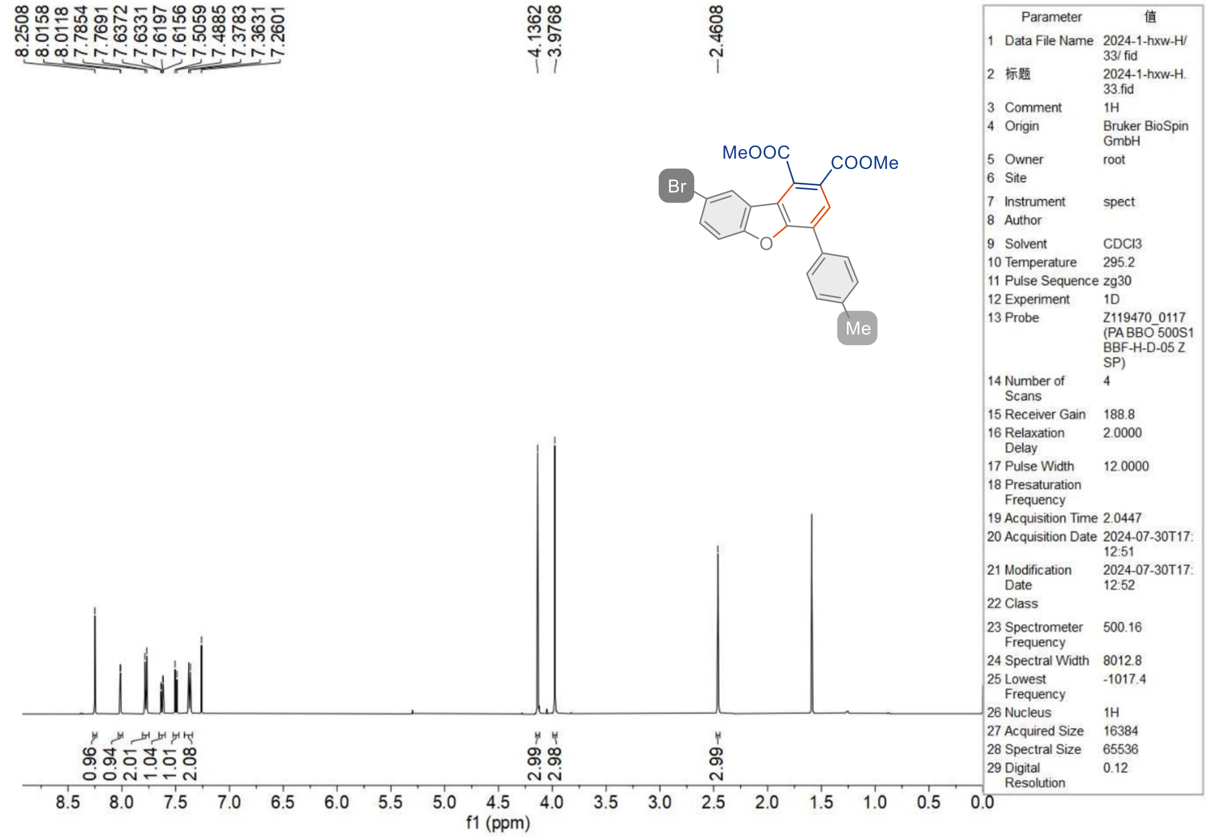

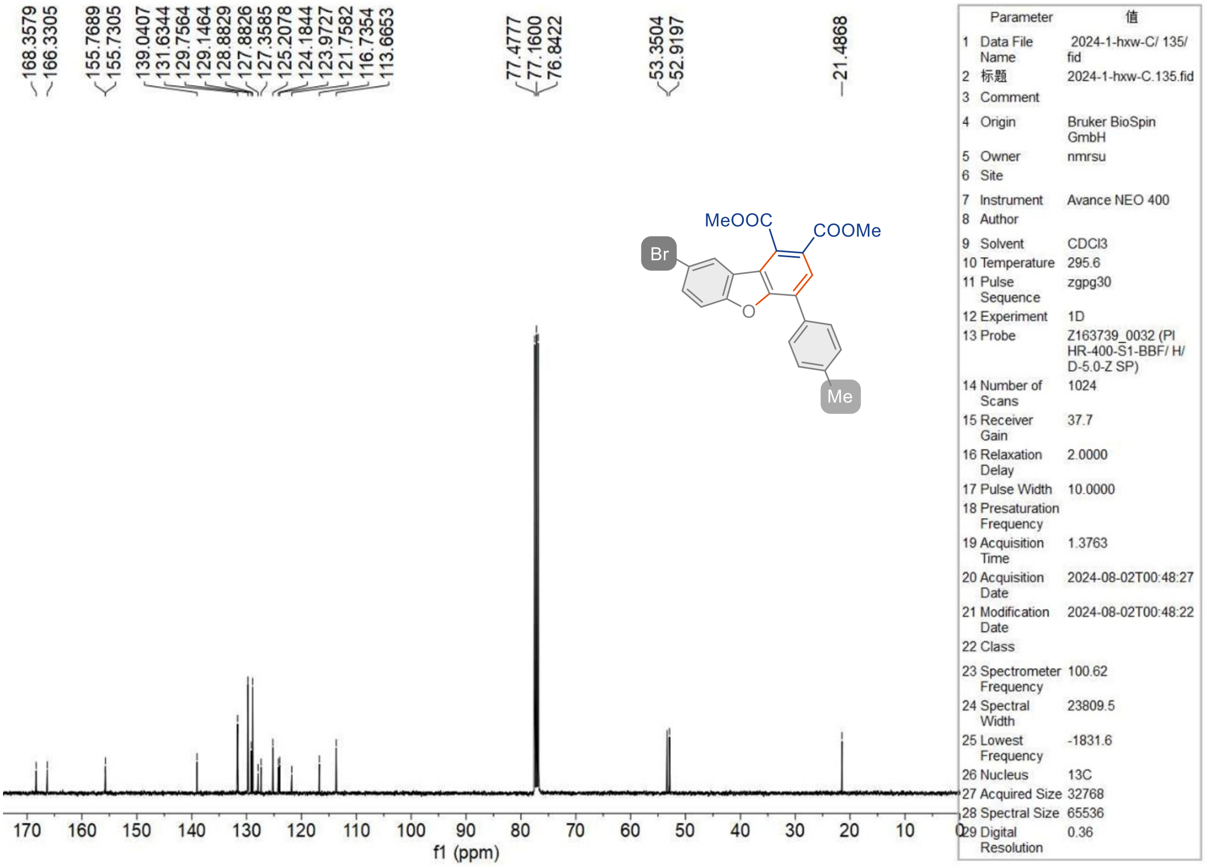

Dimethyl 8-bromo-4-(4-ethylphenyl)dibenzo[*b*,*d*]furan-1,2-dicarboxylate (product 4A)

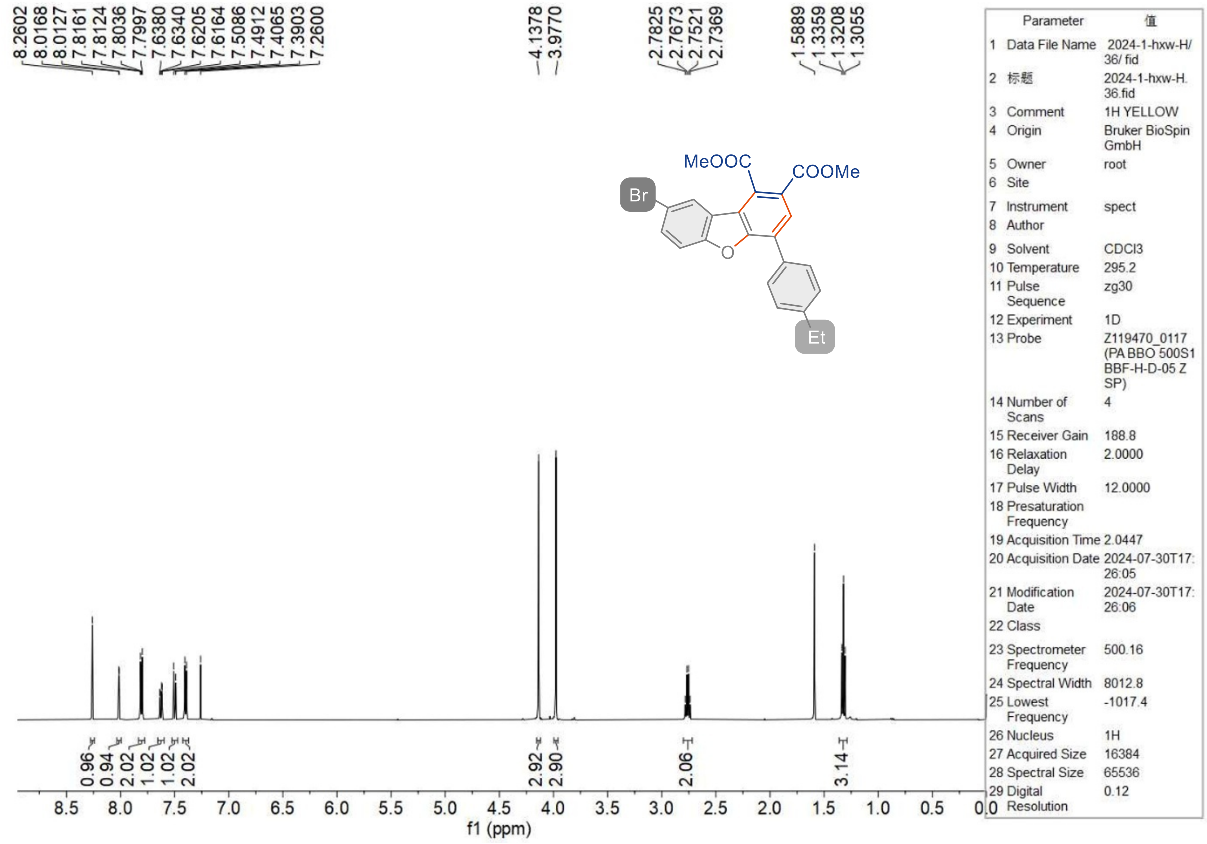

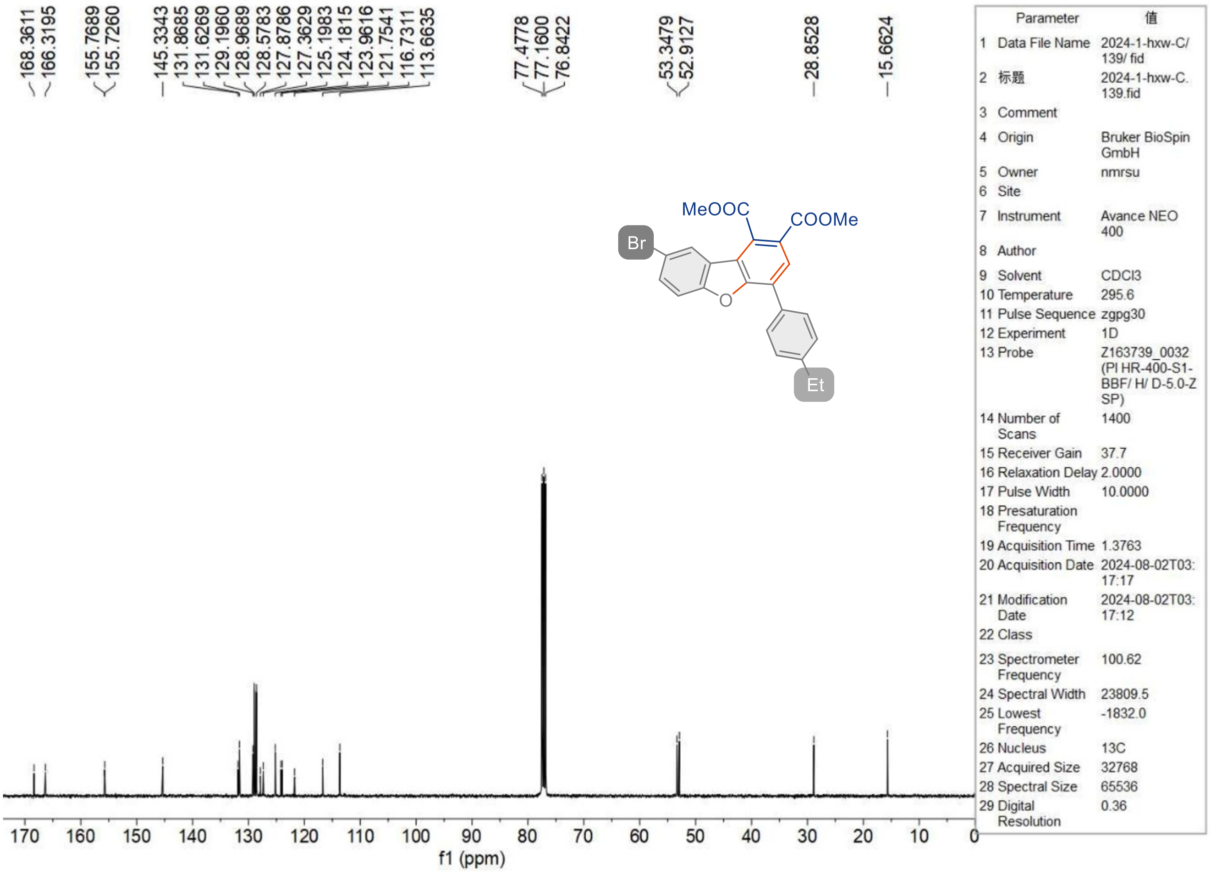

Dimethyl 8-bromo-4-(4-methoxyphenyl)dibenzo[*b,d*]furan-1,2-dicarboxylate (product 4B)

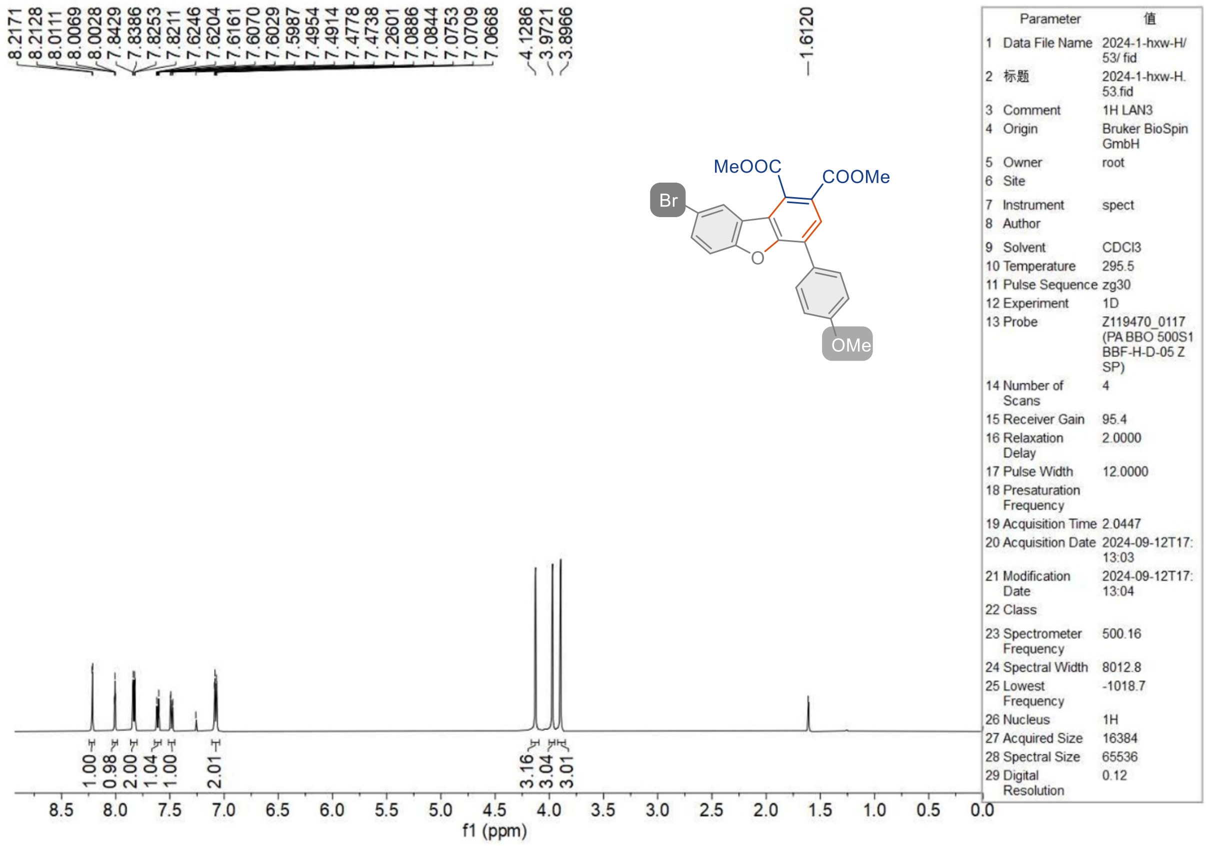

Parameter	值
1 Data File Name	2024-1-hxw-H/53/ fid
2 标题	2024-1-hxw-H.53.fid
3 Comment	1H LAN3
4 Origin	Bruker BioSpin GmbH
5 Owner	root
6 Site	
7 Instrument	spect
8 Author	
9 Solvent	CDCl3
10 Temperature	295.5
11 Pulse Sequence	zg30
12 Experiment	1D
13 Probe	Z119470_0117 (PA BBO 500S1 BBF-H-D-05 Z SP)
14 Number of Scans	4
15 Receiver Gain	95.4
16 Relaxation Delay	2.0000
17 Pulse Width	12.0000
18 Presaturation Frequency	
19 Acquisition Time	2.0447
20 Acquisition Date	2024-09-12T17:13:03
21 Modification Date	2024-09-12T17:13:04
22 Class	
23 Spectrometer Frequency	500.16
24 Spectral Width	8012.8
25 Lowest Frequency	-1018.7
26 Nucleus	1H
27 Acquired Size	16384
28 Spectral Size	65536
29 Digital Resolution	0.12

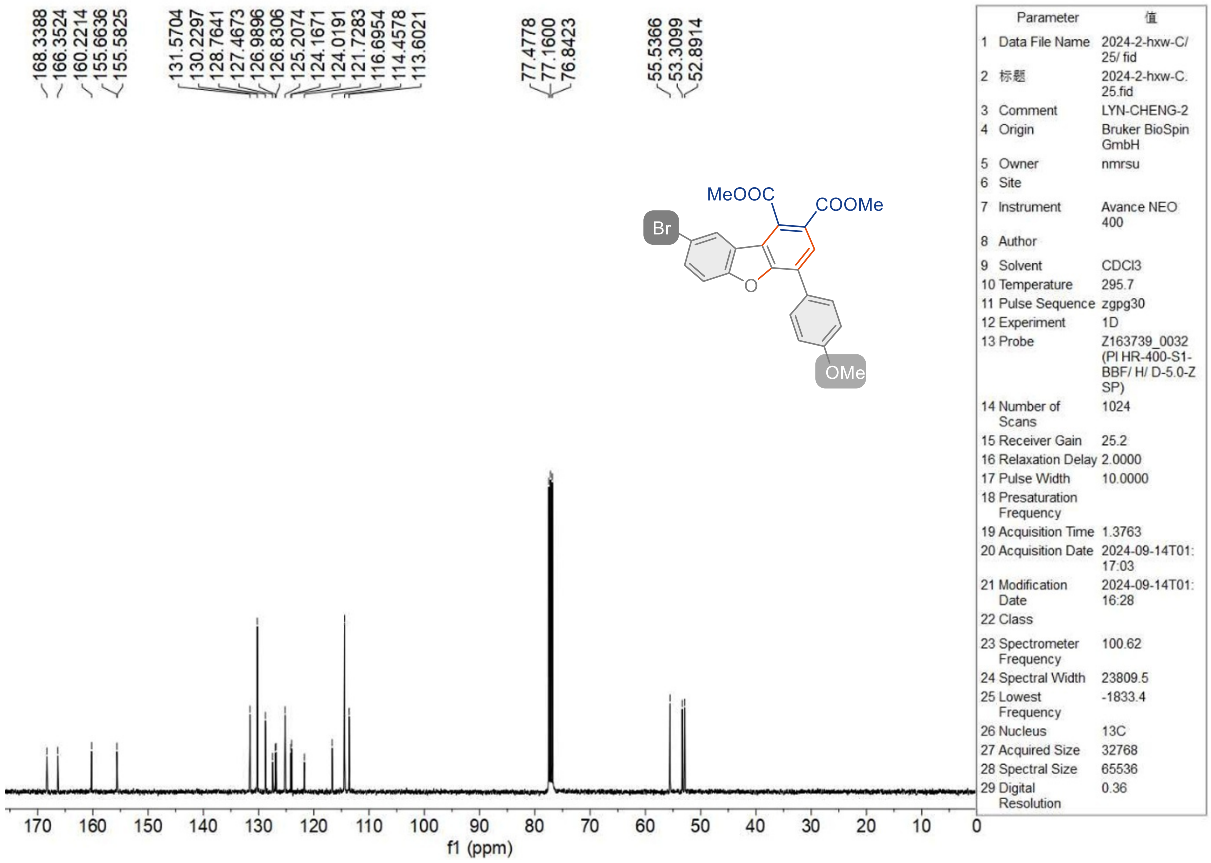

Parameter	值
1 Data File Name	2024-2-hxw-C/25/ fid
2 标题	2024-2-hxw-C.25.fid
3 Comment	LYN-CHENG-2
4 Origin	Bruker BioSpin GmbH
5 Owner	nmrsu
6 Site	
7 Instrument	Avance NEO 400
8 Author	
9 Solvent	CDCl3
10 Temperature	295.7
11 Pulse Sequence	zgpg30
12 Experiment	1D
13 Probe	Z163739_0032 (PI HR-400-S1-BBF/ H/ D-5.0-Z SP)
14 Number of Scans	1024
15 Receiver Gain	25.2
16 Relaxation Delay	2.0000
17 Pulse Width	10.0000
18 Presaturation Frequency	
19 Acquisition Time	1.3763
20 Acquisition Date	2024-09-14T01:17:03
21 Modification Date	2024-09-14T01:16:28
22 Class	
23 Spectrometer Frequency	100.62
24 Spectral Width	23809.5
25 Lowest Frequency	-1833.4
26 Nucleus	13C
27 Acquired Size	32768
28 Spectral Size	65536
29 Digital Resolution	0.36

Dimethyl 6-chloro-4-phenyldibenzo[*b,d*]furan-1,2-dicarboxylate (product 4C)

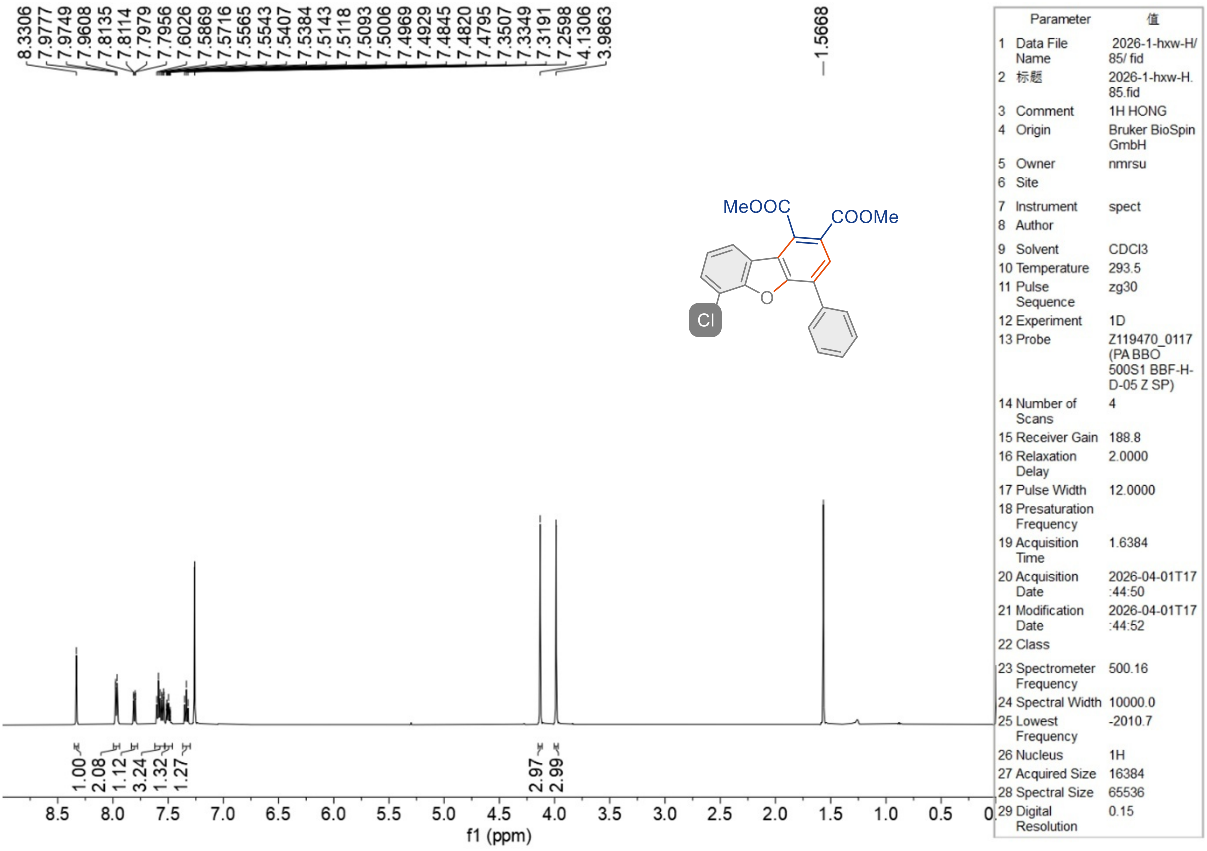

Parameter	值
1 Data File Name	2026-1-hxw-H/ 85/ fid
2 标题	2026-1-hxw-H. 85.fid
3 Comment	1H HONG
4 Origin	Bruker BioSpin GmbH
5 Owner	nmrsu
6 Site	
7 Instrument	spect
8 Author	
9 Solvent	CDCl3
10 Temperature	293.5
11 Pulse Sequence	zg30
12 Experiment	1D
13 Probe	Z119470_0117 (PA BBO 500S1 BBF-H-D-05 Z SP)
14 Number of Scans	4
15 Receiver Gain	188.8
16 Relaxation Delay	2.0000
17 Pulse Width	12.0000
18 Presaturation Frequency	
19 Acquisition Time	1.6384
20 Acquisition Date	2026-04-01T17:44:50
21 Modification Date	2026-04-01T17:44:52
22 Class	
23 Spectrometer Frequency	500.16
24 Spectral Width	10000.0
25 Lowest Frequency	-2010.7
26 Nucleus	1H
27 Acquired Size	16384
28 Spectral Size	65536
29 Digital Resolution	0.15

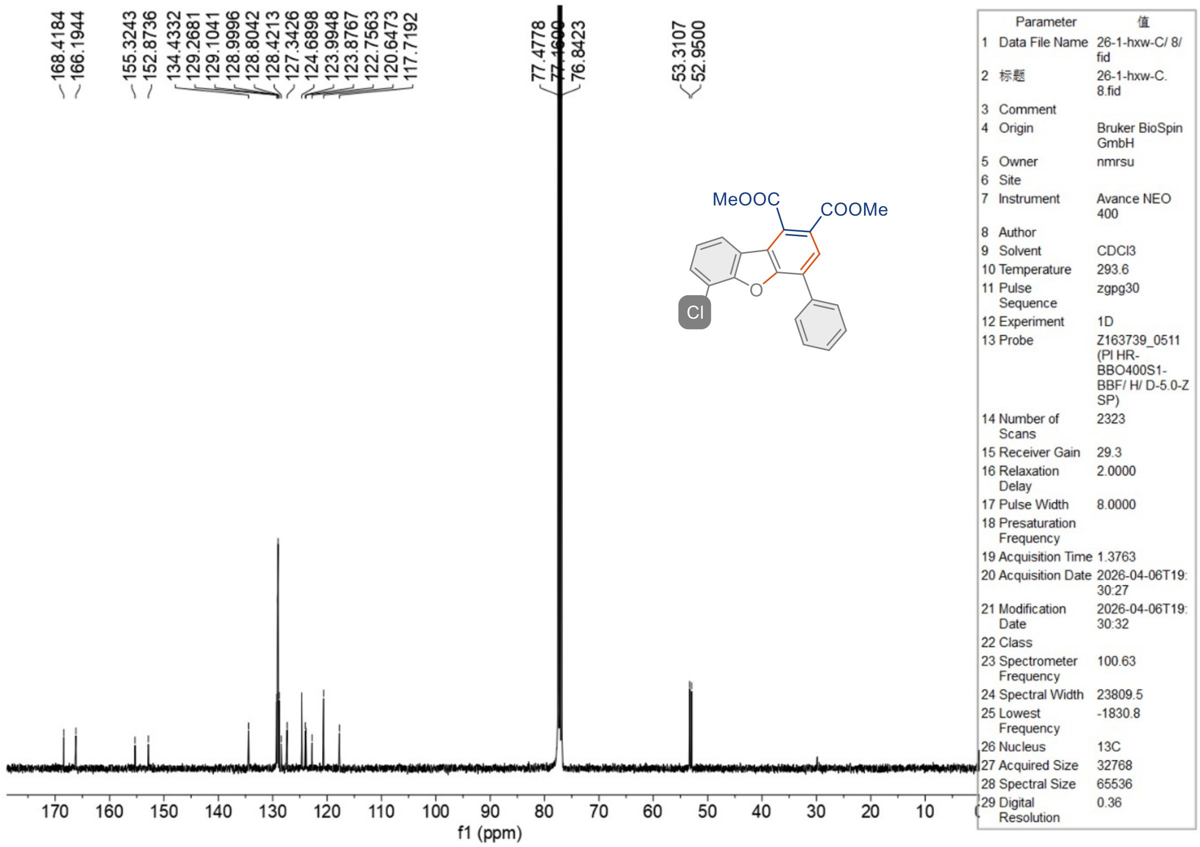

Parameter	值
1 Data File Name	26-1-hxw-C/ 8/ fid
2 标题	26-1-hxw-C. 8.fid
3 Comment	
4 Origin	Bruker BioSpin GmbH
5 Owner	nmrsu
6 Site	
7 Instrument	Avance NEO 400
8 Author	
9 Solvent	CDCl3
10 Temperature	293.6
11 Pulse Sequence	zgpg30
12 Experiment	1D
13 Probe	Z163739_0511 (PI HR-BBO400S1-BBF/ H/ D-5.0-Z SP)
14 Number of Scans	2323
15 Receiver Gain	29.3
16 Relaxation Delay	2.0000
17 Pulse Width	8.0000
18 Presaturation Frequency	
19 Acquisition Time	1.3763
20 Acquisition Date	2026-04-06T19:30:27
21 Modification Date	2026-04-06T19:30:32
22 Class	
23 Spectrometer Frequency	100.63
24 Spectral Width	23809.5
25 Lowest Frequency	-1830.8
26 Nucleus	13C
27 Acquired Size	32768
28 Spectral Size	65536
29 Digital Resolution	0.36

Dimethyl 7-chloro-4-phenyldibenzo[*b*,*d*]furan-1,2-dicarboxylate (product 4D)

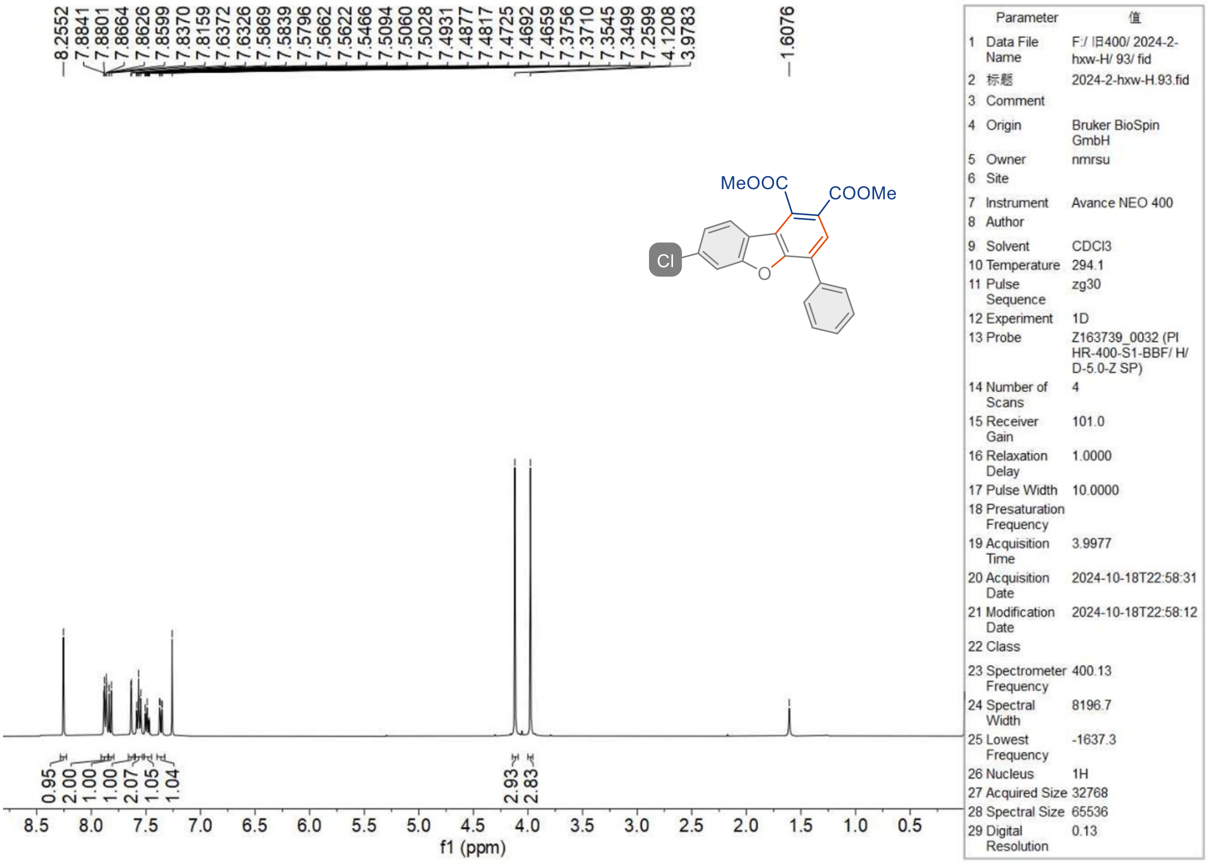

Parameter	值
1 Data File Name	F:/ 旧400/ 2024-2-hxw-H/ 93/ fid
2 标题	2024-2-hxw-H.93.fid
3 Comment	
4 Origin	Bruker BioSpin GmbH
5 Owner	nmrsu
6 Site	
7 Instrument	Avance NEO 400
8 Author	
9 Solvent	CDCl3
10 Temperature	294.1
11 Pulse Sequence	zg30
12 Experiment	1D
13 Probe	Z163739_0032 (PI HR-400-S1-BBF/ H/ D-5.0-Z SP)
14 Number of Scans	4
15 Receiver Gain	101.0
16 Relaxation Delay	1.0000
17 Pulse Width	10.0000
18 Presaturation Frequency	
19 Acquisition Time	3.9977
20 Acquisition Date	2024-10-18T22:58:31
21 Modification Date	2024-10-18T22:58:12
22 Class	
23 Spectrometer Frequency	400.13
24 Spectral Width	8196.7
25 Lowest Frequency	-1637.3
26 Nucleus	1H
27 Acquired Size	32768
28 Spectral Size	65536
29 Digital Resolution	0.13

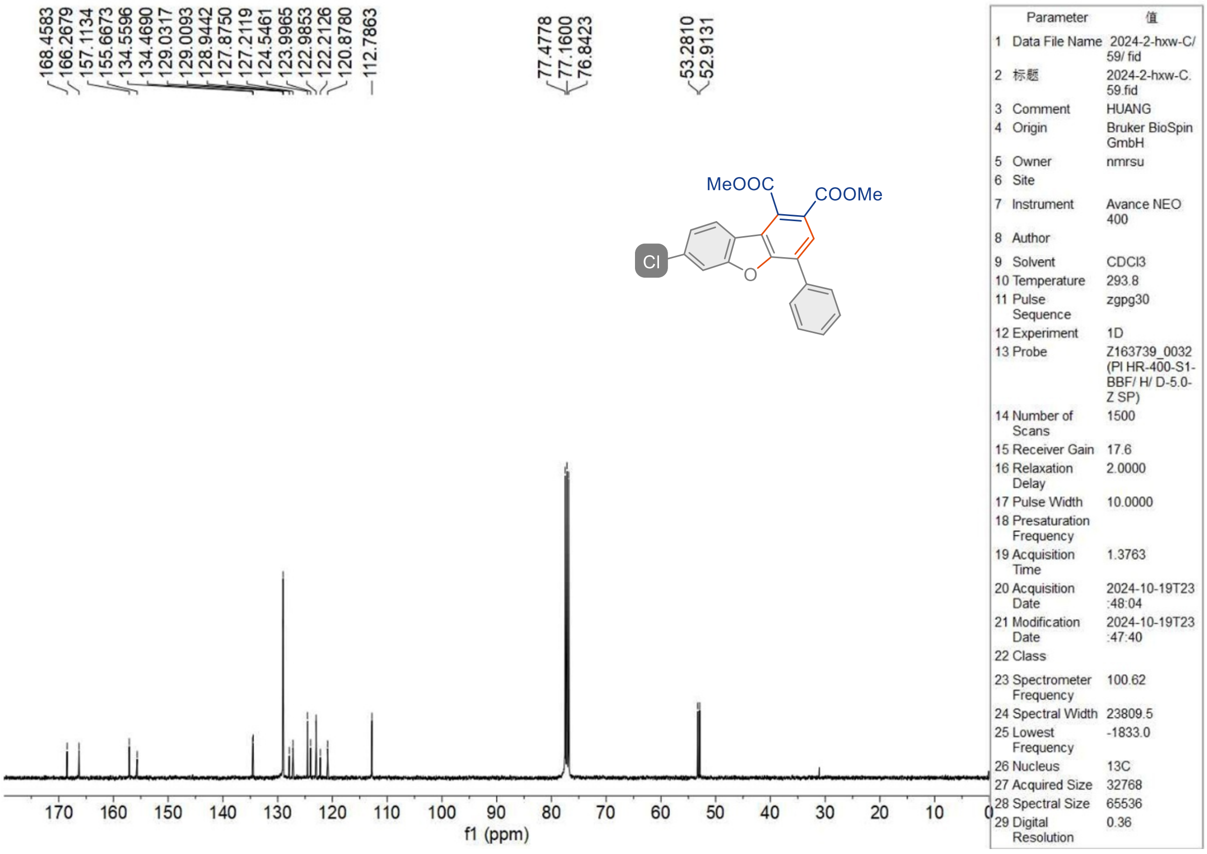

Parameter	值
1 Data File Name	2024-2-hxw-C/ 59/ fid
2 标题	2024-2-hxw-C.59.fid
3 Comment	HUANG
4 Origin	Bruker BioSpin GmbH
5 Owner	nmrsu
6 Site	
7 Instrument	Avance NEO 400
8 Author	
9 Solvent	CDCl3
10 Temperature	293.8
11 Pulse Sequence	zgpg30
12 Experiment	1D
13 Probe	Z163739_0032 (PI HR-400-S1-BBF/ H/ D-5.0-Z SP)
14 Number of Scans	1500
15 Receiver Gain	17.6
16 Relaxation Delay	2.0000
17 Pulse Width	10.0000
18 Presaturation Frequency	
19 Acquisition Time	1.3763
20 Acquisition Date	2024-10-19T23:48:04
21 Modification Date	2024-10-19T23:47:40
22 Class	
23 Spectrometer Frequency	100.62
24 Spectral Width	23809.5
25 Lowest Frequency	-1833.0
26 Nucleus	13C
27 Acquired Size	32768
28 Spectral Size	65536
29 Digital Resolution	0.36

Dimethyl 7-chloro-4-(*p*-tolyl)dibenzo[*b*,*d*]furan-1,2-dicarboxylate (product 4E)

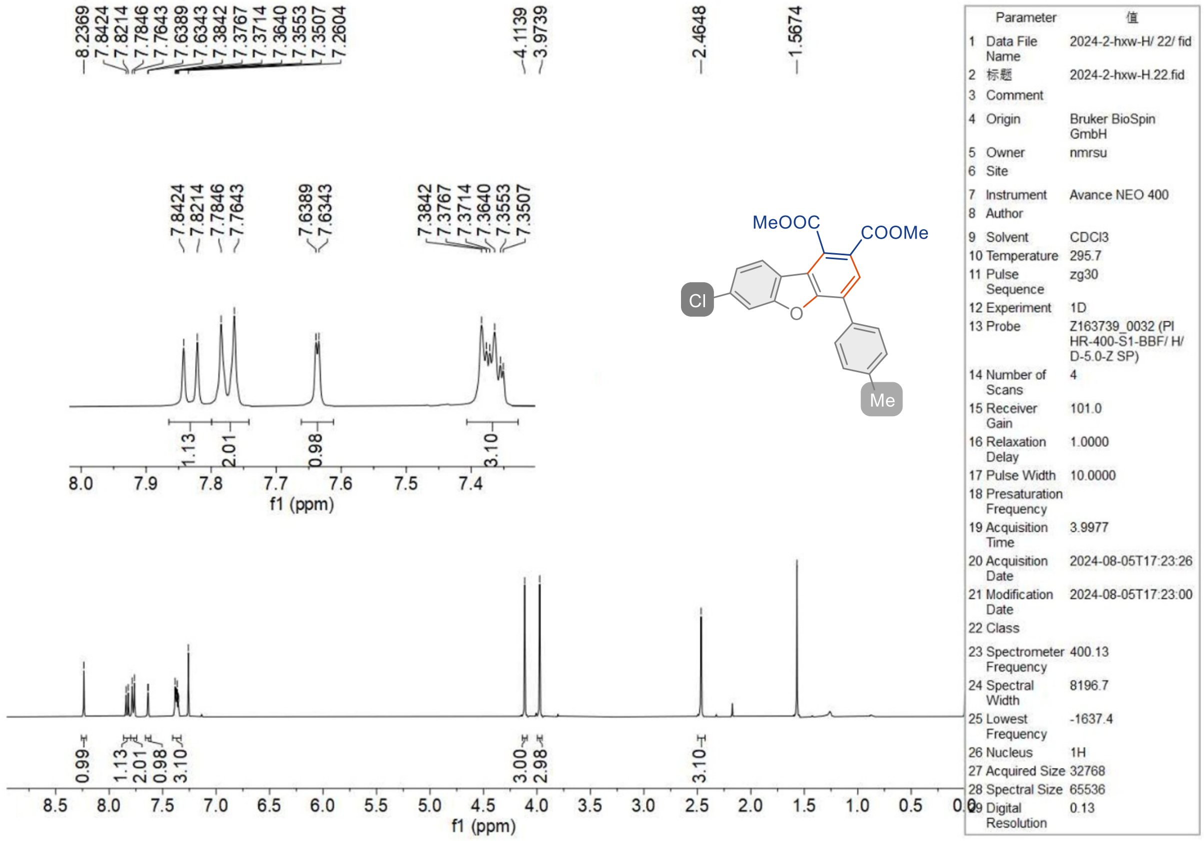

Parameter	值
1 Data File Name	2024-2-hxw-H/ 22/ fid
2 标题	2024-2-hxw-H.22.fid
3 Comment	
4 Origin	Bruker BioSpin GmbH
5 Owner	nmrsu
6 Site	
7 Instrument	Avance NEO 400
8 Author	
9 Solvent	CDCl3
10 Temperature	295.7
11 Pulse Sequence	zg30
12 Experiment	1D
13 Probe	Z163739_0032 (PI HR-400-S1-BBF/ H/ D-5.0-Z SP)
14 Number of Scans	4
15 Receiver Gain	101.0
16 Relaxation Delay	1.0000
17 Pulse Width	10.0000
18 Presaturation Frequency	
19 Acquisition Time	3.9977
20 Acquisition Date	2024-08-05T17:23:26
21 Modification Date	2024-08-05T17:23:00
22 Class	
23 Spectrometer Frequency	400.13
24 Spectral Width	8196.7
25 Lowest Frequency	-1637.4
26 Nucleus	1H
27 Acquired Size	32768
28 Spectral Size	65536
29 Digital Resolution	0.13

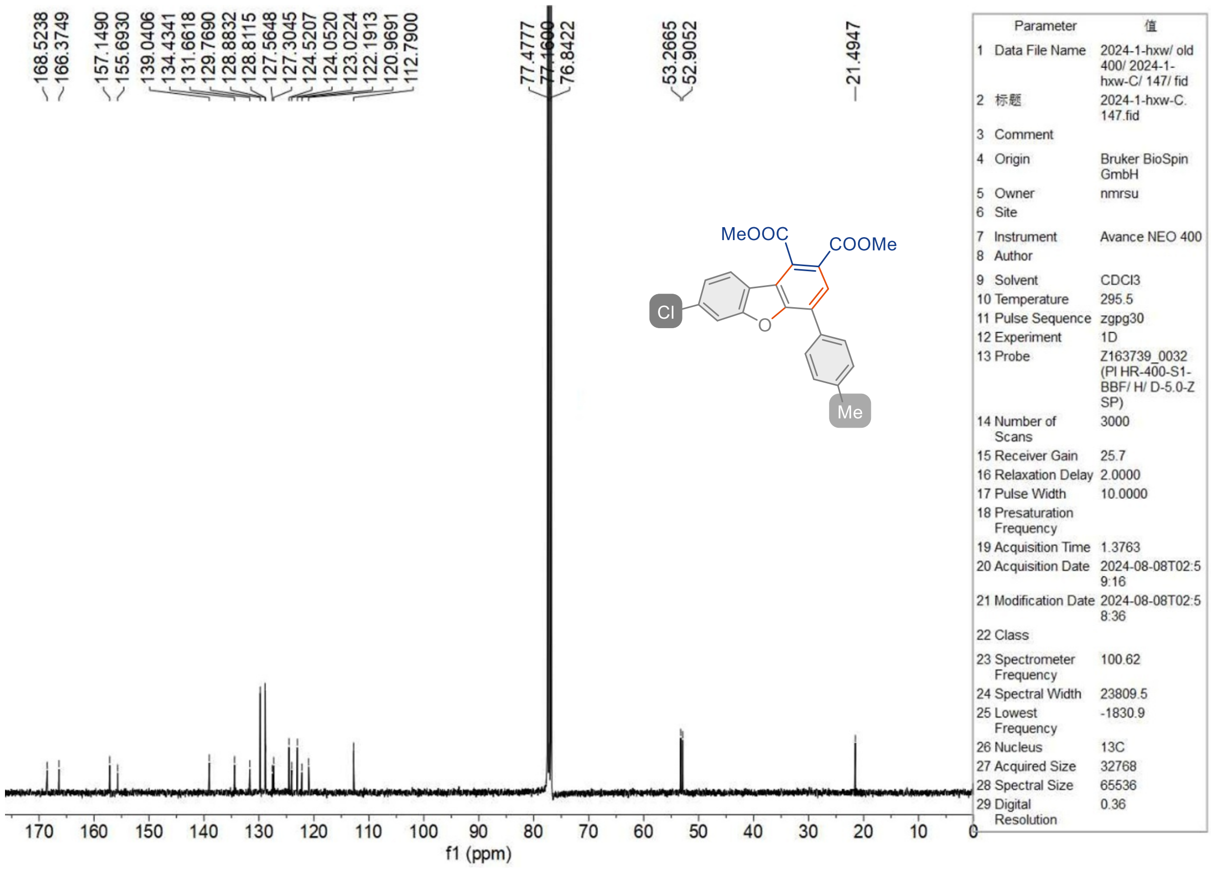

Parameter	值
1 Data File Name	2024-1-hxw/ old 400/ 2024-1-hxw-C/ 147/ fid
2 标题	2024-1-hxw-C.147.fid
3 Comment	
4 Origin	Bruker BioSpin GmbH
5 Owner	nmrsu
6 Site	
7 Instrument	Avance NEO 400
8 Author	
9 Solvent	CDCl3
10 Temperature	295.5
11 Pulse Sequence	zgpg30
12 Experiment	1D
13 Probe	Z163739_0032 (PI HR-400-S1-BBF/ H/ D-5.0-Z SP)
14 Number of Scans	3000
15 Receiver Gain	25.7
16 Relaxation Delay	2.0000
17 Pulse Width	10.0000
18 Presaturation Frequency	
19 Acquisition Time	1.3763
20 Acquisition Date	2024-08-08T02:59:16
21 Modification Date	2024-08-08T02:58:36
22 Class	
23 Spectrometer Frequency	100.62
24 Spectral Width	23809.5
25 Lowest Frequency	-1830.9
26 Nucleus	13C
27 Acquired Size	32768
28 Spectral Size	65536
29 Digital Resolution	0.36

Dimethyl 4-(thiophen-2-yl)dibenzo[*b,d*]furan-1,2-dicarboxylate (product 4F)

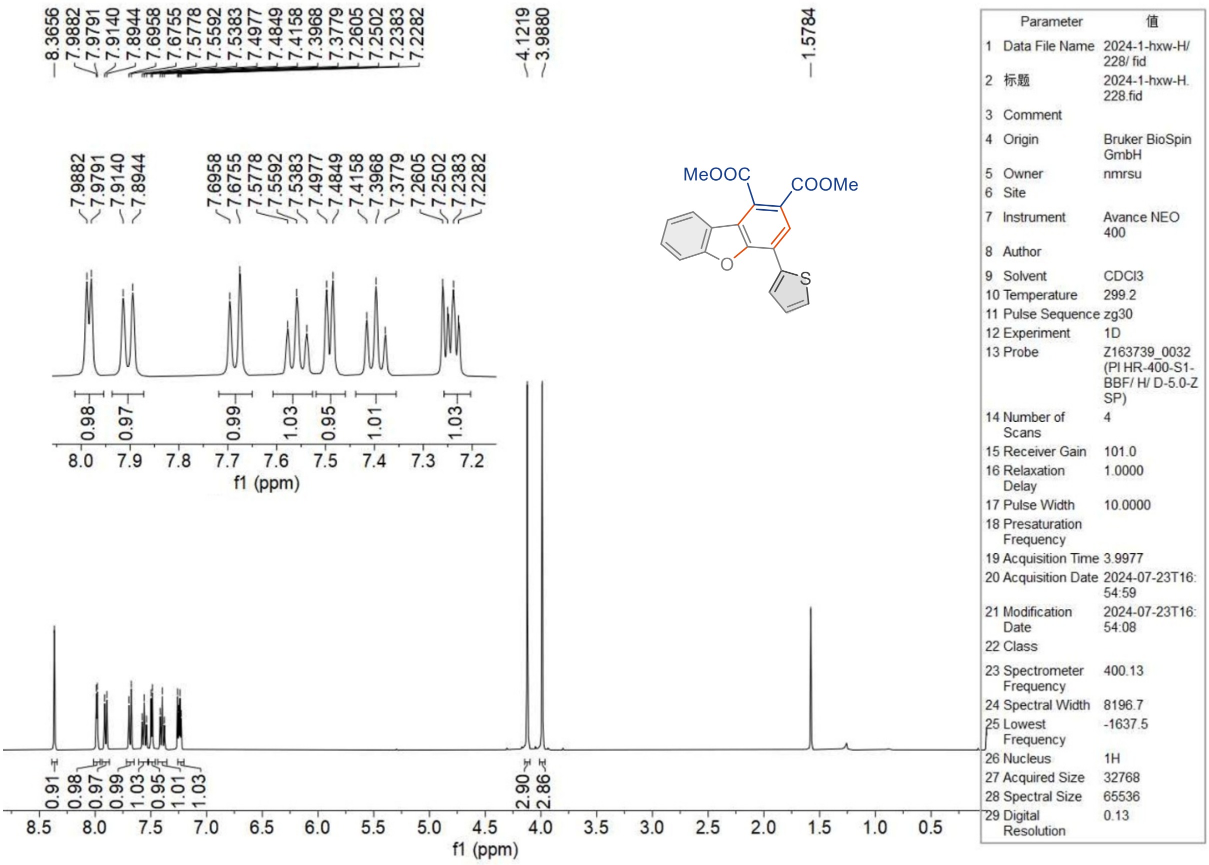

Parameter	值
1 Data File Name	2024-1-hxw-H/228/ fid
2 标题	2024-1-hxw-H.228.fid
3 Comment	
4 Origin	Bruker BioSpin GmbH
5 Owner	nmrsu
6 Site	
7 Instrument	Avance NEO 400
8 Author	
9 Solvent	CDCl3
10 Temperature	299.2
11 Pulse Sequence	zg30
12 Experiment	1D
13 Probe	Z163739_0032 (PI HR-400-S1-BBF/ H/ D-5.0-Z SP)
14 Number of Scans	4
15 Receiver Gain	101.0
16 Relaxation Delay	1.0000
17 Pulse Width	10.0000
18 Presaturation Frequency	
19 Acquisition Time	3.9977
20 Acquisition Date	2024-07-23T16:54:59
21 Modification Date	2024-07-23T16:54:08
22 Class	
23 Spectrometer Frequency	400.13
24 Spectral Width	8196.7
25 Lowest Frequency	-1637.5
26 Nucleus	1H
27 Acquired Size	32768
28 Spectral Size	65536
29 Digital Resolution	0.13

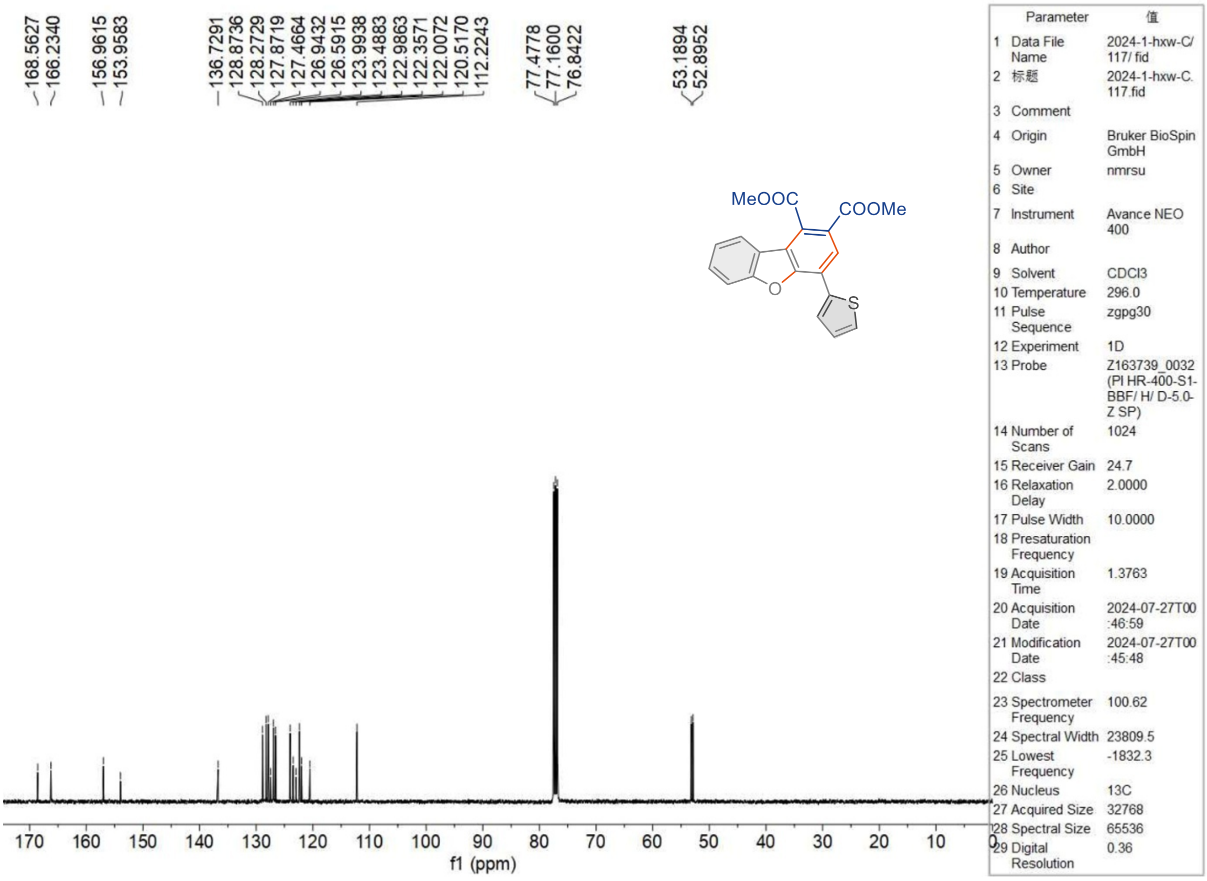

Parameter	值
1 Data File Name	2024-1-hxw-C/117/ fid
2 标题	2024-1-hxw-C.117.fid
3 Comment	
4 Origin	Bruker BioSpin GmbH
5 Owner	nmrsu
6 Site	
7 Instrument	Avance NEO 400
8 Author	
9 Solvent	CDCl3
10 Temperature	296.0
11 Pulse Sequence	zgpg30
12 Experiment	1D
13 Probe	Z163739_0032 (PI HR-400-S1-BBF/ H/ D-5.0-Z SP)
14 Number of Scans	1024
15 Receiver Gain	24.7
16 Relaxation Delay	2.0000
17 Pulse Width	10.0000
18 Presaturation Frequency	
19 Acquisition Time	1.3763
20 Acquisition Date	2024-07-27T00:46:59
21 Modification Date	2024-07-27T00:45:48
22 Class	
23 Spectrometer Frequency	100.62
24 Spectral Width	23809.5
25 Lowest Frequency	-1832.3
26 Nucleus	13C
27 Acquired Size	32768
28 Spectral Size	65536
29 Digital Resolution	0.36

Dimethyl 4-(4-(((5-isopropyl-2-methylcyclohexyl)oxy)carbonyl) phenyl)dibenzo[*b,d*]furan-1,2-dicarboxylate (product 4G)

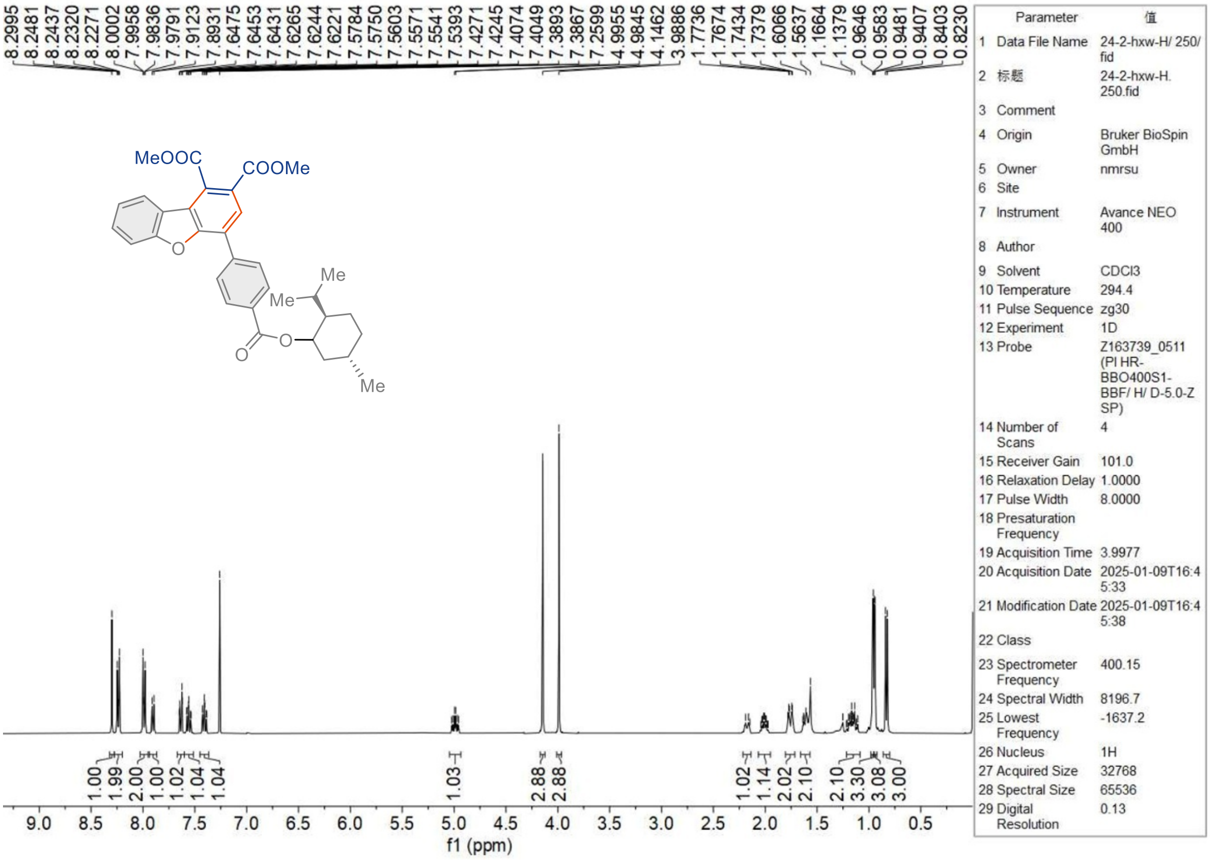

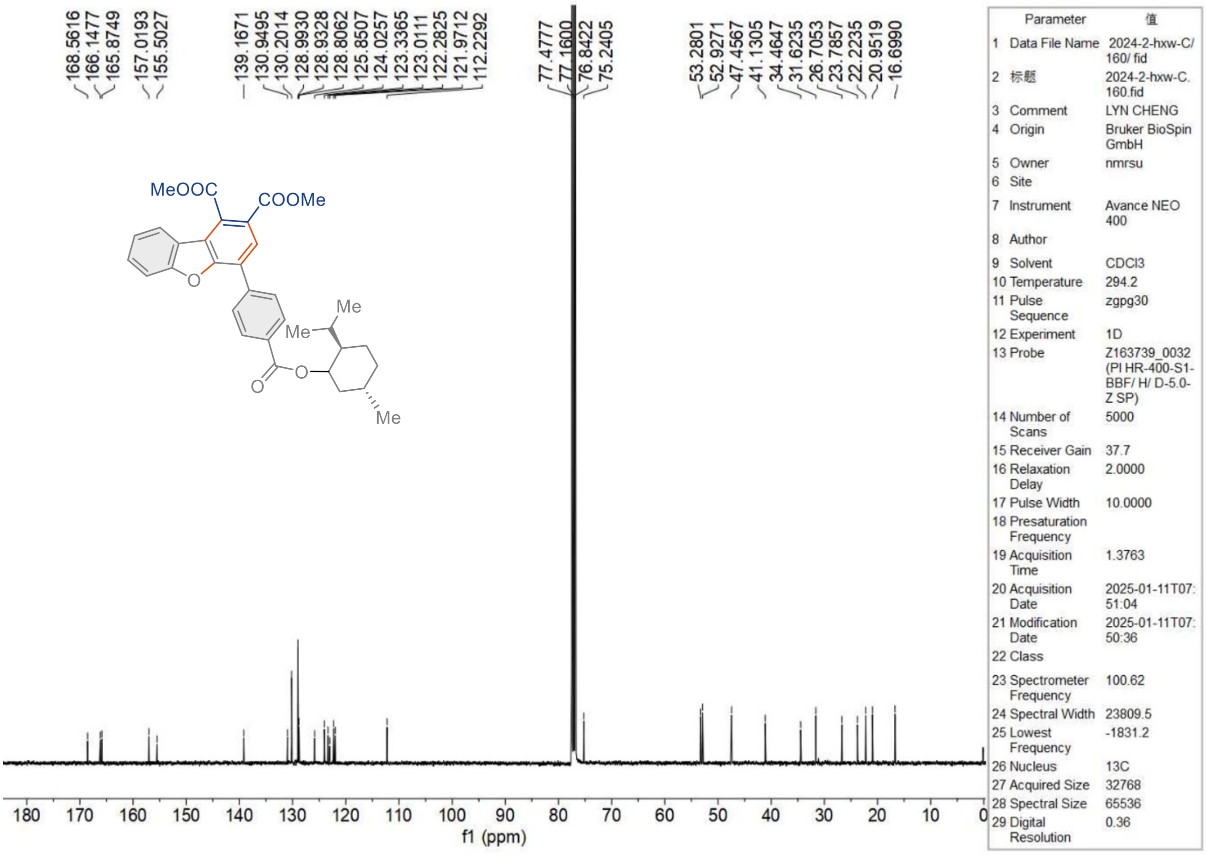

Dimethyl 4-(4-((2-(naphthalen-1-yl)ethoxy) carbonyl)phenyl)dibenzo [*b,d*] furan-1,2-dicarboxylate (product 4H)

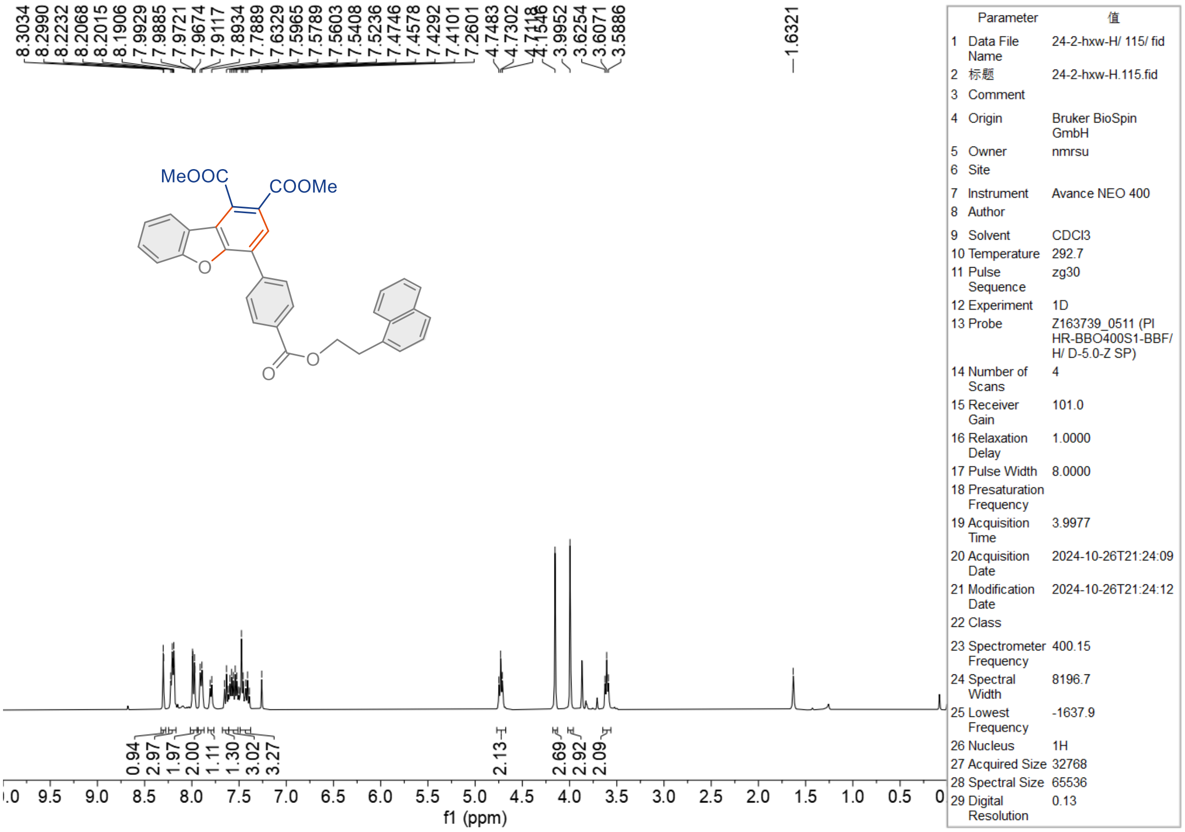

Parameter	值
1 Data File Name	24-2-hxw-H/ 115/ fid
2 标题	24-2-hxw-H.115.fid
3 Comment	
4 Origin	Bruker BioSpin GmbH
5 Owner	nmrsu
6 Site	
7 Instrument	Avance NEO 400
8 Author	
9 Solvent	CDCl3
10 Temperature	292.7
11 Pulse Sequence	zg30
12 Experiment	1D
13 Probe	Z163739_0511 (PI HR-BBO400S1-BBF/ H/ D-5.0-Z SP)
14 Number of Scans	4
15 Receiver Gain	101.0
16 Relaxation Delay	1.0000
17 Pulse Width	8.0000
18 Presaturation Frequency	
19 Acquisition Time	3.9977
20 Acquisition Date	2024-10-26T21:24:09
21 Modification Date	2024-10-26T21:24:12
22 Class	
23 Spectrometer Frequency	400.15
24 Spectral Width	8196.7
25 Lowest Frequency	-1637.9
26 Nucleus	1H
27 Acquired Size	32768
28 Spectral Size	65536
29 Digital Resolution	0.13

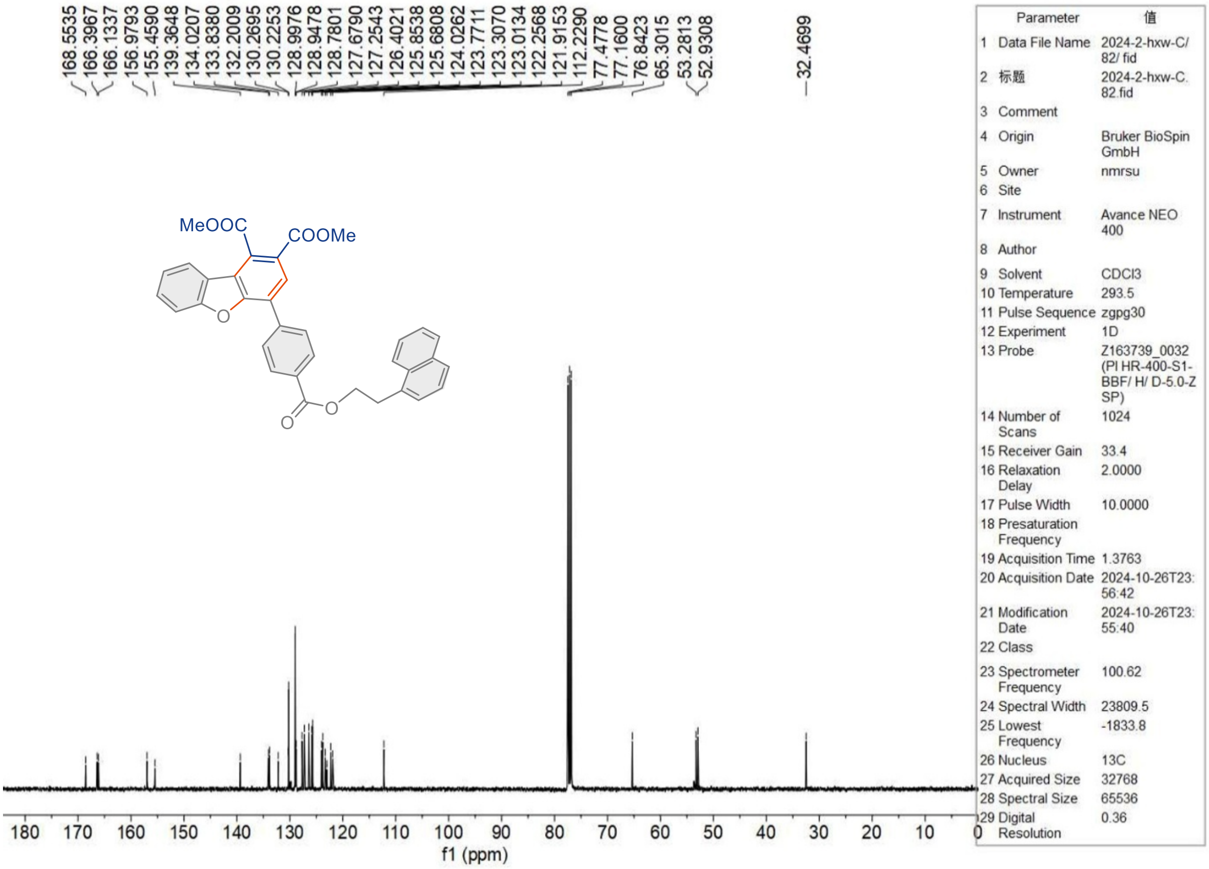

Parameter	值
1 Data File Name	2024-2-hxw-C/ 82/ fid
2 标题	2024-2-hxw-C. 82.fid
3 Comment	
4 Origin	Bruker BioSpin GmbH
5 Owner	nmrsu
6 Site	
7 Instrument	Avance NEO 400
8 Author	
9 Solvent	CDCl3
10 Temperature	293.5
11 Pulse Sequence	zgpg30
12 Experiment	1D
13 Probe	Z163739_0032 (PI HR-400-S1-BBF/ H/ D-5.0-Z SP)
14 Number of Scans	1024
15 Receiver Gain	33.4
16 Relaxation Delay	2.0000
17 Pulse Width	10.0000
18 Presaturation Frequency	
19 Acquisition Time	1.3763
20 Acquisition Date	2024-10-26T23:56:42
21 Modification Date	2024-10-26T23:55:40
22 Class	
23 Spectrometer Frequency	100.62
24 Spectral Width	23809.5
25 Lowest Frequency	-1833.8
26 Nucleus	13C
27 Acquired Size	32768
28 Spectral Size	65536
29 Digital Resolution	0.36

Dimethyl 4-(4-((((3a*S*,5*S*,6*R*)-5-((*S*)-2,2-dimethyl-1,3-dioxolan-4-yl)-2,2-dimethyltetrahydrofuro[2,3-*d*][1,3]dioxol-6-yl) oxy)carbonyl)phenyl)dibenzo[*b*,*d*]furan-1,2-dicarboxylate (product 4I)

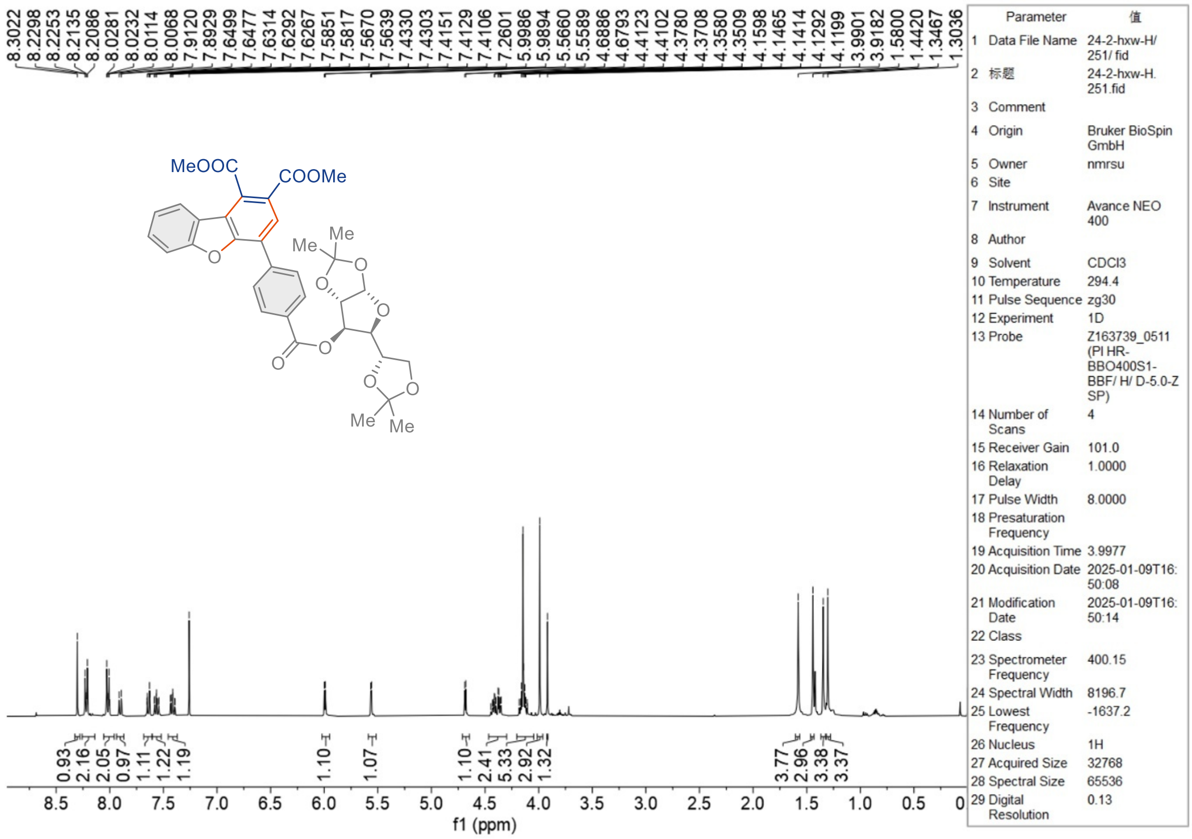

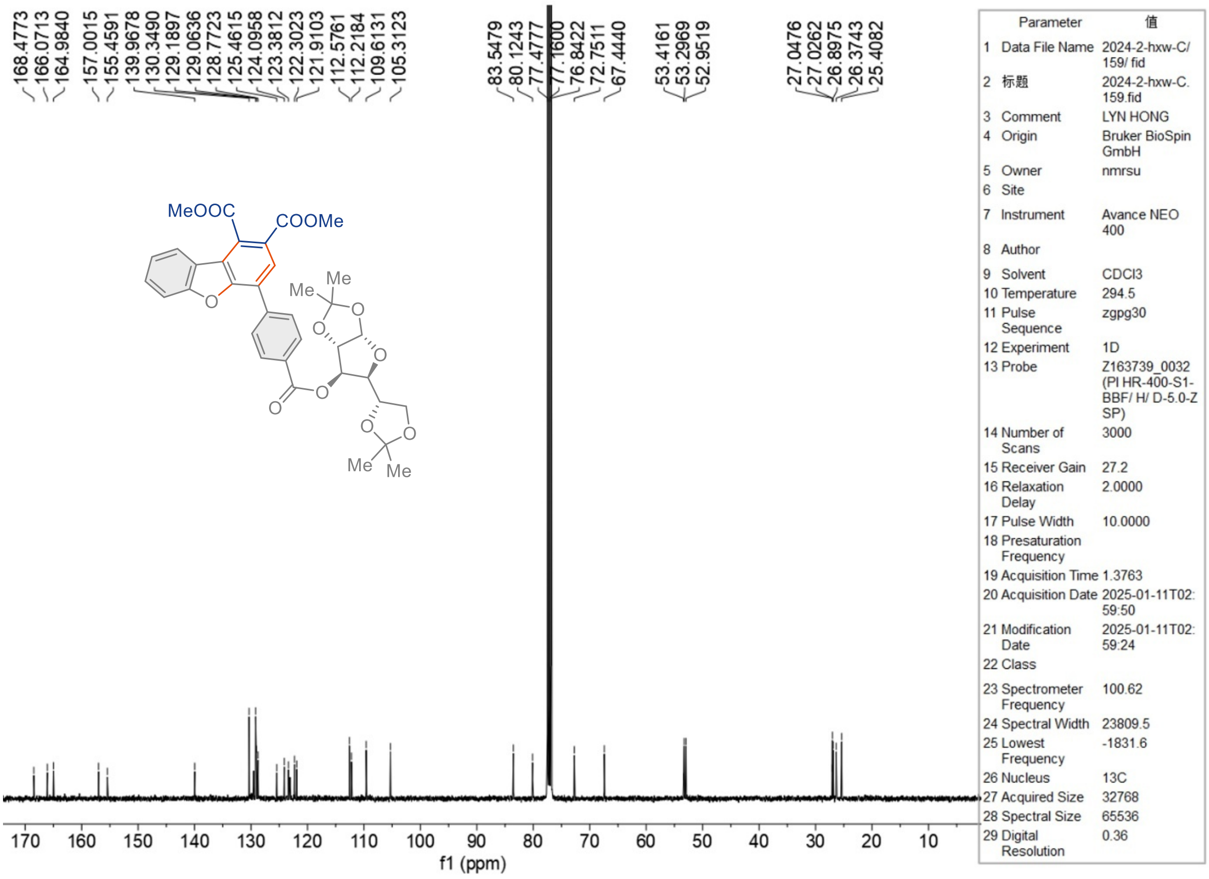

Diethyl 4-phenyldibenzo[*b*,*d*]furan-1,2-dicarboxylate (product 4J)

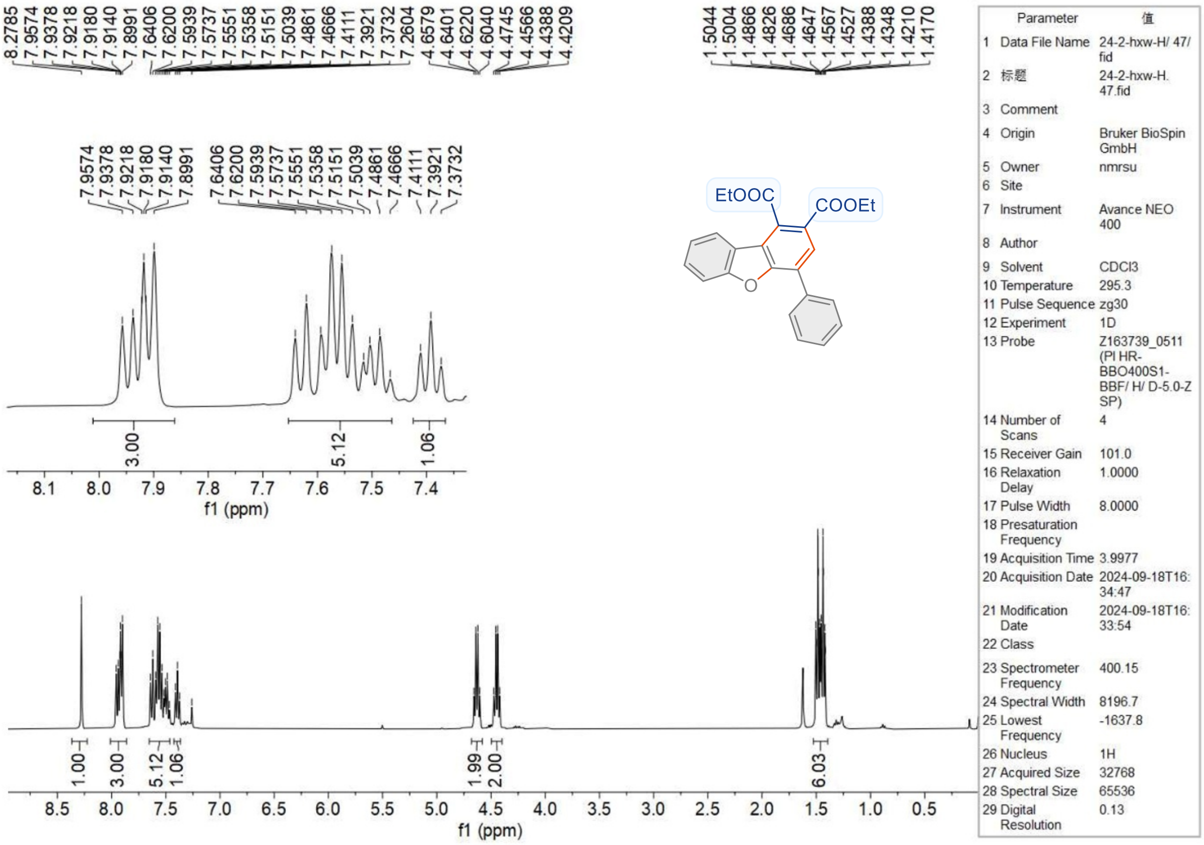

Parameter	值
1 Data File Name	24-2-hxw-H/ 47/ fid
2 标题	24-2-hxw-H. 47.fid
3 Comment	
4 Origin	Bruker BioSpin GmbH
5 Owner	nmrsu
6 Site	
7 Instrument	Avance NEO 400
8 Author	
9 Solvent	CDCl3
10 Temperature	295.3
11 Pulse Sequence	zg30
12 Experiment	1D
13 Probe	Z163739_0511 (PI HR-BBO400S1-BBF/ H/ D-5.0-Z SP)
14 Number of Scans	4
15 Receiver Gain	101.0
16 Relaxation Delay	1.0000
17 Pulse Width	8.0000
18 Presaturation Frequency	
19 Acquisition Time	3.9977
20 Acquisition Date	2024-09-18T16:34:47
21 Modification Date	2024-09-18T16:33:54
22 Class	
23 Spectrometer Frequency	400.15
24 Spectral Width	8196.7
25 Lowest Frequency	-1637.8
26 Nucleus	1H
27 Acquired Size	32768
28 Spectral Size	65536
29 Digital Resolution	0.13

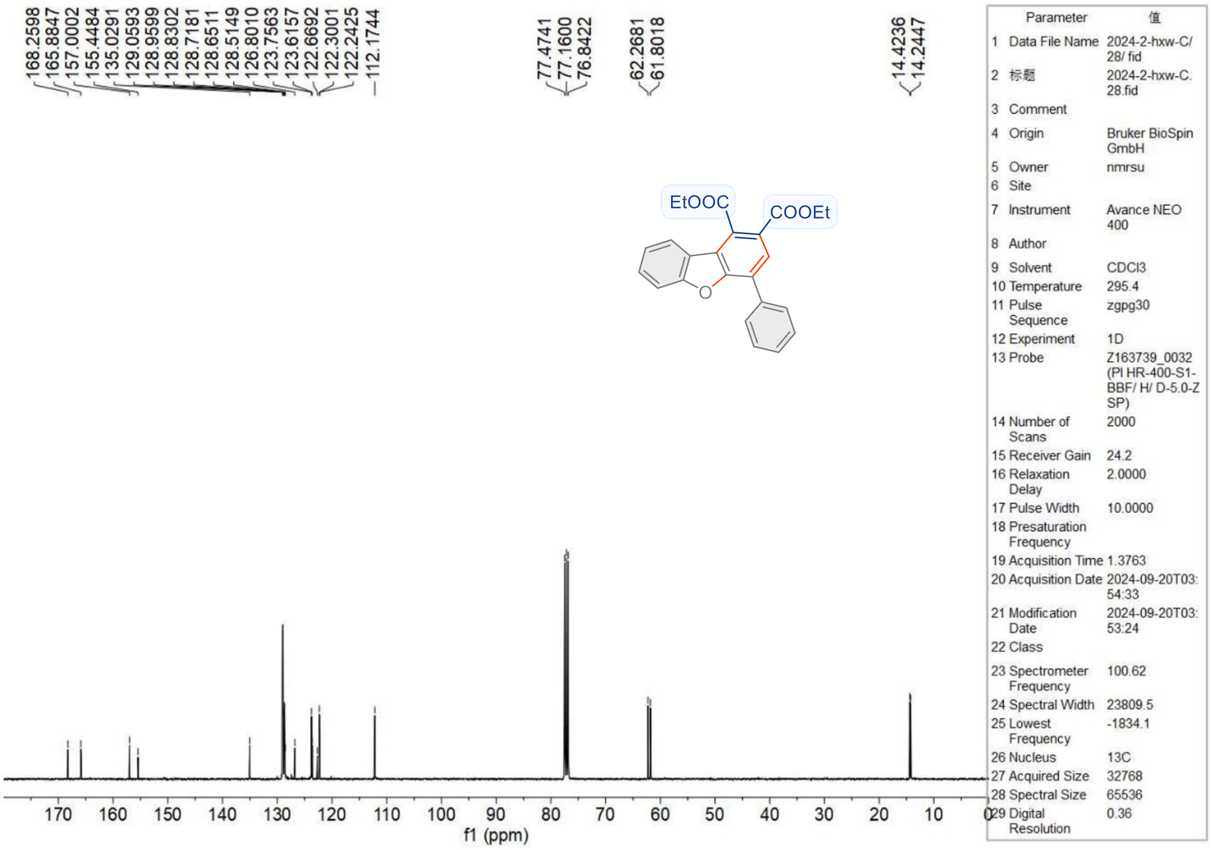

Parameter	值
1 Data File Name	2024-2-hxw-C/ 28/ fid
2 标题	2024-2-hxw-C. 28.fid
3 Comment	
4 Origin	Bruker BioSpin GmbH
5 Owner	nmrsu
6 Site	
7 Instrument	Avance NEO 400
8 Author	
9 Solvent	CDCl3
10 Temperature	295.4
11 Pulse Sequence	zgpg30
12 Experiment	1D
13 Probe	Z163739_0032 (PI HR-400-S1-BBF/ H/ D-5.0-Z SP)
14 Number of Scans	2000
15 Receiver Gain	24.2
16 Relaxation Delay	2.0000
17 Pulse Width	10.0000
18 Presaturation Frequency	
19 Acquisition Time	1.3763
20 Acquisition Date	2024-09-20T03:54:33
21 Modification Date	2024-09-20T03:53:24
22 Class	
23 Spectrometer Frequency	100.62
24 Spectral Width	23809.5
25 Lowest Frequency	-1834.1
26 Nucleus	13C
27 Acquired Size	32768
28 Spectral Size	65536
29 Digital Resolution	0.36

Dimethyl 3-methyl-4-phenyldibenzo[*b,d*]furan-1,2-dicarboxylate (product 4K)

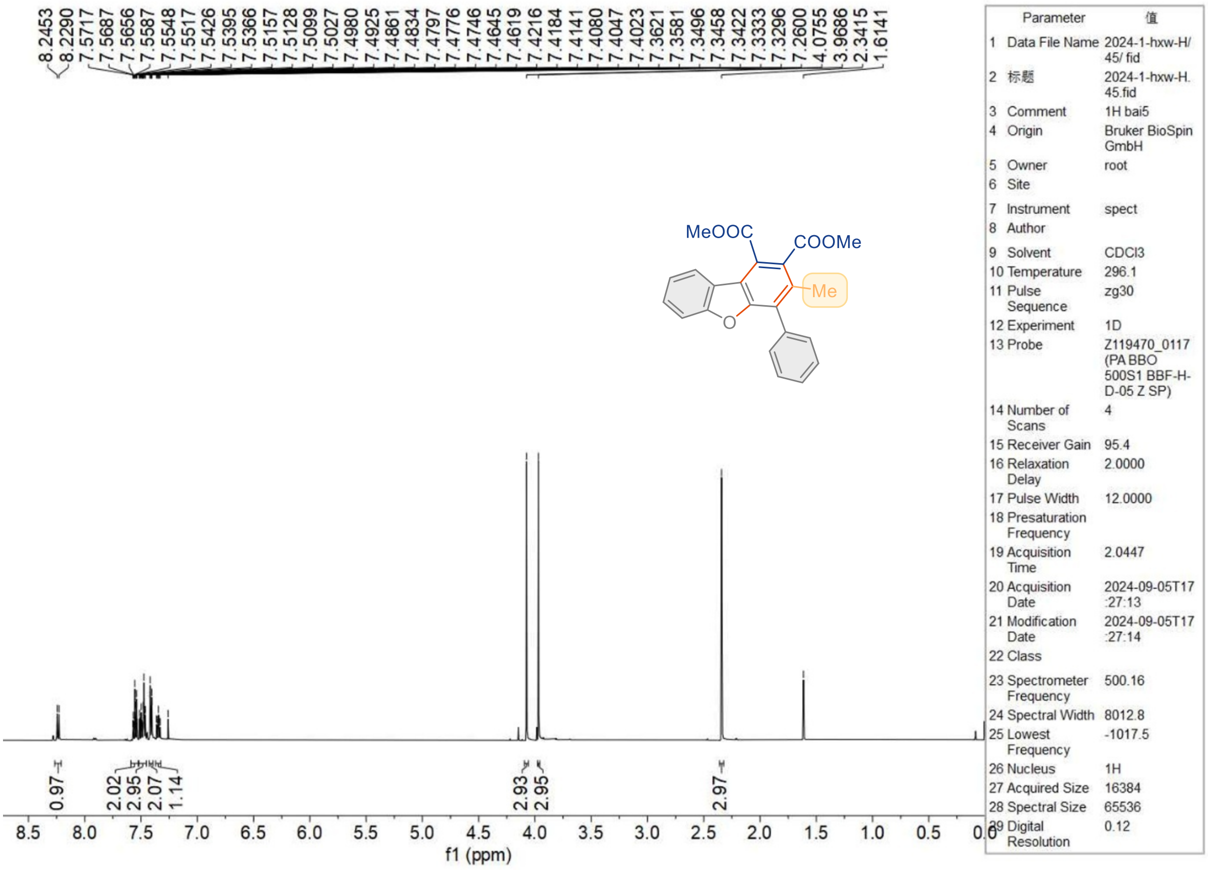

	Parameter	值
1	Data File Name	2024-1-hxw-H/ 45/ fid
2	标题	2024-1-hxw-H. 45.fid
3	Comment	1H bai5
4	Origin	Bruker BioSpin GmbH
5	Owner	root
6	Site	
7	Instrument	spect
8	Author	
9	Solvent	CDCl3
10	Temperature	296.1
11	Pulse Sequence	zg30
12	Experiment	1D
13	Probe	Z119470_0117 (PA BBO 500S1 BBF-H-D-05 Z SP)
14	Number of Scans	4
15	Receiver Gain	95.4
16	Relaxation Delay	2.0000
17	Pulse Width	12.0000
18	Presaturation Frequency	
19	Acquisition Time	2.0447
20	Acquisition Date	2024-09-05T17:27:13
21	Modification Date	2024-09-05T17:27:14
22	Class	
23	Spectrometer Frequency	500.16
24	Spectral Width	8012.8
25	Lowest Frequency	-1017.5
26	Nucleus	1H
27	Acquired Size	16384
28	Spectral Size	65536
29	Digital Resolution	0.12

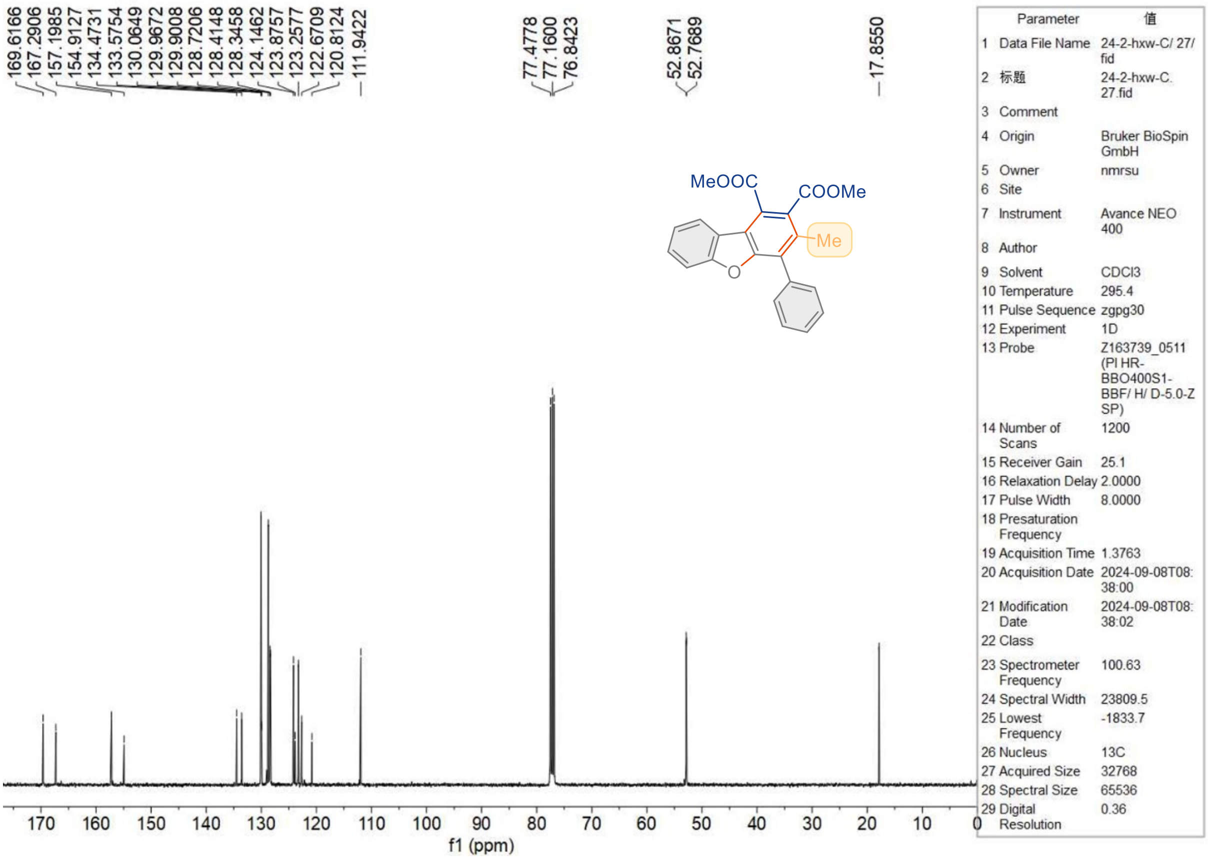

	Parameter	值
1	Data File Name	24-2-hxw-C/ 27/ fid
2	标题	24-2-hxw-C. 27.fid
3	Comment	
4	Origin	Bruker BioSpin GmbH
5	Owner	nmrsu
6	Site	
7	Instrument	Avance NEO 400
8	Author	
9	Solvent	CDCl3
10	Temperature	295.4
11	Pulse Sequence	zgpg30
12	Experiment	1D
13	Probe	Z163739_0511 (PI HR-BBO400S1-BBF/ H/ D-5.0-Z SP)
14	Number of Scans	1200
15	Receiver Gain	25.1
16	Relaxation Delay	2.0000
17	Pulse Width	8.0000
18	Presaturation Frequency	
19	Acquisition Time	1.3763
20	Acquisition Date	2024-09-08T08:38:00
21	Modification Date	2024-09-08T08:38:02
22	Class	
23	Spectrometer Frequency	100.63
24	Spectral Width	23809.5
25	Lowest Frequency	-1833.7
26	Nucleus	13C
27	Acquired Size	32768
28	Spectral Size	65536
29	Digital Resolution	0.36

Dimethyl 3-ethyl-4-phenyldibenzo[*b*,*d*]furan-1,2-dicarboxylate (product 4L)

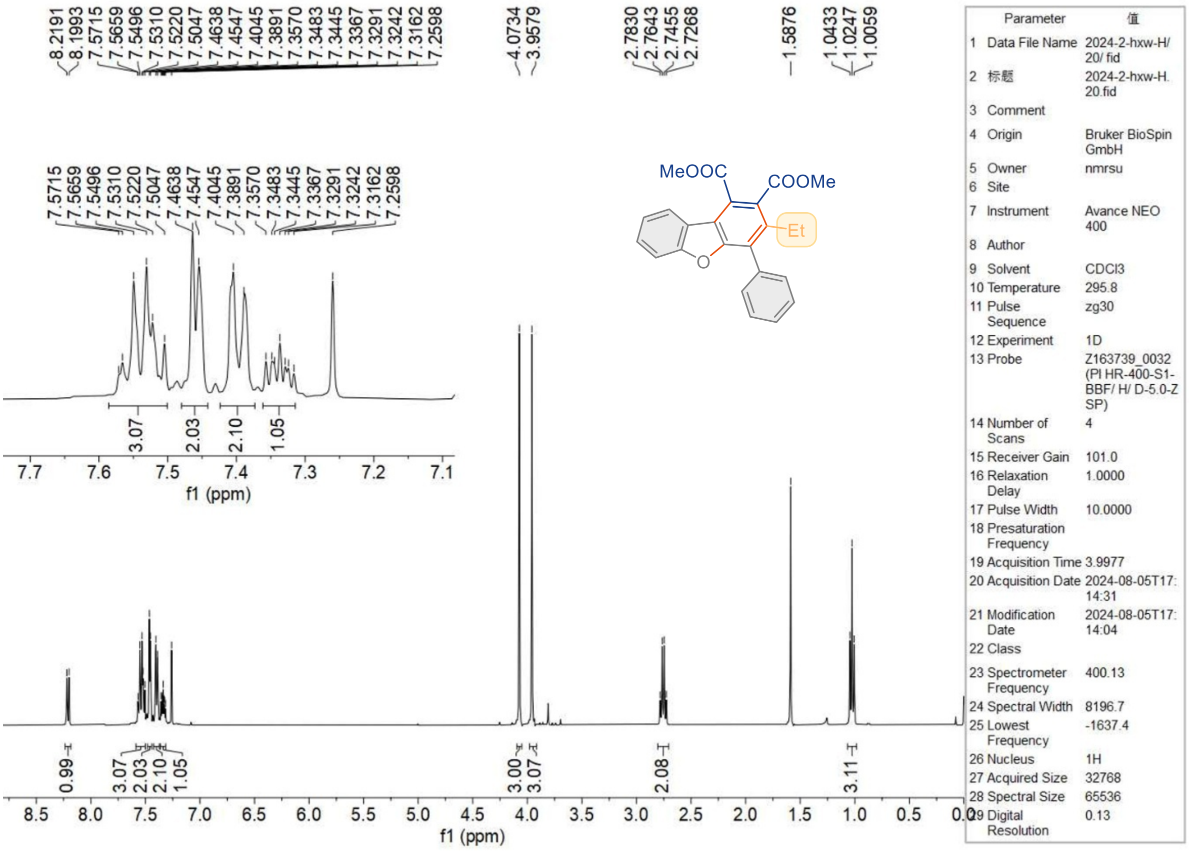

	Parameter	值
1	Data File Name	2024-2-hxw-H/20/ fid
2	标题	2024-2-hxw-H.20.fid
3	Comment	
4	Origin	Bruker BioSpin GmbH
5	Owner	nmrsu
6	Site	
7	Instrument	Avance NEO 400
8	Author	
9	Solvent	CDCl3
10	Temperature	295.8
11	Pulse Sequence	zg30
12	Experiment	1D
13	Probe	Z163739_0032 (PI HR-400-S1-BBF/ H/ D-5.0-Z SP)
14	Number of Scans	4
15	Receiver Gain	101.0
16	Relaxation Delay	1.0000
17	Pulse Width	10.0000
18	Presaturation Frequency	
19	Acquisition Time	3.9977
20	Acquisition Date	2024-08-05T17:14:31
21	Modification Date	2024-08-05T17:14:04
22	Class	
23	Spectrometer Frequency	400.13
24	Spectral Width	8196.7
25	Lowest Frequency	-1637.4
26	Nucleus	1H
27	Acquired Size	32768
28	Spectral Size	65536
29	Digital Resolution	0.13

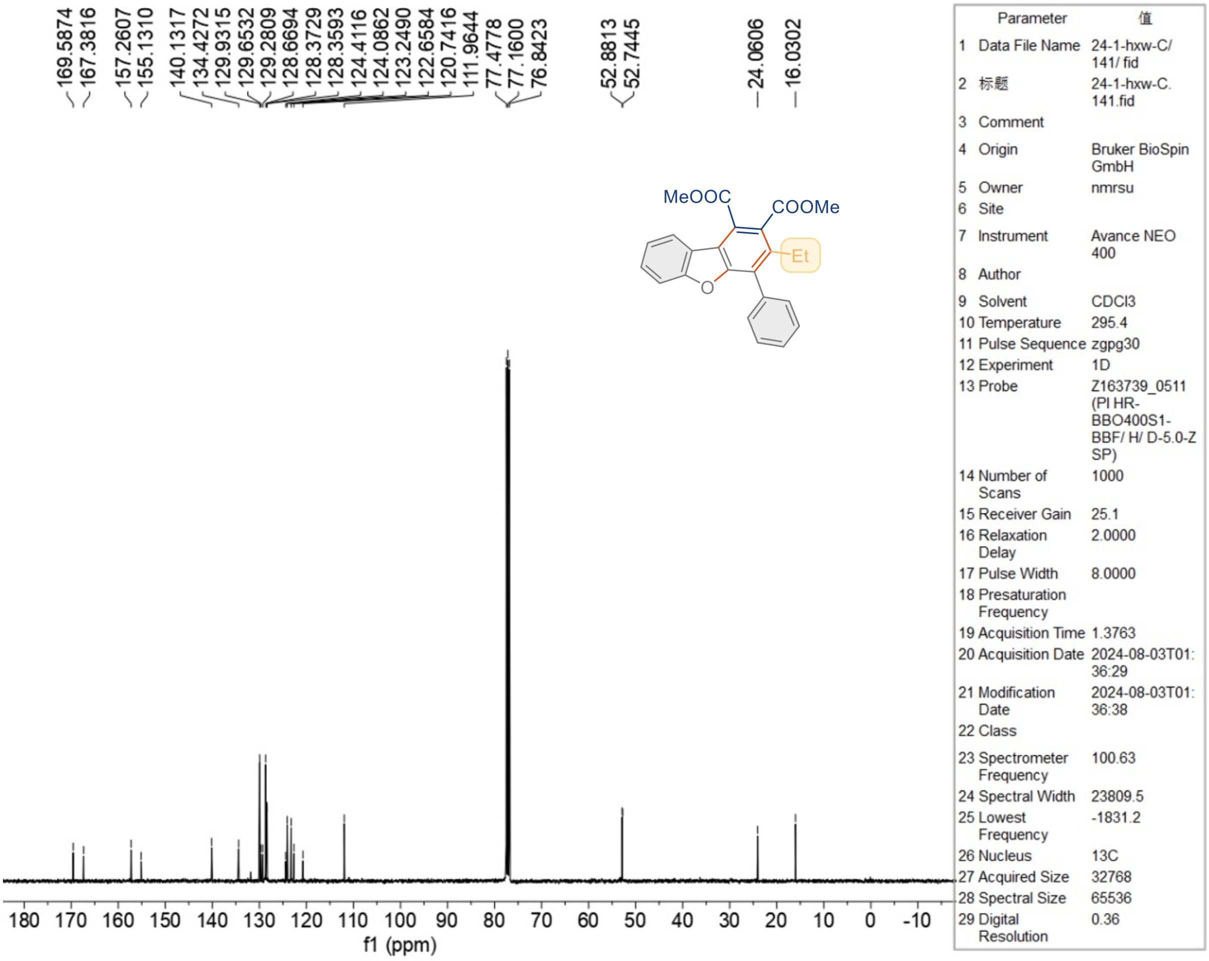

	Parameter	值
1	Data File Name	24-1-hxw-C/141/ fid
2	标题	24-1-hxw-C.141.fid
3	Comment	
4	Origin	Bruker BioSpin GmbH
5	Owner	nmrsu
6	Site	
7	Instrument	Avance NEO 400
8	Author	
9	Solvent	CDCl3
10	Temperature	295.4
11	Pulse Sequence	zgpg30
12	Experiment	1D
13	Probe	Z163739_0511 (PI HR-BBO400S1-BBF/ H/ D-5.0-Z SP)
14	Number of Scans	1000
15	Receiver Gain	25.1
16	Relaxation Delay	2.0000
17	Pulse Width	8.0000
18	Presaturation Frequency	
19	Acquisition Time	1.3763
20	Acquisition Date	2024-08-03T01:36:29
21	Modification Date	2024-08-03T01:36:38
22	Class	
23	Spectrometer Frequency	100.63
24	Spectral Width	23809.5
25	Lowest Frequency	-1831.2
26	Nucleus	13C
27	Acquired Size	32768
28	Spectral Size	65536
29	Digital Resolution	0.36

3-Ethyl 1,2-dimethyl 4-phenyldibenzo[*b,d*]furan-1,2,3-tricarboxylate (product 4M)

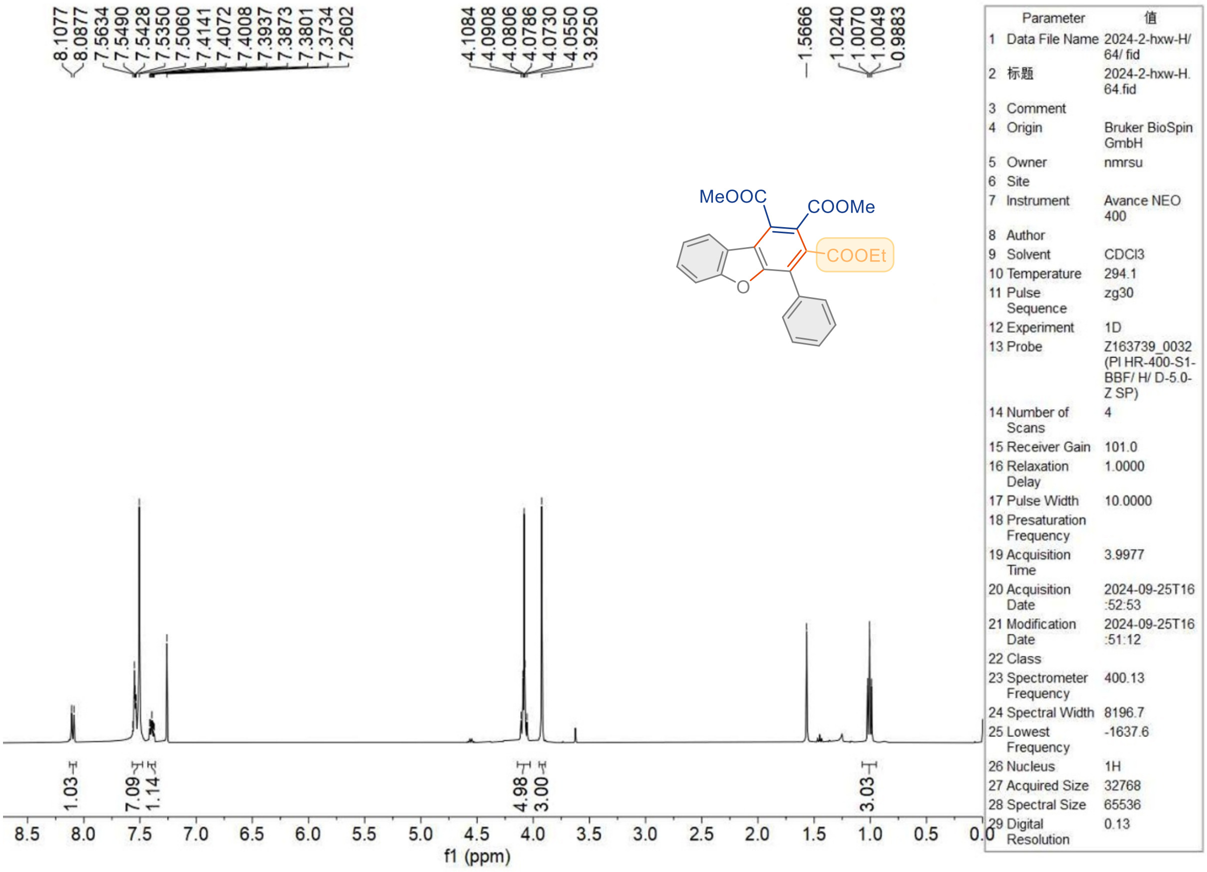

	Parameter	值
1	Data File Name	2024-2-hxw-H/64/ fid
2	标题	2024-2-hxw-H.64.fid
3	Comment	
4	Origin	Bruker BioSpin GmbH
5	Owner	nmrsu
6	Site	
7	Instrument	Avance NEO 400
8	Author	
9	Solvent	CDCl3
10	Temperature	294.1
11	Pulse Sequence	zg30
12	Experiment	1D
13	Probe	Z163739_0032 (PI HR-400-S1-BBF/ H/ D-5.0-Z SP)
14	Number of Scans	4
15	Receiver Gain	101.0
16	Relaxation Delay	1.0000
17	Pulse Width	10.0000
18	Presaturation Frequency	
19	Acquisition Time	3.9977
20	Acquisition Date	2024-09-25T16:52:53
21	Modification Date	2024-09-25T16:51:12
22	Class	
23	Spectrometer Frequency	400.13
24	Spectral Width	8196.7
25	Lowest Frequency	-1637.6
26	Nucleus	1H
27	Acquired Size	32768
28	Spectral Size	65536
29	Digital Resolution	0.13

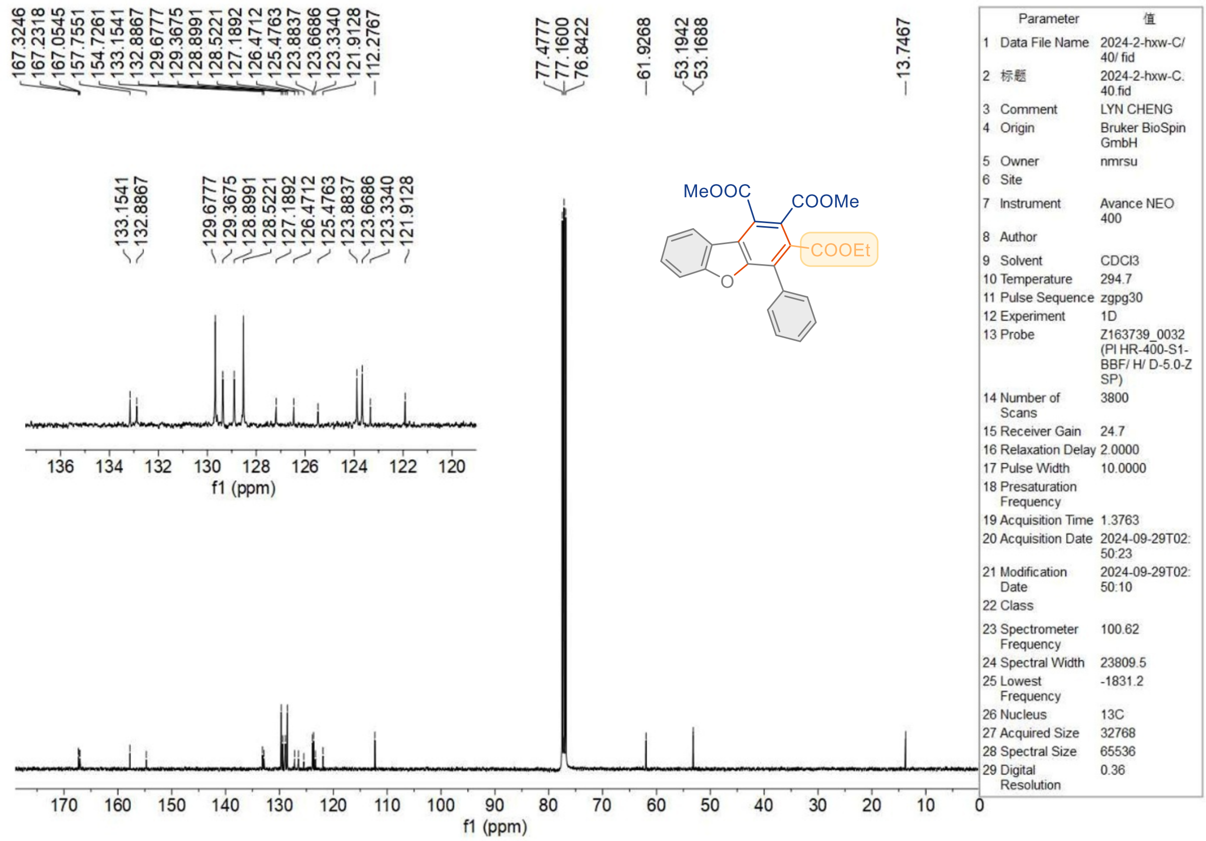

	Parameter	值
1	Data File Name	2024-2-hxw-C/40/ fid
2	标题	2024-2-hxw-C.40.fid
3	Comment	LYN CHENG
4	Origin	Bruker BioSpin GmbH
5	Owner	nmrsu
6	Site	
7	Instrument	Avance NEO 400
8	Author	
9	Solvent	CDCl3
10	Temperature	294.7
11	Pulse Sequence	zgpg30
12	Experiment	1D
13	Probe	Z163739_0032 (PI HR-400-S1-BBF/ H/ D-5.0-Z SP)
14	Number of Scans	3800
15	Receiver Gain	24.7
16	Relaxation Delay	2.0000
17	Pulse Width	10.0000
18	Presaturation Frequency	
19	Acquisition Time	1.3763
20	Acquisition Date	2024-09-29T02:50:23
21	Modification Date	2024-09-29T02:50:10
22	Class	
23	Spectrometer Frequency	100.62
24	Spectral Width	23809.5
25	Lowest Frequency	-1831.2
26	Nucleus	13C
27	Acquired Size	32768
28	Spectral Size	65536
29	Digital Resolution	0.36

Methyl 1-oxo-4-phenyl-1,3-dihydro-1*H*-benzofuro[2,3-f]isobenzofuran-10-carboxylate (product 4N)

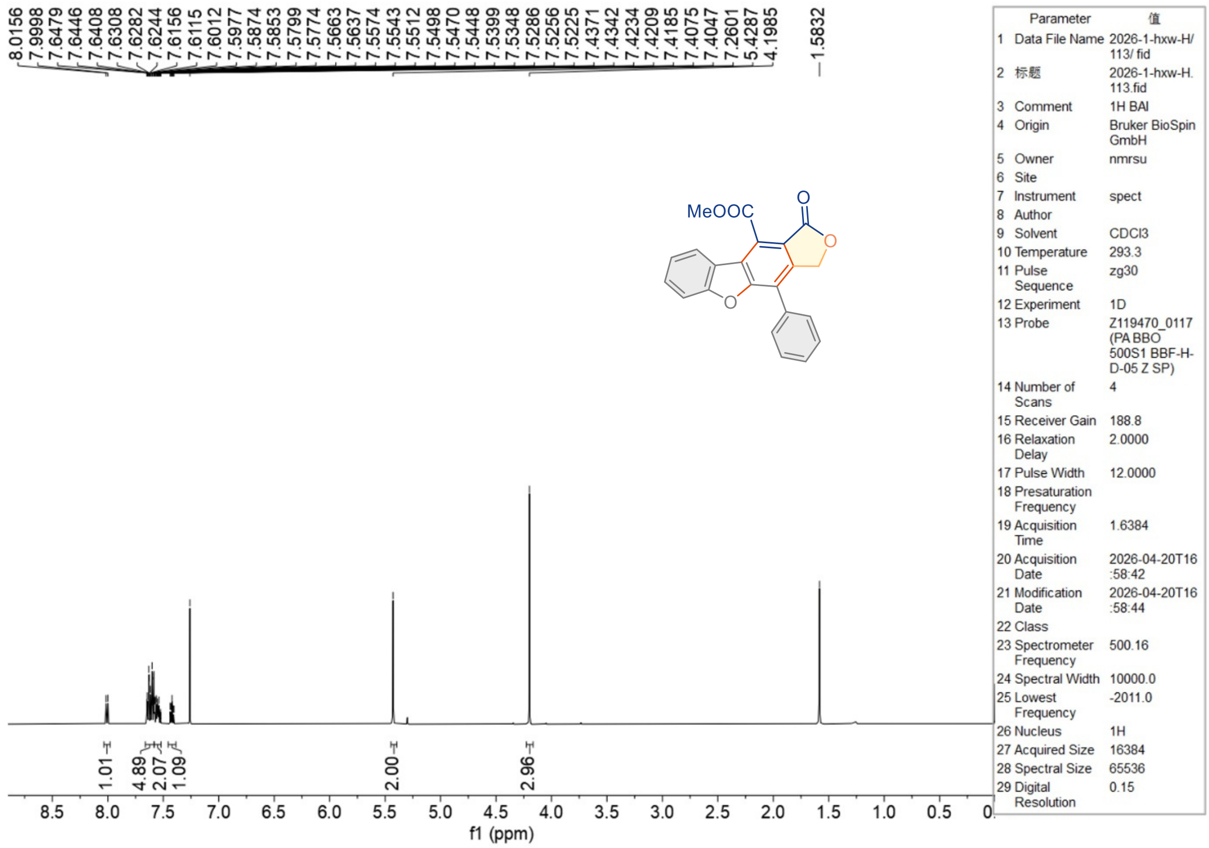

Parameter	值
1 Data File Name	2026-1-hxw-H/113/ fid
2 标题	2026-1-hxw-H.113.fid
3 Comment	1H BAI
4 Origin	Bruker BioSpin GmbH
5 Owner	nmrsu
6 Site	
7 Instrument	spect
8 Author	
9 Solvent	CDCl3
10 Temperature	293.3
11 Pulse Sequence	zg30
12 Experiment	1D
13 Probe	Z119470_0117 (PA BBO 500S1 BBF-H-D-05 Z SP)
14 Number of Scans	4
15 Receiver Gain	188.8
16 Relaxation Delay	2.0000
17 Pulse Width	12.0000
18 Presaturation Frequency	
19 Acquisition Time	1.6384
20 Acquisition Date	2026-04-20T16:58:42
21 Modification Date	2026-04-20T16:58:44
22 Class	
23 Spectrometer Frequency	500.16
24 Spectral Width	10000.0
25 Lowest Frequency	-2011.0
26 Nucleus	1H
27 Acquired Size	16384
28 Spectral Size	65536
29 Digital Resolution	0.15

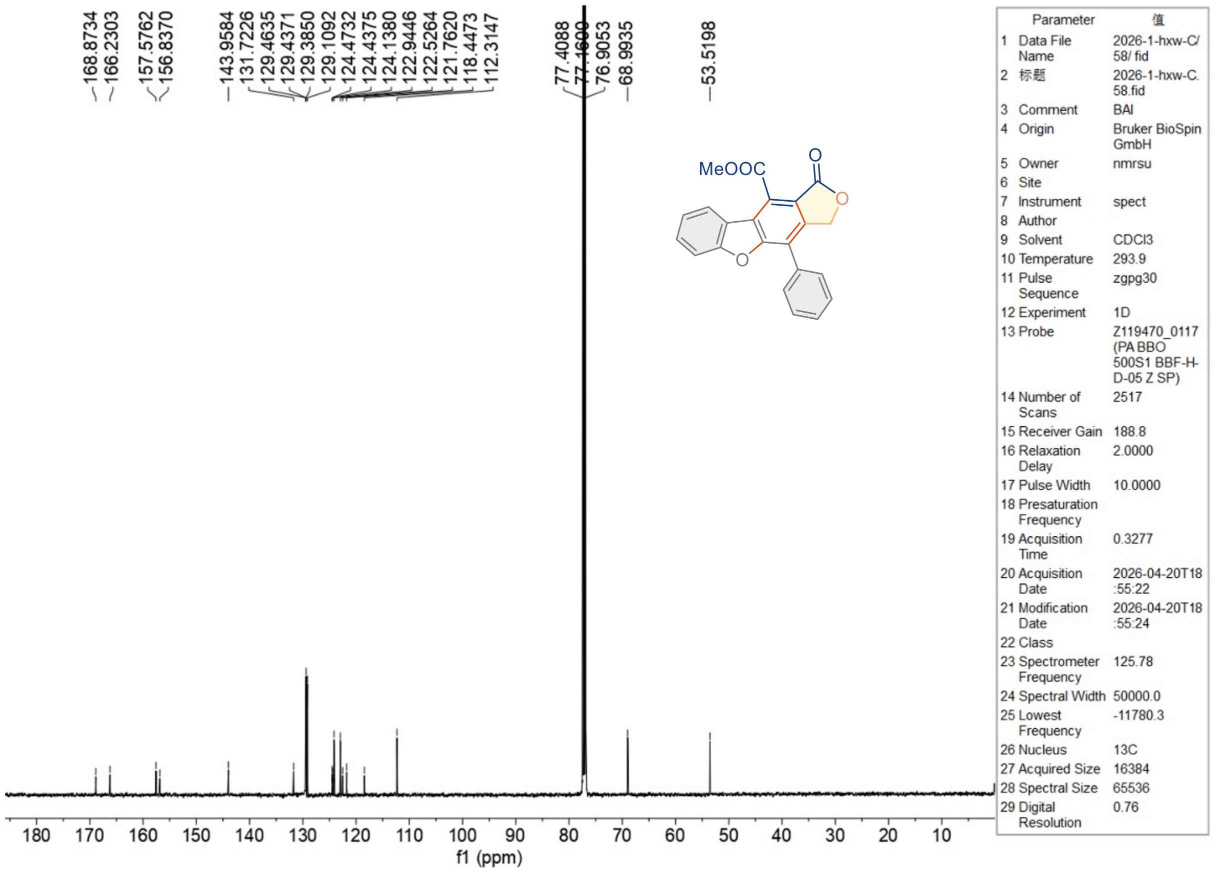

Parameter	值
1 Data File Name	2026-1-hxw-C/58/ fid
2 标题	2026-1-hxw-C.58.fid
3 Comment	BAI
4 Origin	Bruker BioSpin GmbH
5 Owner	nmrsu
6 Site	
7 Instrument	spect
8 Author	
9 Solvent	CDCl3
10 Temperature	293.9
11 Pulse Sequence	zgpg30
12 Experiment	1D
13 Probe	Z119470_0117 (PA BBO 500S1 BBF-H-D-05 Z SP)
14 Number of Scans	2517
15 Receiver Gain	188.8
16 Relaxation Delay	2.0000
17 Pulse Width	10.0000
18 Presaturation Frequency	
19 Acquisition Time	0.3277
20 Acquisition Date	2026-04-20T18:55:22
21 Modification Date	2026-04-20T18:55:24
22 Class	
23 Spectrometer Frequency	125.78
24 Spectral Width	50000.0
25 Lowest Frequency	-11780.3
26 Nucleus	13C
27 Acquired Size	16384
28 Spectral Size	65536
29 Digital Resolution	0.76

Methyl 1-oxo-5-phenyl-3,4-dihydro-1*H*-benzofuro[2,3-*g*]isochromene-11-carboxylate (product 4O)

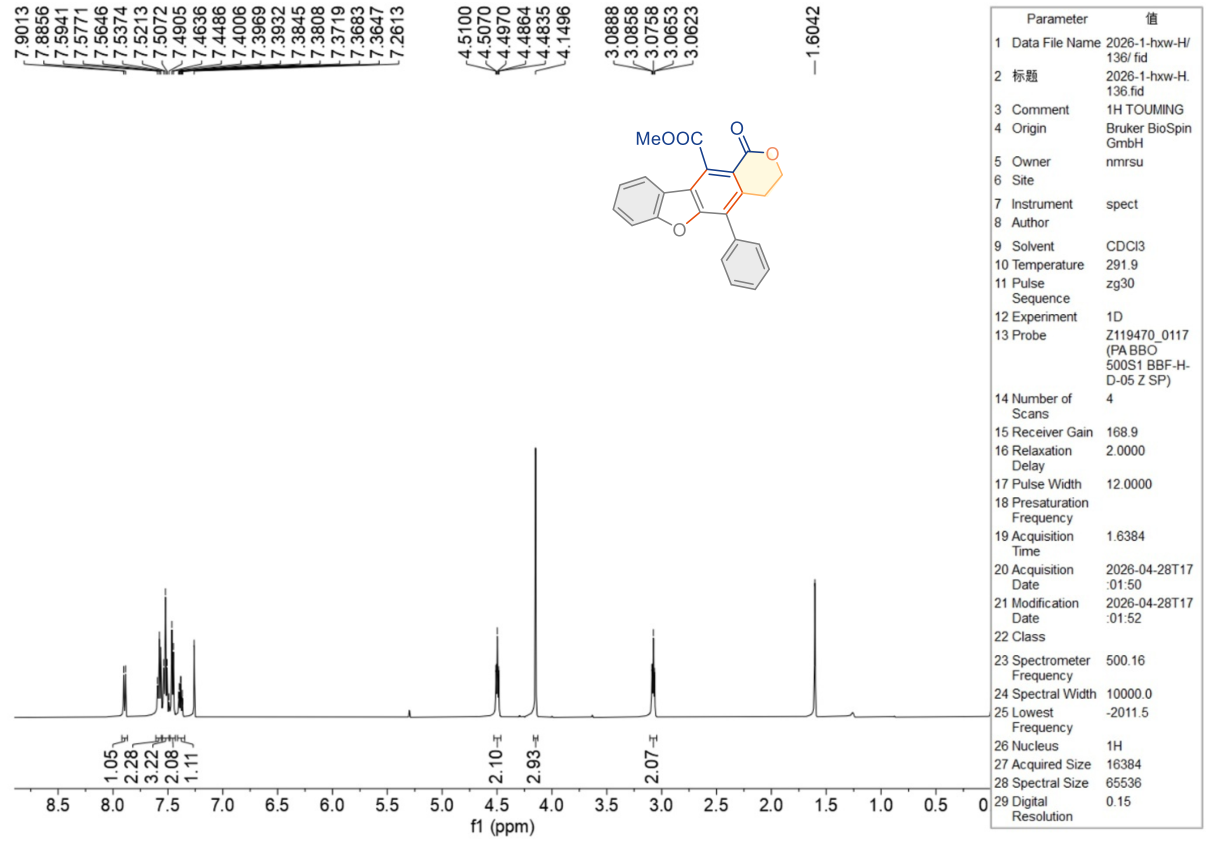

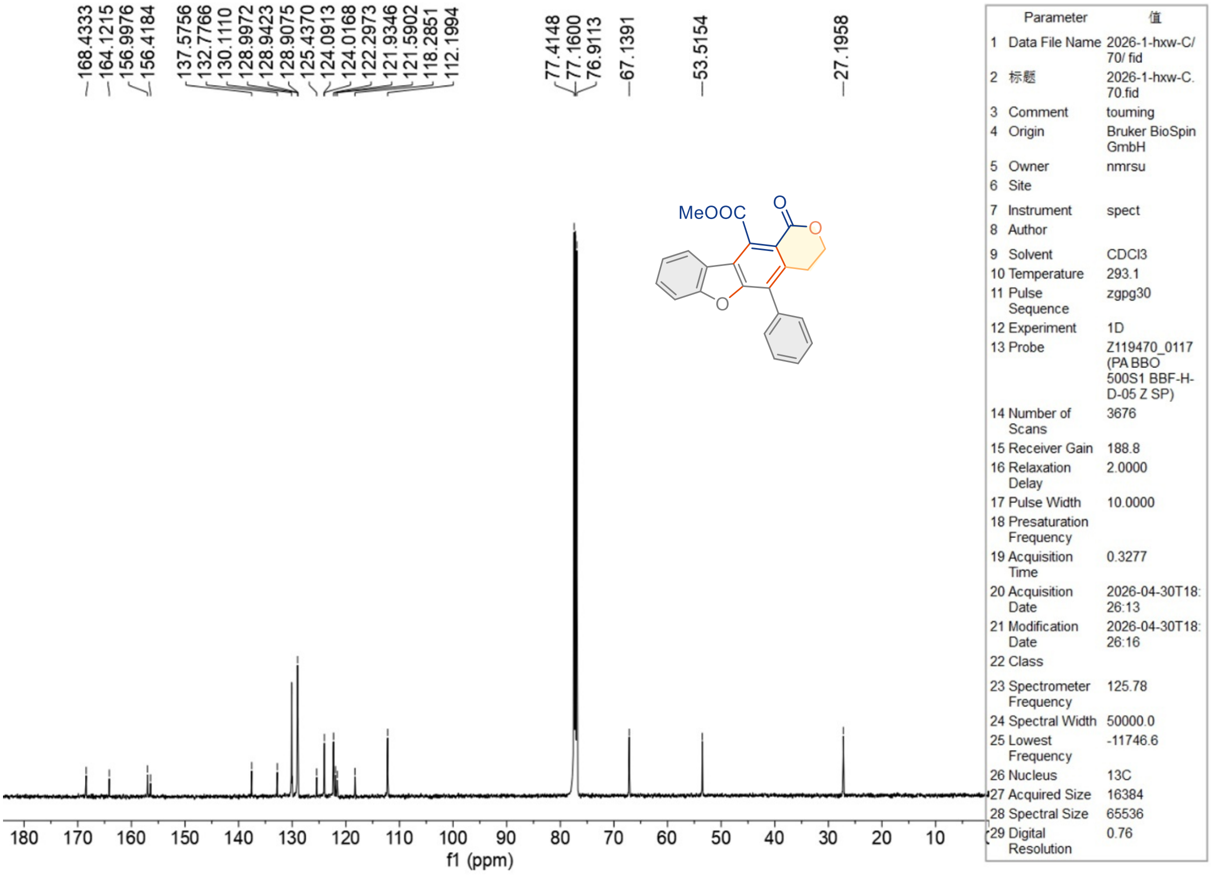

(4-Phenyldibenzo[*b,d*]furan-1,2-diyl)dimethanol (product 5)

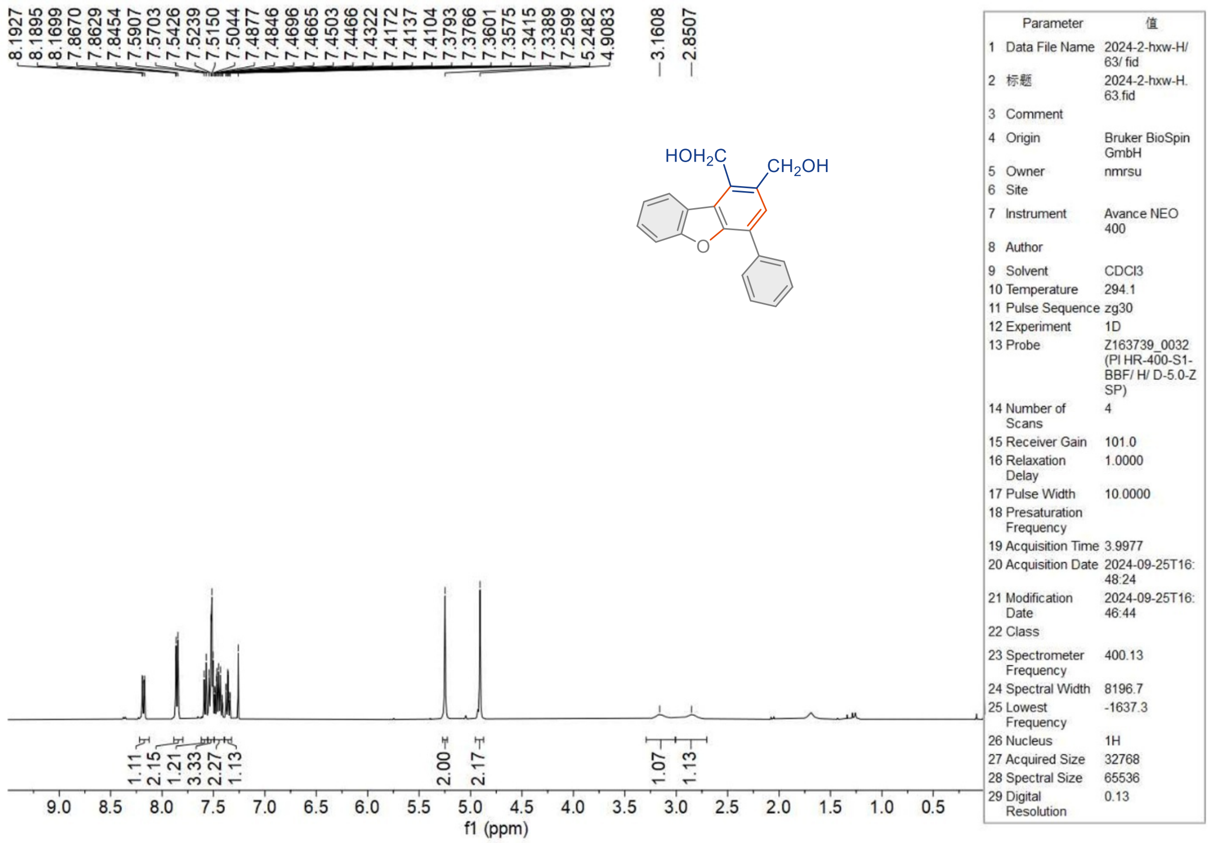

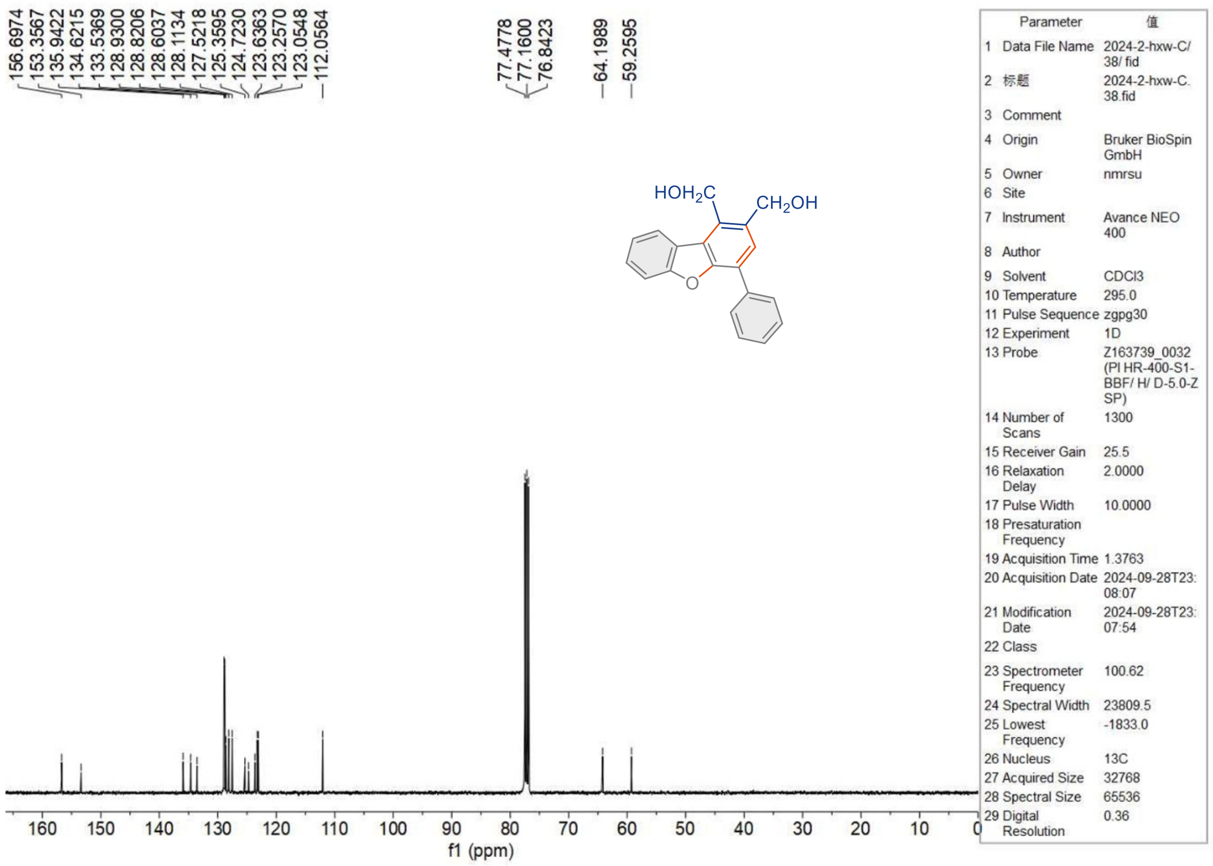

1-(Methoxycarbonyl)-4-phenyldibenzo[*b,d*]furan-2-carboxylic acid (product 6)

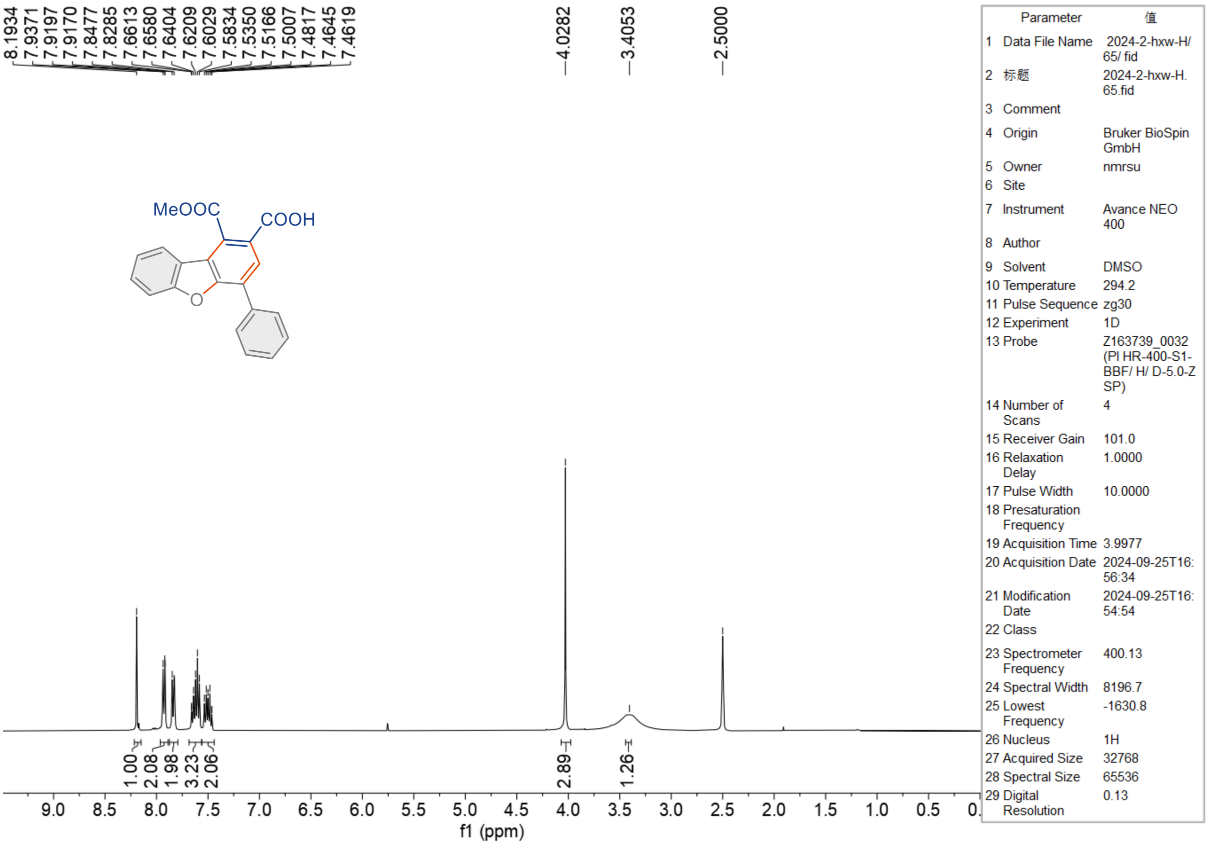

Parameter	值
1 Data File Name	2024-2-hxw-H/65/ fid
2 标题	2024-2-hxw-H.65.fid
3 Comment	
4 Origin	Bruker BioSpin GmbH
5 Owner	nmrsu
6 Site	
7 Instrument	Avance NEO 400
8 Author	
9 Solvent	DMSO
10 Temperature	294.2
11 Pulse Sequence	zg30
12 Experiment	1D
13 Probe	Z163739_0032 (PI HR-400-S1-BBF/ H/ D-5.0-Z SP)
14 Number of Scans	4
15 Receiver Gain	101.0
16 Relaxation Delay	1.0000
17 Pulse Width	10.0000
18 Presaturation Frequency	
19 Acquisition Time	3.9977
20 Acquisition Date	2024-09-25T16:56:34
21 Modification Date	2024-09-25T16:54:54
22 Class	
23 Spectrometer Frequency	400.13
24 Spectral Width	8196.7
25 Lowest Frequency	-1630.8
26 Nucleus	1H
27 Acquired Size	32768
28 Spectral Size	65536
29 Digital Resolution	0.13

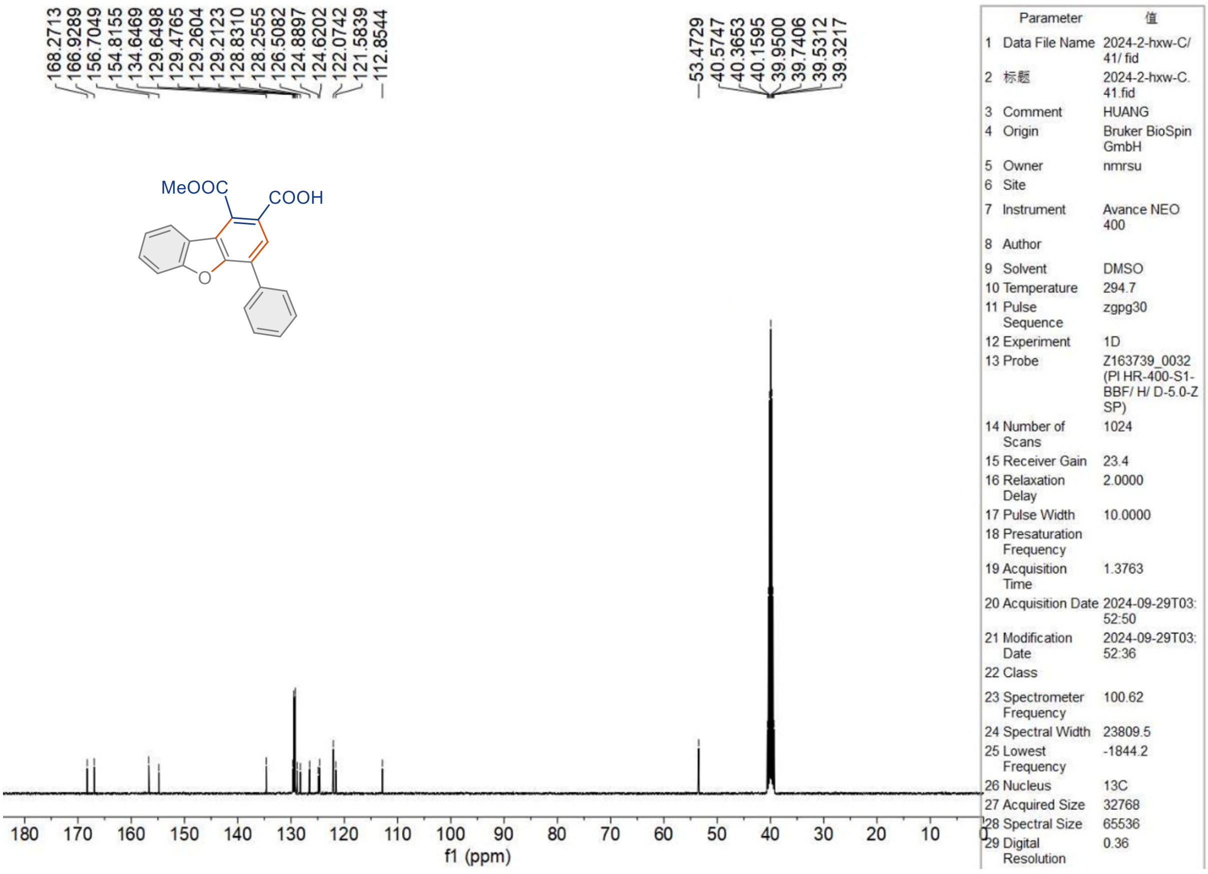

Parameter	值
1 Data File Name	2024-2-hxw-C/41/ fid
2 标题	2024-2-hxw-C.41.fid
3 Comment	HUANG
4 Origin	Bruker BioSpin GmbH
5 Owner	nmrsu
6 Site	
7 Instrument	Avance NEO 400
8 Author	
9 Solvent	DMSO
10 Temperature	294.7
11 Pulse Sequence	zgpg30
12 Experiment	1D
13 Probe	Z163739_0032 (PI HR-400-S1-BBF/ H/ D-5.0-Z SP)
14 Number of Scans	1024
15 Receiver Gain	23.4
16 Relaxation Delay	2.0000
17 Pulse Width	10.0000
18 Presaturation Frequency	
19 Acquisition Time	1.3763
20 Acquisition Date	2024-09-29T03:52:50
21 Modification Date	2024-09-29T03:52:36
22 Class	
23 Spectrometer Frequency	100.62
24 Spectral Width	23809.5
25 Lowest Frequency	-1844.2
26 Nucleus	13C
27 Acquired Size	32768
28 Spectral Size	65536
29 Digital Resolution	0.36

1,3,3-Trimethyl-5-phenyl-isobenzofuro[5,4-*b*]benzofuran-1-ol (product 7)

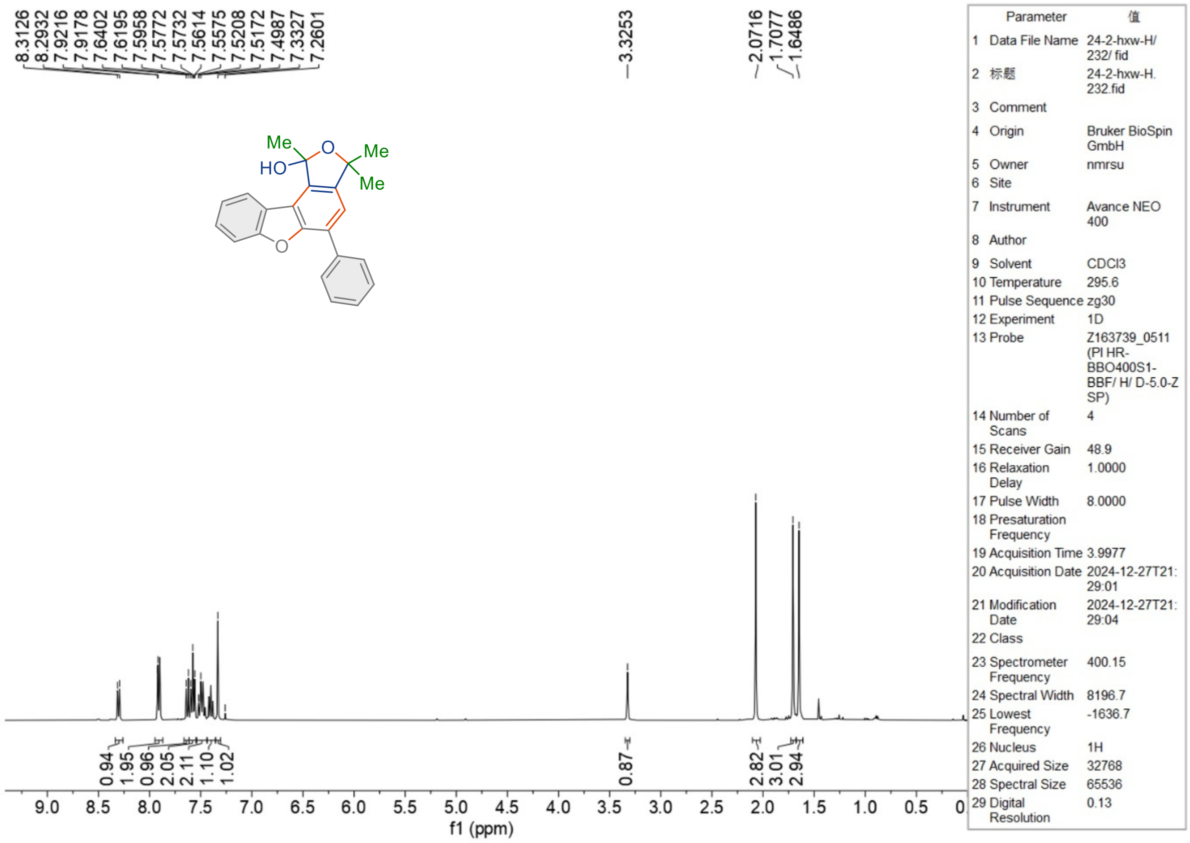

Parameter	值
1 Data File Name	24-2-hxw-H/ 232/ fid
2 标题	24-2-hxw-H. 232.fid
3 Comment	
4 Origin	Bruker BioSpin GmbH
5 Owner	nmrsu
6 Site	
7 Instrument	Avance NEO 400
8 Author	
9 Solvent	CDCl3
10 Temperature	295.6
11 Pulse Sequence	zg30
12 Experiment	1D
13 Probe	Z163739_0511 (PI HR-BBO400S1-BBF/ H/ D-5.0-Z SP)
14 Number of Scans	4
15 Receiver Gain	48.9
16 Relaxation Delay	1.0000
17 Pulse Width	8.0000
18 Presaturation Frequency	
19 Acquisition Time	3.9977
20 Acquisition Date	2024-12-27T21:29:01
21 Modification Date	2024-12-27T21:29:04
22 Class	
23 Spectrometer Frequency	400.15
24 Spectral Width	8196.7
25 Lowest Frequency	-1636.7
26 Nucleus	1H
27 Acquired Size	32768
28 Spectral Size	65536
29 Digital Resolution	0.13

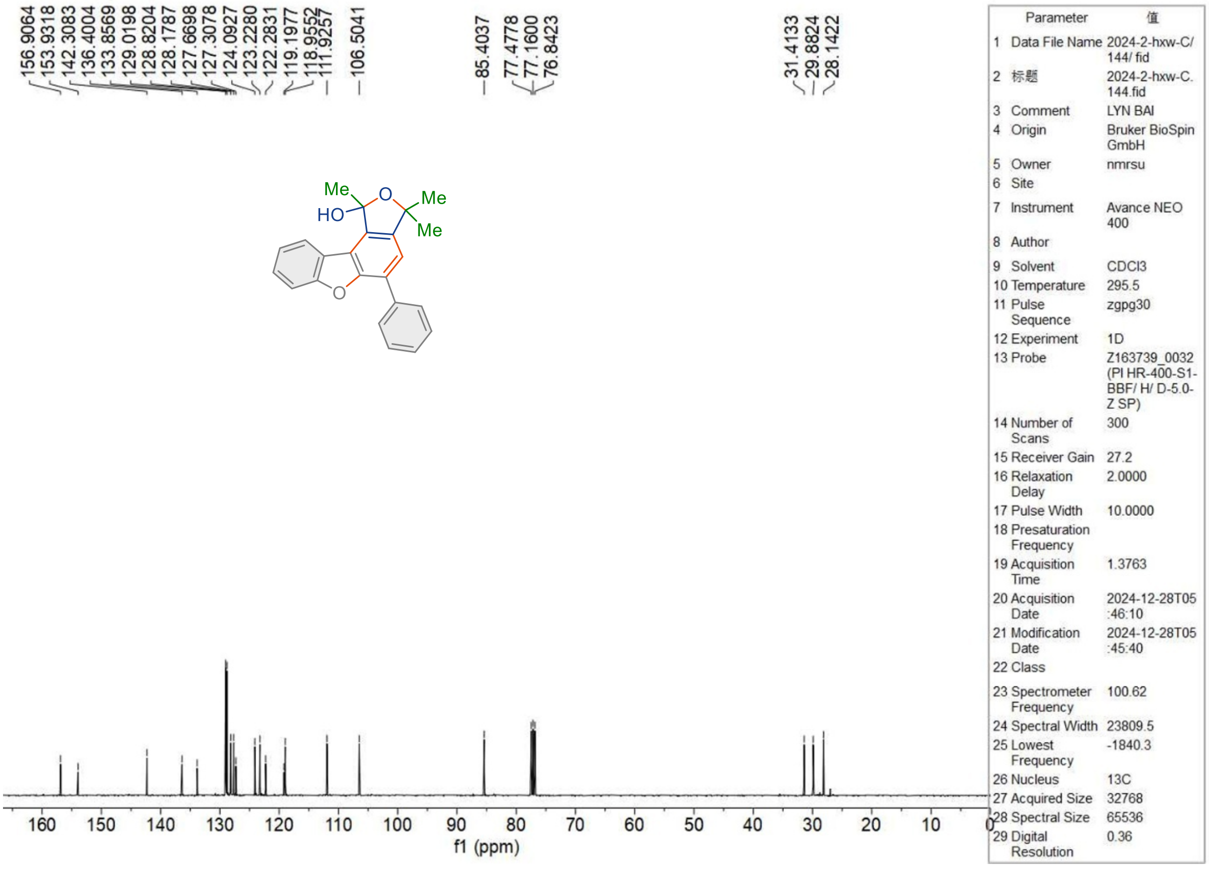

Parameter	值
1 Data File Name	2024-2-hxw-C/ 144/ fid
2 标题	2024-2-hxw-C. 144.fid
3 Comment	LYN BAI
4 Origin	Bruker BioSpin GmbH
5 Owner	nmrsu
6 Site	
7 Instrument	Avance NEO 400
8 Author	
9 Solvent	CDCl3
10 Temperature	295.5
11 Pulse Sequence	zgpg30
12 Experiment	1D
13 Probe	Z163739_0032 (PI HR-400-S1-BBF/ H/ D-5.0-Z SP)
14 Number of Scans	300
15 Receiver Gain	27.2
16 Relaxation Delay	2.0000
17 Pulse Width	10.0000
18 Presaturation Frequency	
19 Acquisition Time	1.3763
20 Acquisition Date	2024-12-28T05:46:10
21 Modification Date	2024-12-28T05:45:40
22 Class	
23 Spectrometer Frequency	100.62
24 Spectral Width	23809.5
25 Lowest Frequency	-1840.3
26 Nucleus	13C
27 Acquired Size	32768
28 Spectral Size	65536
29 Digital Resolution	0.36

Dimethyl 4-phenyl-8-(phenylethynyl)dibenzo[*b,d*]furan-1,2-dicarboxylate (product 8)

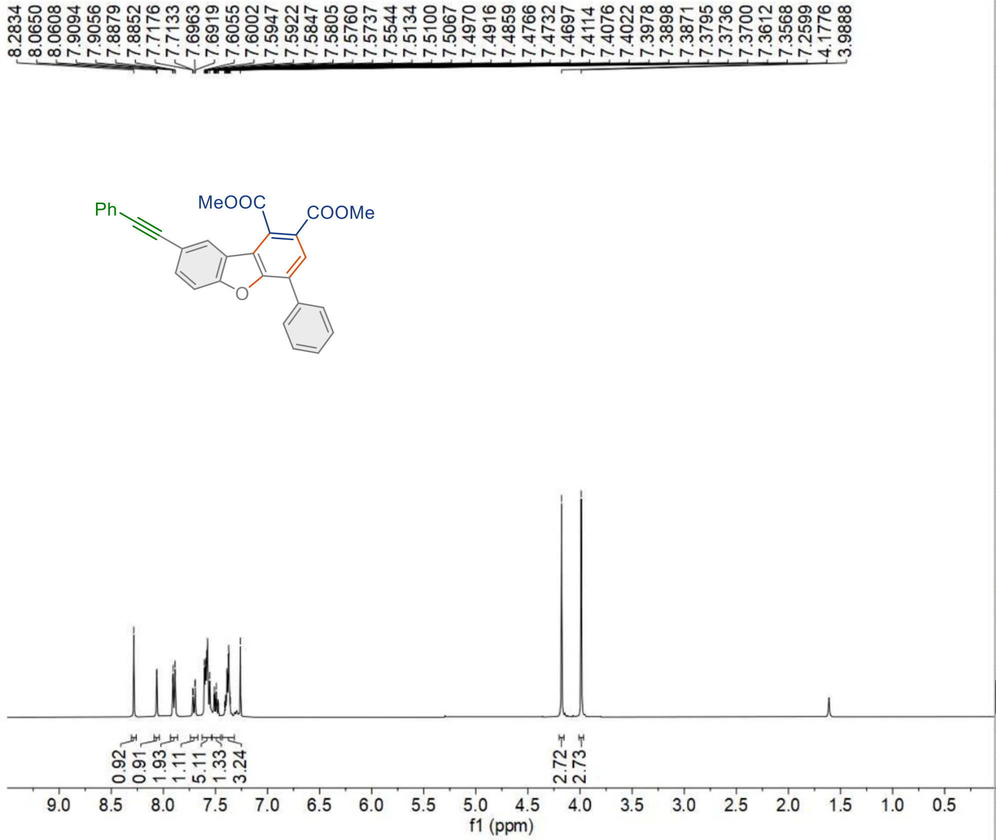

Parameter	值
1 Data File Name	2024-2-hxw-H/ 92/ fid
2 标题	2024-2-hxw-H. 92.fid
3 Comment	
4 Origin	Bruker BioSpin GmbH
5 Owner	nmrsu
6 Site	
7 Instrument	Avance NEO 400
8 Author	
9 Solvent	CDCl3
10 Temperature	294.1
11 Pulse Sequence	zg30
12 Experiment	1D
13 Probe	Z163739_0032 (PI HR-400-S1-BBF/ H/ D-5.0-Z SP)
14 Number of Scans	4
15 Receiver Gain	101.0
16 Relaxation Delay	1.0000
17 Pulse Width	10.0000
18 Presaturation Frequency	
19 Acquisition Time	3.9977
20 Acquisition Date	2024-10-18T22:53:23
21 Modification Date	2024-10-18T22:53:06
22 Class	
23 Spectrometer Frequency	400.13
24 Spectral Width	8196.7
25 Lowest Frequency	-1637.3
26 Nucleus	1H
27 Acquired Size	32768
28 Spectral Size	65536
29 Digital Resolution	0.13

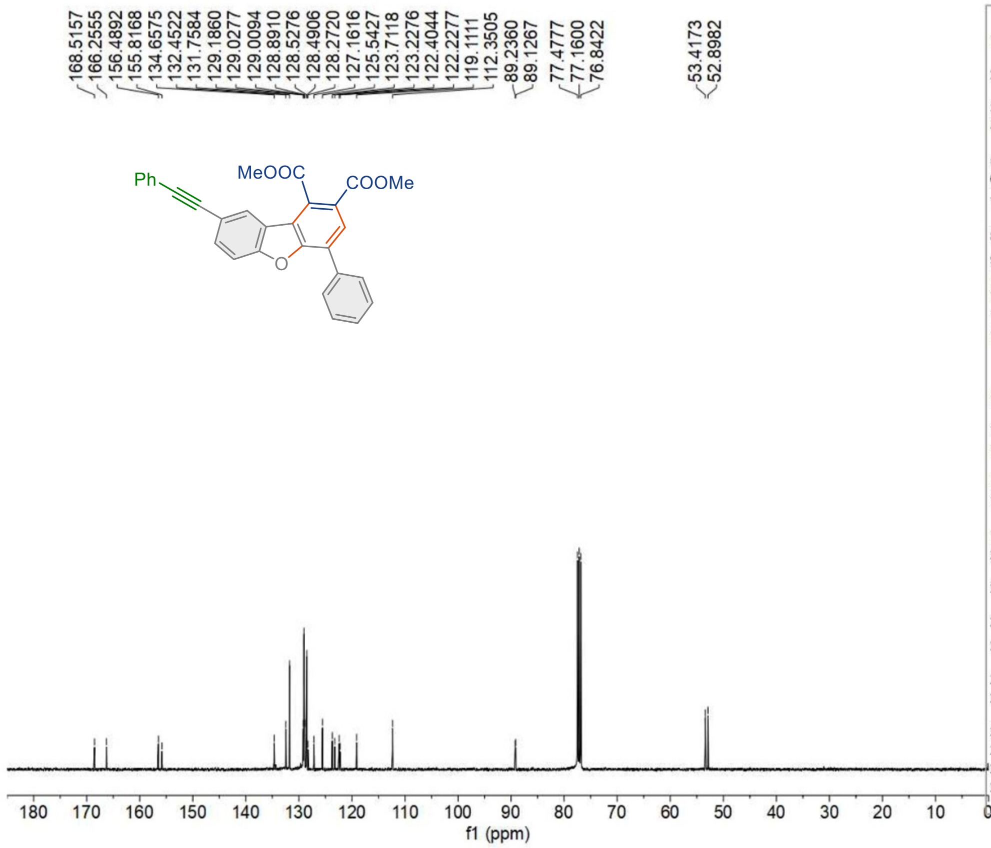

Parameter	值
1 Data File Name	2024-2-hxw-C/ 63/ fid
2 标题	2024-2-hxw-C. 63.fid
3 Comment	HONG
4 Origin	Bruker BioSpin GmbH
5 Owner	nmrsu
6 Site	
7 Instrument	Avance NEO 400
8 Author	
9 Solvent	CDCl3
10 Temperature	294.0
11 Pulse Sequence	zgpg30
12 Experiment	1D
13 Probe	Z163739_0032 (PI HR-400-S1-BBF/ H/ D-5.0-Z SP)
14 Number of Scans	1500
15 Receiver Gain	17.0
16 Relaxation Delay	2.0000
17 Pulse Width	10.0000
18 Presaturation Frequency	
19 Acquisition Time	1.3763
20 Acquisition Date	2024-10-20T02:49:33
21 Modification Date	2024-10-20T02:49:08
22 Class	
23 Spectrometer Frequency	100.62
24 Spectral Width	23809.5
25 Lowest Frequency	-1834.5
26 Nucleus	13C
27 Acquired Size	32768
28 Spectral Size	65536
29 Digital Resolution	0.36

Dimethyl 4-phenyldibenzo[*b,d*]furan-1,2-dicarboxylate-3-*d* (product 3a-*d*)

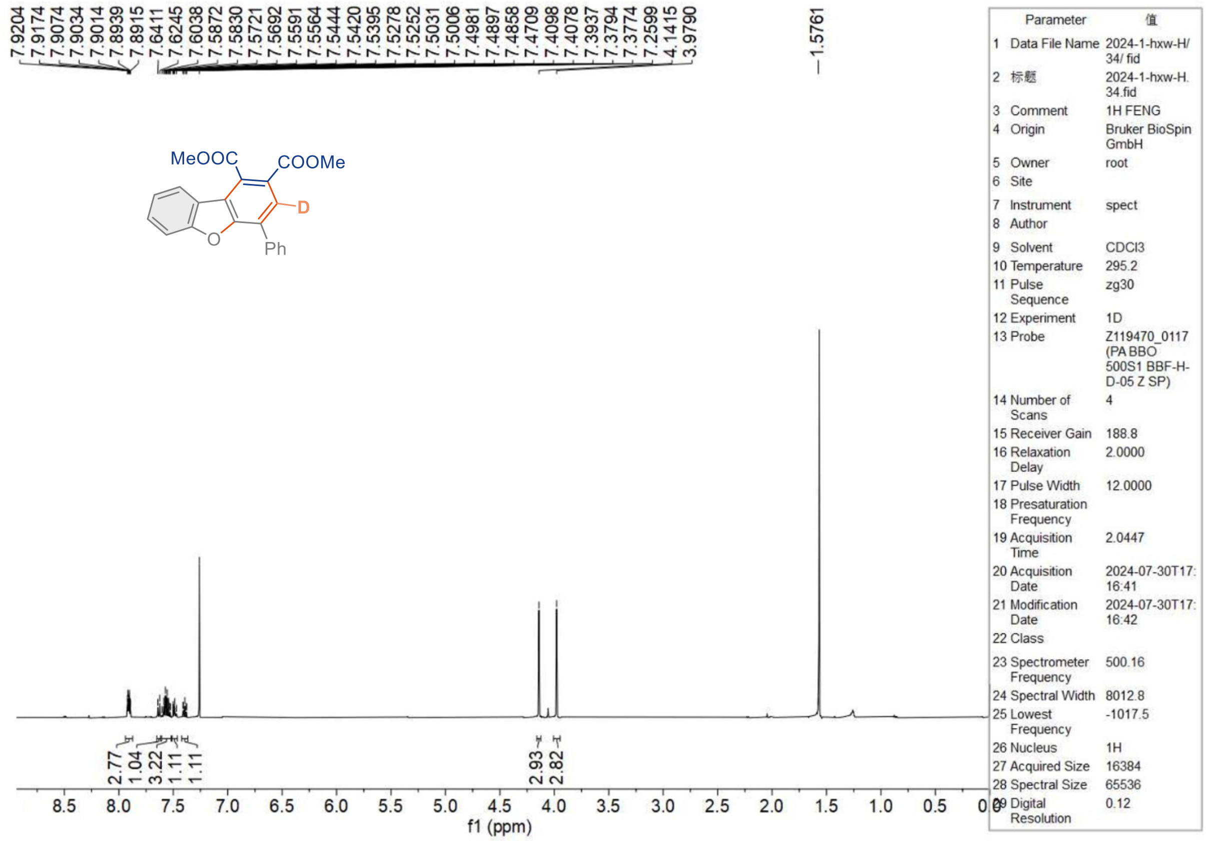

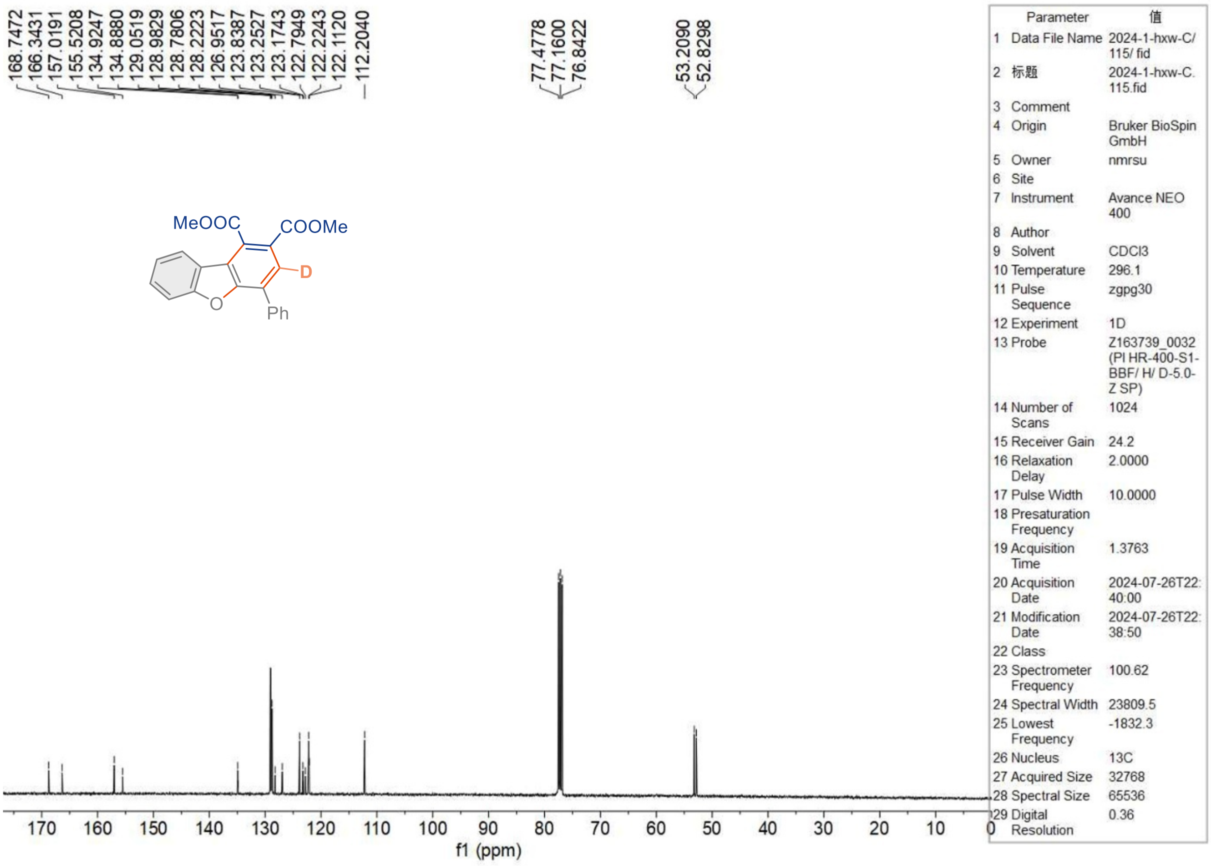

14. References

(1) He, X.; Xie, M.; Li, R.; Choy, P. Y.; Tang, Q.; Shang, Y.; Kwong, F. Y. A Organocatalytic Approach for Assembling Flavanones via a Cascade 1,4-Conjugate Addition/oxa-Michael Addition between Propargylamines with Water. *Org. Lett.* **2020**, *22*, 4306–4310.

(2) Díaz-Jiménez, À.; Kennington, S. C. D.; Roglans, A.; Pla-Quintana, A. Copper(I) Iodide Catalyzed [3 + 3] Annulation of Iodonium Ylides with Pyridinium 1,4-Zwitterionic Thiolates for the Synthesis of 1,4-Oxathiin Scaffolds. *Org. Lett.* **2023**, *25*, 4830–4834.

(3) Li, R.; Zhang, J.; Sun, M.; Wang, Z.; Yang, J. HFIP-Promoted Divergent Cycloadditions of Difluoroenoxysilanes with 2-Indolylmethanols: Synthesis of Fluoro 2H-Pyrano[3,4-b]indoles and gem-Difluoro Cyclopenta[*b*]indoles. *Org. Lett.,* **2024**, *26*, 4610-4615.

(4) Csékei, M.; Novák, Z.; Kotschy, A. Ethynyl-cyclohexanol: an efficient acetylene surrogate in Sonogashira coupling. *Tetrahedron* **2008**, *64*, 975–982.
